# Highly Rigid, Yet Conformationally Adaptable, Bisporphyrin *sp*^2^-Cage Receptors Afford Outstanding Binding Affinities, Chelate Cooperativities, and Substrate Selectivities

**DOI:** 10.1021/jacs.4c13756

**Published:** 2024-12-19

**Authors:** A. Priscila Gia, Alberto de Juan, Daniel Aranda, Fernando G. Guijarro, Juan Aragó, Enrique Ortí, Miguel García-Iglesias, David González-Rodríguez

**Affiliations:** † Nanostructured Molecular Systems and Materials group, Organic Chemistry Department, Universidad Autónoma de Madrid, Madrid 28049, Spain; ‡ Institute for Advanced Research in Chemical Sciences (IAdChem), Universidad Autónoma de Madrid, Madrid 28049, Spain; § Institute of Molecular Science, Universidad de Valencia, Catedrático José Beltrán 2, Paterna 46980, Spain; ∥ QUIPRE Department, Nanomedicine-IDIVAL, Universidad de Cantabria, Avd. de Los Castros, 46, Santander 39005, Spain

## Abstract

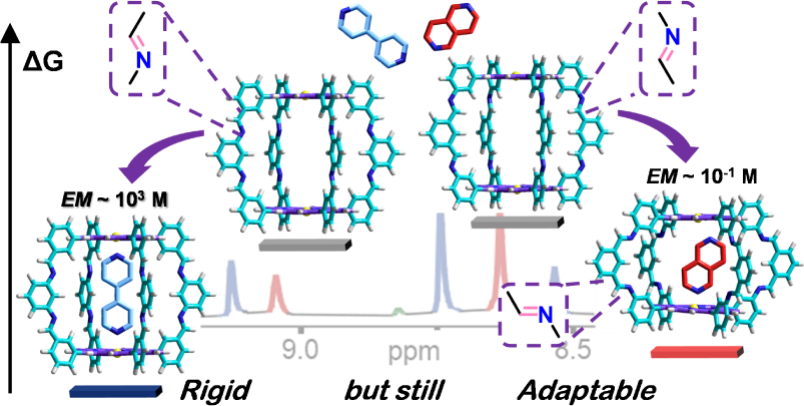

If we aim to develop efficient synthetic models of protein receptors and enzymes, we must understand the relationships of intra- and intermolecular interactions between hosts and guests and how they mutually influence their conformational energy landscape so as to adapt to each other to maximize binding energies and enhance substrate selectivities. Here, we introduce a novel design of cofacial (Zn^II^)bisporphyrin cages based on dynamic imine bonding, which is synthetically simple, but at the same time highly robust and versatile, affording receptors composed of only *sp*^2^-hybridized C and N atoms. The high structural rigidity of these cages renders them ideal hosts for ditopic molecules that can fit into the cavity and bind to both metal centers, leading to association constants as high as 10^9^ M^–1^ in chloroform. These strong binding affinities are a consequence of the remarkable chelate cooperativities attained, with effective molarity (*EM*) values reaching record values over 10^3^ M. However, we discovered that the cages can still adapt their structure to a more *compact* version, able to host slightly smaller guests. Such a conformational transition has an energy cost, which can be very different depending on the direction of the imine linkages in the cage skeleton and which results in *EM* values 2–3 orders of magnitude lower. This interplay between cooperativity and conformational adaptability leads to strong and unusual selectivities. Not only these metalloporphyrin receptors can choose to bind preferably to a particular guest, as a function of its size, but also the guest can select which host to bind, as a function now of the host’s conformational rigidity. Such highly cooperative and selective associations are lost, however, in related flexible receptors where the imine bonds are reduced.

## Introduction

The ability of some molecules to identify and bind tightly to specific substrates is a fascinating phenomenon and one of the key pillars of life, with notorious implications in biomedicine and sensing materials. A major challenge in the field of molecular recognition with synthetic receptors is to rival the association energies and substrate selectivities displayed by many protein–ligand complexes.^1−3^ One of the key tactics used to increase binding affinity by natural systems, and by competing synthetic receptors working in aqueous media,^4,5^ is to provide hydrophobic cavities that release “high-energy” water molecules upon desolvation, which results in a favorable entropic contribution to the free energy of binding.^6^ A second strategy, essential for achieving high selectivities and becoming even more important in nonaqueous solvents, is the decoration of the binding site with multiple, spatially preorganized noncovalent contacts that result in favorable enthalpic contributions upon guest uptake. It is here where chelate cooperativity, arising from the interplay of these multivalent interactions, plays a crucial role. Once the first contact is made, subsequent host–guest interactions become intramolecular, and thus devoid of nonproductive translational and rotational degrees of freedom that entropically penalize bringing two molecules together.

The magnitude of chelate cooperativity is represented by the product *K*·*EM*, where *K* is the reference intermolecular binding constant and *EM* is the effective molarity, a factor that considers the intramolecular nature of the second and subsequent association events. High *EM*s thus contribute to superior binding affinities and selectivities, attributes that are responsible for the strong self-sorting phenomena occurring in complex mixtures of diverse hosts and guests, as it happens in cells.^7^ It is generally established that *EM*s are maximized for highly rigid and geometrically complementary (i.e., preorganized) hosts and guests.^8,9^ On one hand, structural rigidity means that the two binding partners do not need to lose conformational degrees of freedom in the transition from the unbound to the bound state, which would otherwise result in a strong entropic penalty. On the other hand, any deviation from a perfect geometric fit can lead to strained assemblies, which are in this case enthalpically punished. It is however not clear what is the maximum *EM* attainable for a noncovalent intramolecular cyclization process.^10^ A recent analysis by Di Stefano and Mandolini considered an upper limit of 10^5^–10^7^ M, providing internal rotational motions are frozen and excluding solvent effects.^11^ In the real systems reported so far, *EM* values for most supramolecular complexes are in the mM range,^11,12^ although some highly preorganized cyclic assemblies stand out to provide record *EM* values of 10^3^–10^4^ M. These include Zimmerman and Duerr’s cyclotrimers,^13^ our own dinucleoside macrocycles,^14−17^ and Anderson et al.’s porphyrin wheels.^18−20^

Actually, metalloporphyrin (*MP*) assemblies so far constitute one of the most relevant and widely studied families of synthetic receptors. The interest in these compounds clearly lies in their omnipresent role in the most important biological machineries that perform diverse light-harvesting and multiredox chemical transformations in animal and plants.^21−23^ Here, *MP* derivatives are embedded in well-defined environments within protein complexes (chlorophylls, heme groups in cytochromes or hemoglobin, etc.) and enjoy accessible, compartmentalized catalytic sites around their metal centers. With the aim to mimic the extraordinary performance of these multiporphyrin assemblies, several authors have developed various covalent and supramolecular designs in which *MP*s are cofacially arranged,^24−28^ leaving a well-defined interstice between metal centers that can be employed for catalytic^29−31^ and guest-binding^32−39^ purposes. However, while considerable efforts have been made to prepare these promising face-to-face bisporphyrin cages through diverse linkages, like H-bonding or ionic interactions,^40−43^ metal–ligand coordination,^44−50^ and covalent^51−56^ or dynamic covalent bonds,^32,57−64^ fewer attention has been paid to the understanding and optimization of their actual performance as receptors. If we aim to develop efficient synthetic models of protein receptors and enzymes, we must understand the interplay of intra- and intermolecular interactions between host and guest and how they mutually influence their conformational energy landscape so as to adapt to each other to maximize binding energies and enhance substrate selectivities.

Here, we present a novel design of cofacial (Zn^II^)bisporphyrin cages based on dynamic imine bonding, which is synthetically simple, but at the same time extraordinarily robust and versatile, affording highly rigid receptors made only of *sp^2^*-hybridized C and N atoms. We show how the degree of conformational adaptability of these cages, controlled as a function of the direction of the imine bonds in their skeleton, can lead to superior binding affinities as a result of the extremely high chelate cooperativities attained (*EM* ∼ 10^3^ M), as well as to strong and unusual association selectivities, both of a host for binding different guests and also of a guest for binding diverse hosts.

## Results and Discussion

### Design, Synthesis, and Characterization of the *sp*^2^-Cages

**Figure 1:**
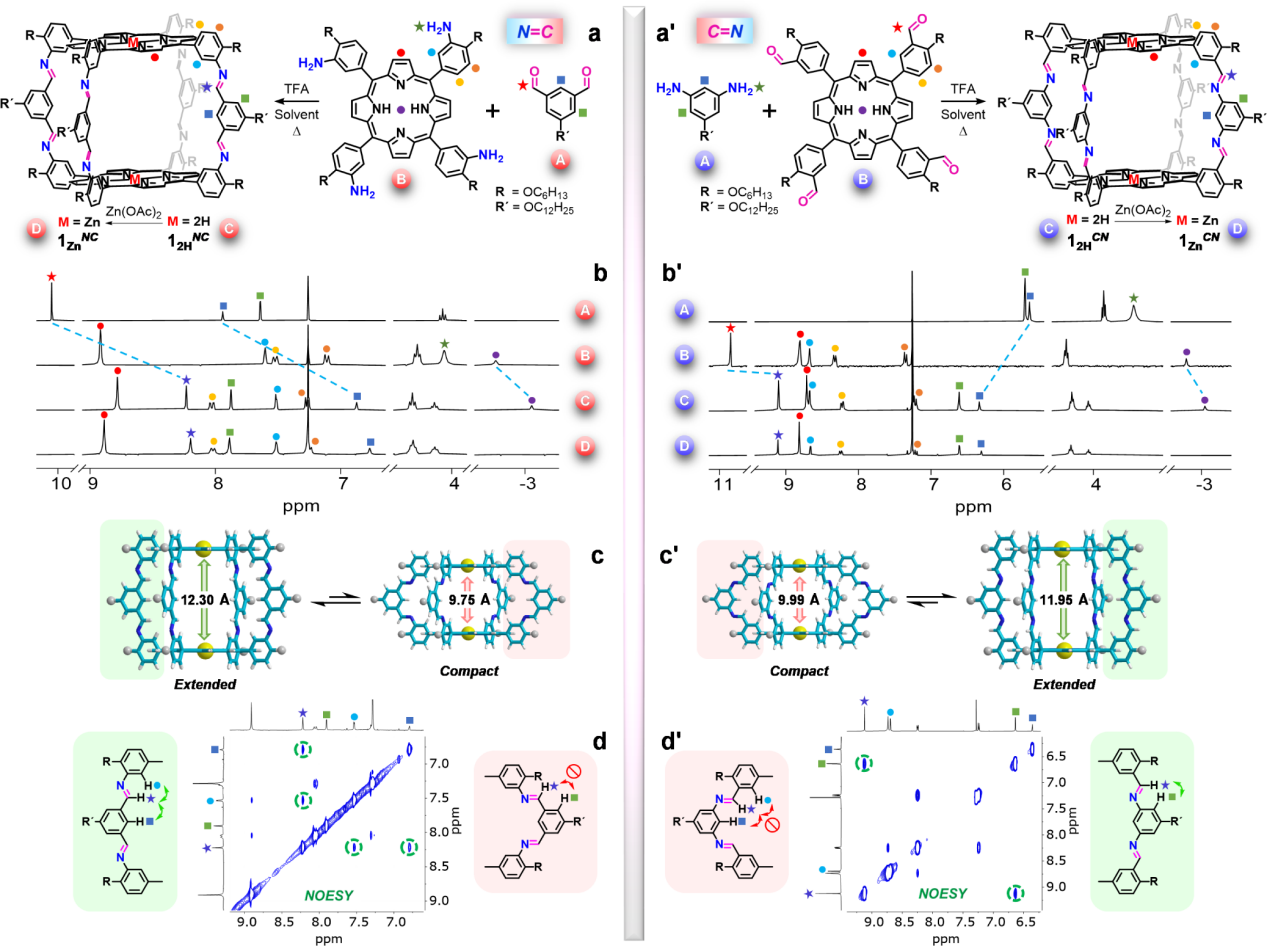
(a,a’) Synthesis of the (a) **1**_**Zn**_^*NC*^ and (a’) **1**_**Zn**_^*CN*^ bisporphyrin cages from the condensation of the corresponding amine and aldehyde precursors. (b,b’) Selected regions of the ^1^H NMR spectrum of (b) **1**_**Zn**_^*NC*^ and (b’) **1**_**Zn**_^*CN*^ in comparison with their respective free base analogues (**1**_**2H**_^*NC*^ and **1**_**2H**_^*CN*^) and their amine and aldehyde precursors. (c,c’) Models of the *extended* and *compact* conformations of (c) **1**_**Zn**_^*NC*^ and (c’) **1**_**Zn**_^*CN*^. (d,d’) Aromatic region of the NOESY spectrum of (d) **1**_**Zn**_^*NC*^ and (d’) **1**_**Zn**_^*CN*^ showing the relevant cross-peaks (green circles and arrows) that demonstrate the prevalence of the *extended* conformation in CDCl_3_ solution.

In order to produce unstrained *sp*^2^-cages having two concentric cyclic sections (Figure 1), six 120° *meta*-connections per cycle were employed, comprising *m*-substituted *meso*-arene groups at the porphyrin (*P*) and *m*-disubstituted linkers (*L*). Thus, a total of 8 dynamic covalent imine bonds are responsible for fusing the cage together. The positions of the corresponding formyl and amine precursor groups, either at the *L* or at the *P* component, can be swapped, leading to *NC* and *CN* cages, as we call them, depending on the linking direction of the imine group from the *meso*-*P* arenes to the *L* units. Hence, *NC* cages are constructed from tetraamino *P*s and dialdehyde *L*s (Figure 1a), whereas *CN* cages are instead assembled from tetraformyl *P*s and diamino *L*s (Figure 1a’). Some of the peripheral positions at the *meso-*arene groups and at the linkers were used to install long solubilizing alkoxy chains.

The synthesis of these cages is extremely simple and efficient (for experimental details and characterization data, see Supporting Information). They can be assembled by mixing their respective free-base *P* and *L* precursors in a stoichiometric 1:2 ratio (only a small excess of *L* is employed), in a variety of solvents (toluene, CHCl_3_, THF), in the presence of small amounts of trifluoroacetic acid (TFA) as a catalyst, and under mild heating. Upon completion, checked by ^1^H NMR, the reaction is quenched by addition of NEt_3_ and the product is precipitated in CH_3_OH or CH_3_CN and filtered, leading, in most cases, to considerably high yields of pure compounds. The free-base *P* cages (**1**_**2H**_^*NC*^ and **1**_**2H**_^*CN*^) can then be metalated in the presence of Zn(OAc)_2_, obtaining the final Zn(II) cage compounds **1**_**Zn**_^*NC*^ and **1**_**Zn**_^*CN*^ in close to quantitative yields.

The cages were characterized by ^1^H and ^13^C NMR, NOESY and DOSY NMR, as well as by HR-MS, UV–vis, emission and FT-IR spectroscopies (Figures S1 and S2). Figure 1b,b’ shows representative portions of their ^1^H NMR spectrum, reflecting their *D*_4h_ symmetry, compared to their corresponding *P* and *L* precursors. Imine bond formation can be characterized by an upfield NMR shift of ca. 1–2 ppm of the aldehyde proton signal and by the vanishing of the amino proton signals, as well as by a shift of the carbonyl stretching IR band from 1700 to 1622 cm^–1^. On the other hand, the incorporation of the Zn^II^ metal centers results in the disappearance of the characteristic resonance of the internal ring protons of the free base *P*s, around −3 ppm.

### Structural Analysis

Interestingly, depending on the intrinsic arrangement of the imine bonds, both the *NC* and *CN* cages may adopt two main conformations that we call “*extended*” and “*compact*”, each of them leading to distinct Zn···Zn distances between *P* planes (Figure 1c,c’). In the totally *extended* conformation all imine double bonds are arranged in the “vertical” direction, perpendicular to the *P* planes. In the fully *compact* cage conformation, the imine bonds are on the contrary arranged in the “horizontal” direction, parallel to the *P* planes. NOESY NMR was very helpful to assess which of these main conformations is preferred by the system in solution (Figures 1d,d’ and S1C–S2C). As displayed in Figure 1d for **1**_**Zn**_^*NC*^, NOE cross-peaks are observed between the imine proton and the nearby protons pointing to the interior of the cage, as expected for the *extended* conformation, but not with the external *L* protons, *ortho* to the imino group, or with the alkoxy methylene protons at the *meso*-arenes, as it would be predicted for the *compact* conformation. However, the opposite NOE correlations are observed for **1**_**Zn**_^*CN*^, as shown in Figure 1d’, which support again the prevalence of an *extended* conformation for this cage. Cross-peaks are now observed between the imine proton and the external *L* protons, but not with the protons that point to the inner space of the cage, as would be anticipated for a *compact* conformation. Thus, both **1**_**Zn**_^*NC*^ and **1**_**Zn**_^*CN*^ clearly prefer to adopt an *extended* conformation in solution, and experimental evidence for the participation of the *compact* conformation in equilibrium was not obtained, even by varying the temperature in NMR experiments in CDCl_3_ (Figure S3).

Single crystals of **1**_**Zn**_^*NC*^ suitable for X-ray diffraction were obtained by slowly cooling a saturated solution of **1**_**Zn**_^*NC*^ in DMF (Figure S1E). The molecular cage, which crystallized in the monoclinic *P*2_1_*/c* space group, presented an *extended* conformation in the crystal, which is in agreement with the NOESY results in solution. The measured Zn···Zn distance is 12.02 Å, while the imine C=N bond lengths are measured between 1.26 and 1.28 Å. Additionally, DMF molecules appear to be coordinated to the Zn atoms within the cage cavity. Interestingly, although the *P* precursor and the **1**_**Zn**_^*NC*^ cage exhibit an average *D*_4h_ symmetry, as confirmed by NMR experiments in solution, the molecular cage observed in the crystal presented a rectangular shape and thus a lower (pseudo-*D*_2h_) symmetry, presumably to achieve enhanced packing interactions (see Section S1E for a more complete description of the cage in the solid state).

**Figure 2:**
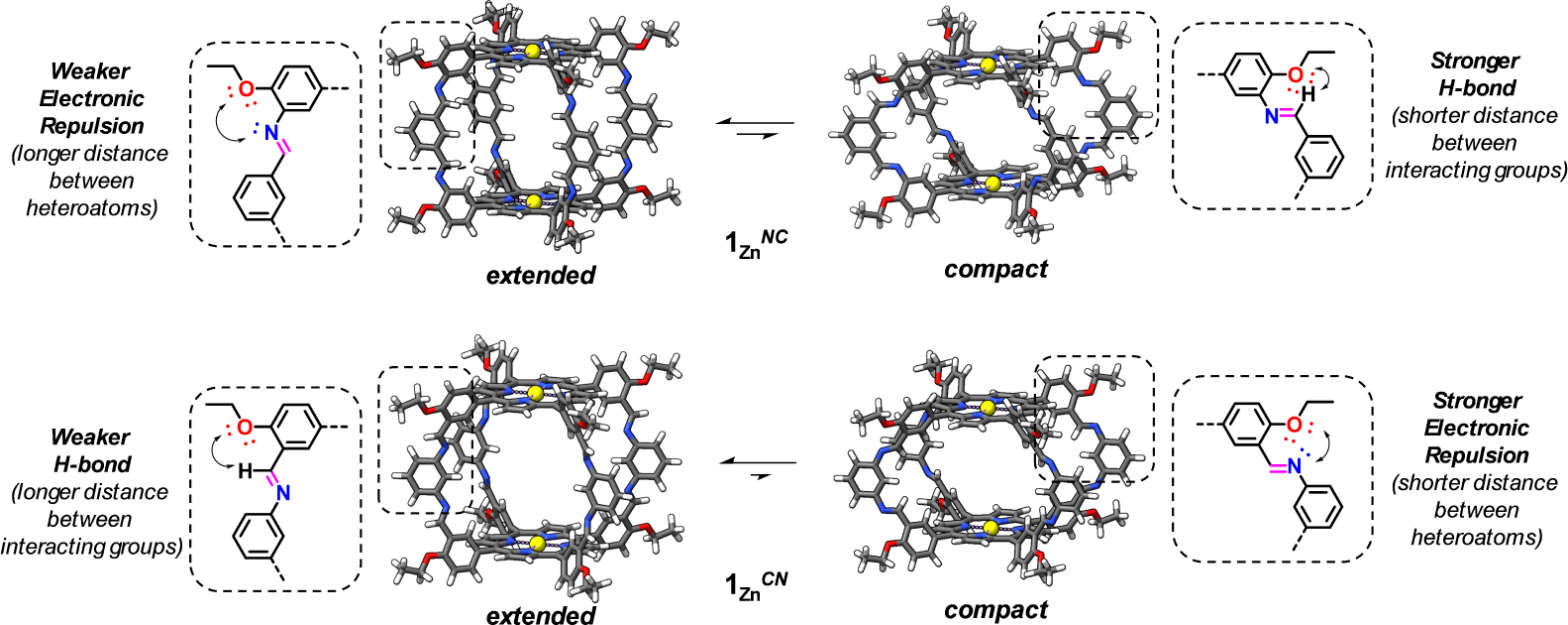
Fully optimized minimum-energy structures calculated at the DFT B3LYP/cc-PVDZ level for the models of the **1**_**Zn**_^*NC*^ (top) and **1**_**Zn**_^*CN*^ (bottom) cages in the fully *extended* (left) and *compact* (right) conformations. Depending on the direction of the imine bonds, each conformation may present repulsive electronic interactions between heteroatoms or attractive H-bonding interactions at longer (1,4) or shorter (1,5) relative positions.

To investigate in more detail the conformations of **1**_**Zn**_^*NC*^ and **1**_**Zn**_^*CN*^, density functional theory (DFT) calculations at the B3LYP/cc-PVDZ level were performed on a simplified structure of the cages, in which the −OC_6_H_13_ groups attached to the phenyl rings in *meso* position were substituted by ethoxy groups and the −OC_12_H_25_ groups of the linker were replaced by hydrogen atoms (see Section S4 for a detailed description of the calculations, as well as Figures 2 and S4A). For both **1**_**Zn**_^*NC*^ and **1**_**Zn**_^*CN*^ the *extended* conformation is predicted to be the most stable structure, in good agreement with experimental evidence in solution and in the solid state. Steric H···H interactions between the hydrogen atom attached to the imine carbon with the nearby *meso*-arene and the *m*-phenylene linker hydrogen atoms play a key role in these conformational equilibria. Indeed, the imino groups are rotated (∼44°) out of the plane of the adjacent *meso-*arene (in *extended***1**_**Zn**_^*NC*^) and of the central phenylene linker (in *compact***1**_**Zn**_^*CN*^) to avoid those interactions, an arrangement that is also observed in the crystal structures (see S.I.). This occurs for the eight imine groups and contributes to the different relative stabilities of the *extended* and *compact* conformers in **1**_**Zn**_^*NC*^ and **1**_**Zn**_^*CN*^.

However, remarkable differences are found when comparing these relative stabilities for **1**_**Zn**_^*NC*^ and **1**_**Zn**_^*CN*^. Whereas for **1**_**Zn**_^*NC*^, the *compact* conformation is 3.38 kcal mol^–1^ higher in energy than the *extended* conformation, for **1**_**Zn**_^*CN*^, the difference raises to 27.30 kcal mol^–1^, 8 times larger than that computed for **1**_**Zn**_^*NC*^. A possible reason for this difference is the proximity of the oxygen atom of the −O*R* group in *meso* arene to the atoms of the imine group (CH for **1**_**Zn**_^*NC*^ and N for **1**_**Zn**_^*CN*^), whose interaction can stabilize or destabilize, respectively, the *compact* conformation (see Figures 2 and S4B). To check this hypothesis, a new set of calculations were performed where the −O*R* group was replaced by a simple H atom and the relative energies were calculated without further optimization (Figure S4B). This comparison reveals that the energy difference between conformations becomes now larger for **1**_**Zn**_^*NC*^, up to 8.24 kcal mol^–1^, and decreases for **1**_**Zn**_^*CN*^ to 3.43 kcal mol^–1^. These energies suggest that the interaction between the imine group and the alkoxy oxygen in the *compact* conformation is decisive. In **1**_**Zn**_^*NC*^, the CH···O favorable interaction is replaced by a steric CH···H interaction, thus raising the energy, whereas for **1**_**Zn**_^*CN*^ the repulsive interaction between the N and O lone pairs, pointing to each other, is replaced by a N···H attractive interaction in the simplified model, resulting in a notable decrease in the energy difference between both conformers.

According to the DFT calculations, the upper and lower bounds of the Zn···Zn distance calculated for the *compact* and *extended* conformation of the **1**_**Zn**_^*NC*^ cage are 9.75 and 12.30 Å, respectively. The latter value is only slightly larger (by ca. 0.3 Å) than the one measured in the crystalline samples (*vide supra*). These values, respectively, change to 9.99 and 11.95 Å for the **1**_**Zn**_*^CN^* cage. In addition, each of these main *extended*/*compact* conformations have their own subset of dynamic conformations, obtained by rotation of the dihedral angle around the *meso*-bonds. A fluctuation of ±20° of the *meso*-arenes around their orthogonal disposition indeed requires a small energy, lower than 1 kcal mol^–1^ (Figure S4C), which slightly influences the Zn···Zn distance. The coordinated motion of all *meso* bonds to the same direction, which also involves a rotation of one *P* plane with respect to the other, would indeed result in an additional compression of the cages of about 0.5 Å.

The inner cavity volume of the **1**_**Zn**_^*NC*^ and **1**_**Zn**_^*CN*^ cages was calculated using the recently developed CageCavityCalc plugin for Python.^65^ The *extended* and *compact* conformations of **1**_**Zn**_^*NC*^ present void spaces of 827 and 650 Å^3^, whereas cage **1**_**Zn**_^*CN*^ exhibited respectively void volumes of 908 and 641 Å^3^ (see Figures S1F and S2E). Thus, the internal volume calculated for **1**_**Zn**_^*CN*^ was larger than the one obtained for **1**_**Zn**_^*NC*^ in the *extended* conformation, whereas the trend observed was the opposite in the *compact* conformer. These results can be explained as a function of the orientation of the imine protons, which point to the inner cavity in the *extended* and *compact* conformation of cages **1**_**Zn**_^*NC*^ and **1**_**Zn**_^*CN*^, respectively, thus decreasing slightly the void volume.

### Guest Binding to Rigid *sp*^2^-Receptors

**Figure 3:**
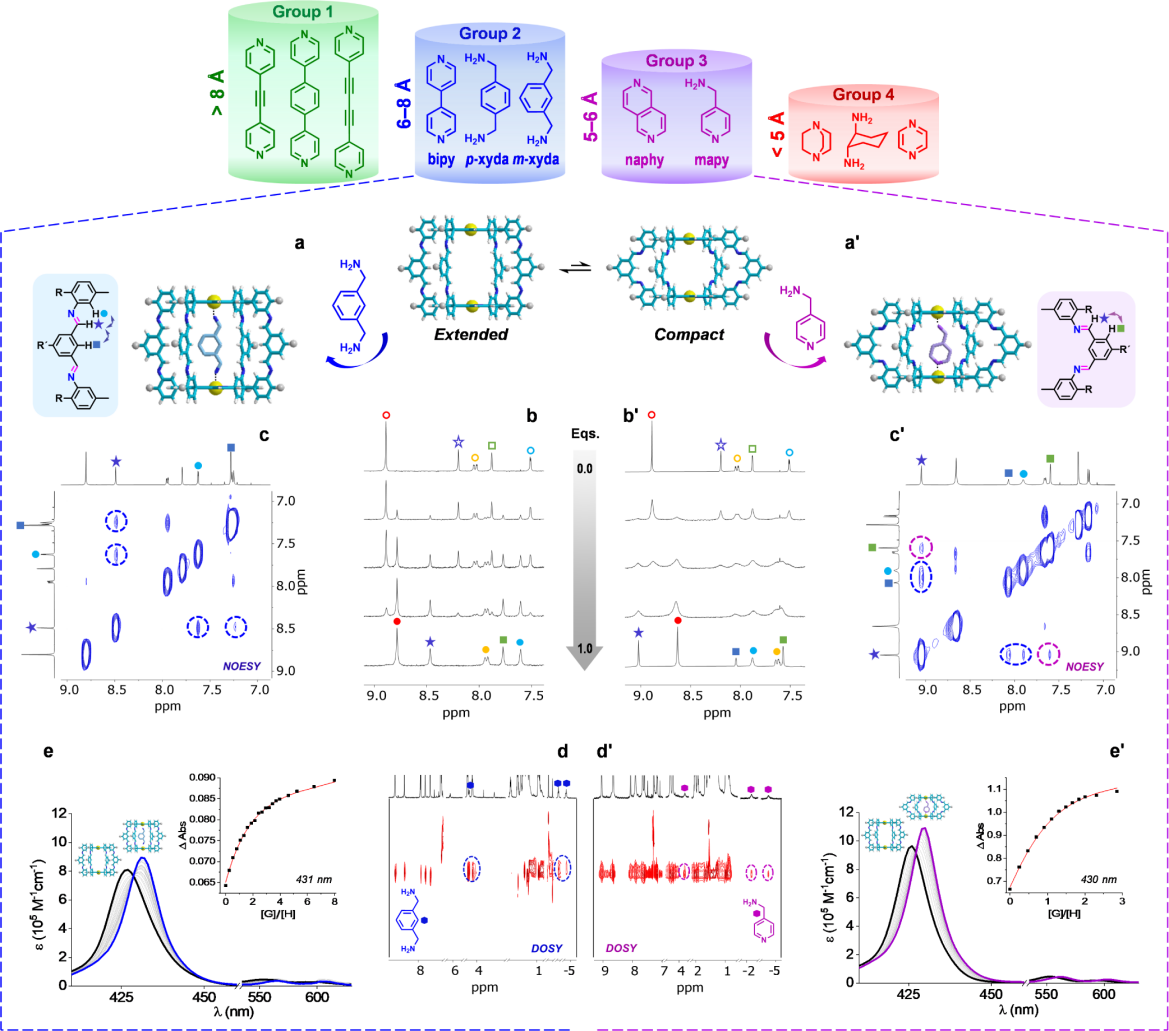
(a,a’) Guest binding of (a) *Group 2* and (a’) *Group 3* guest molecules in the *extended* or *compact* conformations of **1**_**Zn**_^*NC*^, respectively. (b,b’) Changes observed in the aromatic region of the ^1^H NMR spectrum of **1**_**Zn**_^*NC*^ as increasing amounts of (b) *m*-*xyda* or (b’) *mapy* guest molecules are added in CDCl_3_. Empty marks indicate protons of the empty cage, and filled marks protons of the guest-bound cage. (c,c’) NOESY spectra in CDCl_3_ showing ^1^H–^1^H cross-peaks compatible with (c) *extended* (blue circles and arrows) and (c’) *compact* (purple circles and arrows) conformations. (d,d’) DOSY NMR spectrum of a 1:1 mixture of **1**_**Zn**_^*NC*^ and (d) *m*-*xyda* or (d’) *mapy*, showing the guest molecules diffusing together with the cage. (e,e’) Changes observed in the absorption spectrum of **1**_**Zn**_^*NC*^ as increasing amounts of (e) *m*-*xyda* or (e’) *mapy* guest molecules are added in CHCl_3_. Insets: binding isotherms and fitting of the experimental data to a 1:1 association model.

Cages **1**_**Zn**_^*NC*^ and **1**_**Zn**_^*CN*^, having a well-defined, relatively rigid cavity and two Zn^II^ metal centers arranged in parallel planes at a precise distance, are perfectly suited to host diverse ditopic (i.e., bidentate) guest molecules endowed with two heteroatoms matching such distance. Thus, we tested multiple guest molecules having two remote nitrogen atoms, and according to the results, we arranged them in 4 groups (Figure 3).

***Groups 1* and *4***. *Groups 1* and *4* comprise those molecules that present their N atoms at either too large or too short distances, respectively, to bind to both Zn^II^ atoms within the cage cavities. Representative examples of each group are bis(4-pyridyl)acetylene and 1,4-diazabicyclo[2.2.2]octane (*DABCO*), respectively (Figure 3). Since these molecules can only bind to one Zn^II^ center, their supramolecular behavior and coordination strength is similar to that of regular monotopic Zn^II^*P*s (like **P**_**Zn**;_ see Figures S5A-F), having host:guest association constants (*K*_a_) in CHCl_3_ in the 10^3^–10^5^ M^–1^ range, and displaying bound and unbound species in fast exchange in the ^1^H NMR time scale (Figures S6A-B).

***Group 2***. The guest molecules in *Group 2*, on the other hand, possess 2 N atoms at just the right distance to bind to both Zn^II^ centers at the internal cavity of **1**_**Zn**_^*NC*^ or **1**_**Zn**_^*CN*^ in the most stable *extended* conformation. A representative example is 4,4′-bipyridine (*bipy*), with a N···N distance of about 7 Å, but other (hetero)aromatic amines, like *p*- or *m*-xylylenediamine (*p-xyda* and *m-xyda*; Figure 3), belong to this group as well. In this case, as shown in Figures 3b and S7–S11, bound and unbound hosts and guests are seen in slow NMR exchange (Figures S7A–S11A). 2D NOESY experiments (Figures 3c,S7C,S9C, and S10C) confirmed that the cage maintained an *extended* conformation upon binding *Group 2* guests, while DOSY measurements (Figures 3d,S7D, and S10D) revealed host and guest species diffusing with the same diffusion coefficient, which is an additional proof for strong binding. In fact, association constants are now too large (*K*_a_ ≫ 10^5^ M^–1^) to be determined within the NMR concentration range. This thermodynamic parameter was instead calculated in CHCl_3_ by UV–vis titrations at much lower concentrations (Figures S9B–S11B). An illustrative example of such titrations is shown in Figure 3e for *m-xyda*. Upon guest binding, a gradual red shift of the *P* Soret band is recorded, with an isosbestic point at ca. 430 nm, which can be fitted to a 1:1 model to afford *K*_a_ values around 10^7^ M^–1^ in CHCl_3_, as collected in Table 1. An extreme case is the binding of *bipy* to either **1**_**Zn**_^*NC*^ or **1**_**Zn**_^*CN*^, which is too strong (>10^8^ M^–1^) to be accurately calculated by UV–vis titrations even at a low host concentration of 10^–7^ M (Figures S7B and S8B). Unfortunately, the changes recorded by fluorescence emission spectroscopy in CHCl_3_ are not marked enough to calculate *K*_a_ by this technique at even lower concentrations. Instead, we resorted to competition experiments between slightly weaker-binding guests (*m*-*xyda*) and *bipy*, monitored by ^1^H NMR. Examples are shown in Figures S7E and S8C, from which we could indirectly derive the binding constants between *bipy* and **1**_**Zn**_^*NC*^ or **1**_**Zn**_^*CN*^ in CDCl_3_ as *K*_a_ = 1.2 × 10^8^ M^–1^ and *K*_a_ = 8.0 × 10^8^ M^–1^, respectively. We note that the use of aliphatic primary amines as guest molecules, such as *p*-*xyda* and *m*-*xyda*, does not lead to any evidence for cage destruction via imine scrambling, even if an excess of these guests is added or if the mixtures are kept in solution for prolonged times.

**Table 1:**
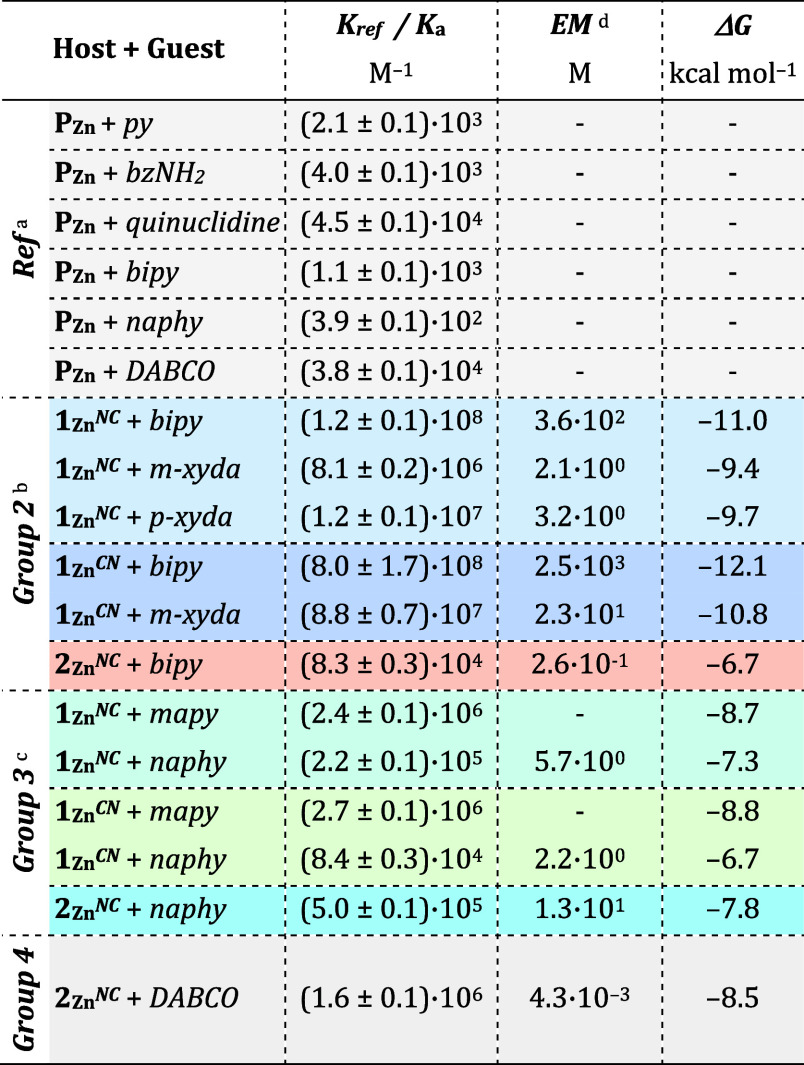
Association Constants (*K*_a_/*K*_ref_) and Effective Molarity (*EM*) Values Calculated for Diverse Bisporphyrin Host–Guest Combinations in CDCl_3_/CHCl_3_

a See Figure S5A–F.

b See Figures S7–S11.

c See Figures S14–S17.

d Determined as *EM* = 4*K*_a_/*K*_ref_^2^ (see Figure S12).

These binding affinities are exceptionally large, clearly outscoring previous bisporphyrin receptors reported in the literature, whose association constants with *bipy* typically lie in between 10^5^–10^7^ M^–1^.^66−70^ From these *K*_a_ values and the reference equilibrium constants (*K*_ref_) calculated for the interaction between the model **P**_**Zn**_ porphyrin macrocycle and suitable nitrogenated guests (see Table 1 and Figures S5A–F), we estimated the statistically corrected effective molarity (*EM*) values associated with the second binding event within the cage cavity as *EM* = 4·*K*_a_/*K*_ref_^2^ (for more details, see Figures S12A,B).^11^ As *K*_ref_, we decided to preferably employ half the value of the association constants calculated for the 1:1 interaction between ditopic guests (i.e., 4,4′-bipyridine, 2,6-naphthyridine or *DABCO*; see Figures S5D–F) and **P**_**Zn**_, since they take into account intramolecular electronic effects that are absent in the corresponding monotopic guests, like pyridine (*py*).^71^ The only exception is the binding of *p*-*xyda* and *m*-*xyda* to the cages, for which the association constant between **P**_**Zn**_ and benzylamine (*bzNH_2_*; see Figure S5B) was considered as the reference *K*_ref_. As shown in Table 1, the *EM*s exhibited by these *Group 2* guests, sometimes reaching over 10^3^ M, are remarkably large, and justify the high *K*_a_ values originated.

In order to corroborate these *EM* values through a different approach, we conducted competition experiments between ditopic *bipy* and monotopic *py* guests, whereby increasing amounts of pyridine-*D*_5_ are added to dissociate the **1**_**Zn**_^*NC*^/**1**_**Zn**_^*CN*^ cage·*bipy* complexes. In these experiments, we are essentially making the intermolecular and intramolecular versions of the same interaction to compete, and the equilibrium constant of this competition (*K*_C_) can be directly related to *EM* as *K*_C_ = 4·*EM* (for details see Figures S13A,C). As can be appreciated in Figures S13B,D, the **1**_**Zn**_^*NC*^/**1**_**Zn**_^*CN*^·*bipy* complexes are virtually unaffected by the addition of a few equivalents of pyridine-*D*_5_. The addition of a larger excess of this monotopic guest, however, progressively results in the dissociation of the 1:1 complex. For instance, ca. 50 equiv of pyridine-*D*_5_ are required to expel 10% of the ditopic *bipy* guest inside **1**_**Zn**_^*CN*^. These experiments provided *EM* values in CDCl_3_ that follow the same trend and are only slightly lower than those shown in Table 1, reaching *EM* = 65 M for the **1**_**Zn**_^*NC*^·*bipy* complex and *EM* = 670 M for the **1**_**Zn**_^*CN*^·*bipy* complex.

The rationale behind these superior *K*_a_ and *EM* values clearly lies in the preorganization of the **1**_**Zn**_^*NC*^ and **1**_**Zn**_^*CN*^ cages for the cooperative binding of *Group 2* guests. On one hand, the cages are considerably rigid in comparison to most previously reported bisporphyrin cages. With the exception of the peripheral solubilizing groups, **1**_**Zn**_^*NC*^ and **1**_**Zn**_^*CN*^ exclusively comprise C *sp*^2^- and N *sp*^2^-hybridized atoms and display π-conjugation along the *x*, *y* (porphyrin planes) and *z* (bis-imine linkers) directions. In addition, due to the 4-fold connection, these receptors are devoid of any internal rotors. Furthermore, the N···N distance of the guest molecules in *Group 2*, which are also relatively rigid, are an excellent match to the Zn···Zn separation in the **1**_**Zn**_^*NC*^/**1**_**Zn**_^*CN*^*extended*, most stable conformation (see NOESY spectrum in Figure 3c). Therefore, it is not surprising that the largest *EM* (ca. 2500 M), which is among the highest values reported in the literature for supramolecular systems,^13−20^ corresponds to the binding of the most rigid guest (*bipy*) to the most rigid receptor (**1**_**Zn**_^*CN*^). Still, the fact that the cages can efficiently host molecules of slightly different N···N distances, such as *p*-*xyda* and *m*-*xyda*, means that they can dynamically adapt to some extent their conformation, and hence the Zn···Zn distance, as discussed above, by rotation of the dihedral angle around the *meso*-bonds. For instance, upon insertion of different guests, calculations predict a decrease in the Zn···Zn distance in **1**_**Zn**_^*NC*^ from 12.30 (empty cage), to 11.70 (*bipy*; Figure S7H), to 11.66 (*p*-*xyda*; Figure S9D), and to 11.27 Å (*m*-*xyda*; Figure S10E). However, despite the *K*_ref_ values obtained for the single binding interactions between **P**_**Zn**_ and *py*/*bipy* or *bzNH_2_* are comparable (Table 1), the *K*_a_ values obtained for the association of *p*-*xyda*/*m*-*xyda* to the **1**_**Zn**_^*NC*^/**1**_**Zn**_^*CN*^ cages are substantially lower compared to *bipy*. This weaker binding must stem from a reduced *EM* (Table 1) attributed to the higher flexibility of the −CH_2_NH_2_ groups, which introduce entropic effects associated with the loss of degrees of freedom upon binding to both metal centers that are not found in the rigid *bipy* guest.

Theoretical calculations are able to nicely reproduce these trends in association strengths. The binding energy (*E*_bind_), calculated as the energy difference between the total energies of the fully optimized structures of the supramolecular complex and the respective components, provides an estimation of the enthalpic gain upon host:guest binding. B3LYP/cc-PVDZ calculations afford *E*_bind_ values of −19.64 and −23.97 kcal mol^–1^ for *bipy* complexed by **1**_**Zn**_^*NC*^ and **1**_**Zn**_^*CN*^, respectively, a trend that is in good accord with the experiments (Table 1). Calculations predict even higher *E*_bind_ values of **1**_**Zn**_^*NC*^ with *m*-*xyda* (−23.32 kcal mol^–1^) and *p*-*xyda* (−25.43 kcal mol^–1^), that reproduce well the increase in *K*_a_ in going from **1**_**Zn**_^*NC*^·*m*-*xyda* to **1**_**Zn**_^*NC*^·*p*-*xyda*, but do not justify their lower *K*_a_ values compared with **1**_**Zn**_^*NC*^·*bipy*. This result, however, indirectly confirms that the weaker binding of these benzylic diamines is caused by entropic differences between bound and free guests, which are not taken into account in these theoretical calculations.

Therefore, binding of a guest to both metal centers brings about a substantial thermodynamic stabilization of the supramolecular complexes. Moreover, such double coordination also results in significant kinetic stabilization. As noted above, the host–guest exchange becomes slow in the NMR time scale, which prompted us to perform 2D EXSY experiments of 1:0.5 mixtures of **1**_**Zn**_^*NC*^/**1**_**Zn**_^*CN*^ and *bipy* in CDCl_3_ (see Figures S7F and S8D). Data analysis revealed exchange rates between free and bound cages in the order of *k*_ex_ = 0.238 s^–1^ for **1**_**Zn**_^*NC*^ and *k*_ex_ = 0.013 s^–1^ for **1**_**Zn**_^*CN*^. The guest release rate constant (*k*_out_) and the guest uptake rate constant (*k*_in_) can be estimated for both supramolecular complexes from the calculated exchange rates (*k*_ex_) knowing that, at equilibrium, *K*_a_ = *k*_in_/*k*_out_, and *k*_out_ can be approximated to *k*_ex_, since *k*_in_ should be dominated by diffusion.^72^ The **1**_**Zn**_^*NC*^·*bipy* complex presented values of *k*_in_ = 2.8 × 10^7^ M^–1^·s^–1^ and *k*_out_ = 2.3 × 10^–1^ s^–1^, respectively, whereas the **1**_**Zn**_^*CN*^·*bipy* complex exhibited rate constant values of *k*_in_ = 1.0 × 10^7^ M^–1^·s^–1^ and *k*_out_ = 1.3 × 10^–2^ s^–1^. The half-life of guest exchange (*t*_1/2_) can be in this case calculated as *t*_1/2_ = (ln 2)/(2*k*_out_) when half of the host is forming the host–guest complex. This leads to values of *t*_1/2_ = 1.4 s and *t*_1/2_ = 26.6 s for the **1**_**Zn**_^*NC*^·*bipy* and **1**_**Zn**_^*CN*^·*bipy* ensembles, respectively, which denotes rather slow processes in comparison to related supramolecular complexes.^24−27^ Notably, the slower exchange derived for the **1**_**Zn**_^*CN*^·*bipy* complex accompanies its higher thermodynamic stability (see Table 1).

Single crystals of supramolecular complex **1**_**Zn**_^*NC*^·*bipy* were grown by slowly cooling a saturated solution in DMF (Figure S7G). The cage complex crystallized again in the monoclinic *P2*_*1*_*/c* space group, exhibiting the lower *D*_2h_-symmetry also seen in the empty cage. Interestingly, although the cage is primarily in an *extended* conformation, a closer analysis showed that actually 6 out of the 8 imine bonds presented the arrangement of the fully *extended* conformation, while the other 2 displayed the arrangement associated with the *compact* conformation. Upon complexation with *bipy*, the Zn···Zn distance in the crystal structure decreased from 12.02 to 11.53 Å, while the Zn···N distance between the porphyrins and the dinitrogenated guest was found as 2.20 Å. These geometric parameters are in line with theoretical calculations (Figure S7H).

***Group 3.*** Now, we wondered what would occur if we provided the cage with guest molecules having N···N distances that are too short to reach both Zn^II^ centers in the *extended* conformation, but that fit nicely the Zn···Zn distances of the, so far undetected, *compact* conformation. This kind of guest belongs to *Group 3*, and representative examples are 4-(methylamino)pyridine (*mapy*) or 2,6-naphthyridine (*naphy*) (Figures 3 and S14–S17).

**Figure 4:**
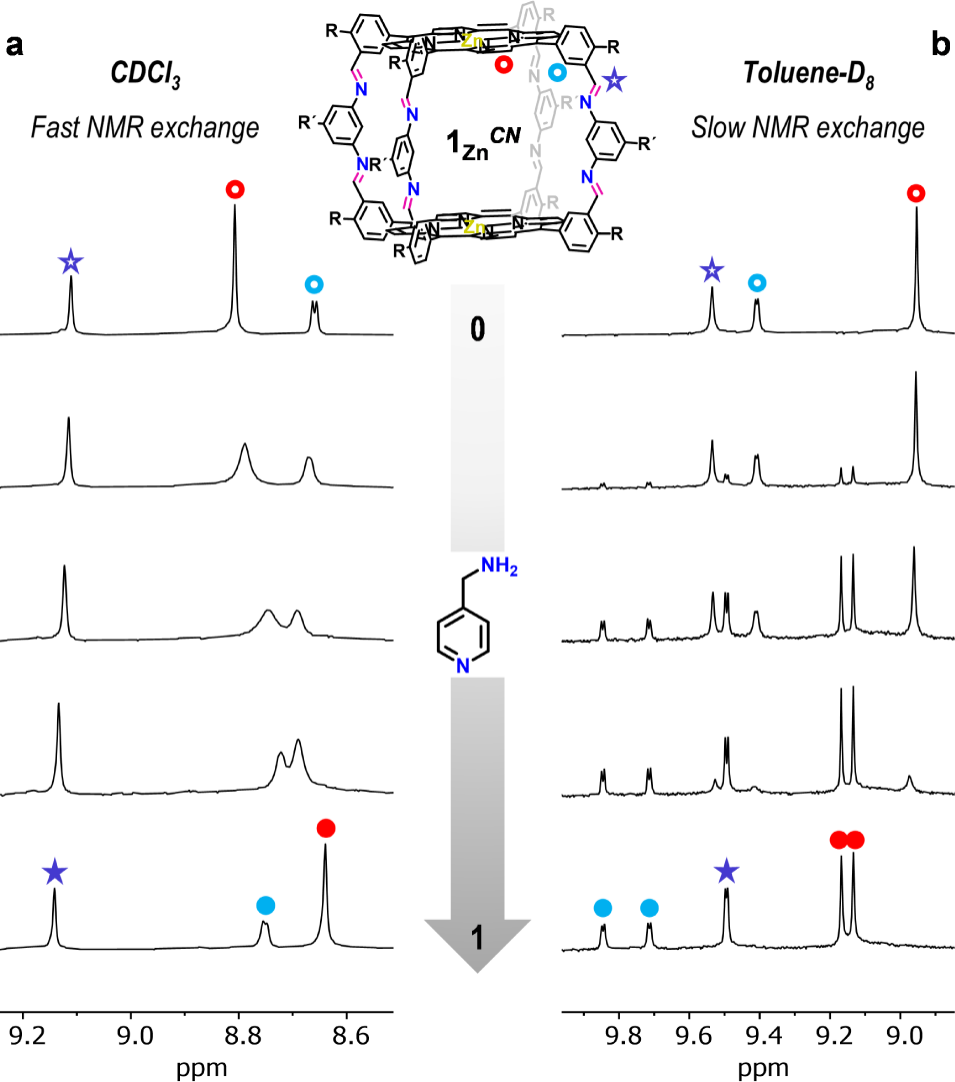
Comparison of the ^1^H NMR spectra obtained along the titration of **1**_**Zn**_^*CN*^ (1.0 × 10^–3^ M) with increasing amounts of *mapy* (up to 1 equiv) at 298 K in (a) CDCl_3_ and (b) toluene-*D*_8_. The solvent change produced both a transition from fast to slow exchange in the NMR time scale and the desymmetrization of the bisporphyrin host upon guest binding, which reveals now two sets of proton signals.

As shown in Figure 3b’, the addition of *mapy* to **1**_**Zn**_^*NC*^ leads again to a slowly exchanging mixture of bound and unbound cages until 1 equiv of guest is reached. However, a notable difference between *Group 2* and *Group 3* guests, appreciated when comparing Figures 3b and 3b’, is that at substoichiometric amounts of added guest ^1^H NMR signals are markedly broad for the latter in CDCl_3_, suggesting the existence of exchange processes that approach the NMR time scale, but sharpen again after the addition of 1 equiv of guest molecule. This effect was noted for both *mapy* and *naphy* (Figures S14A–S17A) and was attributed to a faster guest exchange between empty and occupied cages in comparison to the *Group 2* systems. Unexpectedly, the exchange with these *Group 3* guests was even faster for the **1**_**Zn**_^*CN*^ cage, actually reaching the fast NMR exchange regime, as shown in Figures 4a, S15A and S17A. Such exchange could be slowed down by decreasing the temperature (Figure S14E) or in less polar solvents like toluene (Figures 4b and S14A–S17A), which transformed the ill-defined, broad ^1^H NMR features observed at substoichiometric amounts of guest into sharp sets of signals for the empty and occupied **1**_**Zn**_^*NC*^/**1**_**Zn**_^*CN*^ hosts in equilibrium. Interestingly, the solvent change from CDCl_3_ to toluene-*D*_8_ also revealed the desymmetrization of the cage upon binding of nonsymmetric guest molecules, like *mapy*, in the cavity (Figure 4b; see also Figures S14A,E and S15A). Thus, the 1:1 mixture of *mapy* and **1**_**Zn**_^*NC*^ or **1**_**Zn**_^*CN*^ revealed two sets of ^1^H NMR signals corresponding to the two halves of the cage: one of them bound to the pyridine and the other to the amine fragments of *mapy*.

Another marked difference between *Group 2* and *Group 3* guests, and also between the **1**_**Zn**_^*NC*^ and **1**_**Zn**_^*CN*^ cages, are the ^1^H NMR spectroscopic changes experienced by the protons around the imine group upon guest binding. When the **1**_**Zn**_^*NC*^ cage binds *mapy* or *naphy*, the imine proton signal suffers a large downfield shift from ca. 8.2 to 9.1 ppm (see signal marked as a blue star in Figure 3b’), whereas NOESY experiments reveal as well clear cross-peaks between the imine proton and the protons outside the **1**_**Zn**_^*NC*^ cage cavity (marked in purple in Figure 3c’; see also Figures S14C and S16C). These results altogether afford a clear indication that the **1**_**Zn**_^*NC*^ cage suffers an imine rearrangement into a *compact* conformation, so as to structurally adapt the Zn···Zn distance to these *Group 3* molecules. Surprisingly, when the **1**_**Zn**_^*CN*^ cage binds *mapy* or *naphy*, the imine proton experiences negligible chemical shifts, while the NOESY cross-peaks attributed to *compact* arrangements are much less intense relative to those that support an *extended* conformation (see Figures S15C and S17C). An explanation for these differences between **1**_**Zn**_^*NC*^ and **1**_**Zn**_^*CN*^ based on computational studies will be given below.

Binding constants between **1**_**Zn**_^*NC*^/**1**_**Zn**_^*CN*^ and *Group 3* guests were determined by UV–vis titrations and are displayed in Table 1. These *K*_a_ values are still exceptionally high, around 10^5^–10^6^ M^–1^, certainly larger than single-point binding interactions, which implies that the guests are binding to both metal centers in the cavity. However, they are substantially smaller than those obtained for *Group 2* guests, which is a manifestation of the energetic cost associated with the change to less stable cage conformations with shorter Zn···Zn distances (*vide infra*). *EM* values are, accordingly, smaller by 2–3 orders of magnitude with respect to *Group 2* guests. On the other hand, DOSY experiments (Figures 3d’ and S14D) revealed again the cage diffusing together with the included guest, which displayed strongly upfield shifted ^1^H NMR signals (see also Figures S14A–S17A) due to the aromatic ring currents originated between the two *P*s.

### Conformational Energy Landscape

The energetic cost associated with the conformational transformation required to accommodate *Group 3* guests, presumably through an *extended-*to*-compact* cage rearrangement (Δ*G*^0^_e→c_), can now be roughly estimated experimentally from the difference between the free energy of association of *bipy* and *naphy* to **1**_**Zn**_^*NC*^/**1**_**Zn**_^*CN*^ (see Figure S18) as: 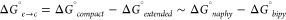


In this approximation, we must assume that the intrinsic contribution of each guest to the binding interaction is the same, which is not entirely correct due to electronic and structural differences (i.e., *naphy*’s N atoms deviate slightly from the linear alignment imposed to optimize binding to the two Zn atoms). However, these differences between the two guests are the same for the two cages, so the mutual comparison may be valid. Interestingly, we found out that the energy cost for this conformational change in CHCl_3_ is appreciably smaller for **1**_**Zn**_^*NC*^ (Δ*G*^0^_e→c_ = 3.73 kcal·mol^–1^) than that for **1**_**Zn**_^*CN*^ (Δ*G*^0^_e→c_ = 5.41 kcal·mol^–1^). In other words, cage **1**_**Zn**_^*CN*^ is indeed even more rigid than cage **1**_**Zn**_^*NC*^ and requires more energy to undergo the conformational change. Looking back to Table 1, this higher structural rigidity explains why the *EM*s exhibited by **1**_**Zn**_^*CN*^ are larger for *Group 2* guests, but smaller for *Group 3* guests, in comparison to **1**_**Zn**_^*NC*^.

These energy differences between conformations determined experimentally are in excellent agreement with those calculated by DFT for **1**_**Zn**_^*NC*^ (3.73 vs 3.38 kcal·mol^–1^), but are about 5 times lower for **1**_**Zn**_^*CN*^ (5.41 vs 27.30 kcal·mol^–1^). However, one must take into account that in the theoretical calculations all imine bonds are transformed from the *extended* to the *compact* arrangement, while we got enough experimental evidence in solution (the NOESY spectrum of the **1**_**Zn**_^*NC*^·*mapy* complex shown in Figure 3c’) and in the solid state (the crystal structure of the **1**_**Zn**_^*NC*^·*bipy* complex shown in Figure S7G) that such *extended*-to-*compact* transformations might not be complete for all imine groups and the cages can adopt intermediate conformations. This last issue might impact to a much higher extent the more rigid **1**_**Zn**_^*CN*^ cage and can be the origin of the differences observed between theory and experiment. Therefore, in order to shed more light on these conformational adaptations as a function of the guest added, we performed a set of calculations in which the 8 imine groups are twisted one-by-one in going from the fully *extended* to the totally *compact* conformation (see Figures S19A–G). By means of these calculations, we were interested to know: (1) the relative energy between conformations, (2) the energy barriers ruling their interconversion, and (3) the changes in Zn···Zn distance for both the empty cages, and the cages were *bipy* or *naphy* were complexed.

When *bipy* is hosted inside **1**_**Zn**_^*NC*^ and **1**_**Zn**_^*CN*^, the cages prefer to stay in the *extended* conformation because the transformation to an elongated *compact* conformation (Zn···Zn distance of 11.75 Å) implies an energy increase of ∼15 and ∼42 kcal·mol^–1^ for **1**_**Zn**_^*NC*^ and **1**_**Zn**_^*CN*^, respectively (Figures S19B,F).

In contrast, when *naphy* (or *mapy*) was included as a guest in **1**_**Zn**_^*NC*^ (Figure S19D), the twisting of the imine groups into a *compact* arrangement results in a more stable complex, a minimum energy being achieved when 4/5 imines are rotated, resulting in a **1**_**Zn**_^*NC*^·*naphy* conformer that is 8.5 kcal·mol^–1^ more stable than the totally *extended* one. Further compaction of the cage by rotating all of the imines slightly destabilizes the complex up to 1.65 kcal·mol^–1^. This suggests that the size of *naphy* (N···N distance of 5.10 Å) is actually optimal for an intermediate conformation in between the *extended* and *compact* cages, which is in perfect accordance with the NOESY measurements for the **1**_**Zn**_^*NC*^·*naphy* and the **1**_**Zn**_^*NC*^·*mapy* complexes (Figures S16C and 3c’), where cross-peaks compatible with coexisting *extended* (blue circles) and *compact* (purple circles) imine arrangements are detected.

In contrast to **1**_**Zn**_^*NC*^, the complexation of *naphy* by the **1**_**Zn**_^*CN*^ cage shows completely different behavior. Due to the considerably higher energy required to rotate the imines from the *extended* to the *compact* form (27.30 kcal·mol^–1^), **1**_**Zn**_^*CN*^ prefers to incorporate the *naphy* guest undergoing a significant compression of the cage (Zn···Zn distance of 9.87 Å), but preserving the *extended* imine arrangements (Figures S19E,F). Considering the binding energy of this compressed **1**_**Zn**_^*CN*^·*naphy* complex (15.39 kcal·mol^–1^) and that of the **1**_**Zn**_^*CN*^·*bipy* complex (23.97 kcal·mol^–1^), the difference in energy of the **1**_**Zn**_^*CN*^ cage in binding *bipy* and *naphy* amounts to 8.58 kcal·mol^–1^, which is in better accordance with the experimental value of 5.41 kcal·mol^–1^.

Theoretical calculations therefore indicate that the **1**_**Zn**_^*NC*^·*naphy* ensemble prefers to adopt conformations in which several, but not all, imine groups are disposed in a *compact* arrangement, whereas the **1**_**Zn**_^*CN*^·*naphy* complex predominantly exists with the cage in an *extended*, but compressed conformation. Experimentally, this difference is supported on one hand by the smaller association constants and faster exchange rates shown by **1**_**Zn**_^*CN*^, and, on the other, by the absence of chemical shifts for the imine proton and NOESY cross-peaks that are mostly compatible with imines in *extended* arrangements in the case of the **1**_**Zn**_^*CN*^·*naphy* complex, as described above.

### Guest Binding to Flexible Receptors

**Figure 5:**
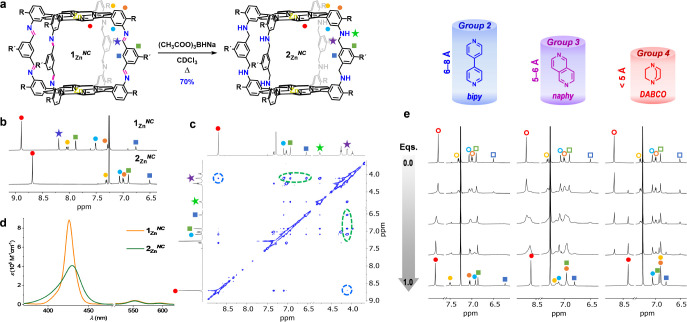
(a) Imine reduction reaction of the rigid *sp*^2^-cage **1**_**Zn**_^*NC*^ into the flexible cage **2**_**Zn**_^*NC*^. (b) Aromatic region of the ^1^H NMR spectra of **1**_**Zn**_^*NC*^ and **2**_**Zn**_^*NC*^ in CDCl_3_. (c) 2D NOESY NMR spectrum of **2**_**Zn**_^*NC*^ in CDCl_3_, showing several relevant cross-peaks between the methylene protons and the linker (in green) or pyrrole (in blue) protons. (d) Comparison of the UV–vis absorption spectra of **2**_**Zn**_^*NC*^ and **1**_**Zn**_^*NC*^ in CHCl_3_. (e) Changes observed in the aromatic region of the ^1^H NMR spectra of **2**_**Zn**_^*NC*^ as increasing amounts of *bipy*, *naphy*, or *DABCO* are respectively added (up to 1.0 equiv).

In order to assess the actual contribution of rigidity to the binding affinities and selectivities attained by these *sp*^2^-cages, the imine C=N bonds of **1**_**Zn**_^*NC*^ were reduced in the presence of NaBH(OAc)_3_, leading to **2**_**Zn**_^*NC*^ in 70% yield (Figures 5a,b and S20A). Preliminary experiments also disclosed that **1**_**Zn**_^*CN*^ can be reduced in the same way. Cage **2**_**Zn**_^*NC*^ now contains *sp*^3^ C and N atoms and, as such, must exhibit higher conformational flexibility. This was confirmed through NOESY NMR measurements, as shown in Figures 5c and S20D, which revealed now cross-peaks between both the amine and the adjacent methylene protons with all of the neighboring aromatic protons of the *meso*-arenes and the linker (marked in green in Figure 5a,c), as well as with the β-pyrrolic protons (marked in blue). On the other hand, the absorption spectrum of **2**_**Zn**_^*NC*^, in comparison to the one of **1**_**Zn**_^*NC*^ or the reference *P*, displays broad features that suggest that the two porphyrins can establish π–π interactions (Figures 5d and S20C). In agreement with this, DFT calculations predict that **2**_**Zn**_^*NC*^ can evolve from a fully *extended* structure with a Zn···Zn distance of 13.54 Å to a collapsed minimum-energy structure, in which the *P* planes are at an average distance of only 3.5 Å (Figure S21). To achieve this highly compacted structure, the linkers must adopt a folded conformation, and solvent molecules should be expelled from inside the cage. This justifies the higher upfield δ-shift observed for the β-pyrrolic protons of **2**_**Zn**_^*NC*^ in CDCl_3_, when compared to **1**_**Zn**_^*NC*^ (Figures 5b and S20A).

As a result of this higher flexibility, **2**_**Zn**_^*NC*^ can bind strongly to guests over a wider range of N···N distances. Whereas this flexible cage is still not able to bind strongly to the longest *Group 1* guests, because the maximum Zn···Zn distance is still limited by the four linkers, it can now accommodate the shortest *Group 4* guests into the cavity so that they can bind to both metal centers. For instance, the addition of *DABCO* now shows bound and unbound receptors as sharp signals in slow NMR exchange (Figures 5e and S22A). NOESY NMR (Figure S22C) revealed the same cross-peaks between the methylene protons and the 3 closest aromatic protons, while the *DABCO* protons at −4.9 ppm displayed NOE cross-peaks with the β-pyrrolic protons and the internal aromatic protons at the *meso*-arene groups, which confirms the inclusion of *DABCO* (Figure S22D).

On the contrary, the binding affinity of **2**_**Zn**_^*NC*^ for *Group 2* guests is severely reduced with respect to **1**_**Zn**_^*NC*^. Binding of *bipy*, for instance, is still detected in slow NMR exchange in CDCl_3_ (Figures 5e and S23A), but the **2**_**Zn**_^*NC*^ proton signals at substoichiometric amounts of guest are broad, which indicates a much faster exchange than with **1**_**Zn**_^*NC*^. Association constants between **2**_**Zn**_^*NC*^ and *bipy* were calculated by absorption titration experiments (Figure S23B) as *K*_a_ = 8.3 × 10^4^ M^–1^ in CHCl_3_ (Table 1). These binding affinities are comparable to other flexible bis-porphyrin receptors published in the literature,^66−68^ and entail an *EM* below 10^–1^ M, which is about 3 orders of magnitude lower than the one exhibited by the rigid **1**_**Zn**_^*NC*^ cage. Turning our attention to the binding between **2**_**Zn**_^*NC*^ and *Group 3* guests, like *naphy*, ^1^H NMR titration measurements (Figures 5e and S24A) infer similar host–guest exchange kinetics as with *bipy*, in view of the broad NMR signals in slow exchange at the NMR time scale obtained in both cases (please also compare Figures S16A and S24A). The binding affinities of the flexible **2**_**Zn**_^*NC*^ and the rigid **1**_**Zn**_^*NC*^ cages for the *naphy* guest are now very similar, with *K*_a_ = 5.0 × 10^5^ and 2.2 × 10^5^ M^–1^, respectively. Interestingly, the *naphy* guest binds now more strongly to **2**_**Zn**_^*NC*^ than *bipy*, and discloses an *EM* that is about 1 order of magnitude larger (Table 1). One of the reasons for this difference might be the larger entropic cost associated with stretching the flexible linker upon inclusion of longer guests, which would reduce the number of conformational degrees of freedom in the bound versus the unbound states. Another reason is that now this flexible **2**_**Zn**_^*NC*^ cage can readapt the relative orientation of the *P* planes to align better the Zn atoms to the N atoms in the *naphy* guest, as shown in Figure S24D, something that was impeded in the rigid **1**_**Zn**_^*NC*^*sp*^2^-cage. DFT calculations predict that the Zn···Zn distance decreases from 13.54 Å in empty **2**_**Zn**_^*NC*^, to 11.98 Å in **2**_**Zn**_^*NC*^·*bipy*, to 9.67 Å in **2**_**Zn**_^*NC*^·*naphy*, and to 7.37 Å in **2**_**Zn**_^*NC*^·*DABCO*, thus supporting the high ability of the **2**_**Zn**_^*NC*^ cage to adjust its cavity to host ditopic guest molecules of different size.

### Binding Selectivity

Although the thermodynamic data acquired along this work and compiled in Table 1 already speak about highly selective association processes when several guests are offered to a particular host or even when several hosts are mixed with a given guest, we designed a set of experiments that could corroborate such binding selectivities.

#### Selective Association of a Specific Guest—Hierarchical Binding Selectivity

**Figure 6:**
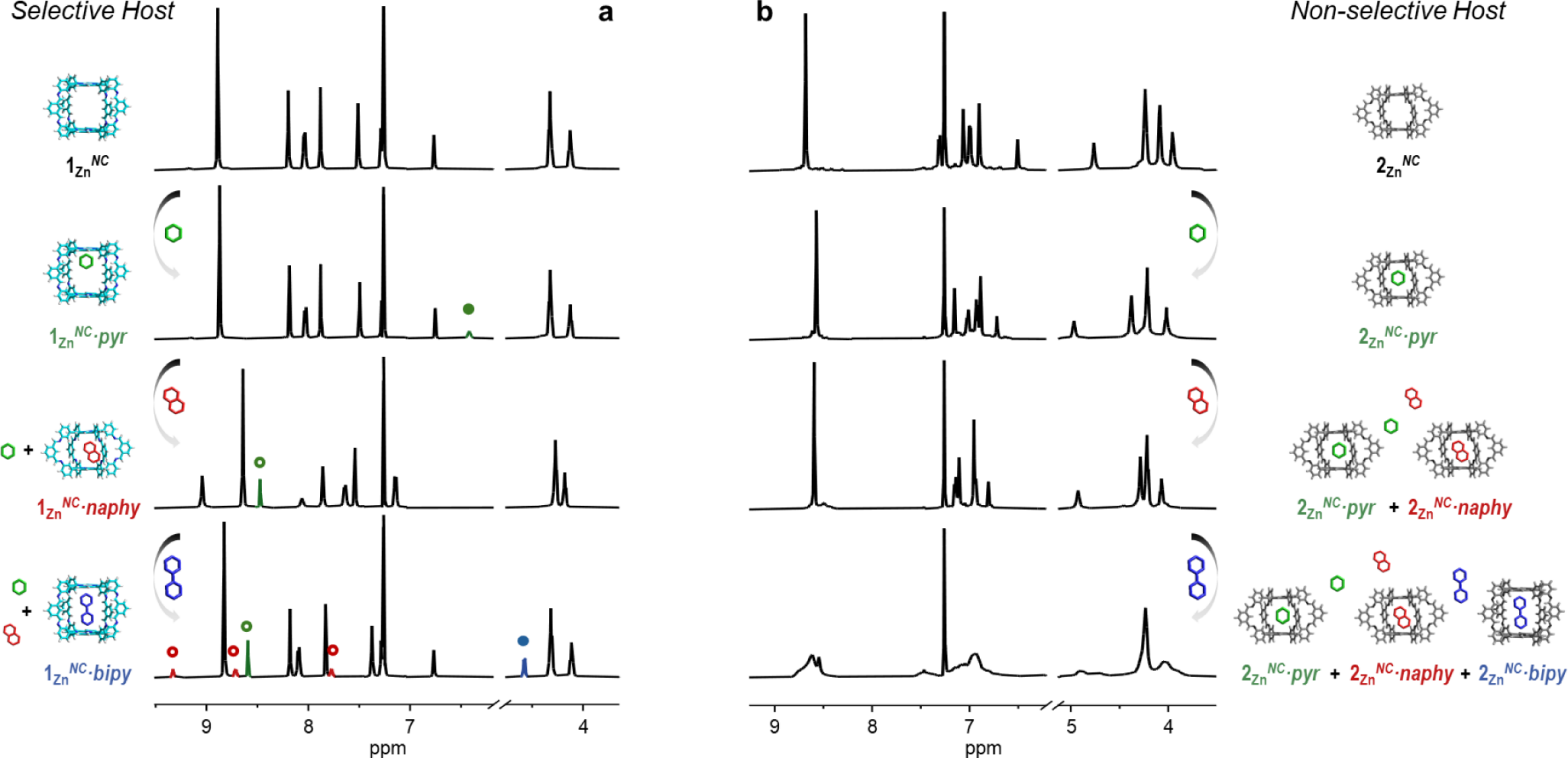
Selective association of a specific guest. Changes observed in selected regions of the ^1^H NMR spectrum of (a) **1**_**Zn**_^*NC*^ or (b) **2**_**Zn**_^*NC*^ (1 mM in CDCl_3_) upon sequential addition of (from top to bottom) 1 equiv of *pyr*, *naphy*, and *bipy*. Filled and empty circles denote bound and unbound guests, respectively.

First, using **1**_**Zn**_^*NC*^ as a model of a rigid host, we made titration experiments in which 1 equiv of ditopic guests with different N···N distances are sequentially added. As shown in Figure 6a, the addition of 1 equiv of pyrazine (*pyr*; *Group 4*) to **1**_**Zn**_^*NC*^ results in the binding of this guest to the Zn^II^*P* metal centers. The host ^1^H NMR signals do not change much in shape or position, but the *pyr* proton signals shift upfield from 8.5 to 6.4 ppm upon binding (green filled circle in Figure 6a). The addition of 1 equiv of *naphy* (*Group 3*) to this 1:1 mixture results then in nearly quantitative formation of the **1**_**Zn**_^*NC*^·*naphy* complex (please, compare with Figure S16A), and the unbound *pyr* molecules are found again at 8.5 ppm (green empty circle). Subsequently, the addition of 1 equiv of *bipy* (*Group 2*) to this 1:1:1 **1**_**Zn**_^*NC*^/*pyr*/*naphy* mixture leads to virtually complete formation of the **1**_**Zn**_^*NC*^·*bipy* ensemble (please, compare with Figures 3b and S7A). The proton signals of the bound *bipy* guest can be seen at 5.1 ppm (blue filled circle in Figure 6a), whereas the signals of the expelled *naphy* are now detected in the 7.5–9.5 ppm region (red empty circles).

Therefore, binding selectivity takes place here along two levels of hierarchy as a function of the guest N···N distances: a bottom one that discriminates between *Group 4* (<5 Å) or *Group 1* (>8 Å) guests and *Group 3* (5.0–6.0 Å) guests, through double Zn^II^:N coordination in a strained *compact* receptor conformation, and a top one that selects *Group 2* (6.0–7.5 Å) guests among the rest, by cooperative binding in a relaxed *extended* conformation. However, as demonstrated in Figures 6b and S25 where the same addition protocol was applied to **2**_**Zn**_^*NC*^, such remarkable selectivity is lost when this flexible host was employed, and the combination of the three guests produced, in this case, a complex mixture displaying broad, ill-defined signals.

#### Selective Association to a Specific Host—Self-Sorting Driven by Conformational Adaptability

As demonstrated before, while **1**_**Zn**_^*NC*^ and **1**_**Zn**_^*CN*^ cages are nearly isostructural and differ only in the direction of the imine bonds, this has an important effect on the rigidity of the cage. This panorama invited us to propose an extreme case of self-sorting: What would be the supramolecular complexes formed in a 1:1:1:1 quaternary mixture of **1**_**Zn**_^*NC*^, **1**_**Zn**_^*CN*^, *bipy*, and *naphy*? Both cages display a strong preference for the *extended* conformation and thus similar Zn···Zn distance, but this preference is even higher for **1**_**Zn**_^*CN*^, so maybe **1**_**Zn**_^*NC*^ is the receptor that makes the sacrifice to undergo a conformational change to bind *naphy*, resulting in a higher abundance of the **1**_**Zn**_^*CN*^·*bipy* and **1**_**Zn**_^*NC*^·*naphy* complexes.

**Figure 7:**
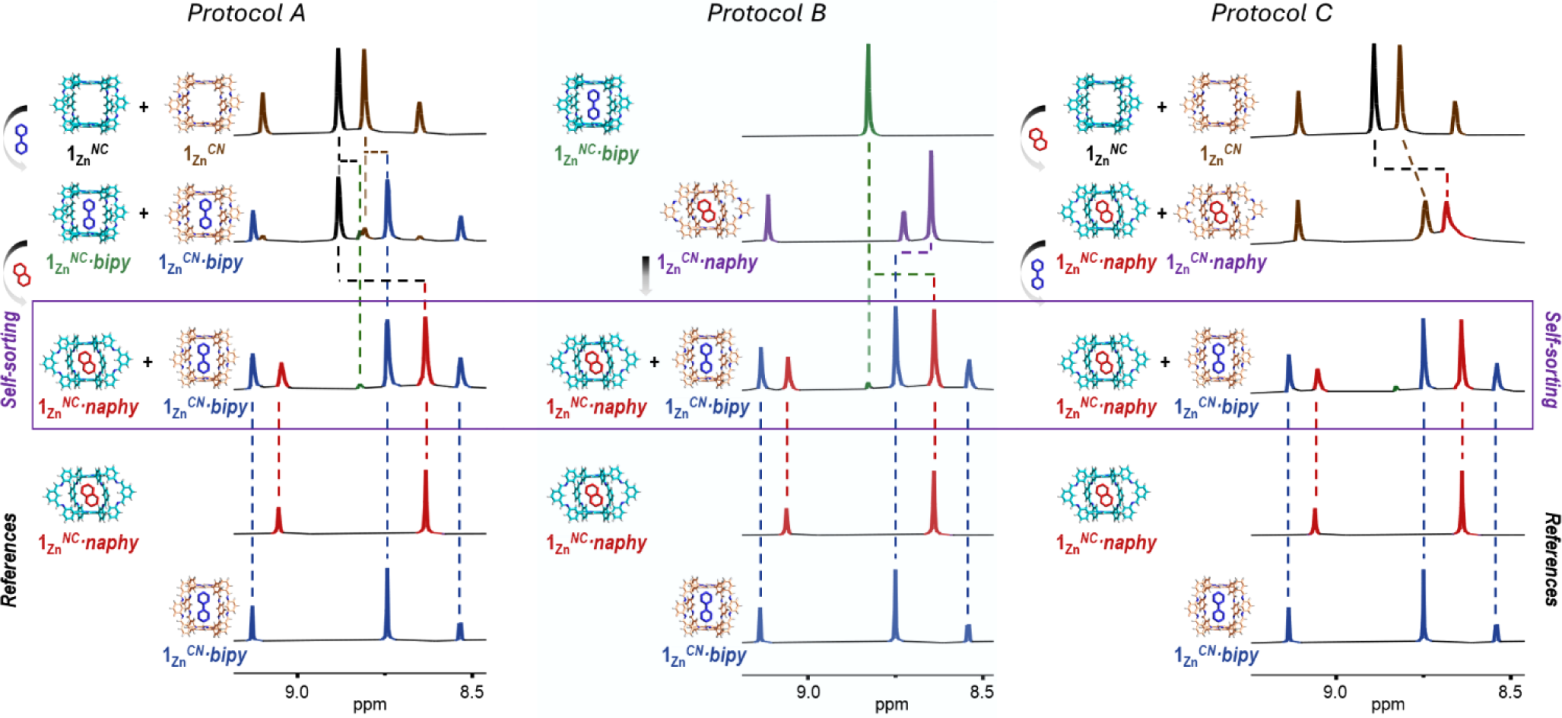
Selective association to a specific host. Self-sorting experiments monitored by ^1^H NMR and performed by sequential addition of 1 eq. of *bipy* and 1 eq. of *naphy* (Protocol A) or 1 eq. of *naphy* and 1 eq. of *bipy* (Protocol C) to a 1 eq. + 1 eq. mixture of **1**_**Zn**_^*NC*^ and **1**_**Zn**_^*CN*^ receptors. In Protocol B, a 1:1 mixture of the **1**_**Zn**_^*CN*^·*naphy* and **1**_**Zn**_^*NC*^·*bipy* complexes is directly combined. The same self-sorted mixture, constituted primarily by the **1**_**Zn**_^*CN*^·*bipy* and **1**_**Zn**_^*NC*^·*naphy* complexes, is achieved independently of the protocol followed, as confirmed by comparison with the spectra of the corresponding reference complexes, shown at the bottom. ^1^H NMR signal assignment to each species is shown with different colors, matching the complex names below the models. Dashed lines are meant to guide the eye to follow the evolution of the proton signals.

Thus, in the first set of experiments, *bipy* and *naphy* were sequentially added in a different order to a 1:1 mixture of **1**_**Zn**_^*CN*^ and **1**_**Zn**_^*NC*^ in CDCl_3_ (see Figures 7 and S26). Remarkably, upon addition of 1 equiv of *bipy* (Protocol A in Figure 7), this guest is primarily hosted by **1**_**Zn**_^*CN*^, as calculated by integration of the proton signals of the **1**_**Zn**_^*CN*^·*bipy* and **1**_**Zn**_^*NC*^·*bipy* complexes (ca. 90:10% ratio). On the other hand, the addition of 1 equiv of *naphy* in first place to this equimolar mixture of cages (Protocol C in Figure 7) results in the preferential uptake of this guest by the **1**_**Zn**_^*NC*^ cage, although this cannot be clearly quantified due to the faster exchange kinetics, as demonstrated before, which leads to broad signals at substoichiometric amounts of guest, and also to the chemical shifts experienced by the **1**_**Zn**_^*CN*^ proton signals (see Figures S16A and S17A). Thus, the guest can prefer to bind selectively one receptor or the other as a function of its conformational adaptability. The most rigid **1**_**Zn**_^*CN*^ affords higher *EM*s and is preferred by *bipy*, while *naphy*, on the contrary, chooses to bind **1**_**Zn**_^*NC*^ because this cage is less reluctant to change conformation. Obviously, the addition of 1 eq. of the other guest in each experiment, i.e., *naphy* in Protocol A and *bipy* in Protocol C, leads to the same equilibrium mixture, essentially constituted by the **1**_**Zn**_^*CN*^·*bipy* and **1**_**Zn**_^*NC*^·*naphy* host–guest complexes (ca. >95% of the total species), while the participation of the two other complexes, **1**_**Zn**_^*CN*^·*naphy* and **1**_**Zn**_^*NC*^·*bipy*, is virtually irrelevant. This confirms the existence of a strong and quite unique self-sorting phenomenon in this quaternary mixture, which is basically ruled by the different conformational adaptabilities of each *sp*^2^-receptor. Interestingly, the same equilibrium mixture is accessed when the **1**_**Zn**_^*CN*^·*naphy* and **1**_**Zn**_^*NC*^·*bipy* supramolecular assemblies are mixed in a 1:1 ratio (Protocol B in Figure 7). Thus, the ditopic guests are in this case exchanged between cages to arrive again to the most thermodynamically stable scenario.

## Conclusion

Much of the message in the title of this work, “highly rigid receptors afford outstanding binding affinities, chelate cooperativities, and substrate selectivities,” constitutes one of the fundamental principles established in supramolecular chemistry, and we believe this work represents a rather illustrative and appealing demonstration. Two families of nearly isostructural cofacial Zn^II^-bisporphyrin cages (**1**_**Zn**_^*NC*^ and **1**_**Zn**_^*CN*^), that only differ in the direction of the imine linkages as a result of the kind of amine and aldehyde precursors combined in their synthesis, have been the focus of study in this work. With the exception of the lateral solubilizing chains, these receptors comprise only *sp*^2^-hybridized C and N atoms and are devoid of internal rotors. This provides them with high structural rigidity, preferring to adopt an *extended* conformation in solution and the solid state. Yet, we discovered that they show some extent of conformational adaptability arising from (1) the dihedral angle at the *meso P*-arene bonds, (2) the imine bond arrangement, either parallel or perpendicular to the *P* planes, and (3) the intrinsic deformation of the whole system. Such exceptional structural features afford two levels of hierarchy in the selective binding of dinitrogen guests, the **1**_**Zn**_^*NC*^ and **1**_**Zn**_^*CN*^*sp*^*2*^-receptors being able to discriminate between guests belonging to *Groups 1–4*, with N···N distances differing in only ca. 1–2 Å. The top level includes guests having N···N distances between 6.0 and 7.5 Å (*Group 2*), like *bipy*, which can bind to both Zn^II^ centers into the cavity of **1**_**Zn**_^*NC*^ and **1**_**Zn**_^*CN*^ in their most stable *extended* conformation. They display remarkably high binding affinities (i.e., *K*_a_ up to 10^9^ M^–1^) and, as a result, full association selectivity to these hosts with respect to other dinitrogen guests. The reason behind such strong association, far superior to other related metalloporphyrin receptors, stems from an exceptionally high chelate cooperativity (i.e., *EM* ∼ 10^3^ M), which situates these complexes within the scarce but growing family of strongly cooperative synthetic supramolecular assemblies. The second level of selectivity comprises slightly smaller guests with N···N distances of about 5.0–6.0 Å (*Group 3*), such as *naphy*. Their binding demands conformational restructuring of the cage, which brings about an enthalpic penalty that is quite different for each *sp*^2^-receptor. The more flexible **1**_**Zn**_^*NC*^ cage undergoes a (partial) rearrangement of imine bonds into a *compact* conformation, while the more rigid **1**_**Zn**_^*CN*^ host is instead forced to compress slightly in an *extended* conformation so as to avoid electronic repulsion between N and O lone pairs upon imine rearrangement. Interestingly, this difference in rigidity gave rise to an orthogonal case of selectivity, not between guests this time, but between cages. A 1 + 1 + 1 + 1 quaternary mixture of *bipy*, *naphy*, and **1**_**Zn**_^*NC*^ and **1**_**Zn**_^*CN*^ almost exclusively generates the self-sorted **1**_**Zn**_^*CN*^·*bipy* and **1**_**Zn**_^*NC*^·*naphy* complexes. This is because *bipy* displayed a strong preference to bind the most rigid **1**_**Zn**_^*CN*^ cage, while *naphy* prefers to be hosted within the most flexible **1**_**Zn**_^*NC*^ receptor. Finally, a third group of guests involves molecules having N···N distances below 5.0 Å (*Group 4*) and above 8.0 Å (*Group 1*), which are unable to coordinate to both Zn^II^ centers within the cage cavity and therefore cannot compete with the others to bind strongly to these *sp*^2^-receptors. These remarkable binding affinities, chelate cooperativities, and substrate selectivities exhibited by **1**_**Zn**_^*NC*^ and **1**_**Zn**_^*CN*^ decay upon losing their structural rigidity, as demonstrated with the highly flexible **2**_**Zn**_^*NC*^ cage, obtained by reduction of the imine bonds.

Our design is synthetically simple and efficient, and at the same time extraordinarily robust and versatile, which invites us to study and apply these cage receptors and their derivatives in manifold future research directions.

## Supporting Information Available

This material is available free of charge via the Internet at http://pubs.acs.org. Experimental details, procedures, and compound characterization data (^1^H and ^13^C NMR, NOESY and DOSY NMR, as well as by HR-MS, UV–vis, emission and FT-IR spectroscopies, and X-ray diffraction), computational details, and additional host–guest supramolecular complexes information (PDF)
