# Supplementary material for: Highly Rigid, Yet Conformationally Adaptable, Bisporphyrin sp2-Cage Receptors Afford Outstanding Binding Affinities, Chelate Cooperativities, and Substrate Selectivities
Source: J Am Chem Soc. 2024 Dec 19;147(1):918–31. doi: 10.1021/jacs.4c13756 (PMC11726566; doi:10.1021/jacs.4c13756)
Supplement: Supplementary file 1 — ja4c13756_si_001.pdf [file ja4c13756_si_001.pdf]

SUPPORTING INFORMATION

Highly Rigid, yet Conformationally Adaptable, Bisporphyrin *sp*^{2}-Cage Receptors Afford Outstanding Binding Affinities, Chelate Cooperativities, and Substrate Selectivities

A. Priscila Gia,[a,‡] Alberto de Juan,[a,b,‡]* Daniel Aranda,[c] Fernando G. Guijarro,[a] Juan Aragó,[c] Enrique Ortí,[c]* Miguel García-Iglesias,[d]* and David González-Rodríguez[a,d]*

[a] A. P. Gia, Dr. A. de Juan, Dr. F. G. Guijarro, Prof. D. González-Rodríguez
Nanostructured Molecular Systems and Materials group, Organic Chemistry Department, Universidad Autónoma de Madrid, 28049 Madrid, Spain
E-mail: alberto.dejuan@uam.es, david.gonzalez.rodriguez@uam.es

[b] Dr. A. de Juan, Prof. D. González-Rodríguez
Institute for Advanced Research in Chemical Sciences (*IAdChem*), Universidad Autónoma de Madrid, 28049 Madrid, Spain

[c] Dr. D. Aranda, Dr. J. Aragó, Prof. E. Ortí
Institute of Molecular Science, Universidad de Valencia, Catedrático José Beltrán 2, 46980 Paterna (Valencia), Spain
E-mail: enrique.orti@uv.es

[d] Dr. M. Garcia-Iglesias
QUIPRE Department, Nanomedicine-IDIVAL, Universidad de Cantabria, Avd. de Los Castros, 46, 39005 Santander, Spain
E-mail: miguel.garciaiglesias@unican.es

[‡] Contributed equally to this work

SUPPORTING INFORMATION

TABLE OF CONTENTS

General Methods 3

Synthetic Procedures 4

S1. Characterization of 1_{Zn}^{NC} 44

S2. Characterization of 1_{Zn}^{CN} 49

S3. Temperature-dependent experiments of 1_{Zn}^{NC} and 1_{Zn}^{CN} 52

S4. Theoretical calculations on 1_{Zn}^{NC} and 1_{Zn}^{CN} 53

S5. Calculation of reference association constants with monotopic and ditopic compounds. 55

S6. ^{1}H NMR titration experiments with representative molecules from *Groups 1* and *4.* 60

S7. Host-Guest Chemistry. Binding of 4,4'-bipyridine (*bipy*) to 1_{Zn}^{NC} 62

S8. Host-Guest Chemistry. Binding of 4,4'-bipyridine (*bipy*) to 1_{Zn}^{CN} 72

S9. Host-Guest Chemistry. Binding of *p*-xylylenediamine (*p*-xyda) to 1_{Zn}^{NC} 77

S10. Host-Guest Chemistry. Binding of *m*-xylylenediamine (*m*-xyda) to 1_{Zn}^{NC} 80

S11. Host-Guest Chemistry. Binding of *m*-xylylenediamine (*m*-xyda) to 1_{Zn}^{CN} 83

S12. Calculation of *EM* from K_a and K_{ref} 85

S13. Calculation of *EM* from competition experiments 86

S14. Host-Guest Chemistry. Binding of 4-(methylamino)pyridine (mapy) to 1_{Zn}^{NC} 89

S15. Host-Guest Chemistry. Binding of 4-(methylamino)pyridine (mapy) to 1_{Zn}^{CN} 96

S16. Host-Guest Chemistry. Binding of 2,6-naphthyridine (*naphy*) to 1_{Zn}^{NC} 99

S17. Host-Guest Chemistry. Binding of 2,6-naphthyridine (naphy) to 1_{Zn}^{CN} 102

S18. Estimation of the energy associated to the *extended*-to-*compact* conformational rearrangement 105

S19. Theoretical Calculations. Conformational Energy Landscapes 106

S20. Characterization of 2_{Zn}^{NC} 114

S21. Theoretical calculations. Binding of guest molecules to 2_{Zn}^{NC} 117

S22. Host-Guest Chemistry. Binding of *Group 4* guests (*DABCO*) to 2_{Zn}^{NC} 118

S23. Host-Guest Chemistry. Binding of *Group 2* guests (*bipy*) to 2_{Zn}^{NC} 121

S24. Host-Guest Chemistry. Binding of *Group 3* guests (*naphy*) to 2_{Zn}^{NC} 124

S25. Non-selective performance of reduced cage 127

S26. Self-Sorting 127

References 131

General Methods

Mass Spectrometry (MS) and **High Resolution-Mass Spectrometry (HRMS)** MALDI-TOF spectra were obtained from a BRUKER ULTRAFLEX III instrument equipped with a nitrogen laser operating at 337 nm, and APCI and ESI spectra were obtained from a BRUKER MAXIS II instrument. **NMR** spectra were recorded with a *BRUKER AV-II* 300 MHz, *BRUKER AV* 500 MHz or a *BRUKER DRX* 500 MHz instruments. The temperature was actively controlled at 298 K. Chemical shifts are measured in ppm using the signals of the deuterated solvent as the internal standard [$CDCl_3$ calibrated at 7.26 (^1H) and 75.0 ppm (^{13}C), DMSO-D_6 calibrated at 2.50 (^1H) and 39.5 ppm (^{13}C), and Toluene-D_8 calibrated at 7.09 (^1H) and 137.86 ppm (^{13}C)]. **Manual column chromatography** was carried out on silica gel *Merck-60* (230-400 mesh, 60 Å), and TLC on aluminum sheets precoated with silica gel 60 F254 (Merck). **Automatic column chromatography** was carried out on CombiFlash® Rf 150-Teledyne Isco. **UV-Visible** experiments were conducted using a *JASCO V-660* apparatus. **Emission spectra** were recorded in a *JASCO FP-8600* equipment using excitation and emission bandwidths of 5-10 nm in both cases, and a 1000 ms response. In both instruments the temperature was controlled using a *JASCO* Peltier thermostatted cell holder at 298 K, adjustable temperature slope, and accuracy of ± 0.1 K. **X-ray structures** were obtained from *Rigaku XtaLAB Synergy R* diffractometer equipped with a *HyPix-Arc 100º* Curved X-ray detector irradiating with a rotator anode (Cu Kα, λ = 1.54184) at low temperature (150 K).

Starting materials

Chemicals were purchased from commercial suppliers and used without further purification. Solid hygroscopic reagents were dried in a vacuum oven before use. Pyrrole was distilled prior to be used.

Synthetic Procedures

Synthesis of porphyrins 4a and 4b.

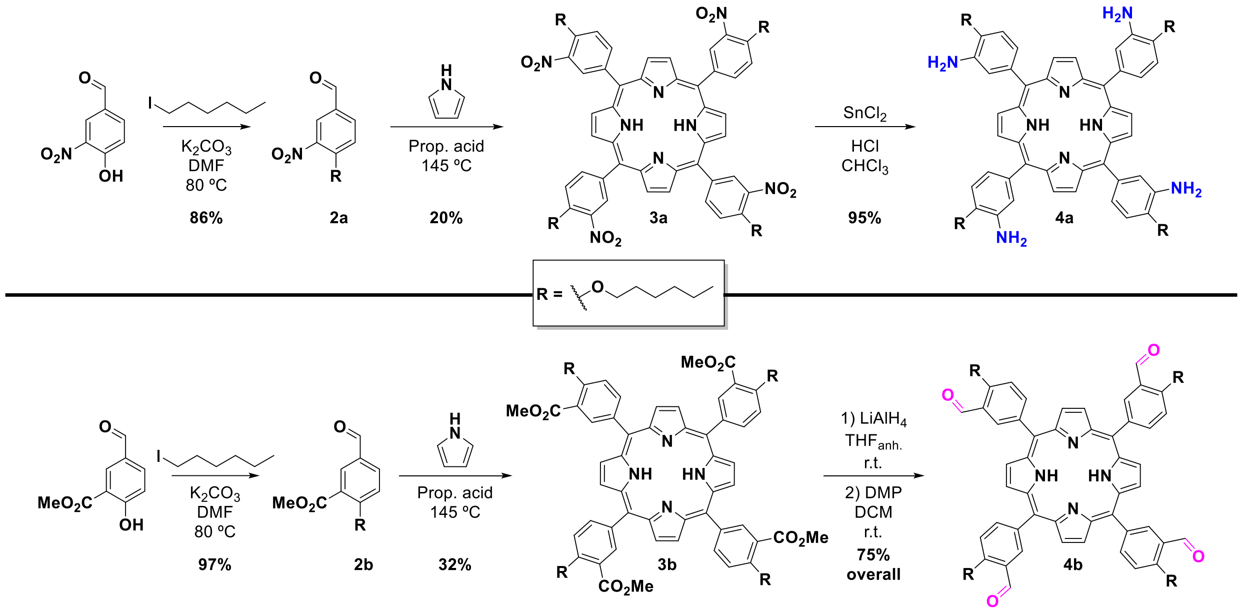

Scheme S1. Synthesis of tetra-amino-porphyrin **4a** and tetra-formyl-porphyrin **4b**.

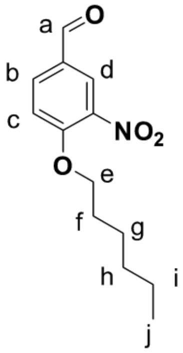

2a. Commercial 4-hidroxy-3-nitrobenzaldehyde (10.43 g, 62.4 mmol, 1 eq) and K_2CO_3 (17.23 g, 123.8 mmol, 2 eq) were dissolved in DMF (133 mL). Then, 1-iodohexane (13.88 mL, 93.6 mmol, 1.5 eq) was added and the mixture was heated at 80 ºC, overnight. Upon the reaction was completed, the solvent was removed under vacuum, redissolved in ethyl acetate and washed successively with an aqueous solution of LiCl (5 M) and brine. The organic layer was dried with $MgSO_4$, filtered through cotton plug and the solvent removed under reduced pressure. The crude product was purified by silica chromatography column using a mixture of cyclohexane:EtOAc (9:1) as eluent, achieving the product **2a** as a yellow oil (13.44 g, Y = 86%).

^{1}H NMR (300 MHz, $CDCl_3$) δ (ppm) = 9.93 (s, 1H, H^a), 8.33 (d, J = 2.1 Hz, 1H, H^d), 8.10 – 8.02 (dd, J = 8.6, 2.0 Hz, 1H, H^b), 7.20 (d, J = 8.7 Hz, 1H, H^c), 4.21 (t, J = 6.4 Hz, 2H, H^e), 1.96 – 1.79 (m, 2H, H^f), 1.61 – 1.44 (m, 2H, H^g), 1.42 – 1.25 (m, 4H, H^{h+i}), 0.90 (t, J = 6.8 Hz, 3H, H^j).

^{13}C NMR (75 MHz, $CDCl_3$) δ (ppm) = 188.9, 156.8, 140.0, 134.7, 128.8, 127.3, 114.6, 70.5, 31.4, 28.7, 25.5, 22.5, 14.0.

HRMS (APCI+): Mass m/z calculated for $C_{13}H_{18}NO_4$ $[M+H]^+$ 252.1158, found: 252.1128.

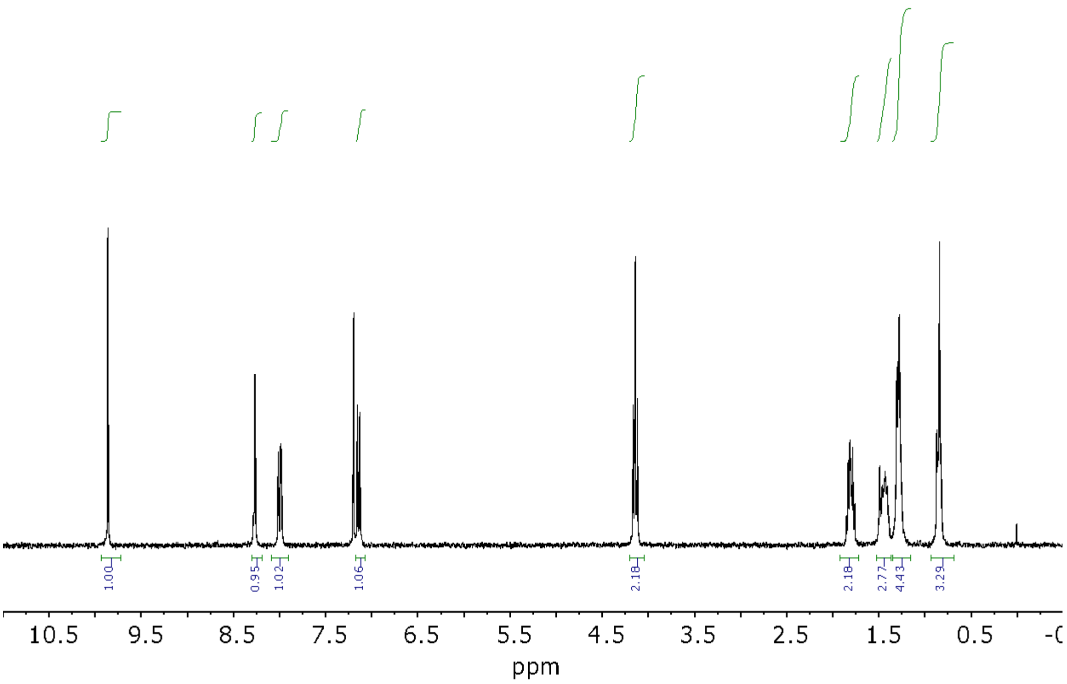

^{1}H NMR spectra ($CDCl_3$) of compound **2a**.

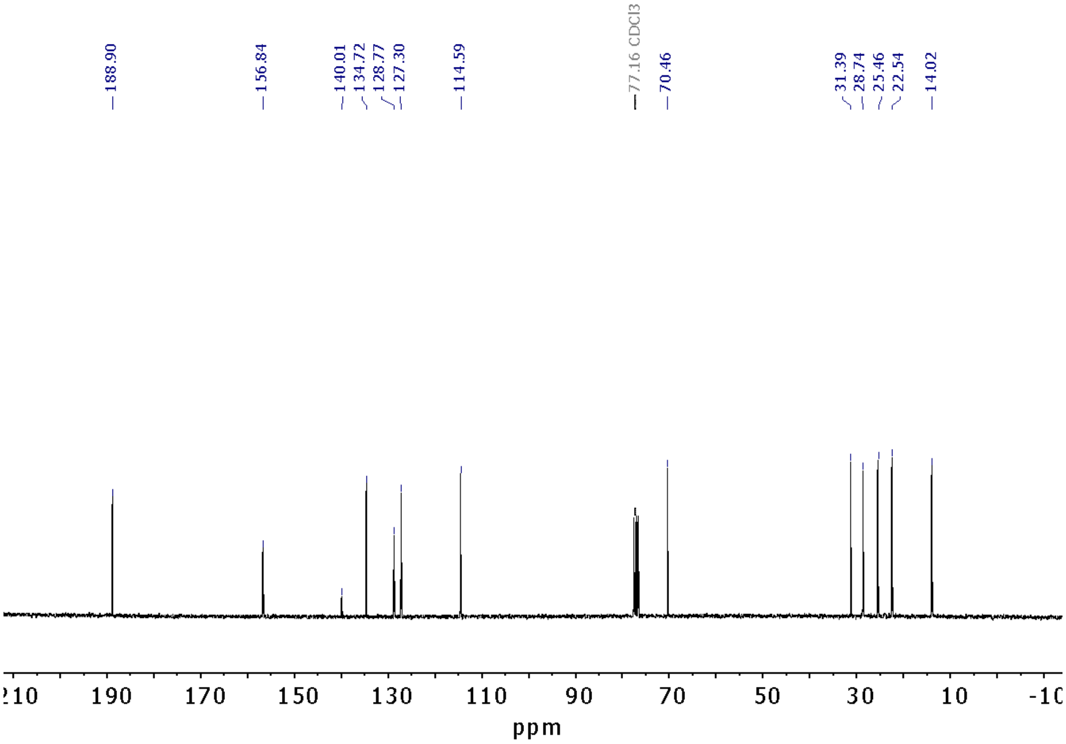

^{13}C NMR spectra ($CDCl_3$) of compound **2a**.

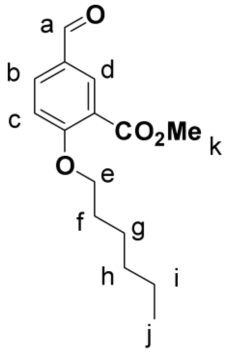

2b. Commercial methyl 5-formyl-2-hydroxybenzoate (8.4 g, 46.8 mmol, 1 eq) and K_2CO_3 (12.9 g, 93.6 mmol, 1.5 eq) were dissolved in DMF (100 mL). Then, 1-iodohexane (10.4 mL, 70.2 mmol, 1.5 eq) was added and the mixture was heated at 80 ºC, overnight. Upon the reaction was completed, the solvent was removed under vacuum, redissolved in ethyl acetate and washed successively with an aqueous solution of LiCl (5M) and brine. The organic layer was dried with $MgSO_4$, filtered through cotton plug and the solvent removed under reduced pressure. The crude product was purified by silica chromatography column using a mixture of cyclohexane:EtOAc (8:2) as eluent, achieving the product **2b** as a yellow oil (12.0 g, Y = 97%).

^{1}H NMR (300 MHz, $CDCl_3$) δ (ppm) = 9.90 (s, 1H, H^a), 8.31 (d, J = 2.2 Hz, 1H, H^d), 7.99 (dd, J = 8.7, 2.2 Hz, 1H, H^b), 7.07 (d, J = 8.7 Hz, 1H, H^c), 4.13 (t, J = 6.5 Hz, 2H, H^e), 3.91 (s, 3H, H^k), 1.94 – 1.79 (m, 2H, H^f), 1.61 – 1.25 (m, 6H, $H^{g\text{-}i}$), 0.90 (t, J= 6.9 Hz, 3H, H^j).

^{13}C NMR (76 MHz, $CDCl_3$) δ (ppm) = 190.2, 165.9, 163.3, 134.6, 134.4, 129.0, 120.9, 113.2, 69.5, 52.3, 31.5, 29.0, 25.7, 22.7, 14.1.

HRMS (ESI+): Mass m/z calculated for $C_{15}H_{21}O_4$ $[M+H]^+$ 265.1434, found: 265.1434.

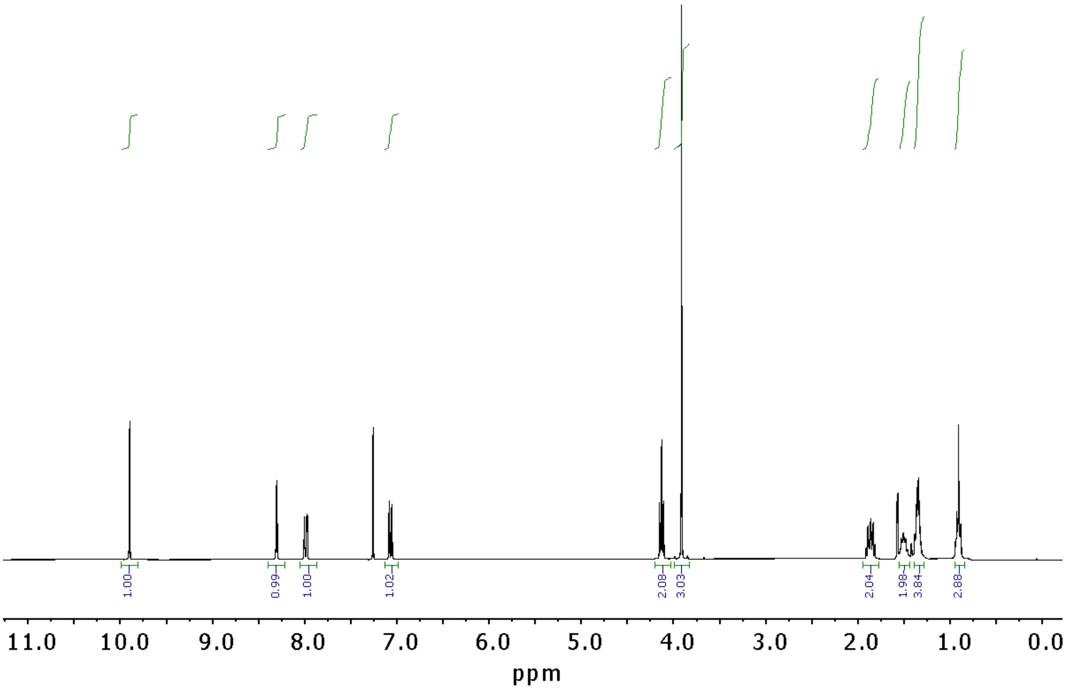

^{1}H NMR spectra ($CDCl_3$) of compound **2b**.

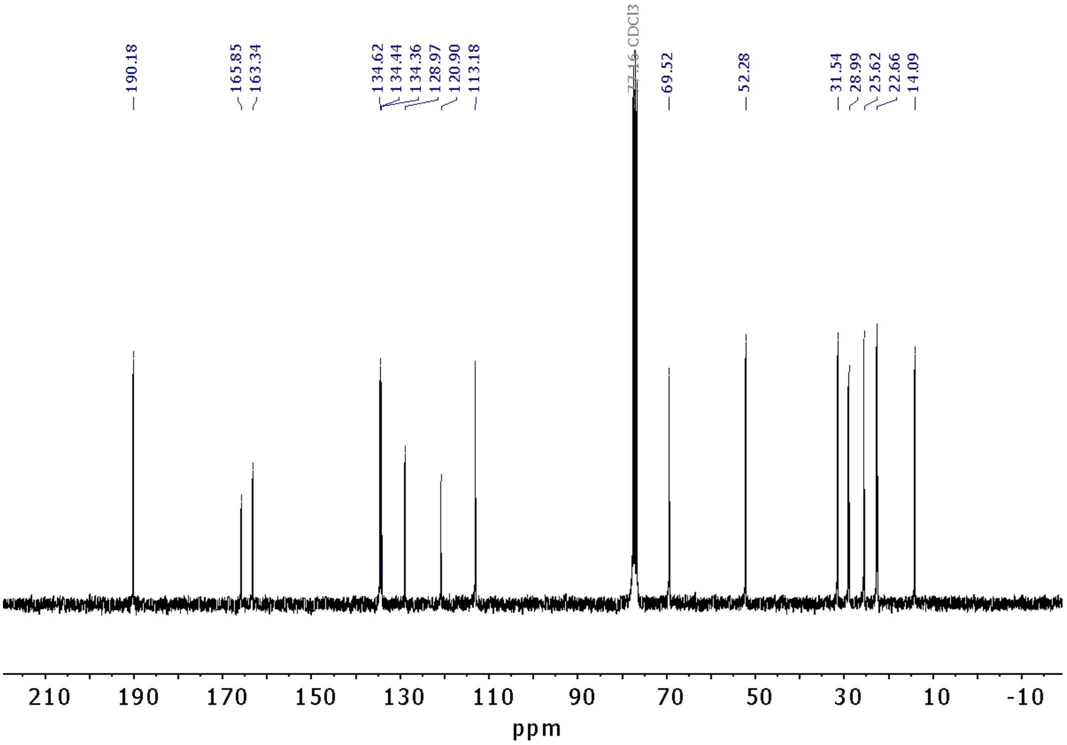

^{13}C NMR spectra ($CDCl_3$) of compound **2b**.

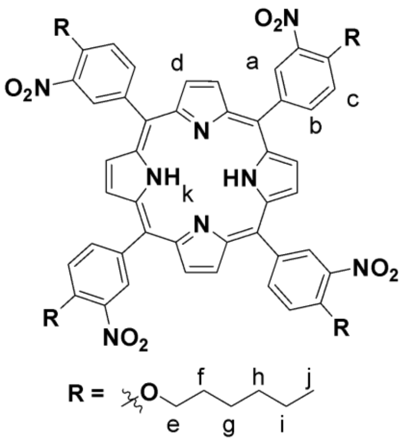

3a. Compound **2a** (13.73 g, 54.64 mmol, 1 eq) was dissolved in propionic acid (178 mL) under inert atmosphere. Upon heating at 145 ºC, fresh distilled pyrrole (3.79 mL, 54.64 mmol, 1 eq) was added and the mixture stirred at this temperature, keeping away from light, for 2 hours. Then, the heating source was removed, and the mixture stirred at room temperature, overnight. The resulting black solution was filtered and the solid rinsed with a small portion of propionic acid. Then, the solid was triturated with MeOH obtaining the desired compound **3a** as a purple solid (3.25 g, Y = 20%).

^{1}H NMR (300 MHz, $CDCl_3$) δ (ppm) = 8.88 (s, 8H, H^d), 8.66 (d, *J* = 2.2 Hz, 4H, H^a), 8.33 (d, *J* = 8.3 Hz, 4H, H^b), 7.48 (d, *J* = 8.5 Hz, 4H, H^c), 4.42 (t, *J* = 6.4 Hz, 8H, H^e), 2.14 – 1.94 (m, 8H, H^f), 1.77 – 1.61 (m, 8H, H^g), 1.58 – 1.37 (m, 16H, H^{h+i}), 1.00 (t, *J* = 7.0 Hz, 12H, H^j), –2.86 (s, 2H, H^k).

^{13}C NMR (76 MHz, $CDCl_3$) δ (ppm) = 152.6, 139.3, 138.7, 133.9, 131.5 (pyrr), 130.5, 117.9, 113.0, 77.4, 70.3, 31.7, 29.3, 25.9, 22.8, 14.2.

HRMS (ESI+): Mass m/z calculated for $C_{68}H_{75}N_8O_{12}$ [M+H]$^+$ 1195.5499, found: 1195.5494.

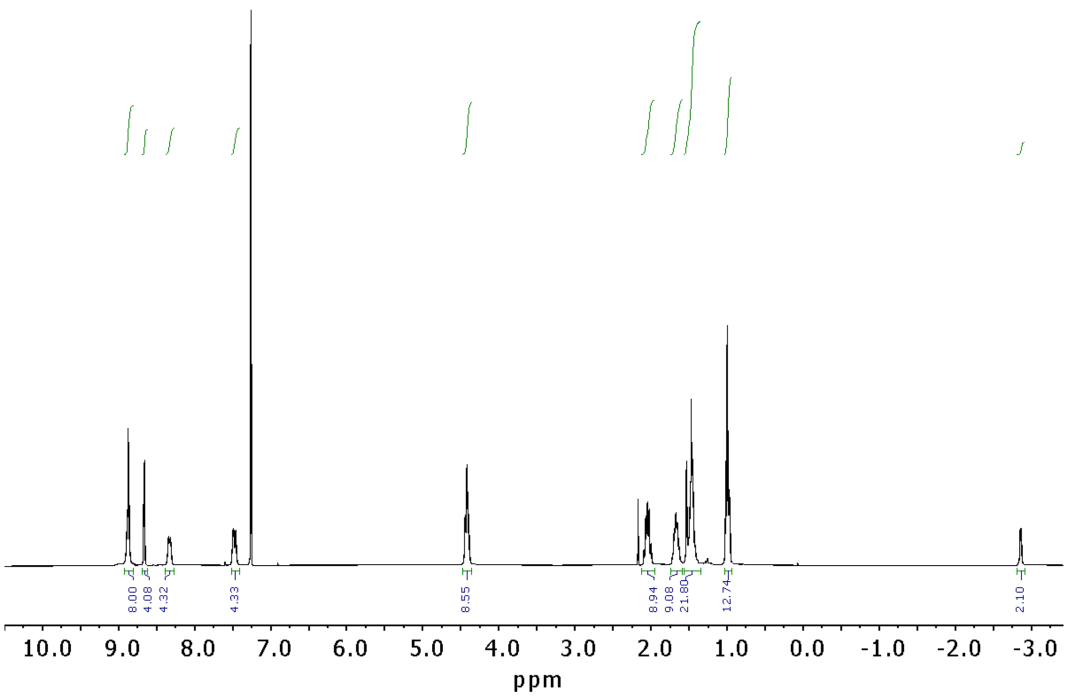

^{1}H NMR spectra ($CDCl_3$) of compound **3a**.

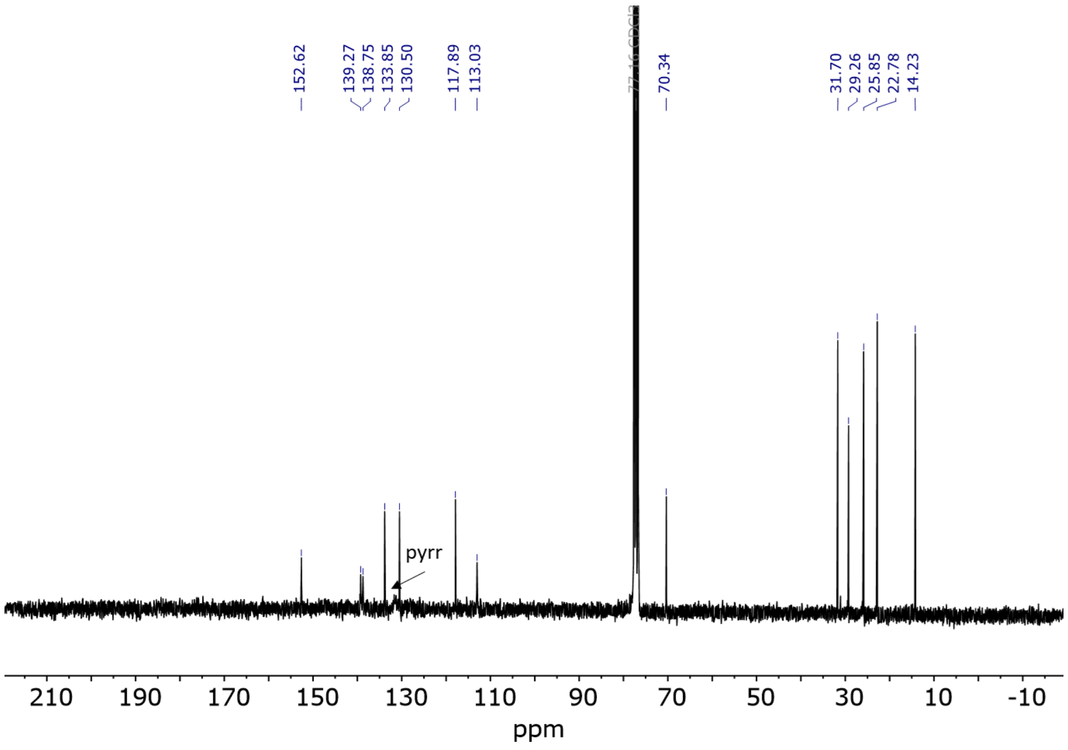

^{13}C NMR spectra ($CDCl_3$) of compound **3a**.

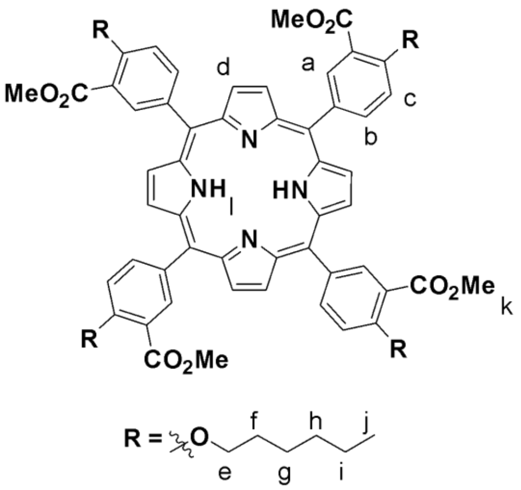

3b. Compound **2b** (5.73 g, 21.7 mmol, 1 eq) was dissolved in propionic acid (71 mL) under inert atmosphere. Upon heating at 145 ºC, fresh distilled pyrrole (1.50 mL, 21.7 mmol, 1 eq) was added and the mixture stirred at this temperature, keeping away from light, for 2 hours. Then, the heating source was removed, and the mixture stirred at room temperature, overnight. The resulting black solution was filtered and the solid rinsed with a small portion of propionic acid. Then, the solid was triturated with MeOH obtaining the desired compound **3b** as a purple solid (2.18 g, Y = 32%).

^{1}H NMR (300 MHz, $CDCl_3$) δ (ppm) = 8.86 (s, 8H, H^d), 8.63 (d, J = 2.3 Hz, 4H, H^a), 8.25 (dd, J = 7.9, 2.3 Hz, 4H, H^b), 7.35 (d, J = 7.9 Hz, 4H, H^c), 4.34 (t, J = 6.5 Hz, 8H, H^e), 3.94 (s, 12H, H^k), 2.15 – 1.97 (m, 8H, H^f), 1.78 – 1.37 (m, 24H, H^{g-i}), 1.00 (t, J = 7.0 Hz, 12H, H^j), -2.80 (s, 2H, H^l).

^{13}C NMR (76 MHz, $CDCl_3$) δ (ppm) = 167.1, 158.6, 138.9, 137.0, 133.9, 131.3, 119.0, 111.6, 69.5, 52.2, 31.8, 29.5, 26.0, 22.8, 14.2.

HRMS (ESI+): Mass m/z calculated for $C_{76}H_{87}N_4O_{12}$ $[M+H]^+$ 1247.6315, found: 1247.6305.

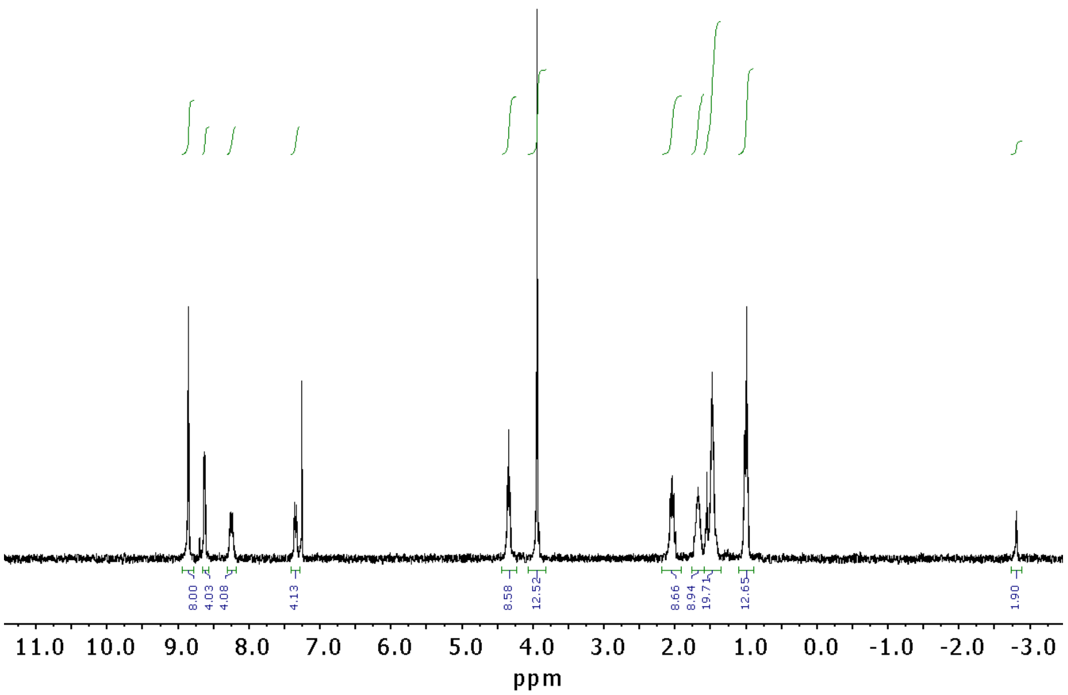

^1H NMR spectra ($CDCl_3$) of compound **3b**.

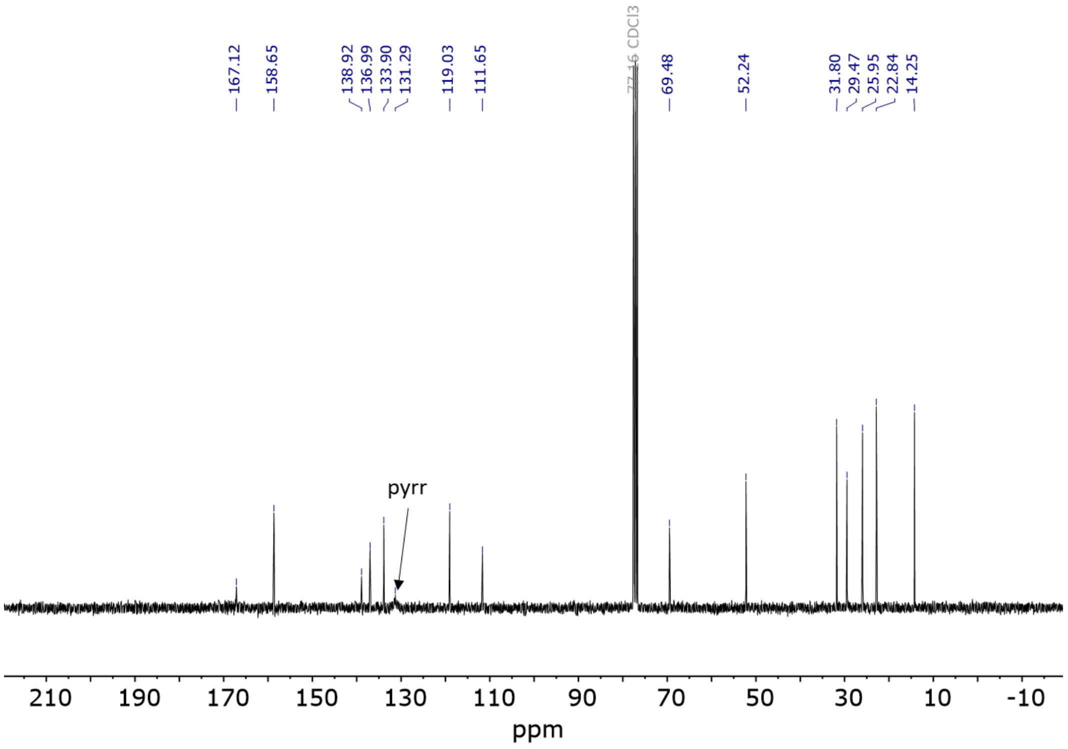

^{13}C NMR spectra ($CDCl_3$) of compound **3b**.

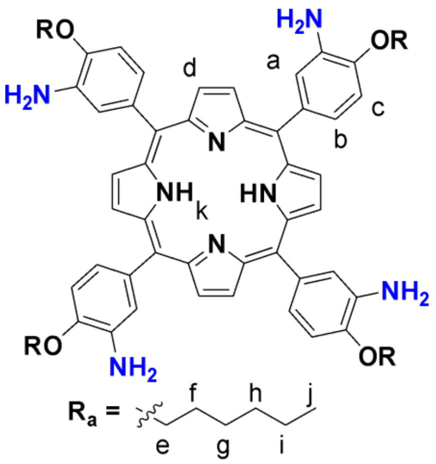

4a. Compound **3a** (0.66 g, 0.55 mmol, 1 eq.) and $SnCl_2$ (1.68 g, 8.83 mmol, 16 eq.) were dissolved in $CHCl_3$ (146 mL) under inert atmosphere. Commercial HCl (8.14 mL, 37%) was injected, and the mixture heated at reflux, keeping it away from light, overnight. Upon this time, the reaction crude was washed with aqueous solution of NaOH (1N) until the organic layer colour changed from green to red-purple, and then the organic phase was dried with Na_2SO_4, the solid was filtered and the solvent removed under vacuum. The resulting solid was triturated with MeOH and filtered obtaining the desired product as a purple solid (0.56 g, Y = 95%).

^{1}H-RMN (300 MHz, $CDCl_3$) δ (ppm) = 8.91 (s, 8H, H^d), 7.60 (d, J = 2.1 Hz, 4H, H^a), 7.52 (dd, J = 8.1, 2.1 Hz, 4H, H^b), 7.12 (d, J = 8.1 Hz, 4H, H^c), 4.28 (t, J = 6.5 Hz, 8H, H^e), 4.06 (s, 8H, **NH_2**), 2.08 – 1.95 (m, 8H, H^f), 1.73 – 1.23 (m, 24H, H^{g+h+i}), 0.99 (t, J = 7.1 Hz, 12 H, H^j), -2.76 (s, 2H, H^k).

^{13}C NMR (76 MHz, $CDCl_3$) δ (ppm) = 146.6, 135.0, 134.1, 131.9 (pyrr), 125.8, 122.1, 120.0, 109.4, 77.2, 68.6, 31.8, 29.6, 26.0, 22.7, 14.1.

HRMS (MALDI): Mass m/z calculated for $C_{68}H_{82}N_8O_4$ $[M+H]^+$ 1074.6454, found: 1074.6436.

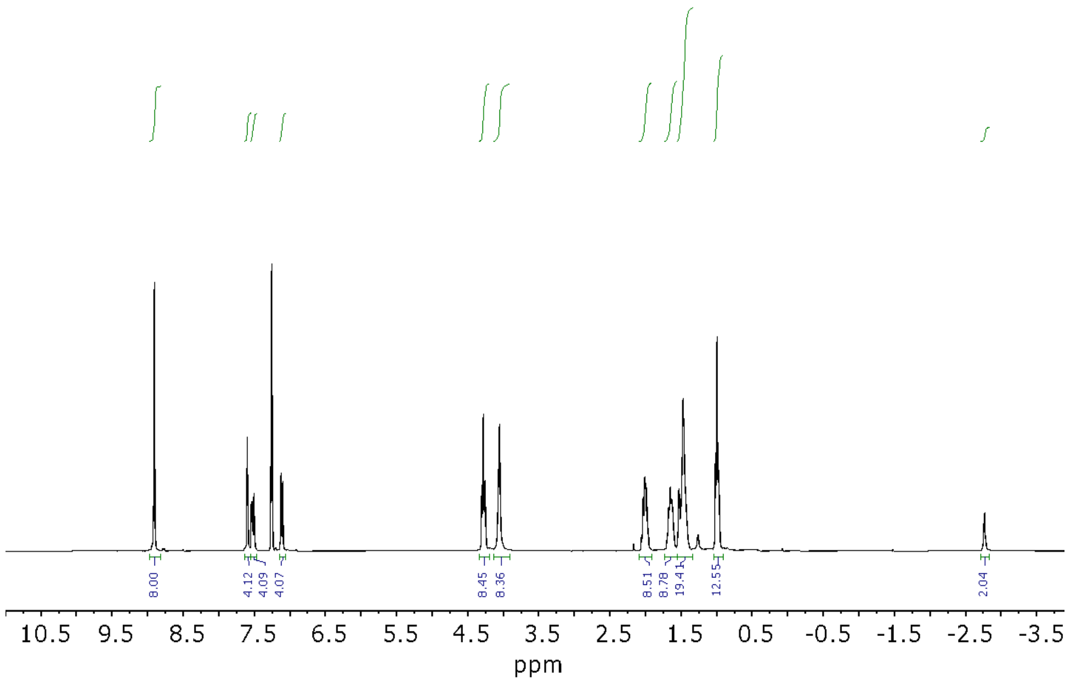

^{1}H NMR spectra ($CDCl_3$) of **4a**.

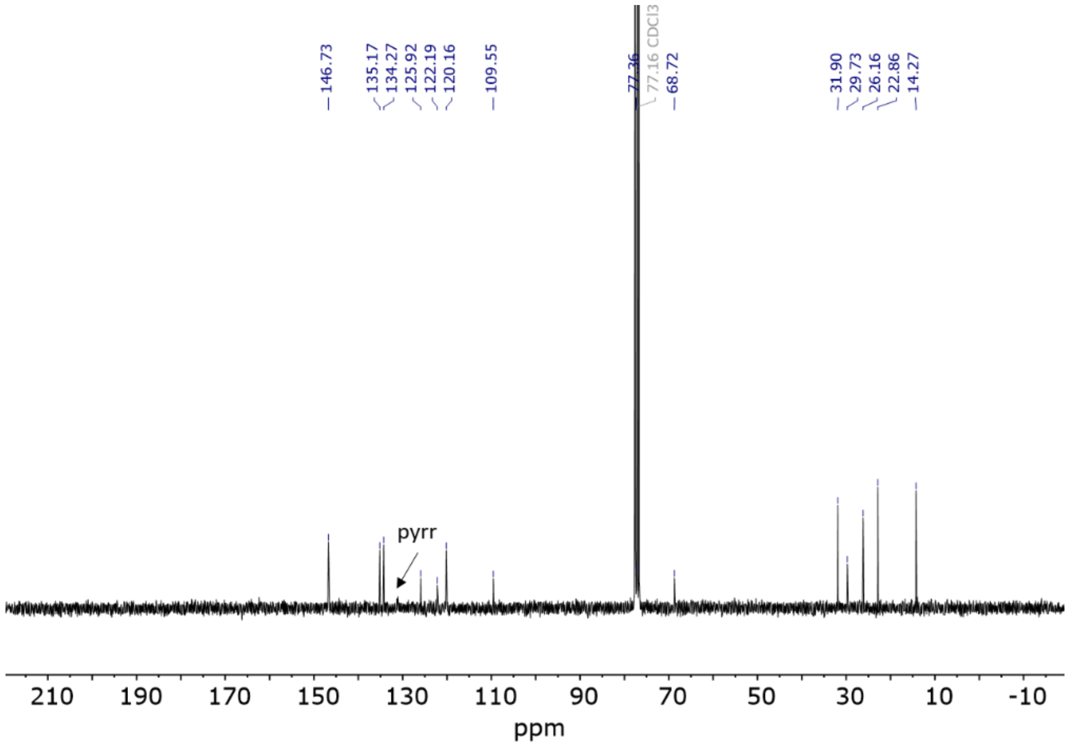

^{13}C NMR spectra ($CDCl_3$) of **4a**.

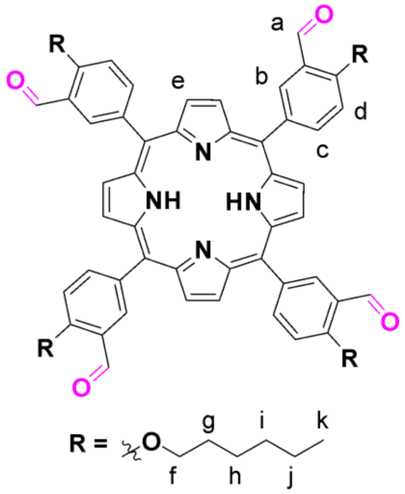

4b. Compound **3b** (0.66 g, 0.53 mmol, 1 eq.) was dissolved in anhydrous THF (60 mL) under argon atmosphere. Then, 1M $LiAlH_4$ solution (2.1 mL, 2,1 mmol, 4 eq) was injected dropwise at 0º C, and stirred at room temperature 24 hours. Later, the excess of $LiAlH_4$ was quenched by addition of EtOAc (3 mL) followed by water (5 mL). Upon generation of H_2 was not observed, the mixture was washed with water (30 mL), the organic layer was dried with Na_2SO_4, the solid was filtered and the solvent removed under vacuum. The resulting purple solid (0.58 g) was used in the next step without further purification. To a solution of tetra-alcohol intermedia (0.58 g, 0.52 mmol, 1 eq) in DCM (30 mL), Dess-Martin Periodinane (0.24 g, 0.57 mmol, 10 eq) was added. The reaction mixture was stirred at room temperature overnight. Upon the reaction was completed the solvent was removed under vacuum and the crude was purified by silica chromatography column using DCM as eluent, achieving the product **4b** as a purple solid (0.44 g, Y = 75% over two steps).

^{1}H NMR (300 MHz, $CDCl_3$) δ (ppm) = 10.84 (s, 4H, H^a), 8.80 (s, 8H, H^e), 8.67 (bs, 4H, H^b), 8.33 (dd, J = 8.3, 2.3 Hz, 4H, H^c), 7.36 (d, J = 8.3 Hz, 4H, H^d), 4.38 (t, J = 6.4 Hz, 8H, H^f), 2.16 – 1.94 (m, 8, H^g), 1.84 – 1.21 (m, 24H, $H^{h\text{-}j}$), 1.00 (t, J = 6.9 Hz, 12H, H^k), –2.80 (s, 2H, **NH**).

^{13}C NMR (76 MHz, $CDCl_3$) δ (ppm) = 190.2, 161.5, 141.3, 134.4, 133.3, 131.2, 123.4, 118.9, 111.2, 69.1, 31.7, 29.3, 26.0, 22.8, 14.2.

HRMS (ESI+): Mass m/z calculated for: $C_{72}H_{79}N_4H_8$ $[M+H]^+$ 1127.5892, found: 1127.5876.

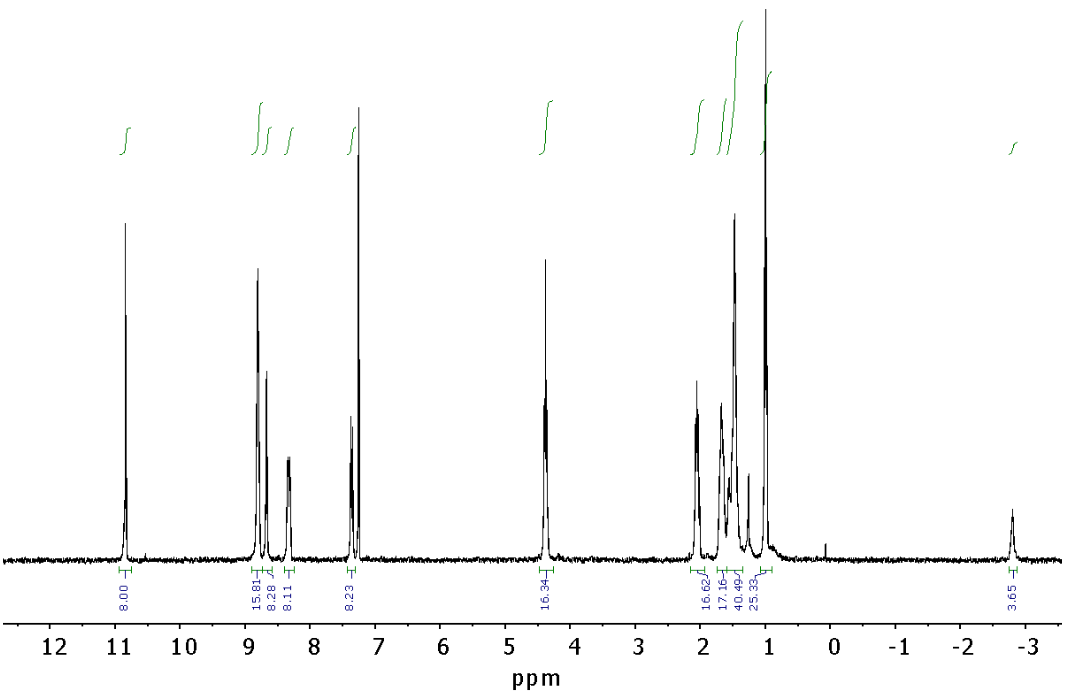

^{1}H NMR spectra ($CDCl_3$) of **4b**.

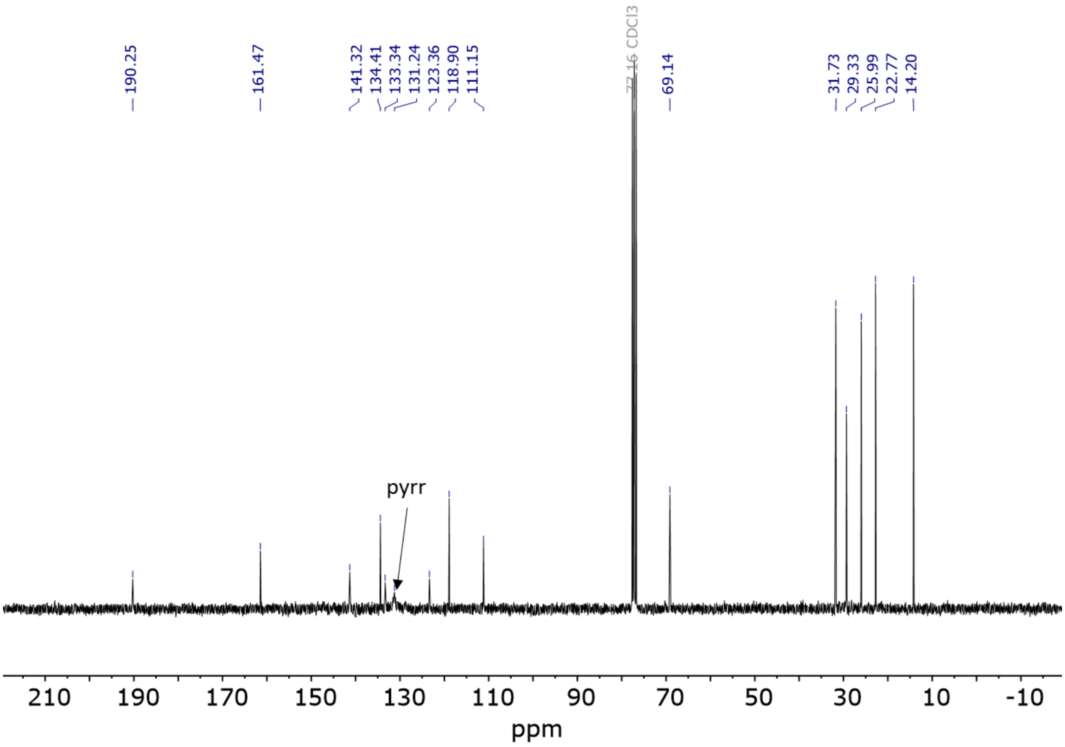

^{13}C NMR spectra ($CDCl_3$) of **4b**.

Synthesis of *m*-di-formyl and *m*-diamine linkers.

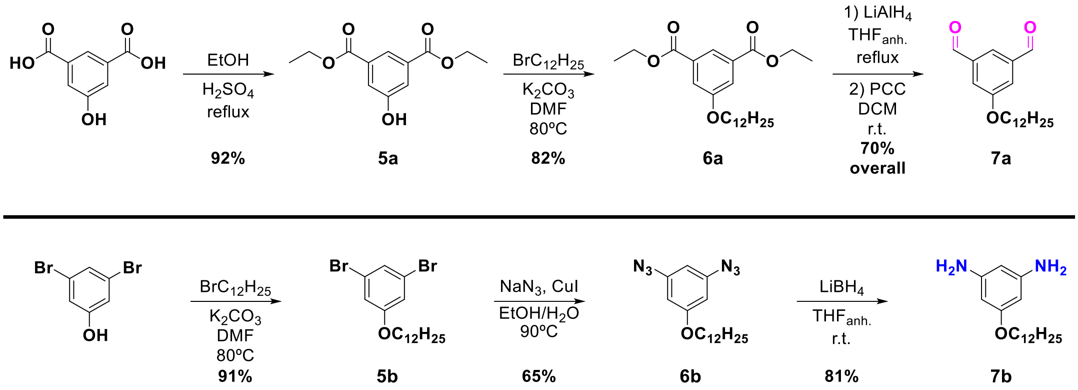

Scheme S2. Synthesis of linkers **7a** and **7b.**

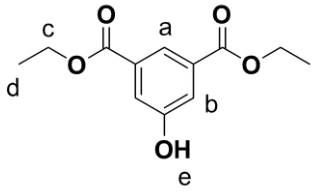

5a. This compound was prepared according to a previously reported procedure.[1] A catalytic amount of H_2SO_4 98% was added to a solution of 5-hydroxyisophthalic acid (5.03 g, 27.5 mmol, 1 eq) in EtOH (125 mL) and the mixture was heated at reflux, overnight. Then, the solvent was removed under vacuum and the crude purified by silica chromatography column using a mixture of cyclohexane:EtOAc (9:1) as eluent, obtaining the product **6** as a white solid (6.0 g, 92%).

^{1}H NMR (300 MHz, $CDCl_3$) δ (ppm) = 8.27 (t, J = 1.5 Hz, 1H, H^a), 7.72 (d, J = 1.4 Hz, 2H, H^b), 5.35 (bs, 1H, H^e), 4.40 (q, J = 7.1 Hz, 4H, H^c), 1.41 (t, J = 7.1 Hz, 6H, H^d).

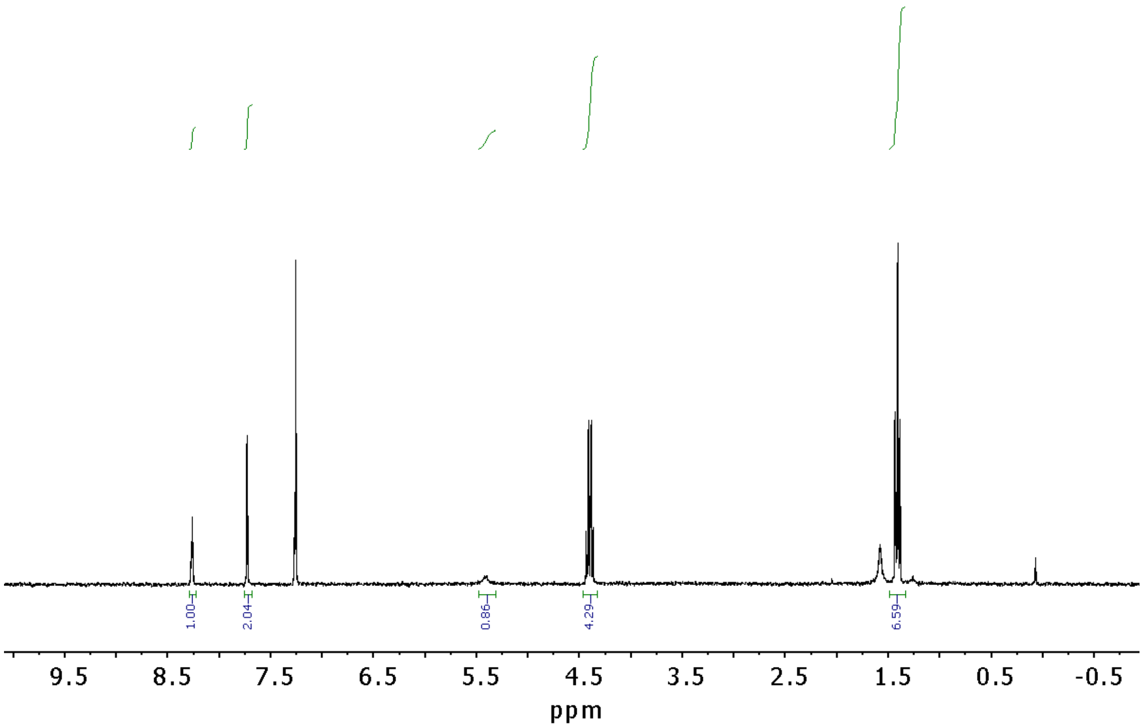

^{1}H NMR spectra ($CDCl_3$) of **5a**.

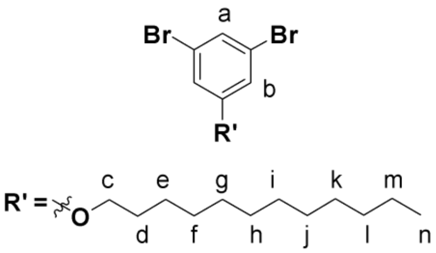

5b. This compound was prepared according to a previously reported procedure with slight modifications.[2] 3,5-dibromophenol (3.70 g, 14.70 mmol, 1 eq) and K_2CO_3 (4.06 g, 29.40 mmol, 2.0 eq) were dissolved in DMF (41 mL). Then, 1-bromododecane (5.30 mL, 22.05 mmol, 1.5 eq) was added and the mixture was heated at 80 ºC, overnight. Upon the reaction was completed, the solvent was removed under vacuum, redissolved in EtOAc and washed successively with an aqueous solution of LiCl (5M) and brine. The organic layer was dried with $MgSO_4$, filtered through a cotton plug and the solvent removed under reduced pressure. The crude product was purified by silica chromatography column using cyclohexane as eluent, achieving the product **5b** as a yellow oil (5.60 g, 91%).

^{1}H NMR (300 MHz, $CDCl_3$) δ (ppm) = 7.22 (t, J = 1.6 Hz, 1H, H^a), 6.98 (d, J = 1.6 Hz, 2H, H^b), 3.91 (t, J = 6.5 Hz, 2H, H^c), 1.81 – 1.68 (m, 2H, H^d), 1.48 – 1.36 (m, 2H, H^e), 1.51 – 1.13 (m, 16H, $H^{f\text{-}m}$), 0.87 (t, J = 6.6 Hz, 3H, H^n).

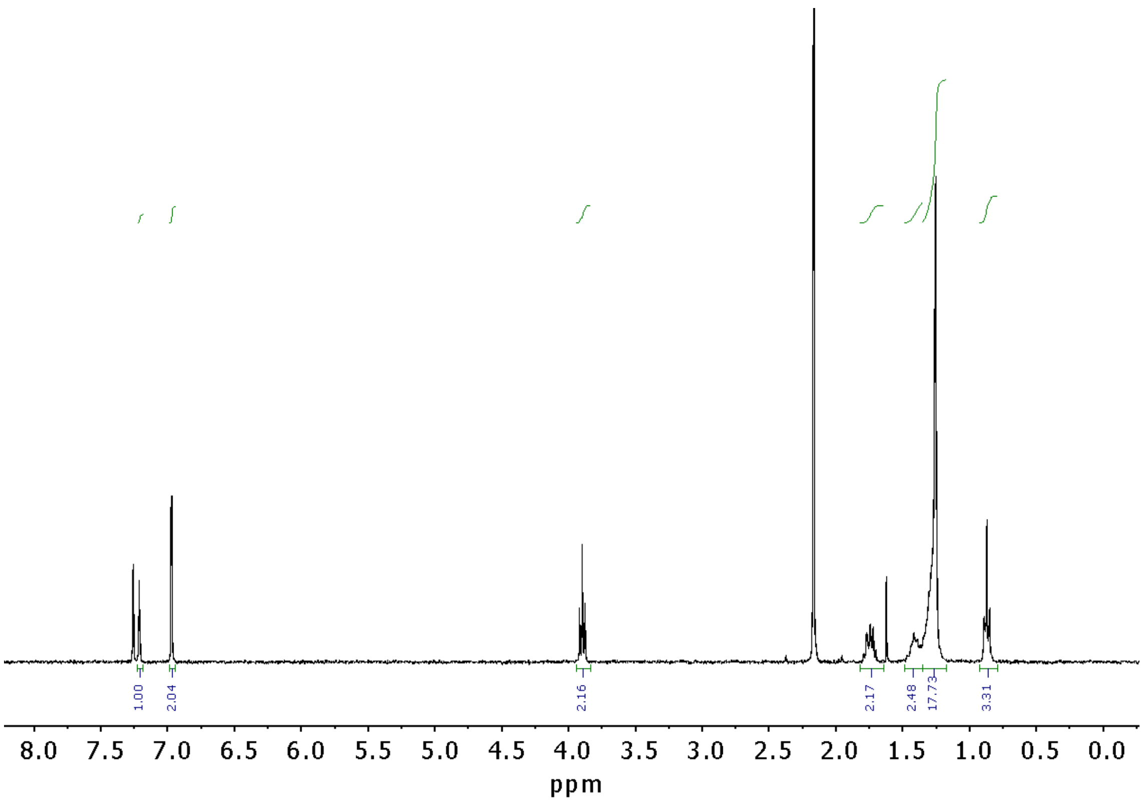

^{1}H NMR spectra ($CDCl_3$) of **5b**.

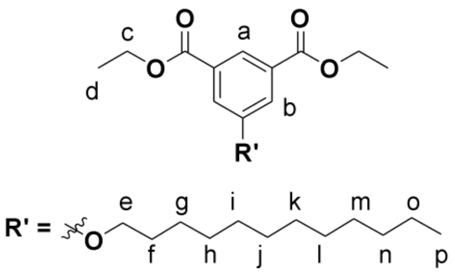

6a. Compound **5a** (3.37 g, 13.0 mmol, 1 eq) and K_2CO_3 (2.76 g, 20 mmol, 1.5 eq) were dissolved in DMF (46 mL). Then, 1-bromododecane (4.79 mL, 20.0 mmol, 1.5 eq) was added and the mixture was heated at 80 ºC, overnight. Upon the reaction was completed, the solvent was removed under vacuum, redissolved in EtOAc and washed successively with an aqueous solution of LiCl (5M) and brine. The organic layer was dried with $MgSO_4$, filtered through a cotton plug and the solvent removed under reduced pressure. The crude product was purified by silica chromatography column using a mixture of cyclohexane:EtOAc (7:3) as eluent, achieving the product **7** as a yellow oil (4.3 g, 82%).

^{1}H NMR (300 MHz, $CDCl_3$) δ (ppm) = 8.26 (t, J = 1.5 Hz, 1H, H^a), 7.73 (d, J = 1.5 Hz, 2H, H^b), 4.40 (q, J = 7.1 Hz, 4H, H^c), 4.03 (t, J = 6.5 Hz, 2H, H^e), 1.80 (p, J = 6.5 Hz, 2H, H^f), 1.52 – 1.18 (m, 24H, $H^{d+g\text{-}o}$), 0.94 – 0.85 (t, J = 7.1 Hz, 3H, H^p).

^{13}C NMR (76 MHz, $CDCl_3$) δ (ppm) = 165.9, 159.3, 132.2, 122.8, 119.8, 68.7, 61.5, 32.0, 29.8, 29.8, 29.7, 29.7, 29.5, 29.3, 26.1, 22.8, 14.4, 14.2.

HRMS (ESI+): Mass m/z calculated for $C_{24}H_{39}O_5$ $[M+H]^+$ 407.2792, found: 407.2801.

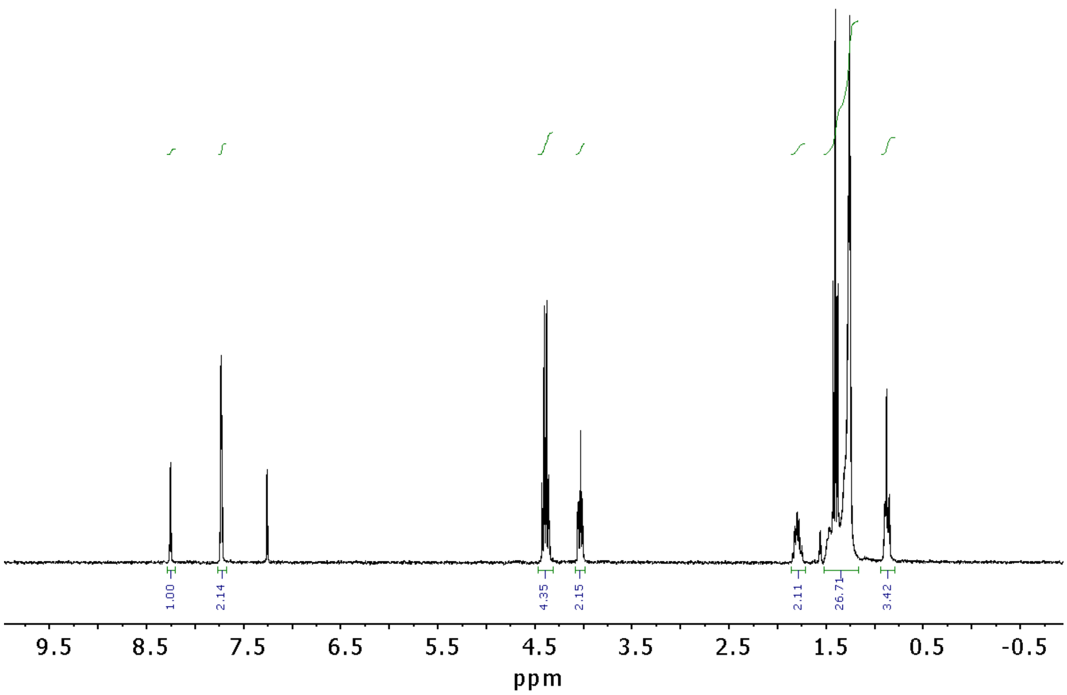

^{1}H NMR spectra ($CDCl_3$) of **6a**.

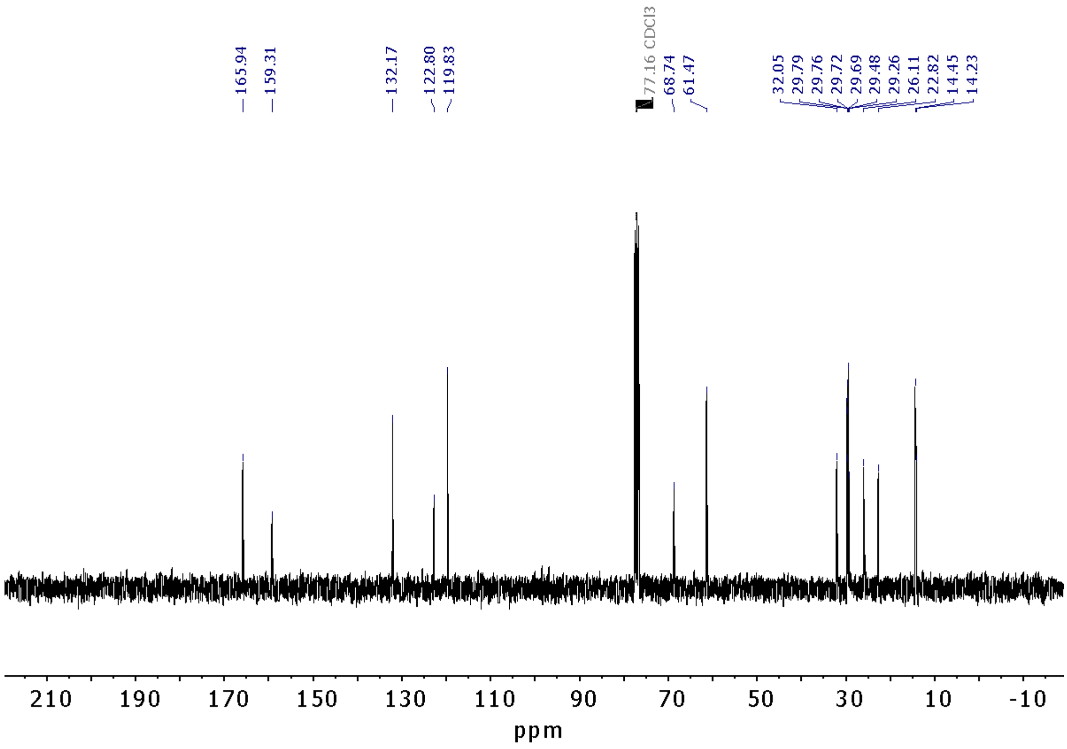

^{13}C NMR spectra ($CDCl_3$) of **6a**.

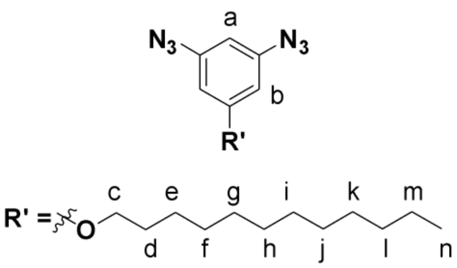

6b. Compound **5b** (1.51 g, 3.6 mmol, 1 eq), ascorbic acid (0.51 g, 2.87 mmol, 0.8 eq) and CuI (0.55 g, 2.87 mmol, 0.8 eq) placed in a Schlenk tube were dissolved in a degassed mix of EtOH/H_2O (7:1, 16 mL) under argon atmosphere. *N*,*N*-dimethylethylenediamine (0.31 mL, 2.87 mmol, 0.8 eq) and NaN_3 (0.94 g, 14.4 mmol, 4 eq) were added. Then, the reaction mixture was heated at 90 ºC, overnight, protected by a methacrylate shield. Once the mixture was cold, EtOAc was added, and the crude washed with a saturated EDTA aqueous solution (twice) and brine. The organic phase was dried with $MgSO_4$, the solid filtrated and the solvent removed under vacuum. The crude was purified by silica chromatography column using cyclohexane as eluent, obtaining the isolated product **6b** as a yellow solid (0.80 g, 65%).

^{1}H NMR (300 MHz, $CDCl_3$) δ (ppm) = 6.34 (d, J = 2.0 Hz, 2H, H^b), 6.27 (t, J = 2.0 Hz, 1H, H^a), 3.92 (t, J = 6.5 Hz, 2H, H^c), 1.82 – 1.60 (m, 2H, H^d), 1.50 – 1.00 (m, 18H, H^{e-m}), 0.94 – 0.80 (t, J = 6.1 Hz, 3H, H^n).

^{13}C NMR (76 MHz, $CDCl_3$) δ (ppm) = 161.4, 142.7, 102.3, 102.2, 68.6, 32.1, 29.8, 29.8, 29.7, 29.7, 29.5, 29.2, 26.1, 22.8, 14.2.

MS (ESI+): The molecular ion of this molecule could not be found due to the instability of the azide group.

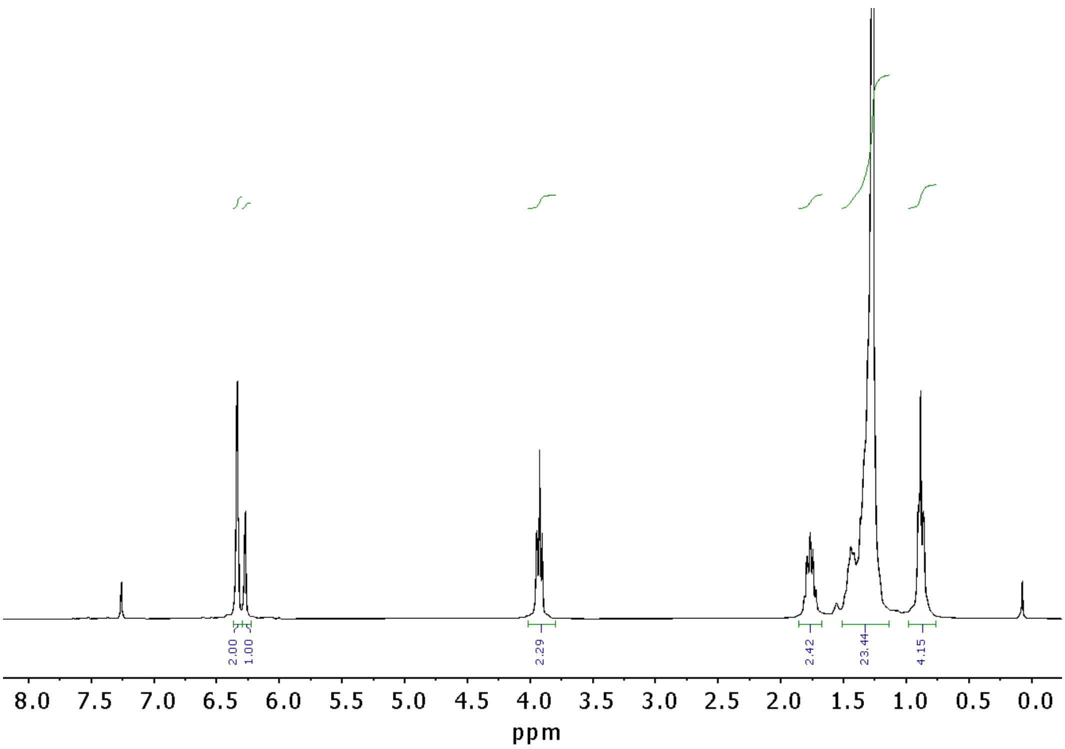

^{1}H NMR spectra ($CDCl_3$) of **6b**.

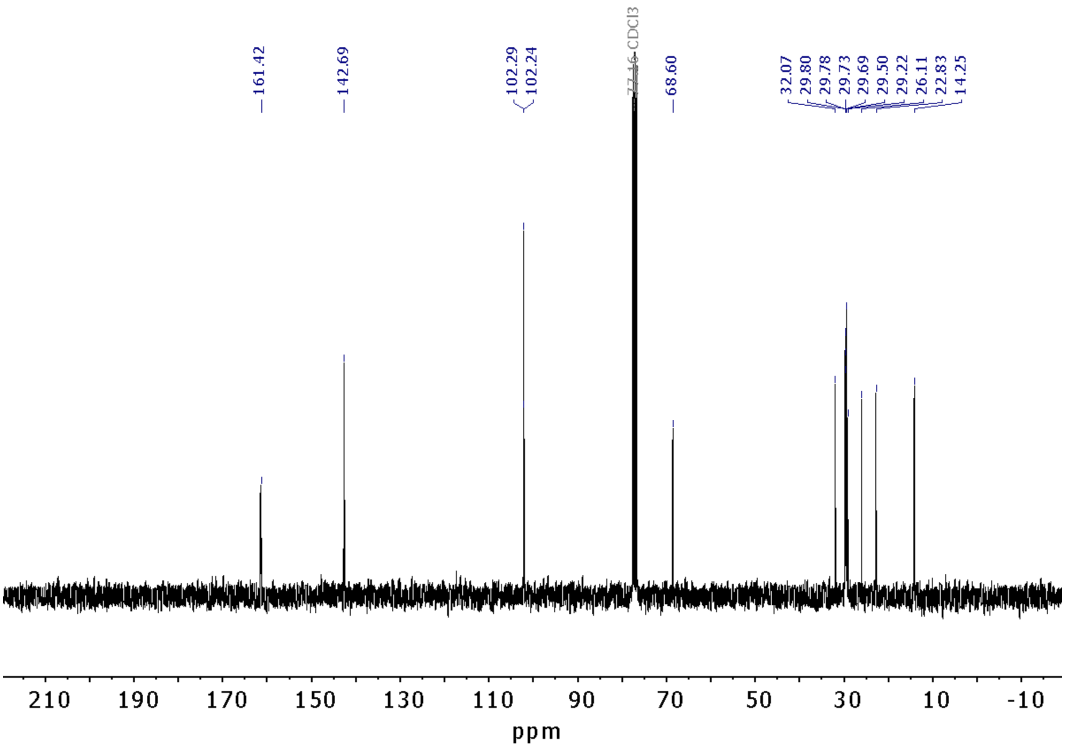

^{13}C NMR spectra ($CDCl_3$) of **6b**.

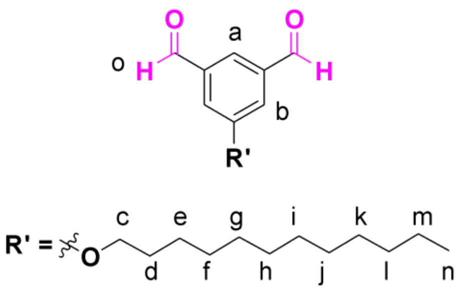

7a. To a solution of compound **6a** (2.18 g, 5.4 mmol, 1 eq) in anhydruous THF (22 mL) a solution of $LiAlH_4$ 1M (16.2 mL, 16.2 mmol, 3 eq) was added dropwise at 0 ºC, for 30 minutes. Then, the mixture was heated at reflux, overnight. Upon the starting material was consumed, the excess of $LiAlH_4$ was quenched with EtOAc (1.5 mL), EtOH (1.5 mL) and H_2O (5 mL) at 0 ºC. The mixture was dried over $MgSO_4$, filtered and the solvent removed under vacuum, obtaining the corresponding alcohol (1.25 g), which was used without further purification in the next step. A portion of the alcohol intermedia (1.04 g, 3.23 mmol, 1 eq), PCC (2.1 g, 9.69 mmol, 3 eq) and celite (1,00 g) were suspended in DCM (30 mL) and the mixture was stirred at room temperature, overnight. Upon the reaction was completed, the suspension was filtered through a silica plug rinsing with DCM. The solvent was removed under vacuum and the crude was purified by silica chromatography column using a mix of cyclohexane:DCM (1:1) as eluent, obtaining the isolated product **7a** as a white solid (0.71 g, 70%, over two steps).

^{1}H NMR (300 MHz, $CDCl_3$) δ (ppm) = 10.05 (s, 1H, H^o), 7.94 (t, J = 1.4 Hz, 1H, H^a), 7.64 (d, J = 1.4 Hz, 2H, H^b), 4.07 (t, J = 6.5 Hz, 2H, H^c), 1.89 – 1.77 (m, 2H, H^d), 1.52 – 1.44 (m, 2H, H^e), 1.40 – 1.20 (m, 14H, $H^{f\text{-}l}$), 0.89 (t, J = 5.9 Hz, 3H, H^n).

^{13}C NMR (126 MHz, $CDCl_3$) δ (ppm) = 191.1, 160.5, 138.4, 124.1, 120.0, 69.0, 32.0, 29.8, 29.8, 29.7, 29.7, 29.5, 29.4, 29.1, 26.1, 22.8, 14.2.

HRMS (ESI+): Mass m/z calculated for $C_{20}H_{30}O_3Na$ $[M+Na]^+$ 341.2087 found: 341.2090.

FT-IR (ATR): ν (cm^{-1}) = 2911 (C-H), 2847 (C-H), 1700 (C=O), 1381, 1301, 1141, 1021, 946, 718.

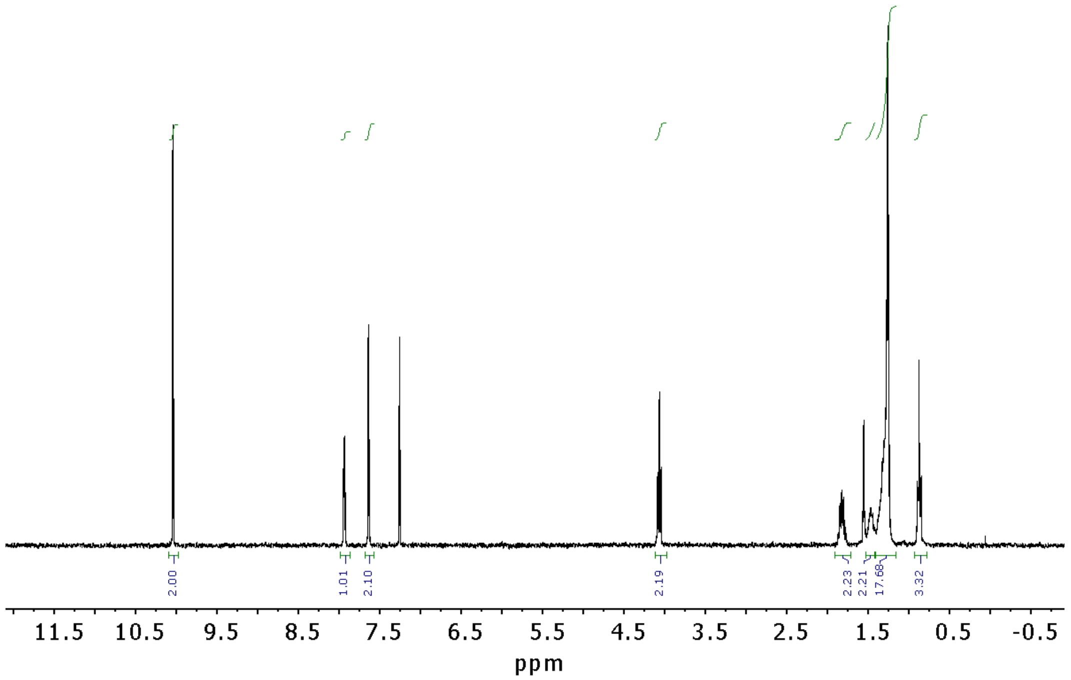

^{1}H NMR spectra ($CDCl_3$) of **7a**.

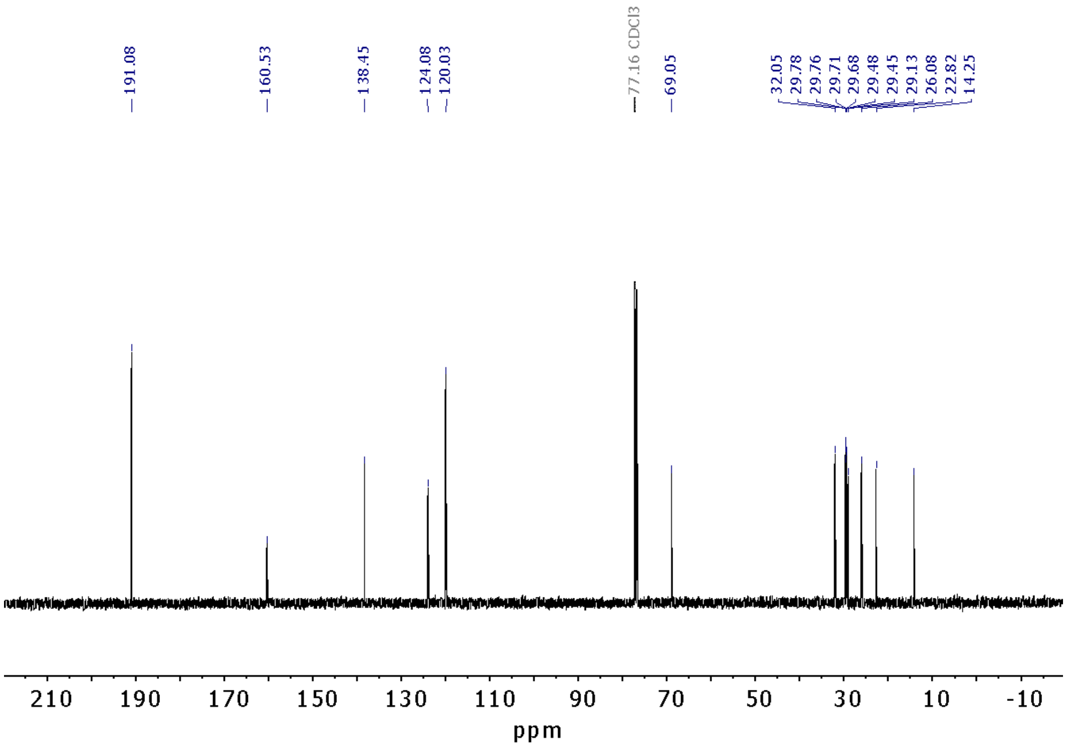

^{13}C NMR spectra ($CDCl_3$) of **7a**.

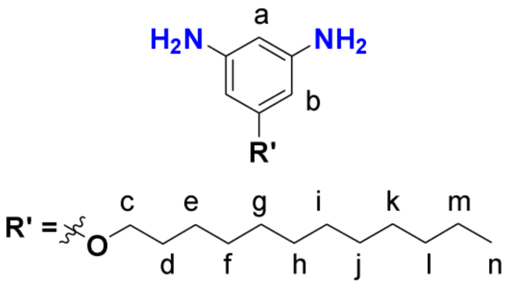

7b. To a solution of compound **6b** (0.59 g, 1.71 mmol, 1 eq) in anhydrous THF (12 mL), 2M solution of $LiBH_4$ in THF (1.72 mL, 3.43 mmol, 2 eq) was added dropwise under argon atmosphere, and the mixture was stirred at room temperature, overnight. Upon the reaction was completed, the excess of $LiBH_4$ was quenched by addition of EtOAc (5 mL) and water (5 mL). The organic phase was dried with $MgSO_4$, the solid was filtrated and the solvent removed under reduced pressure. The crude was purified by silica chromatography column (previously neutralized with 1% NEt_3 in cyclohexane) using a mix of cyclohexane:EtOAc (1:0 to 1:4) as eluent, isolating compound **7b** as a brown solid (0.40 g, 81%).

^{1}H NMR (300 MHz, $CDCl_3$) δ (ppm) = 5.71 (d, J = 1.9 Hz, 2H, H^b), 5.66 (t, J = 2.0 Hz, 1H, H^a), 3.86 (t, J = 6.6 Hz, 2H, H^c), 3.55 (s, 4H, **NH_2**), 1.72 (m, 2H, H^d), 1.50 – 1.13 (m, 18H, $H^{e\text{-}m}$), 0.88 (t, J = 6.5 Hz, 3H, H^n).

^{13}C NMR (76 MHz, $CDCl_3$) δ (ppm) = 160.9, 148.0, 94.5, 92.2, 67.2, 31.4, 29.2, 29.2, 29.1, 29.1, 28.9, 28.9, 25.6, 22.2, 13.6.

HRMS (ESI+): Mass m/z calculated for $C_{18}H_{33}N_2O$ $[M+H]^+$ 293.2515 found: 293.2580.

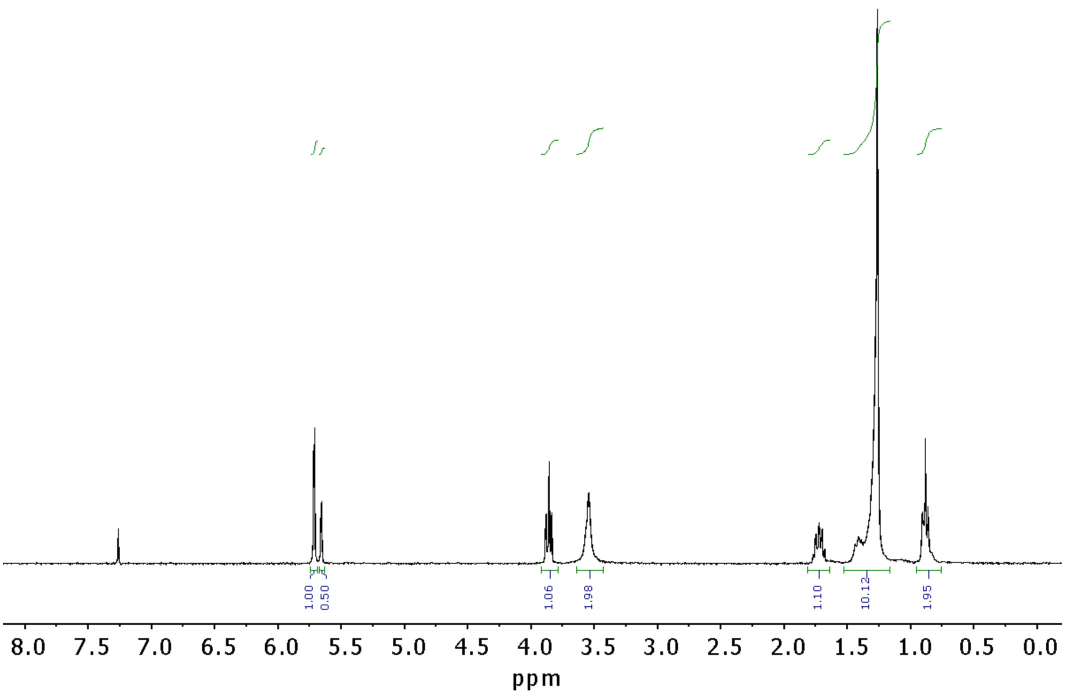

^{1}H NMR spectra ($CDCl_3$) of **7b**.

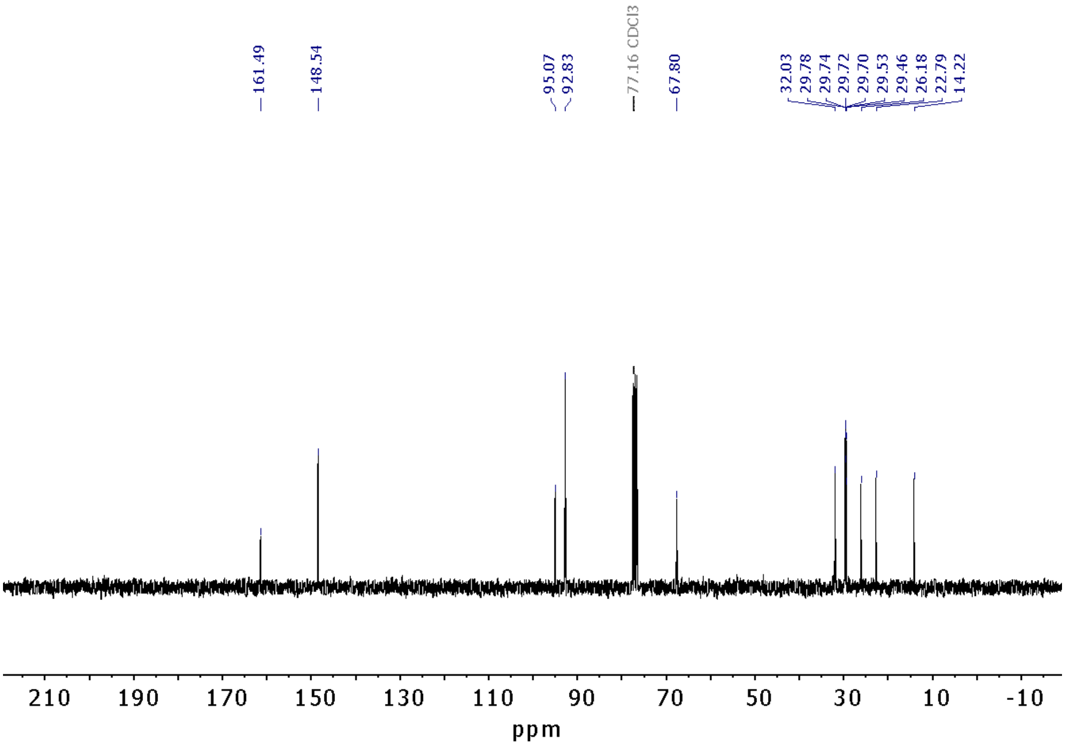

^{13}C NMR spectra ($CDCl_3$) of **7b**.

Synthesis of molecular cages $1_{Zn}{}^{NC}$ and $1_{Zn}{}^{CN}$.

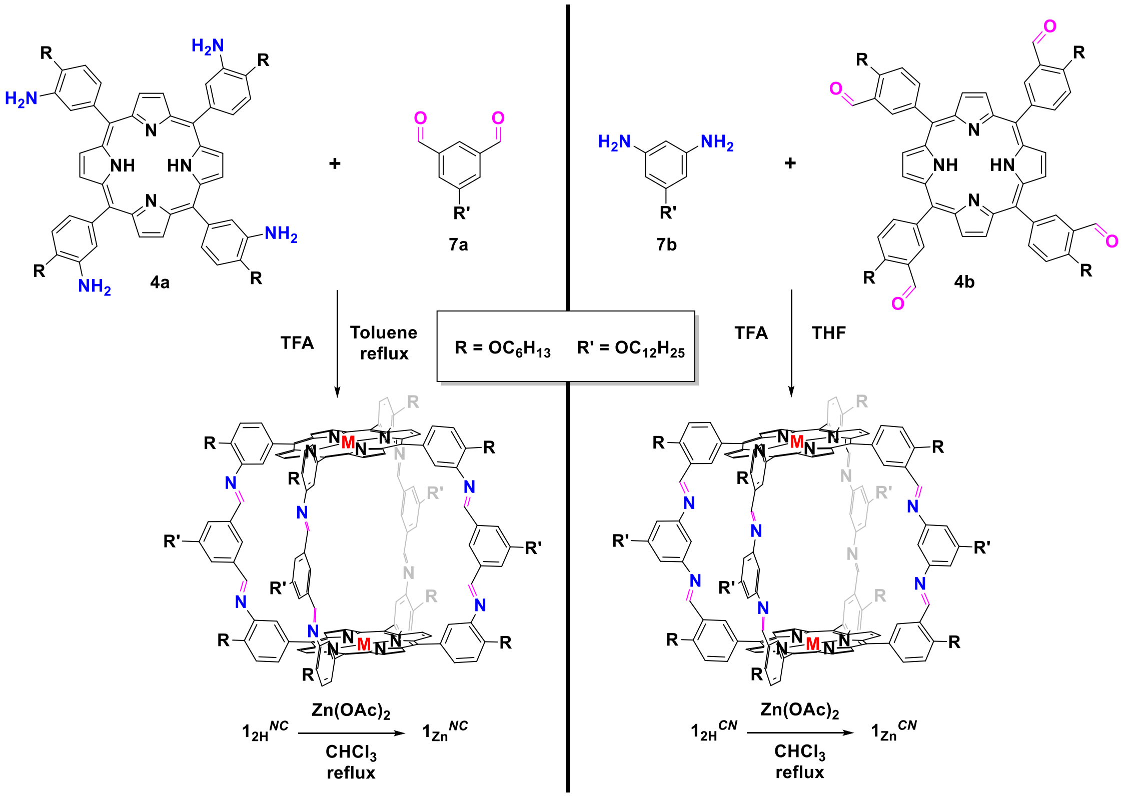

Scheme S3. Synthesis of molecular cages $\mathbf{1}_{Zn}{}^{NC}$ and $\mathbf{1}_{Zn}{}^{CN}$.

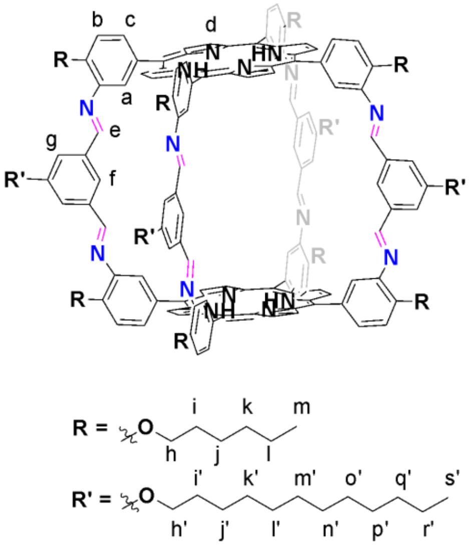

Molecular cage $1_{2H}{}^{NC}$. To a solution of porphyrin **4a** (105 mg, 0.098 mmol, 1 eq) and linker **7a** (65 mg, 0.205 mmol, 2.1 eq) in toluene (20 mL), TFA (0.098 mmol, 1 eq) was added under inert atmosphere. The mixture was heated at reflux, overnight. Then, MeOH was added helping to crash out a purple solid and the suspension was filtered and the solid rinsed with MeOH, obtaining the desired compound as a purple solid (160.0 mg, 99%).

^{1}H NMR (300 MHz, $CDCl_3$) δ (ppm) = 8.77 (s, 16H, H^{d}), 8.23 (s, 8H, H^{e}), 8.02 (dd, J = 7.9, 2.3 Hz, 8H, H^{c}), 7.86 (d, J = 1.3 Hz, 8H, H^{g}), 7.51 (d, J = 2.3 Hz, 8H, H^{a}), 7.27 (d, J = 7.9 Hz, 8H, H^{b}), 6.87 (t, J = 1.3 Hz, 4H, H^{f}), 4.31 (t, J = 6.5 Hz, 16H, H^{h}), 4.13 (t, J = 6.4 Hz, 8H, $H^{h'}$), 2.01 (p, J = 6.7 Hz, 16H, H^{i}), 1.85 (d, J = 7.4 Hz, 8H, $H^{i'}$), 1.70 – 1.22 (m, 120H, $H^{j\text{-}l+j'\text{-}r'}$), 1.01 – 0.87 (m, 36H, $H^{m+s'}$), –3.06 (s, 4H, **NH**).

^{13}C NMR (76 MHz, $CDCl_3$) δ (ppm) = 160.2, 159.9, 152.3, 139.5, 138.0, 135.1, 132.4, 131.2 (pyrr), 126.8, 125.8, 119.4, 115.5, 111.5, 77.4, 69.6, 68.7, 32.1, 31.9, 29.9, 29.8, 29.8, 29.7, 29.6, 29.6, 29.5, 26.2, 26.1, 22.9, 22.8, 14.3, 14.2.

HRMS (MALDI): Mass m/z calculated for $C_{216}H_{268}N_{16}O_{12}$ $[M]^{+}$ 3278.0847, found: 3278.0831.

FT-IR (ATR): ν (cm^{-1}) = 3314 (N-H), 2923, 2852, 1622 (C=N), 1588, 1496, 1467, 1292, 1244, 1125, 925, 799.

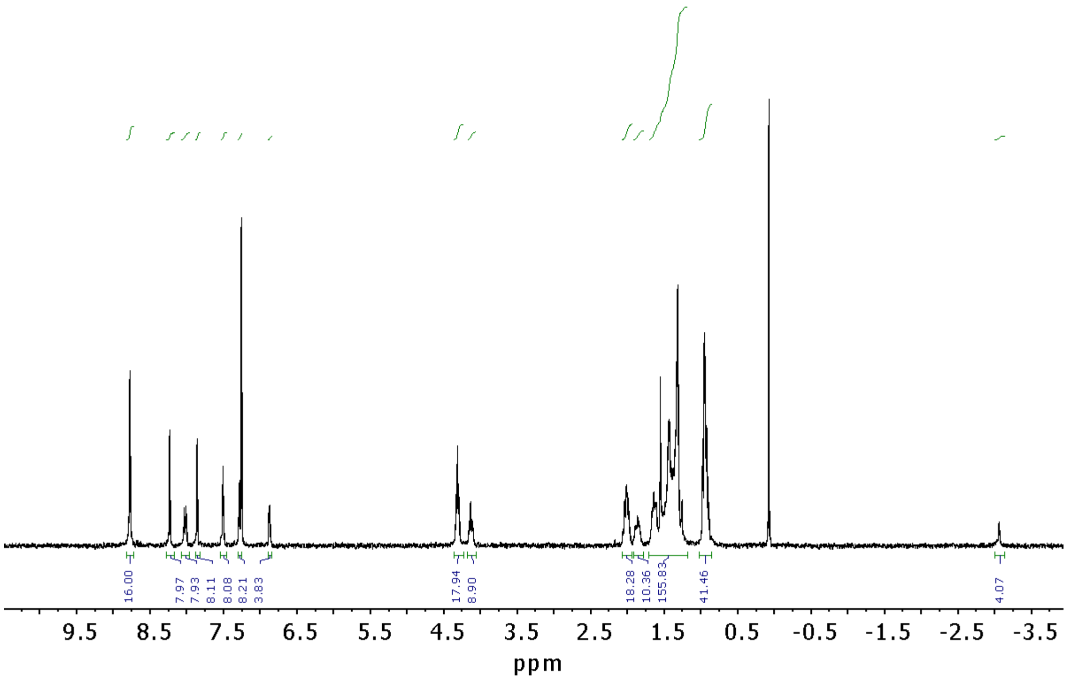

^{1}H NMR spectra ($CDCl_3$) of **1_{2H}**^{NC}.

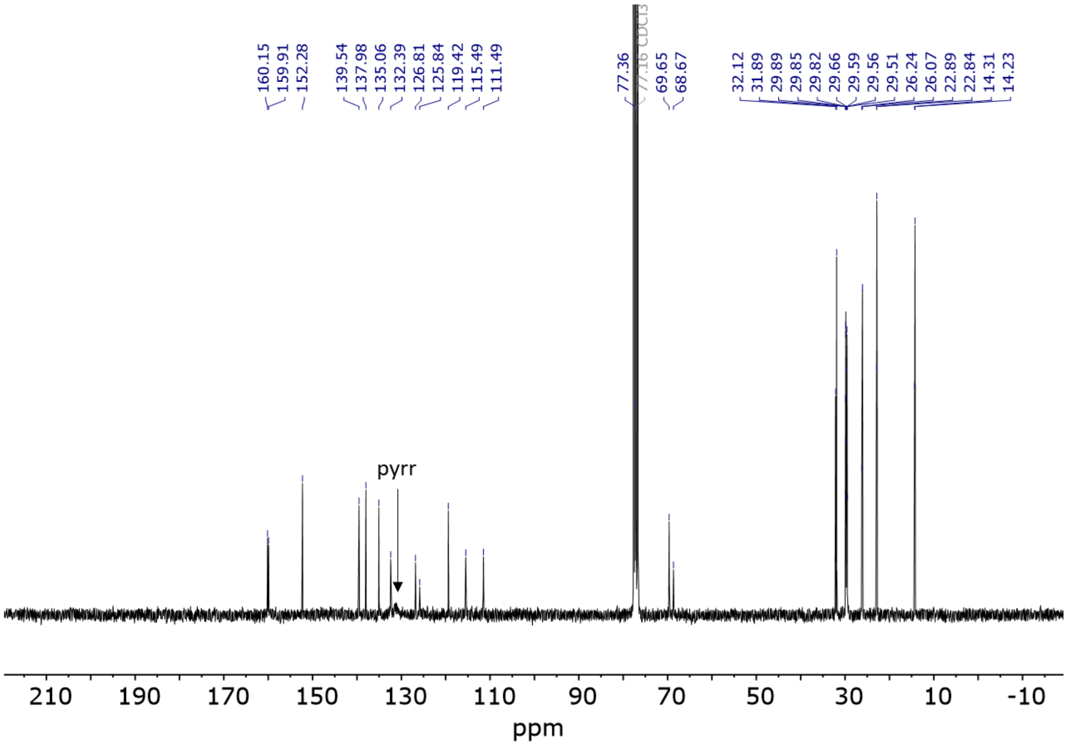

^{13}C NMR spectra ($CDCl_3$) of **1_{2H}**^{NC}.

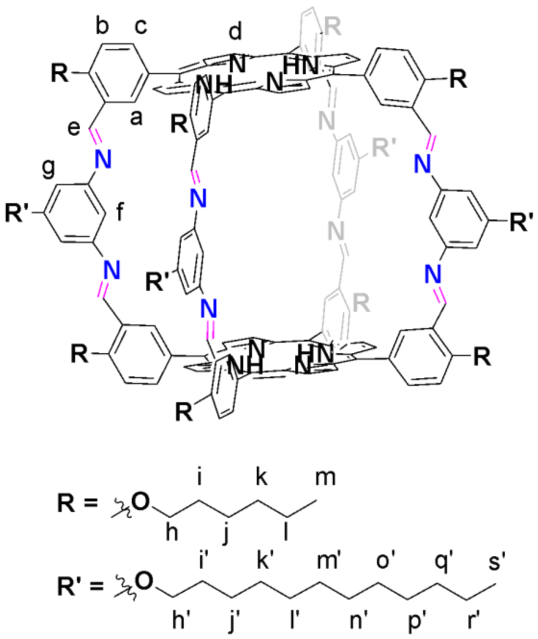

Molecular cage $\mathbf{1_{2H}}^{\mathit{CN}}$. To a solution of porphyrin **4b** (19.9 mg, 0.017 mmol, 1 eq) and linker **7b** (10.3 mg, 0.035 mmol, 2 eq) in THF (3.5 mL), TFA (0.0088 mmol, 1 eq) was added under inert atmosphere. The mixture was heated at room temperature 10 minutes and then, MeCN was added helping to crash out a purple solid and the suspension was filtered and the solid rinsed with MeCN, obtaining the desired compound cage $\mathbf{1_{2H}}^{\mathit{CN}}$ as a purple solid (19.7 mg, 66%).

^{1}H NMR (300 MHz, $CDCl_3$) *δ* (ppm) = 9.10 (s, 8H, H^e), 8.71 (s, 16H, H^d), 8.67 (d, *J* = 2.4 Hz, 8H, H^a), 8.23 (dd, *J* = 8.4, 2.4 Hz, 8H, H^b), 7.22 (d, *J* = 8.6 Hz, 8H, H^c), 6.62 (d, *J* = 1.9 Hz, 4H, H^g), 6.34 (t, *J* = 1.7 Hz, 8H, Hf), 4.30 (t, *J* = 6.6 Hz, 16H, H^h), 4.07 (t, *J* = 6.7 Hz, 8H, $H^{h´}$), 2.21 – 1.91 (m, 16H, H^i), 1.90 – 1.76 (m, 8H, $H^{i´}$), 1.67 – 1.13 (m, 120H, $H^{j\text{-}l+j´\text{-}r´}$), 0.97 (t, *J* = 6.95 Hz, 24H, H^m), 0.91 (t, *J* = 6.60 Hz, 12H, $H^{s´}$), –3.05 (s, 4H, **NH**).

HRMS (MALDI): Mass m/z calculated for $C_{216}H_{268}N_{16}O_{12}$ $[M]^+$ 3278.0847, found: 3278.0859.

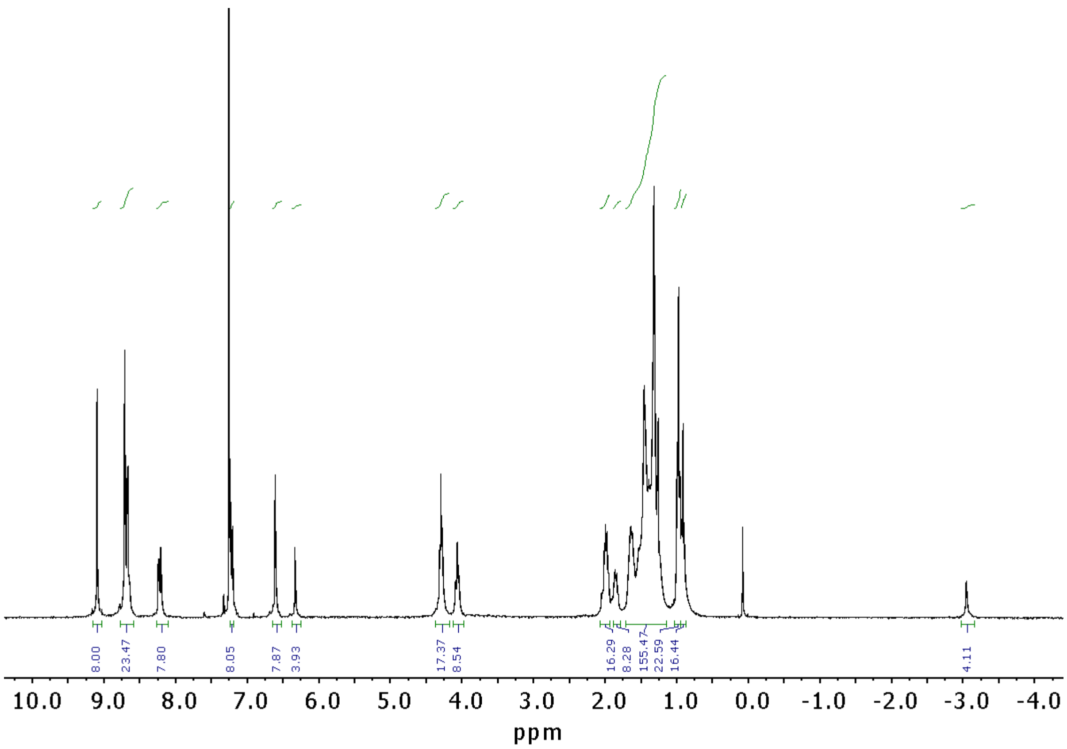

^{1}H NMR spectra ($CDCl_3$) of $\mathbf{1_{2H}}^{CN}$.

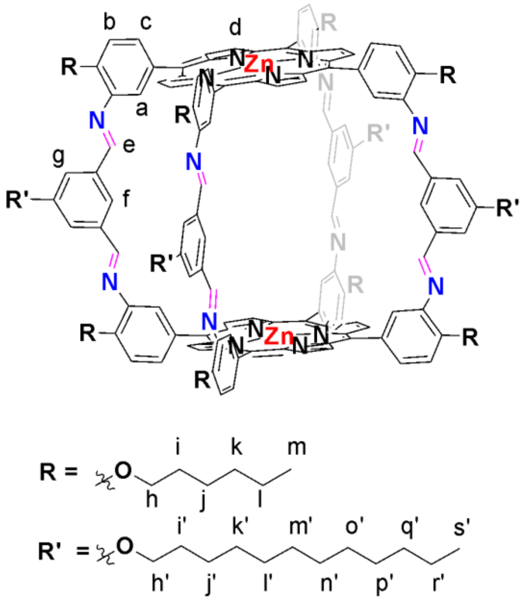

Molecular cage $\mathbf{1}_{\mathbf{Zn}}{}^{\boldsymbol{NC}}$. To a solution of cage $\mathbf{1}_{\mathbf{2H}}{}^{\boldsymbol{NC}}$ (160 mg, 0.049 mmol, 1 eq) in $CHCl_3$ (37 mL) $Zn(OAc)_2$ (20 mg, 0.107 mmol, 2.2 eq) was added and heated at reflux under inert atmosphere, overnight. Then, the solvent was removed under vacuum, re-dissolving the solid in the minimum volume of $CHCl_3$ or toluene to precipitate the molecular cage by the addition of MeCN. The solid was recovered by filtration and rinsed with MeCN and MeOH, achieving the final compound $\mathbf{1}_{\mathbf{Zn}}{}^{\boldsymbol{NC}}$ as a purple solid (166 mg, 99%).

^{1}H NMR (300 MHz, $CDCl_3$) δ (ppm) = 8.88 (s, 16H, H^{d}), 8.20 (s, 8H, H^{e}), 8.04 (dd, J = 8.2, 2.1 Hz, 8H, H^{c}), 7.88 (d, J = 1.4 Hz, 8H, H^{g}), 7.51 (d, J = 2.2 Hz, 8H, H^{c}), 7.27 (d, J = 8.2 Hz, 8H, H^{b}), 6.77 (t, J = 1.4 Hz, 4H, H^{f}), 4.32 (t, J = 6.5 Hz, 16H, H^{h}), 4.13 (t, J = 6.4 Hz, 8H, $H^{h'}$), 2.02 (p, J = 6.6 Hz, 16H, H^{i}), 1.93 – 1.79 (m, 8H, $H^{i'}$), 1.70 – 1.58 (m, 16H, H^{j}), 1.57 – 1.17 (m, 104H, $H^{k-l+j'-r'}$), 1.03 – 0.85 (m, 36H, $H^{m+s'}$).

^{13}C NMR (76 MHz, $CDCl_3$) δ (ppm) = 160.1, 159.5, 152.2, 150.5, 139.4, 137.98, 135.6, 132.2, 132.0, 131.2 (pyrr), 126.5, 125.9, 120.4, 115.4, 111.4, 77.4, 69.6, 68.6, 32.1, 31.9, 29.9, 29.8, 29.8, 29.6, 29.6, 29.6, 29.5, 26.2, 26.1, 22.9, 22.8, 14.3, 14.3, 14.2, 1.2.

HRMS (MALDI): Mass m/z calculated for: $C_{216}H_{264}N_{16}O_{12}Zn_2$ $[M]^+$ 3401.9117, found: 3401.9153.

FT-IR (ATR): ν (cm^{-1}) = 2922, 2852, 1622 (C=N), 1588, 1491, 1467, 1247, 1126, 1003, 941, 798, 722.

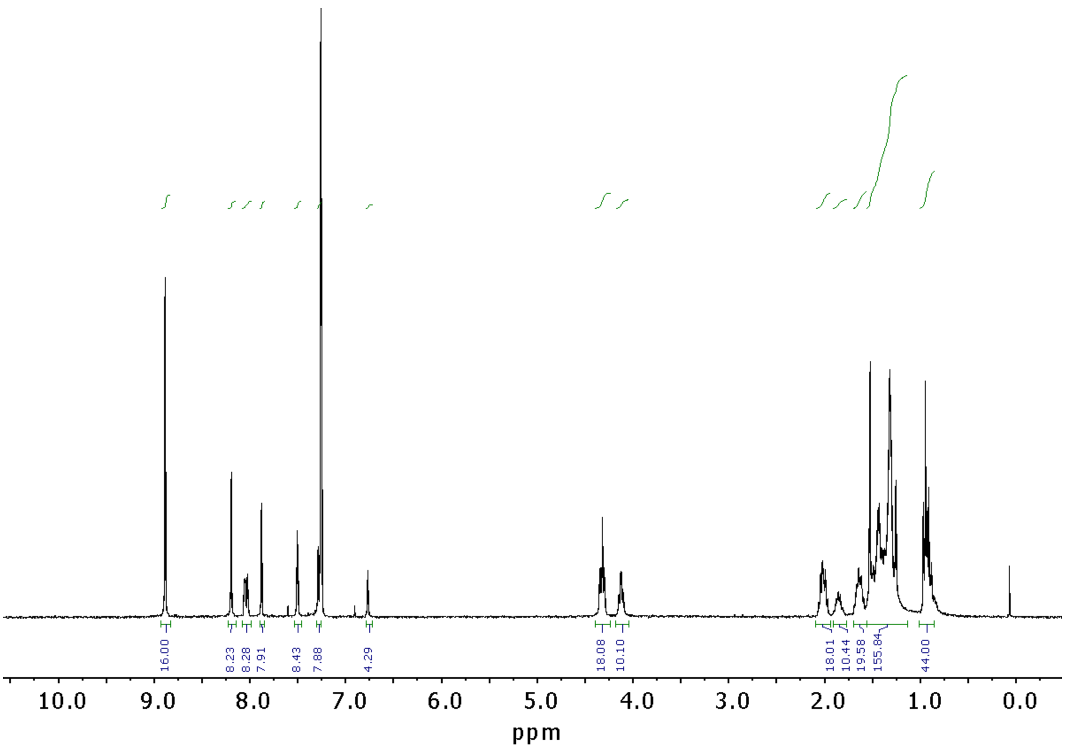

^{1}H NMR spectra (CDCl$_3$) of $\mathbf{1}_{Zn}^{NC}$.

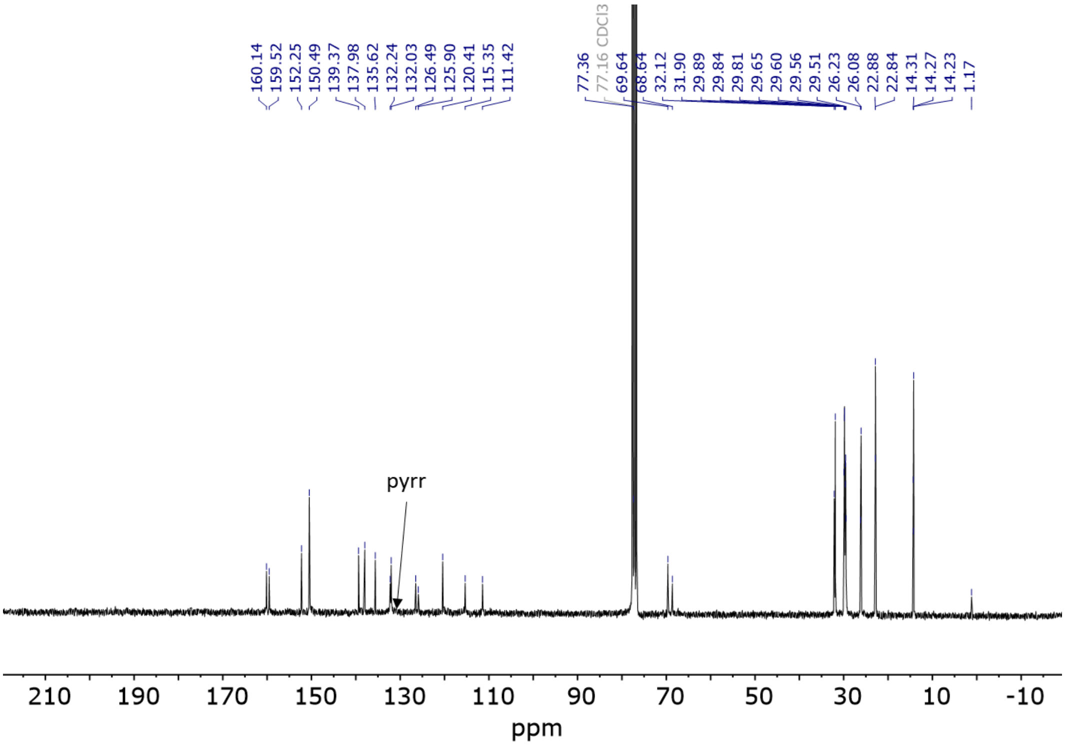

^{13}C NMR spectra (CDCl$_3$) of $\mathbf{1}_{Zn}^{NC}$.

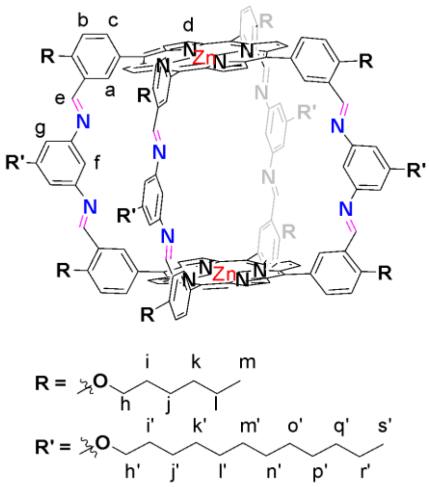

Method A. Molecular cage $\mathbf{1}_{\mathbf{Zn}}{}^{CN}$. To a solution of cage $\mathbf{1}_{\mathbf{2H}}{}^{CN}$ (110 mg, 0.033 mmol, 1 eq) in $CHCl_3$ (60 mL) $Zn(OAc)_2$ (18 mg, 0.1 mmol, 3 eq) was added and heated at reflux under inert atmosphere, overnight. Then, the solvent was removed under vacuum, re-dissolving the solid in the minimum volume of $CHCl_3$ to precipitate the molecular cage by the addition of MeCN. The solid was recovered by filtration and rinsed with MeCN and MeOH, achieving the final compound $\mathbf{1}_{\mathbf{Zn}}{}^{NC}$ as a purple solid (113 mg, 98%).

Method B. Molecular cage $\mathbf{1}_{\mathbf{Zn}}{}^{CN}$. To a solution of porphyrin **4b** (82.4 mg, 0.0731 mmol, 1 eq) and linker **7b** (42.7 mg, 0.1461 mmol, 2 eq) in THF (12.6 mL), TFA (0.0365 mmol, 0.5 eq) was added under inert atmosphere. The mixture was stirred at room temperature 10 minutes and TEA (0.0731 mmol, 1 eq) was subsequently added and the reaction stirred 10 minutes more. After that, MeCN was added to trigger the emergence of purple solid suspension. The solid was filtered and rinsed with MeCN. Finally, the solid compound was solubilized with $CHCl_3$ (30 mL), and $Zn(OAc)_2$ (53.6 mg, 0.2920 mmol, 4.0 eq) was added and heated at reflux under inert atmosphere, overnight. Then, the solvent was removed under vacuum, re-dissolving the solid in the minimum volume of $CHCl_3$ to precipitate the molecular cage by the addition of MeCN. The solid was recovered by filtration and rinsed with MeCN and MeOH, the final compound $\mathbf{1}_{\mathbf{Zn}}{}^{CN}$ as a purple solid (88.2 mg, 71%, over two steps).

^{1}H NMR (300 MHz, $CDCl_3$) δ (ppm) = 9.11 (s, 8H, H^{e}), 8.82 (s, 16H, H^{d}), 8.66 (d, J = 2.5 Hz, 8H, H^{a}), 8.24 (dd, J = 8.3, 2.5 Hz, 8H, H^{c}), 7.23 (d, J = 8.4 Hz, 8H, H^{b}), 6.61 (d, J = 1.9 Hz, 8H, H^{g}), 6.31 (d, J = 1.8 Hz, 4H, H^{f}), 4.31 (t, J = 6.3 Hz, 16H, H^{h}), 4.07 (t, J = 6.5 Hz, 8H, $H^{h'}$), 2.08 – 1.92 (m, 16H, H^{i}), 1.91 – 1.83 (m, 8H, $H^{i'}$), 1.75 – 1.10 (m, 120H, $H^{j\text{-}l+j'\text{-}r'}$), 1.03 – 0.86 (m, 36efH, $H^{m+s'}$).

^{13}C NMR (75 MHz, $CDCl_3$) δ (ppm) = 160.4, 158.6, 155.0, 153.4, 150.6, 136.5, 135.5, 133.8, 132.1, 123.2, 120.3, 115.6, 110.0, 101.1, 77.4, 68.9, 68.4, 32.1, 31.8, 29.9, 29.8, 29.7, 29.6, 29.6, 29.5, 26.4, 26.1, 22.9, 22.8, 14.3, 14.3.

HRMS (MALDI): Mass m/z calculated for: $C_{216}H_{264}N_{16}O_{12}Zn_2$ $[M]^+$ 3401.9117, found: 3401.9185.

FT-IR (ATR): ν (cm^{-1}) = 2922, 2852, 1623 (C=N), 1576, 1488, 1467, 1366, 1337, 1250, 1116, 1002, 798.

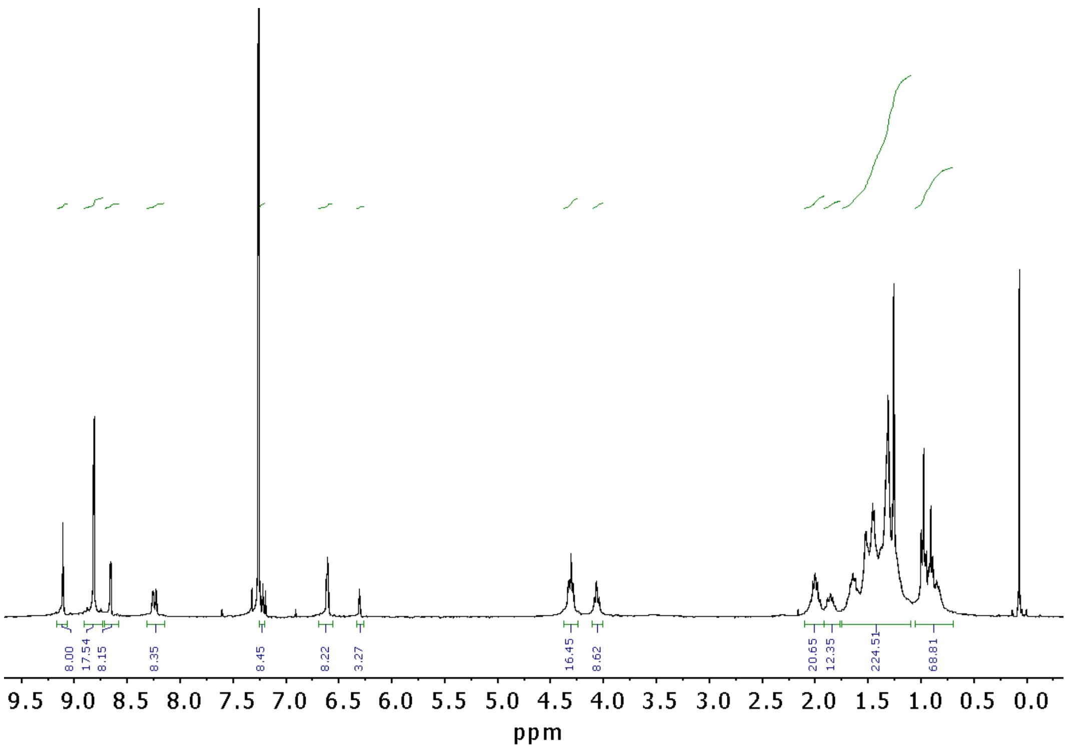

^{1}H NMR spectra (CDCl3) of $\mathbf{1}_{Zn}^{CN}$.

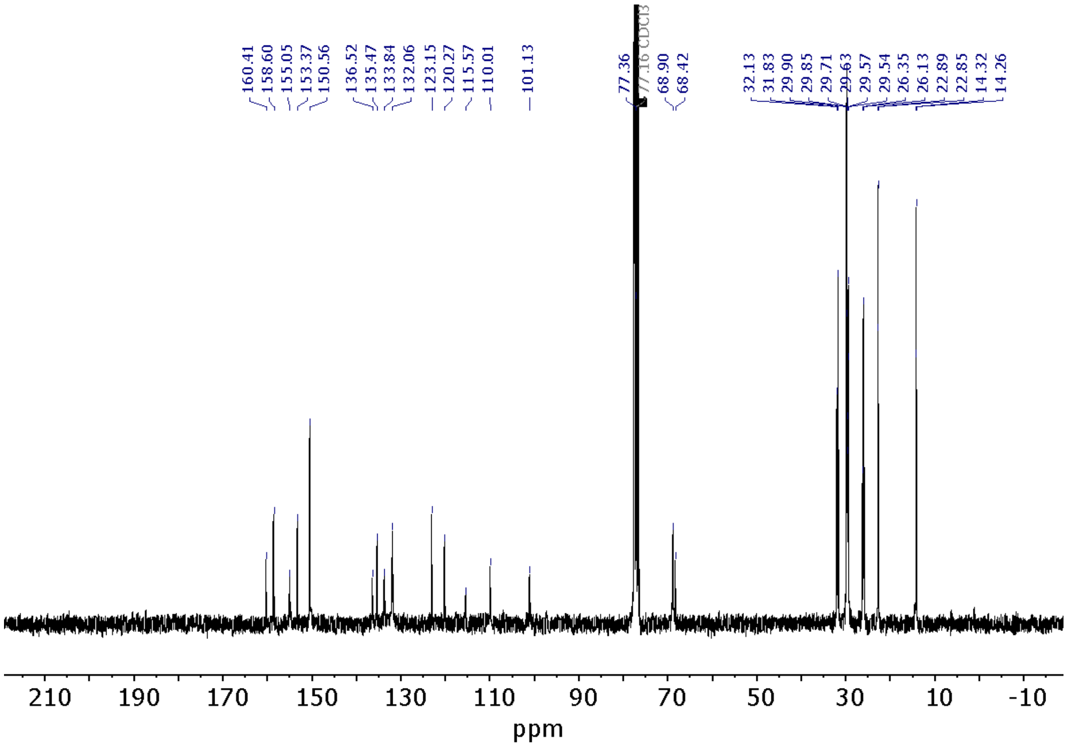

^{13}C NMR spectra (CDCl3) of $\mathbf{1}_{Zn}^{CN}$.

Synthesis of reduced cages 2_{Zn}^{NC}.

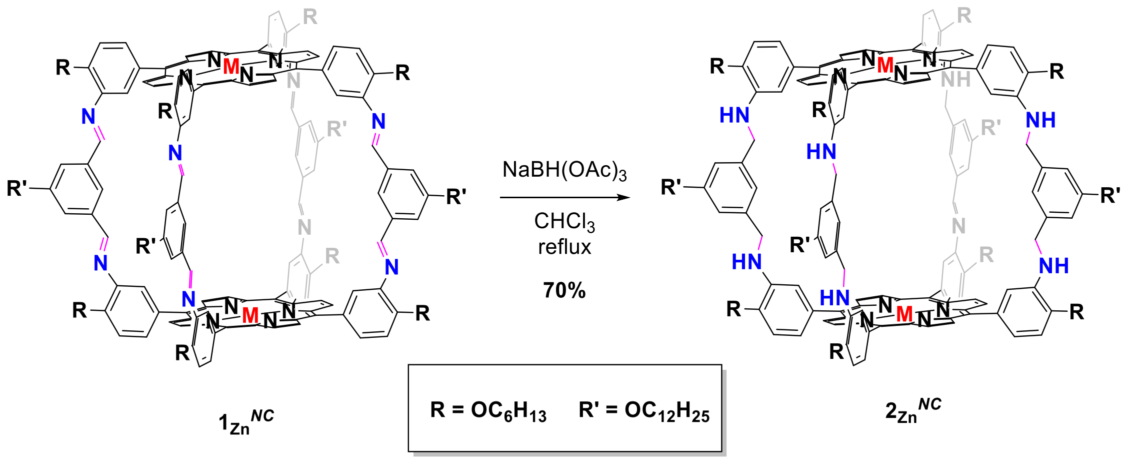

Scheme S4. Synthesis of molecular cages $\mathbf{2}_{Zn}^{NC}$.

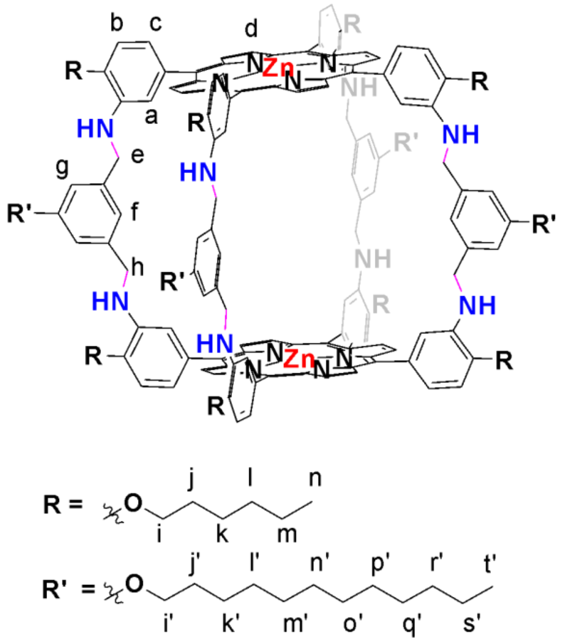

Molecular cage $\mathbf{2}_{Zn}^{NC}$. To a solution of cage $\mathbf{1}_{Zn}^{NC}$ (44.0 mg, 0.013 mmol, 1 eq) in $CHCl_3$ (3.6 mL), $NaBH(OAc)_3$ (87.2 mg, 0.41 mmol, 32 eq) was added and heated at reflux under inert atmosphere, 90 min. Then, the product was precipitated by the addition of MeOH and the solid was recovered by filtration and rinsed with MeOH, achieving the final compound $\mathbf{2}_{Zn}^{NC}$ as a purple solid (31.0 mg, 70%).

^{1}H NMR (500 MHz, $CDCl_3$) δ (ppm) = 8.68 (s, 16H, H^d), 7.31 (dd, J = 7.8, 2.0 Hz, 8H, H^c), 7.06 (d, J = 2.0 Hz, 8H, H^a), 7.00 (d, J = 8.0 Hz, 8H, H^b), 6.90 (bs, 8H, H^g), 6.51 (bs, 4H, H^f), 4.77 (bs, 8H, **NH**), 4.24 (t, J = 6.6 Hz, 16H, H^i), 4.09 (bs, 16H, H^h), 3.96 (t, J = 6.5 Hz, 8H, $H^{i'}$), 2.02 – 1.93 (m, 16H, H^j), 1.84 – 1.77 (m, 8H, $H^{j'}$), 1.64 –1.56 (m, 16H, H^k), 1.52 – 1.18 (m, 104H, $H^{l\text{-}m,k'\text{-}s'}$), 0.97 (t, J = 6.9 Hz, 24H, $H^{t'}$), 0.87 (t, J = 6.74 Hz, 12H, H^n).

^{13}C NMR (126 MHz, $CDCl_3$) δ (ppm) = 160.2, 150.3, 145.8, 141.1, 135.9, 135.6, 131.5, 123.6, 121.2, 119.2, 117.6, 111.9, 108.2, 68.7, 68.2, 48.1, 32.1, 32.1, 31.9, 29.9, 29.9, 29.8, 29.8, 29.7, 29.7, 29.57, 26.4, 26.2, 22.7, 14.3, 14.3.

HRMS (MALDI): Mass m/z calculated for: $C_{216}H_{280}N_{16}O_{12}Zn_2$ $[M]^+$ 3418.0369, found: 3418.0085.

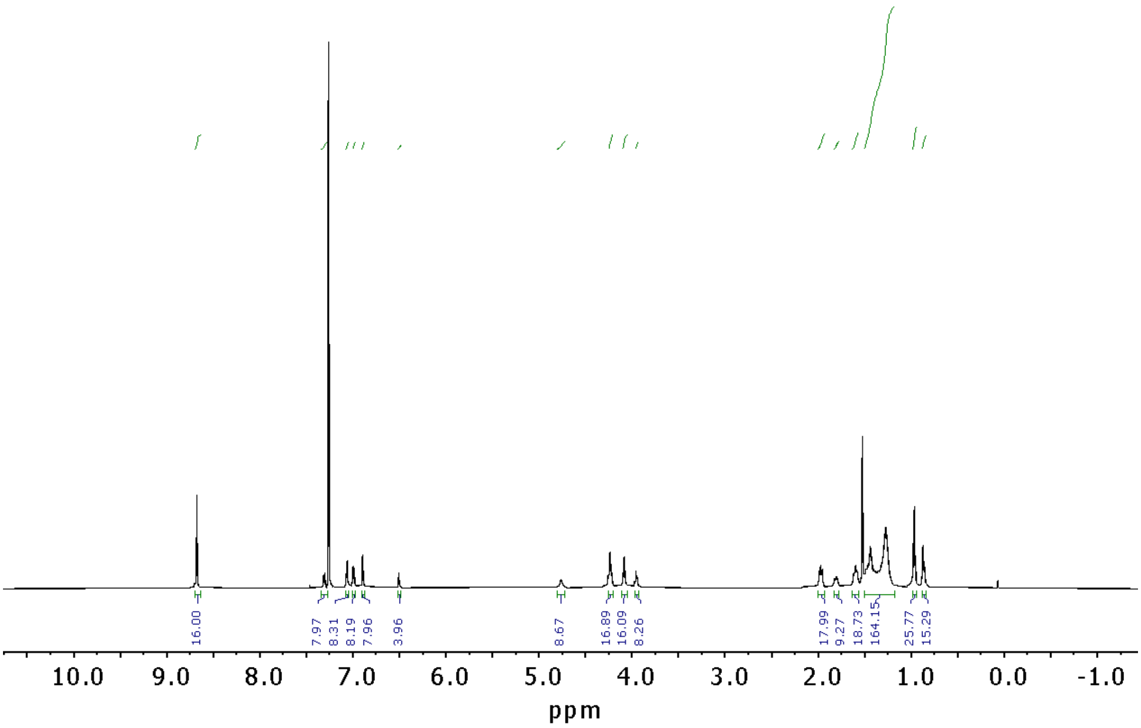

^{1}H NMR spectrum ($CDCl_3$) of **2**$_{Zn}$^{NC}.

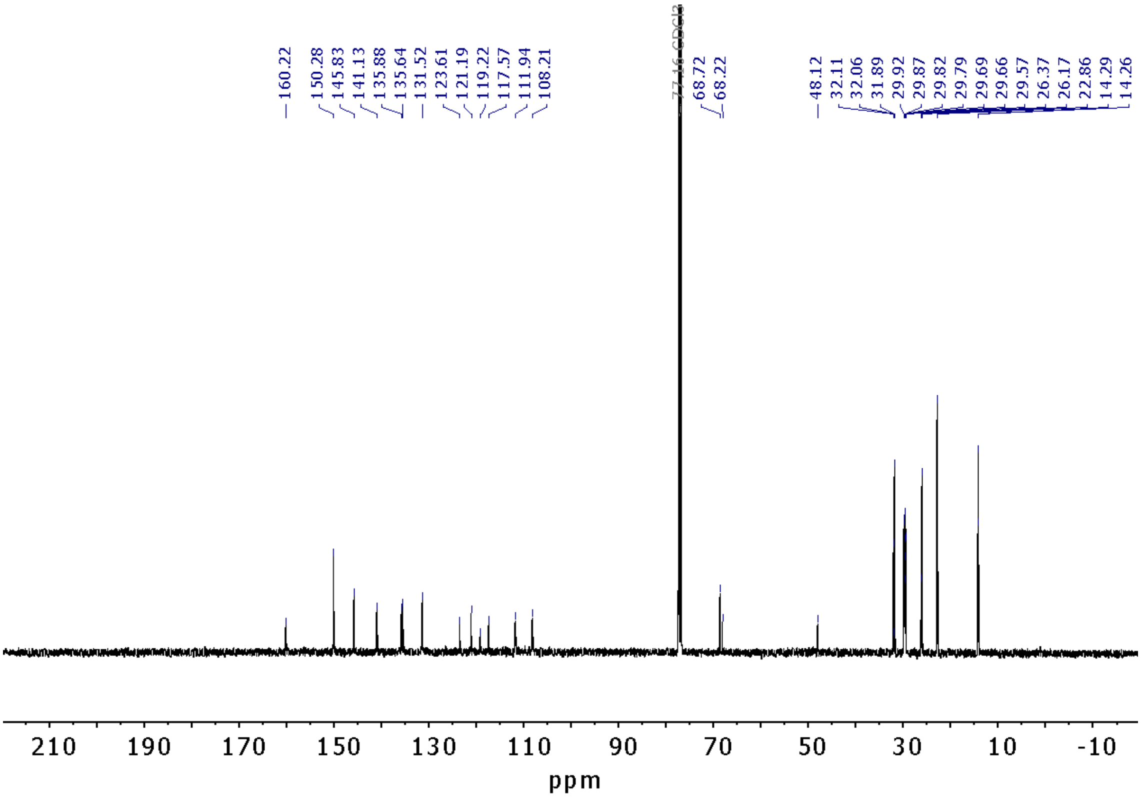

^{13}C NMR spectrum ($CDCl_3$) of **2**$_{Zn}$^{NC}.

NMR titration experiments.

To determine the association constants between the reference porphyrin $\mathbf{P_{Zn}}$ with pyridine or benzylamine, the experiments were conducted at a constant concentration of the host molecule (porphyrin). Briefly, to a solution of $\mathbf{P_{Zn}}$ (5.0 10^{-4} M) in the corresponding solvent, increasing amounts of a solution containing $\mathbf{P_{ref}}$ (5.0 10^{-4} M) with a large excess of the guest molecule (20-50 equivalents) were added. The data obtained was then fitted to 1:1 equilibrium model using the free software from www.supramolecular.org, as well as the software *equilibria.exe*. In this procedure, the chemical shift of diverse ^{1}H NMR signals of $\mathbf{P_{Zn}}$ was employed to calculate the association constant value.

For $\mathbf{1_{Zn}}^{NC}$, $\mathbf{1_{Zn}}^{CN}$ or $\mathbf{2_{Zn}}^{NC}$ qualitative experiments were preliminarily carried out to inspect the supramolecular behaviour of these molecular cages in the presence of increasing amounts of different guest molecules, that is if they exhibited fast or slow exchange in the NMR timescale, which is an indication of cooperative binding to both metal centers within the cavity.

In all experiments conducted in $CDCl_3$, this solvent was previously neutralized with K_2CO_3 to avoid a possible cage degradation or imine exchange.

UV-Vis and fluorescence titration experiments.

To determine the association constant between the different molecular cages and the di-nitrogenated guest molecules, all the experiments were conducted at a constant concentration of the host molecule (cage). Depending on the association constant expected for a specific host-guest combination, the titration experiment was done at higher or lower concentrations, monitoring the process by either UV-vis spectroscopy, in the high concentration range (10^{-5}–10^{-7}), or by fluorescence spectroscopy, in the low concentration regime (down to 10^{-9} M). Experimentally, to a solution of $\mathbf{1_{Zn}}^{NC}$, $\mathbf{1_{Zn}}^{CN}$ or $\mathbf{2_{Zn}}^{NC}$ (10^{-5}–10^{-9} M) in the corresponding solvent, increasing amounts of a solution containing the same cage at the same concentration (10^{-5}–10^{-9} M) and a large excess of the guest molecule (20–50 equivalents) were added. The data obtained was then fitted to a 1:1 equilibrium model, in view of the results previously obtained by NMR, using the *Reactlab Equilibria* software, which calculates the association constants through data fitting from the whole spectra.

X-ray Crystal Structure Analyses.

In both structures, Z' (the number of molecules in the asymmetric unit) is 0.5, as there is an inversion centre in the middle of the molecule. As a consequence, the number of molecules in the unit cell (Z) is 2 instead of 1.

The presence of very long solubilizing alkyl chains in these structures, which was useful for solution experiments, difficulted the crystal´s diffraction as well as the optimization of the structures achieved. The solubilizing chains presented a great degree of disorder that increased as the distance from the rigid cage core increased. Due to this reason, it was necessary to apply numerous geometrical restrictions (in the side chains) to reach model convergence.

Unfortunately, this disorder was not possible to model through alternative positions, thus only the position with higher electronic density was included.

Cavity size of molecular cages.

The inner void space of the cages (**1**$_{Zn}^{NC}$ and **1**$_{Zn}^{CN}$) in both extended and compact conformation was calculated using CageCavityCalc (*C*$_3$), a Python based computational tool developed by Martí-Centelles et al.[3] *C*$_3$ employs an algorithm that allows the calculation of the cavity size upon loading a .pdb file of the corresponding geometry optimized cage. The void space was calculated using different grid parameters, founding the best results for grid 0.3 in all the cases.

S1. Characterization of 1_{Zn}^{NC}

^{1}H NMR

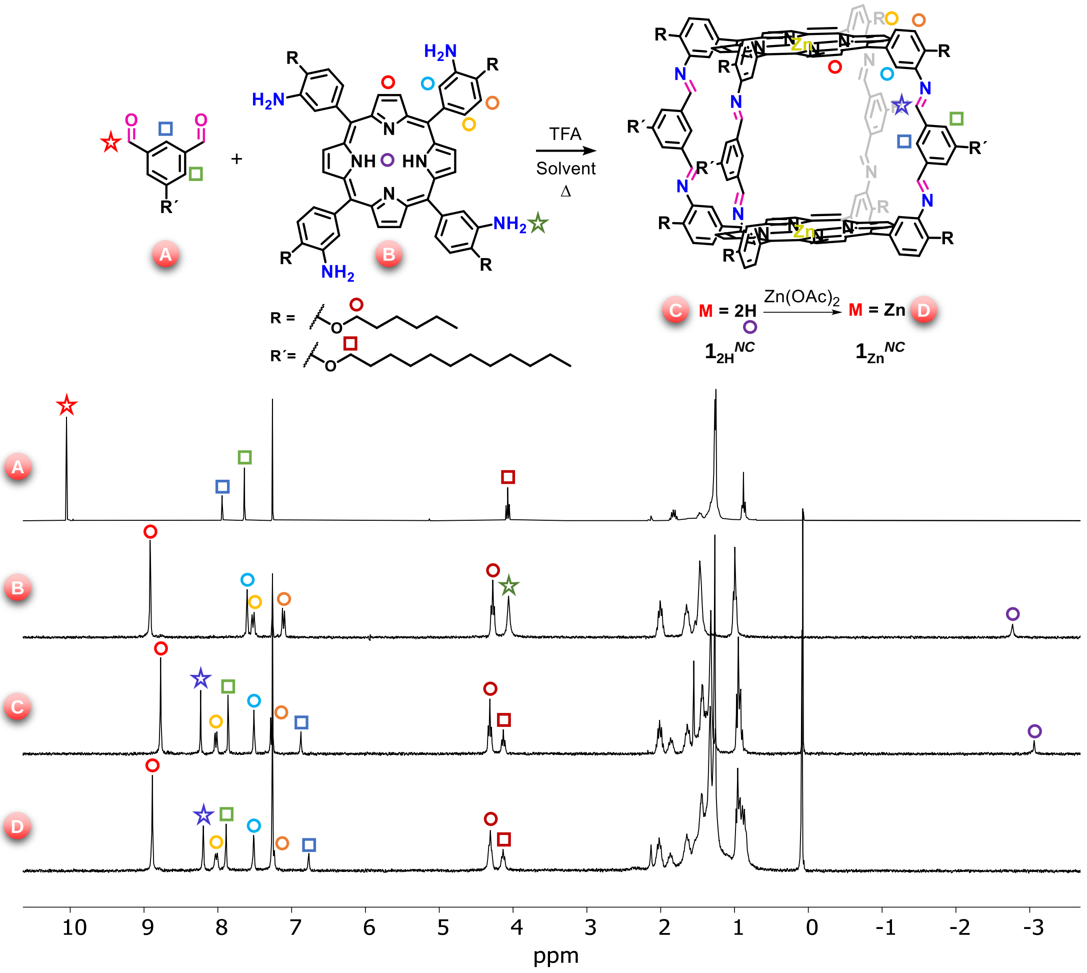

Figure S1A. ^{1}H NMR spectra of 1_{Zn}^{NC}, 1_{2H}^{NC}, as well as of the corresponding tetraaminoporphyrin (**4a**) and dialdehyde (**7a**) precursors in $CDCl_3$ at 298 K.

Absorption and Emission

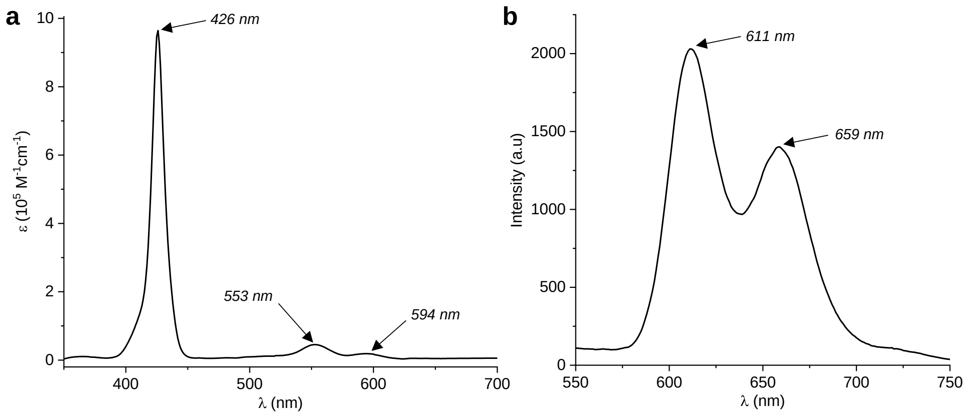

Figure S1B. (**a**) UV-vis absorption and (**b**) fluorescence emission spectra of 1_{Zn}^{NC} in $CHCl_3$ at 298 K.

NOESY Experiments

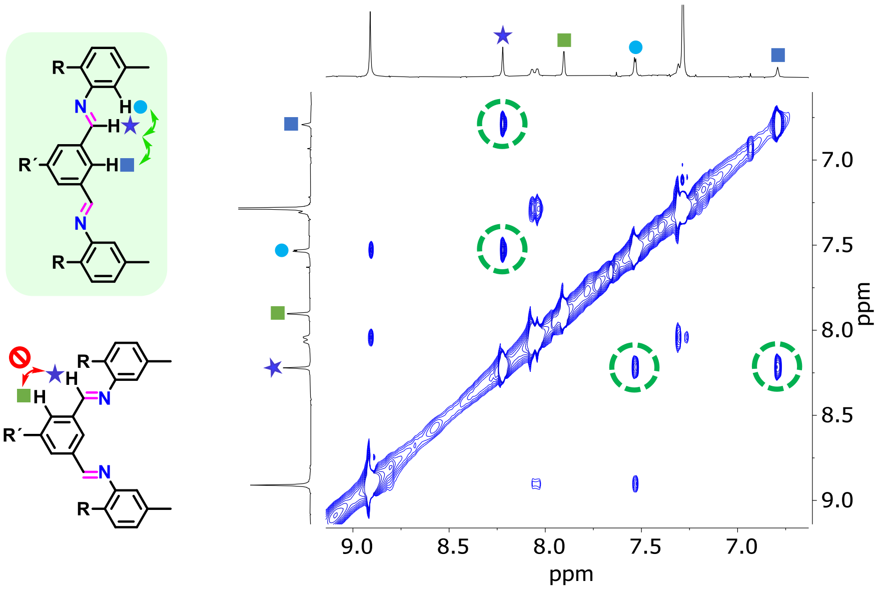

Figure S1C. 2D NOESY spectrum of the $\mathbf{1}_{Zn}^{NC}$ in $CDCl_3$ at 298 K, showing NOE cross-peaks that support the prevalence of an *extended* conformation. Cross-peaks between protons that could suggest the coexistence of a *compact* conformation were not observed.

Mass spectrometry

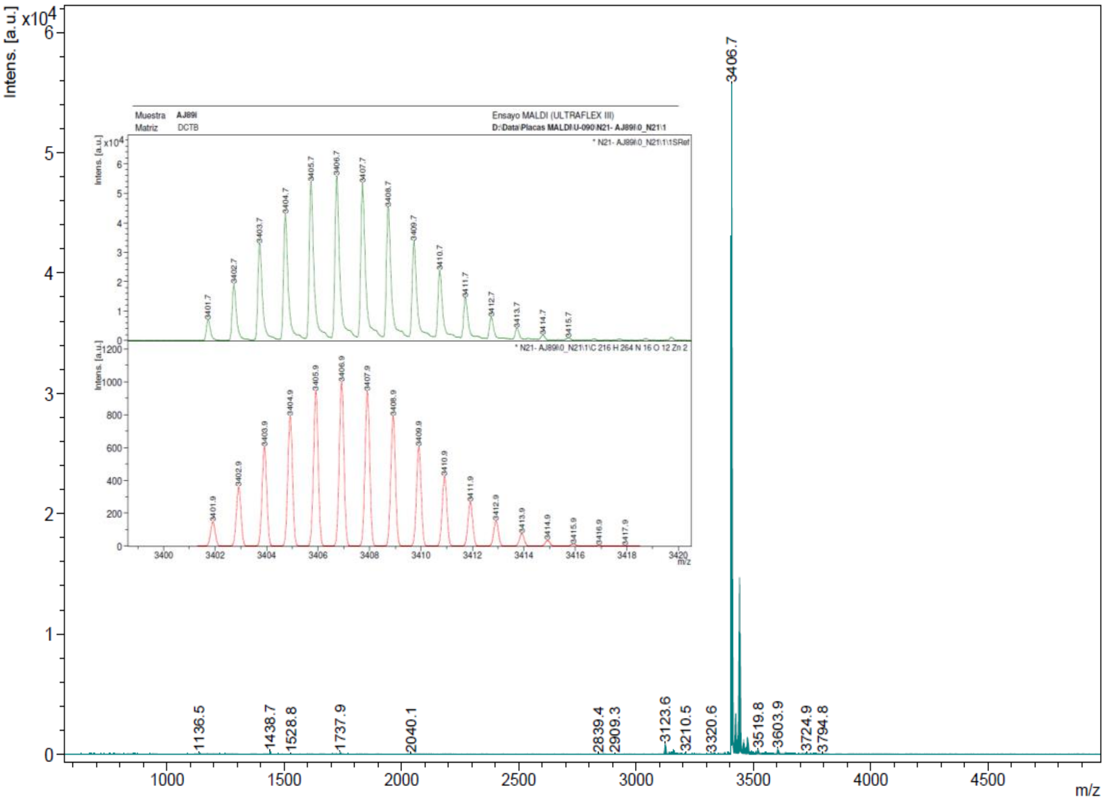

Figure S1D. MS (MALDI) spectra of $\mathbf{1}_{Zn}^{NC}$, showing the comparison of the experimental and theoretical isotopic distribution of the parent *ion* $[M]^+$

Solid state crystal structure of $\mathbf{1}_{Zn}^{NC}$

Single crystals of $\mathbf{1}_{Zn}^{NC}$ were obtained by a slow cooling process from 140 to 25 ºC of a saturated solution of $\mathbf{1}_{Zn}^{NC}$ in dimethylformamide (DMF). The crystals obtained are dark red and have the macroscopic shape of flat hexagonal prisms.

Molecular cage $\mathbf{1}_{Zn}^{NC}$ crystallizes in the monoclinic $P2_1/c$ space group with an asymmetric unit containing one porphyrin unit and two linkers. The crystal structure obtained incorporates DMF molecules coordinated to the Zn atoms through the inner face of the cage (hidden for clarity) as well as interstitial molecules of water and DMF in both the inner and outer areas of the cavity.

The crystal structure of $\mathbf{1}_{Zn}^{NC}$ presented the so-called *extended* conformation, attending to the arrangement of the imine bonds presented in the four linkers, which was the same than the conformation determined in solution NOESY experiments. Interestingly, although the porphyrin used to prepare the molecular cage was D_{4h}-symmetric, the molecular cage achieved presented a rectangular shape and thus a lower (pseudo-D_{2h}) symmetry in the solid state. This symmetry reduction was due to the presence of two kinds of gates or pores. The first type of gate featured a square shape (see Figure S1E, front view) formed by the edge of two porphyrin units separated by 12.77 Å and two linkers by 11.02 Å (area *ca*. 140 Å^2). The second type has a rectangular shape, showing two different sides of 12.14 Å and 8.97 Å (see Figure S1E, side view), forming a pore of *ca*. 108 Å^2.

The Zn···Zn distance measured for the empty cage was 12.02 Å, slightly shorter than the distance between porphyrins, because the metal atoms were a slightly displaced out of the porphyrin ring´s plane. The torsional angles formed by the aryl substituents at the *meso* positions, and the porphyrin plane oscillated between 65º and 70º. A ~42-47º deviation of the imines from a planar conformation with respect to the aromatic rings at the *meso* position is also observed in the crystal structures. Additionally, the −C=N− bond length of the imine groups was measured as 1.26-1.28 Å, while the −C=N−C angles oscillate between 117.2º and 119.3º.

The packing of molecular cage $\mathbf{1}_{Zn}^{NC}$ in the unit cell resulted in a porous 3D structure where the solubilizing alkyl chains were placed in the inter-space between cages, helping to “glue” the structure together. Furthermore, porphyrin cages were stacked forming a columnar structure where the porphyrin lids were tilted *ca*. 55º between successive floors (see Figure S1E).

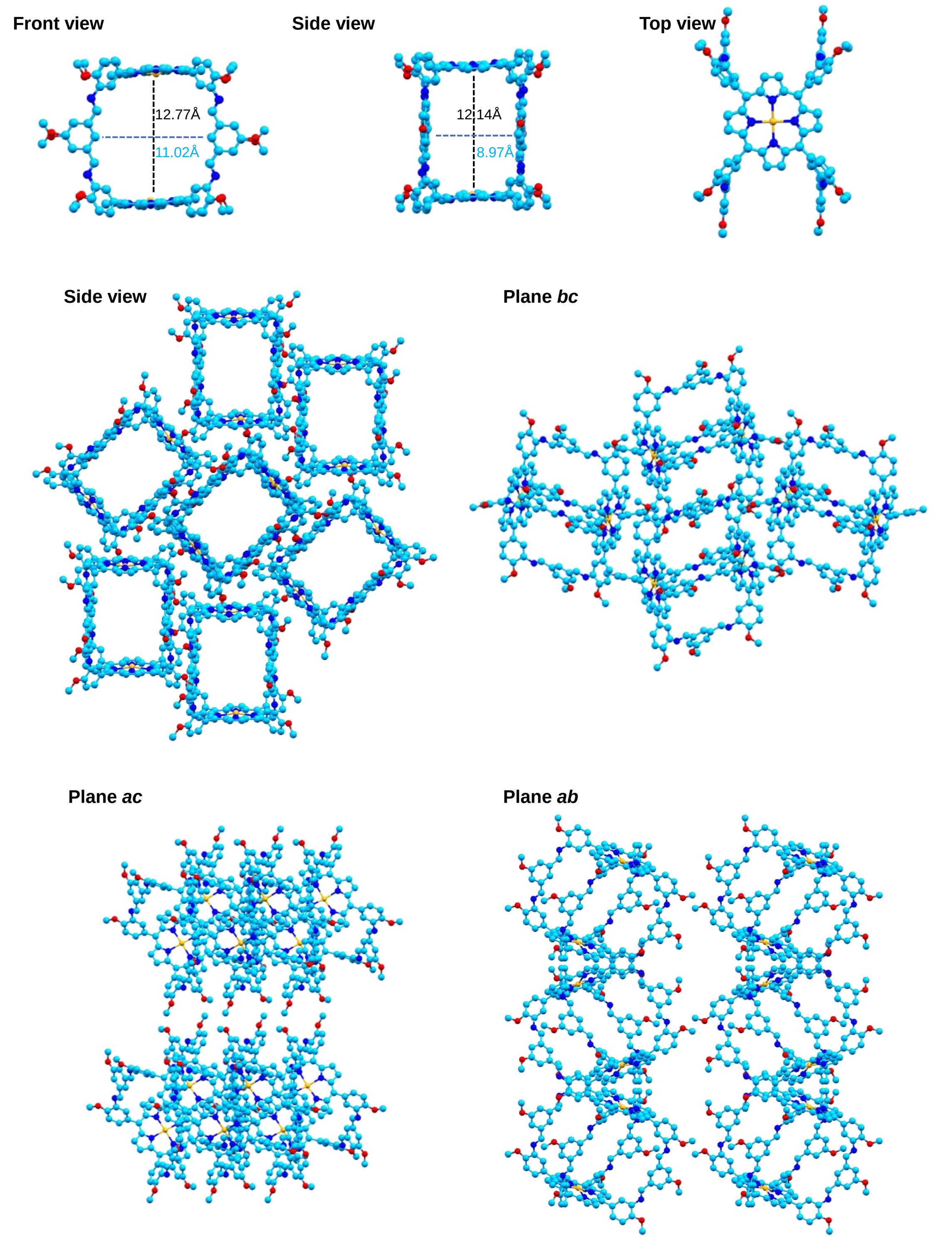

Figure S1E. Crystal structure and packing of $\mathbf{1}_{Zn}^{NC}$. The solubilizing alkyl chains and hydrogen atoms were omitted for clarity. DMF molecules coordinated to Zn atoms and residual solvent are also omitted. The top images show selected front, side and top views of the cage and the packing arrangement. The bottom images show the corresponding packing as collected from side view and planes *bc*, *ac*, and *ab* of the unit cell.

Table S1. Crystallographic data of 1_{Zn}^{NC}

CCDC number	2375303
Empirical formula*	$C_{191.5}H_{204}N_{18}O_{14}Zn_2$,3.5 ($C_3H_7NO$),1.5 (O)
Formula weight	3397.83
Temperature [K]	150.01(10)
Crystal system	monoclinic
Space group	$P2_1/c$
a [Å]	23.6692(2)
b [Å]	30.9935(3)
c [Å]	14.88040(10)
α [°]	90
β [°]	95.0810(10)
γ [°]	90
Volume [Å^3]	10873.24(16)
Z	2
ρ_{calc} [g/cm^3]	1.038
μ [mm^{-1}]	0.743
λ	1.54184 (Cu Kα)
2Θ range for data collection/°	4.71 to 136.494
Data/restraints/parameters	19823/1049/1234
Goodness-of-fit on F^2	1.045
R [I>=2σ (I)]	0.0914
wR_2	0.2932

*The empirical formula was deduced from the atoms that could be modelled from the crystal structure.

Cavity size of 1_{Zn}^{NC}

Extended conformation

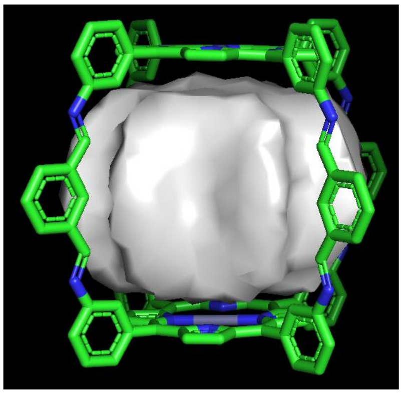

Volume of the cavity= **827.58 A^3**

Compact conformation

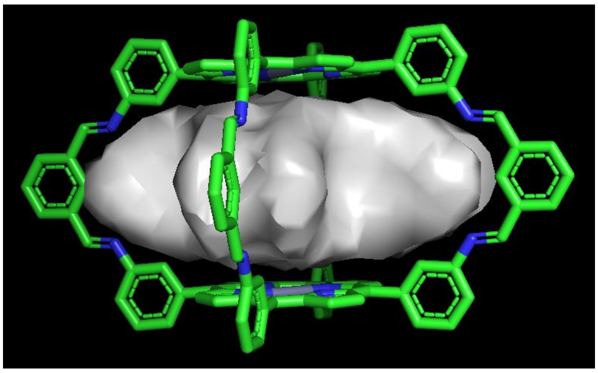

Volume of the cavity= **650.49 A^3**

Figure S1F. Computed cavity size of the cage $\mathbf{1}_{Zn}^{NC}$ in both the extended and compact conformations. Carbon atoms are coloured in green, nitrogen atoms in blue and zinc atoms in grey.

S2. Characterization of 1_{Zn}^{CN}

^{1}H NMR

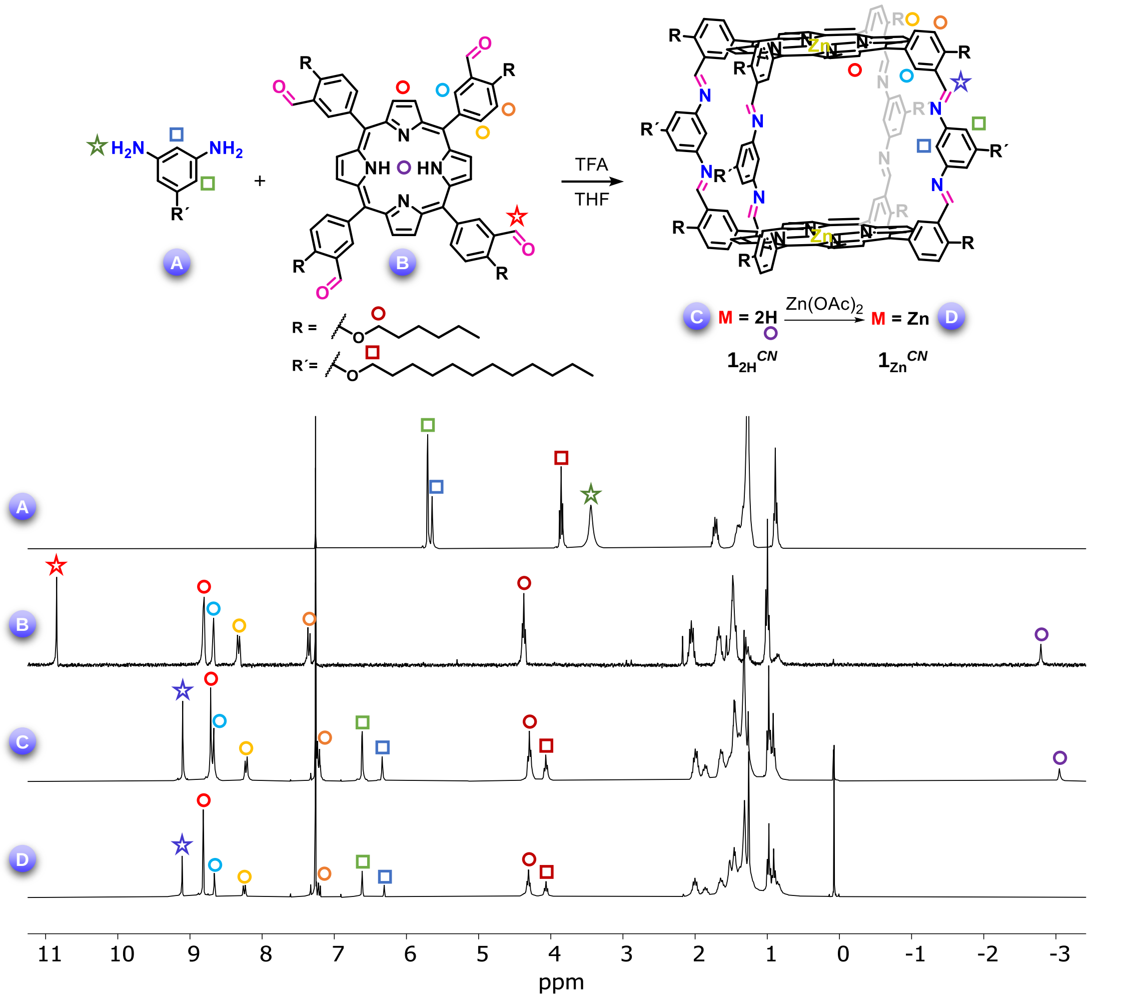

Figure S2A. ^{1}H NMR spectra of $\mathbf{1_{Zn}}^{CN}$, $\mathbf{1_{2H}}^{CN}$, as well as the corresponding tetraformylporphyrin (**4b**) and dialdehyde (**7b**) precursors in $CDCl_3$ at 298 K.

Absorption and Emission

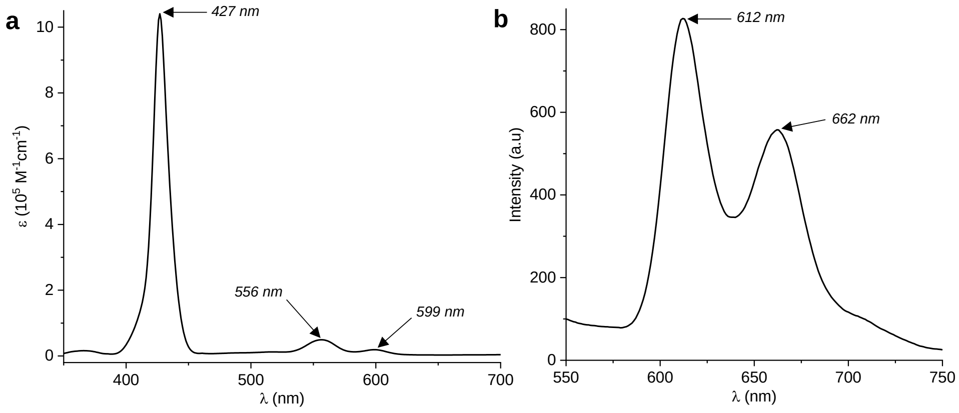

Figure S2B. (**a**) UV-vis absorption and (**b**) fluorescence emission spectra of $\mathbf{1_{Zn}}^{CN}$ in $CHCl_3$ at 298 K.

NOESY Experiments

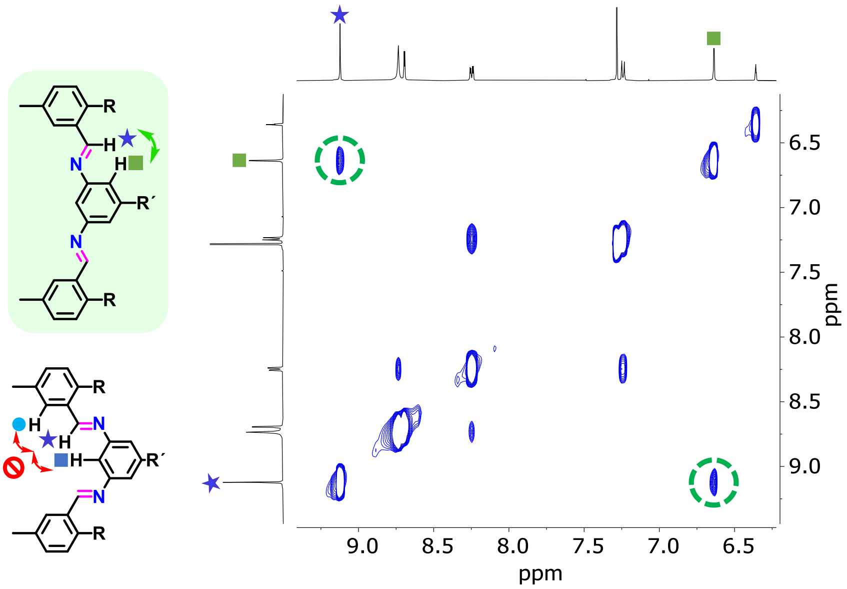

Figure S2C. 2D NOESY spectrum of the $\mathbf{1}_{Zn}^{CN}$ in $CDCl_3$ at 298 K, showing NOE cross-peaks that support the prevalence of an *extended* conformation. Cross-peaks between protons that could suggest the coexistence of a *compact* conformation were not observed.

Mass spectrometry

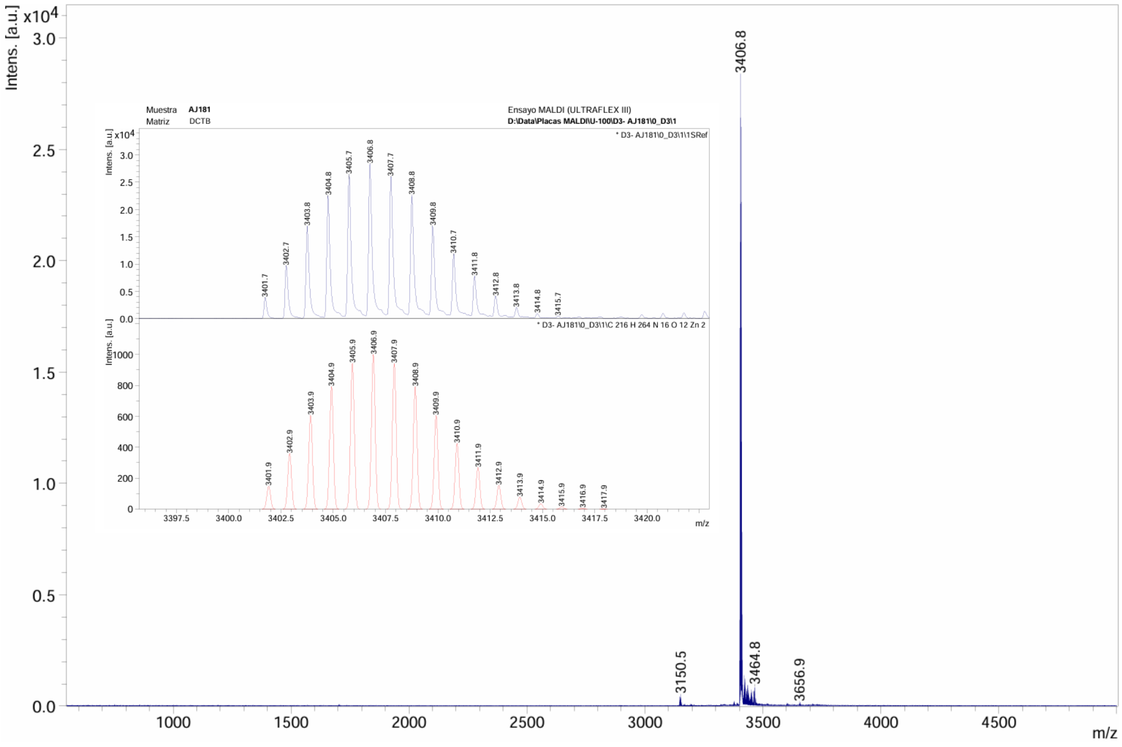

Figure S2D. MS (MALDI) spectra of $\mathbf{1}_{Zn}^{CN}$, showing the comparison of the experimental and theoretical isotopic distribution of the parent *ion* $[M]^+$.

Cavity size of 1_{Zn}^{CN}

Extended conformation	*Compact conformation*
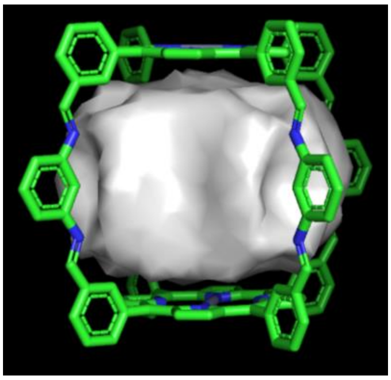	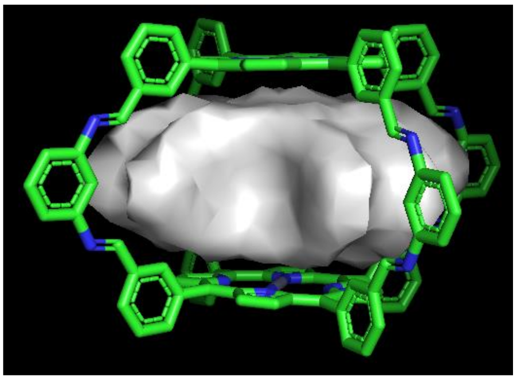
Volume of the cavity= **908.39 A^3**	Volume of the cavity= **641.17 A^3**

Figure S2E. Computed cavity size of the cage **1_{Zn}^{CN}** in both the extended and compact conformations. Carbon atoms are coloured in green, nitrogen atoms in blue and zinc atoms in grey.

S3. Temperature-dependent experiments of 1_{Zn}^{NC} and 1_{Zn}^{CN}

Temperature-dependent experiments were carried out in $CDCl_3$ (Figure S3) to ascertain whether different conformations - *extended* and *compact* – could be detected in slow exchange upon cooling the samples. However, a single set of signals was observed for both **1_{Zn}^{NC}** and **1_{Zn}^{CN}** along the whole temperature range, which, in combination with the NOESY data (see Figures S1C and S2C), is compatible with the prevalence of a single *extended* conformation. Upon cooling in $CDCl_3$, the only observation was that some proton signals did not shift significantly (pyrrolic protons, red circle), others shifted slightly downfield (some *meso*-arene protons, yellow circle), while most of them experienced minor up field shifts (imine proton, purple star; linker proton; blue square). These shifts were attributed to a rigidification and planarization of the π-conjugated system connecting the two porphyrins (including the linker, the imine bonds, and the *meso*-arenes) as the temperature decreases.

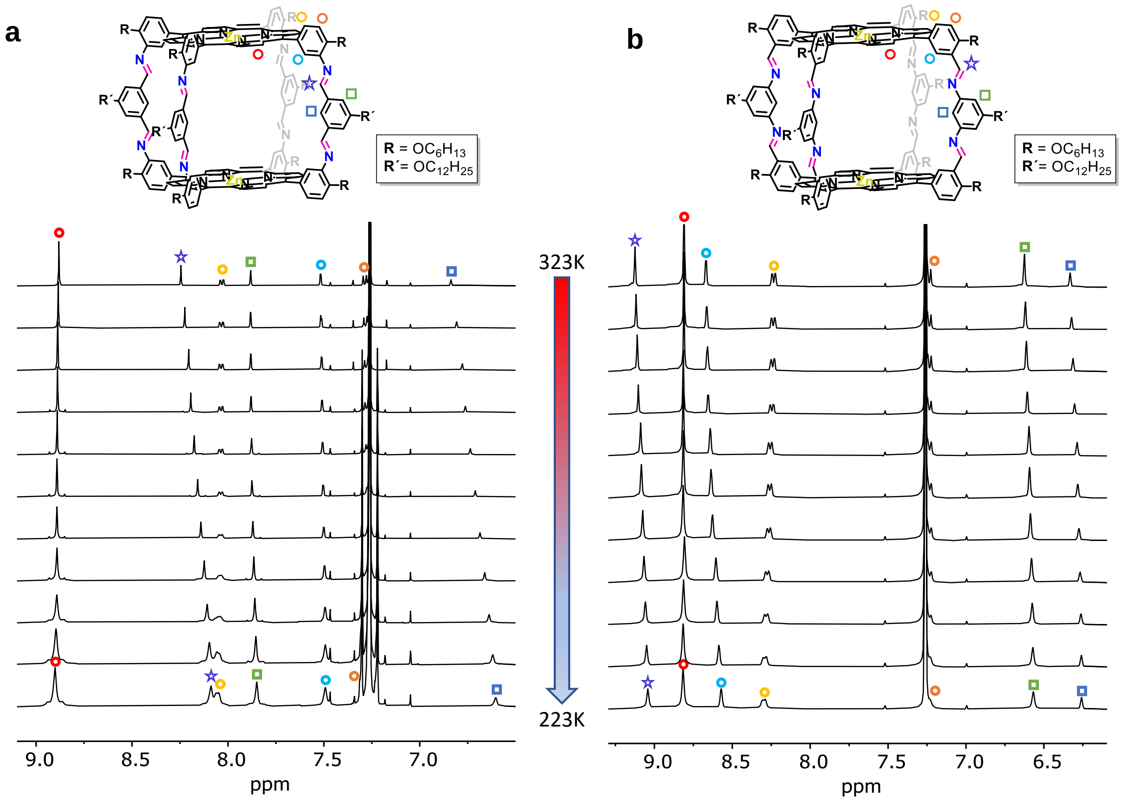

Figure S3. ^{1}H NMR spectra of (**a**) **1_{Zn}^{NC}** and (**b**) **1_{Zn}^{CN}** in $CDCl_3$ as a function of the temperature.

S4. Theoretical calculations on $\mathbf{1_{Zn}}^{NC}$ and $\mathbf{1_{Zn}}^{CN}$

Electronic structure calculations, ground state optimizations and relaxed scans along specific dihedral angles were performed using density functional theory (DFT) at the B3LYP/cc-PVDZ level of theory as implemented in Gaussian16C.[4-8]

Figure S4A shows the optimized structures calculated for the *extended* and *compact* conformations of $\mathbf{1_{Zn}}^{NC}$ and $\mathbf{1_{Zn}}^{CN}$. Figure S4B depicts the most relevant interactions determining the relative stability of both conformations, mainly determined by the interactions between the imine groups and the adjacent alkoxy groups attached to the meso-phenyl rings that largely destabilize the *compact* conformation for $\mathbf{1_{Zn}}^{CN}$. This was tested by replacing the EtO groups by H atoms to exchange the repulsive N···O interaction by an attractive N···H interaction. Finally, Figure S4C shows the potential energy surface for the rotation of the meso-phenyl ring with respect to the porphyrin.

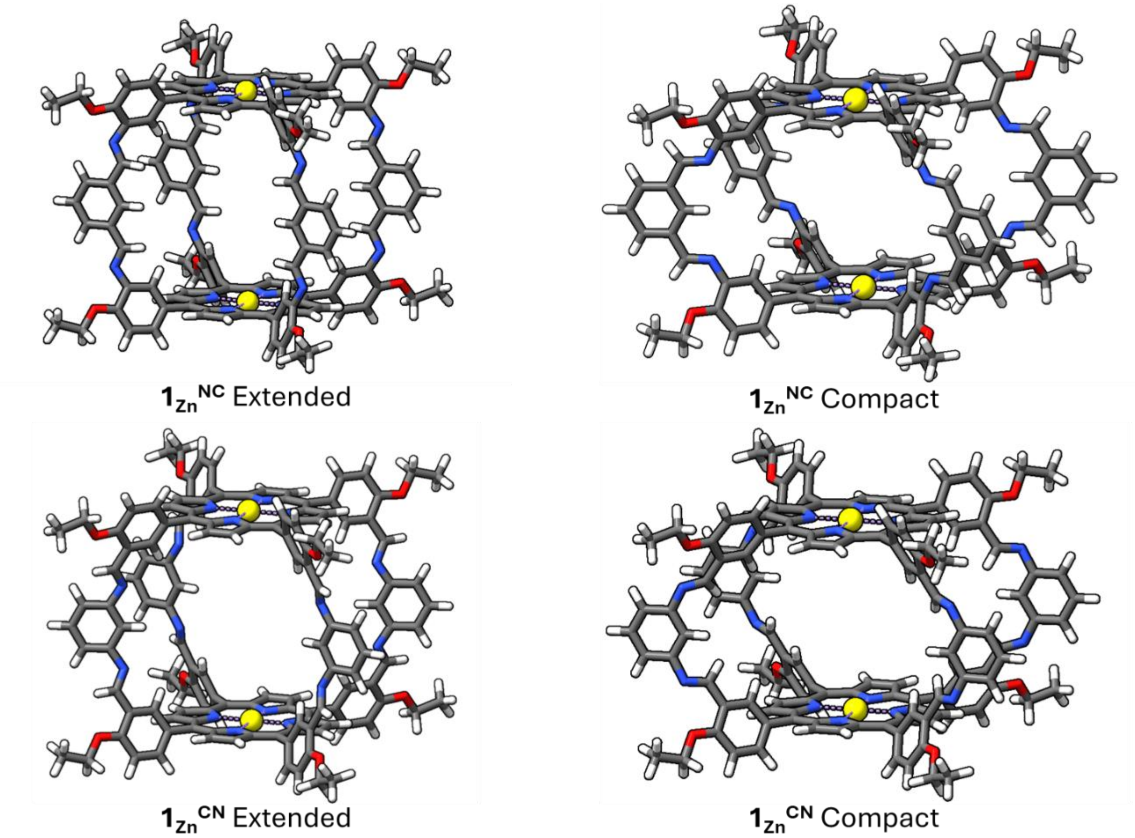

Figure S4A. Fully optimized minimum-energy structures calculated at the DFT B3LYP/cc-PVDZ level for the models of the $\mathbf{1_{Zn}}^{NC}$ (top) and $\mathbf{1_{Zn}}^{CN}$ (bottom) cages in *extended* (left) and *compact* (right) conformations.

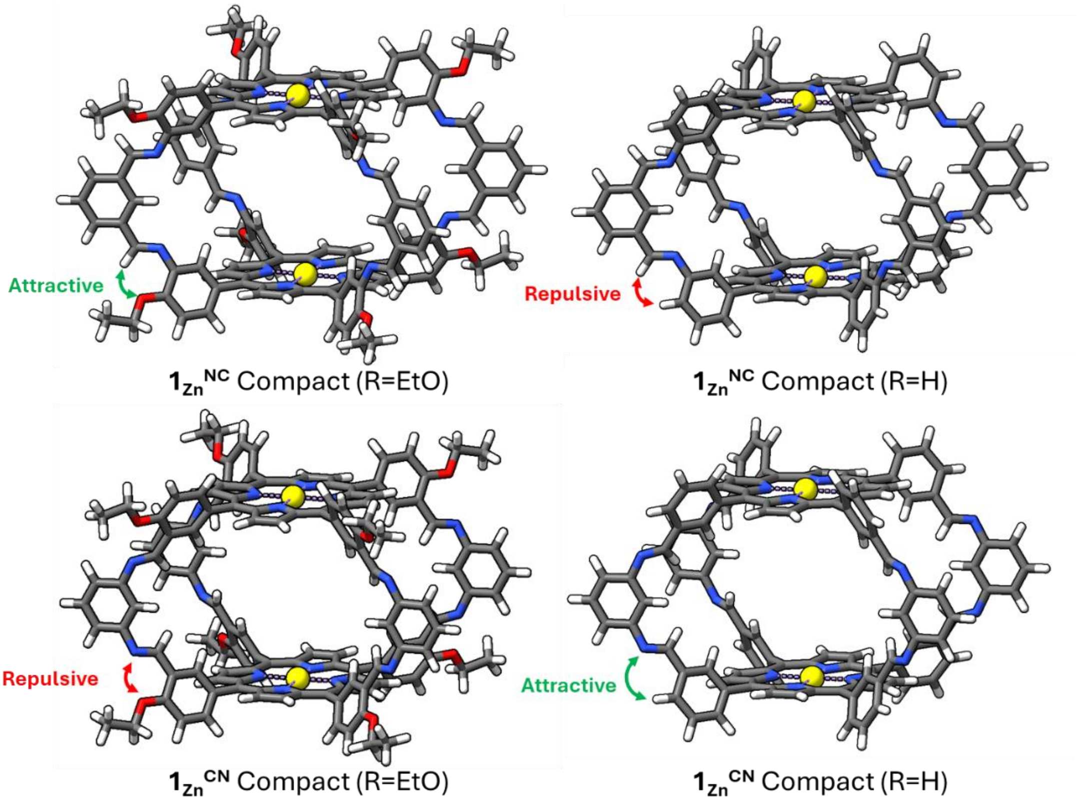

Figure S4B. Structures of the $\mathbf{1}_{Zn}^{NC}$ (top) and $\mathbf{1}_{Zn}^{CN}$ (bottom) models in *compact* conformation changing the R substituent attached to the *meso*-phenyl groups: R = EtO (left) or R = H (right). The relevant interactions between the R substituent and the imine groups are highlighted.

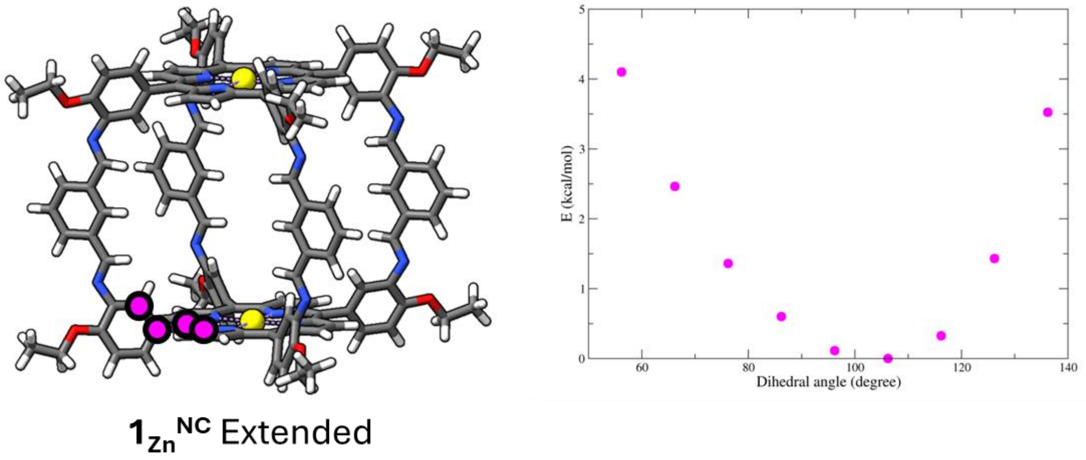

Figure S4C. Structure of $\mathbf{1}_{Zn}^{NC}$ in its extended conformation (left) and potential energy profile (right) calculated for the twisting of the *meso*-phenyl group around the dihedral angle highlighted with magenta circles. A 90º dihedral corresponds to the conformation where the *meso*-phenyl is fully perpendicular to the porphyrin ring.

S5. Calculation of reference association constants with monotopic and ditopic compounds

The association constants (K_a) between reference monotopic / monodentate compounds, *i.e.* $\mathbf{P_{Zn}}$ and either pyridine or benzylamine, were calculated in $CDCl_3$ (Figures S5A-B) by ^1H NMR titration experiments. The resulting binding isotherms were fitted to a 1:1 association model, affording K_a values in the 10^3-10^4 M^{-1} range, and displaying bound and unbound species in fast exchange in the ^1H NMR timescale. In general, the association with pyridine was slightly weaker than with the primary amine. On the other hand, the association constant with quinuclidine (Figure S5C) was calculated by titration experiments in $CHCl_3$ at lower concentrations, using in this case absorption spectroscopy, in view of the higher association strength, which is above 10^4 M^{-1}.

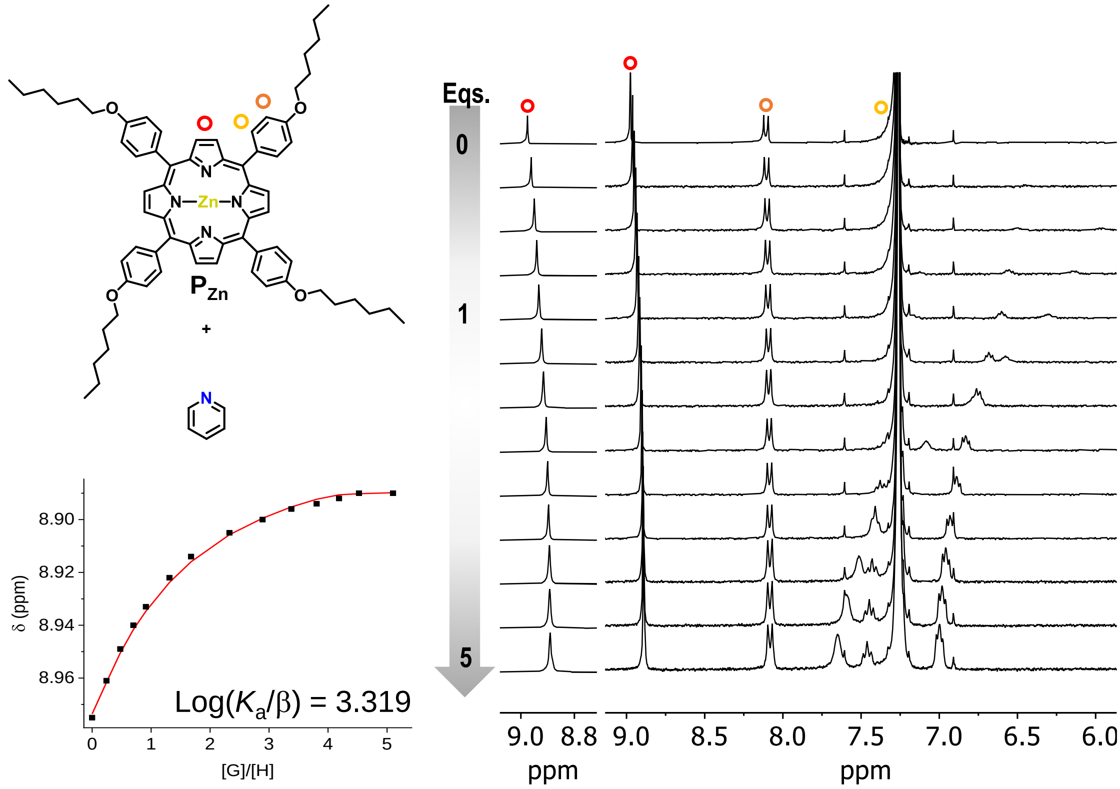

Figure S5A. ^1H NMR changes recorded along the titration of $\mathbf{P_{Zn}}$ with increasing amounts of pyridine at a constant $\mathbf{P_{Zn}}$ concentration of $5.0 \cdot 10^{-4}$ M in $CDCl_3$ at 298 K. Chemical shift variation of the β-pyrrolic protons (in ppm) as a function of the equivalents of pyridine added and association constant (K_a) calculated by fitting to a 1:1 model.

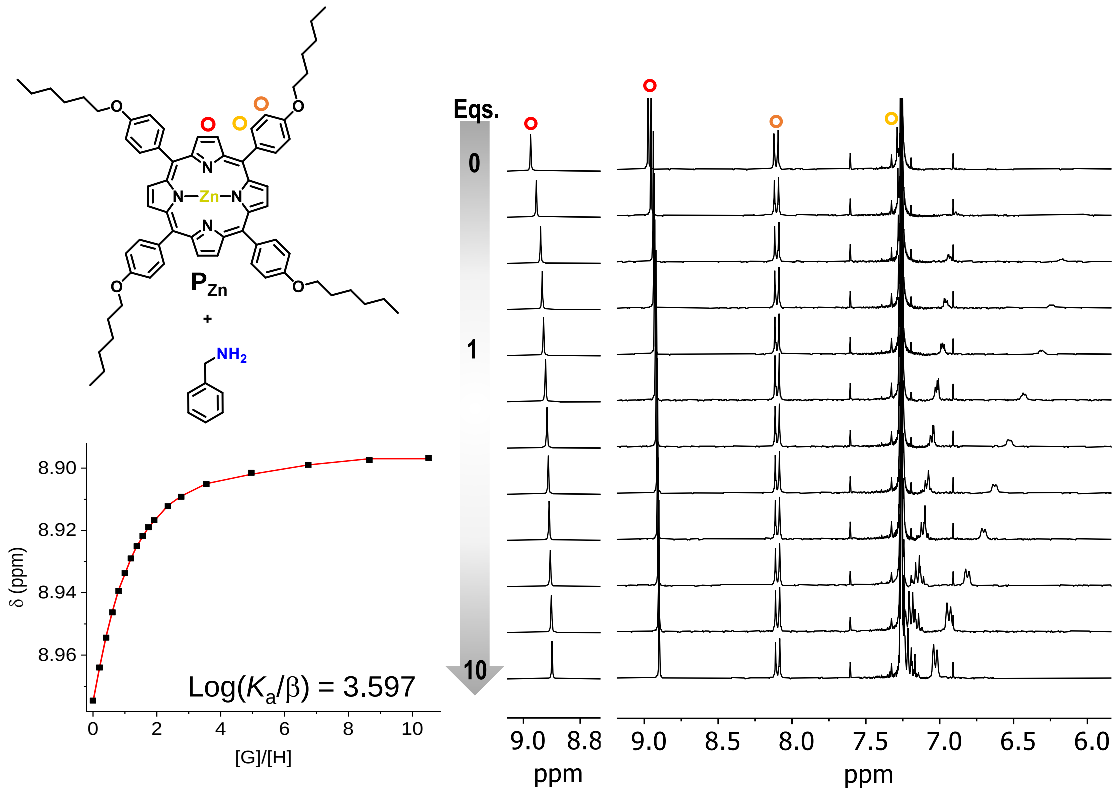

Figure S5B. ^{1}H NMR changes recorded along the titration of **P**$_{\mathbf{Zn}}$ with increasing amounts of benzylamine at a constant **P**$_{\mathbf{Zn}}$ concentration of $5.0 \cdot 10^{-4}$ M in $CDCl_3$ at 298 K. Chemical shift variation of the β-pyrrolic protons (in ppm) as a function of the equivalents of benzylamine added and association constant (K_a) calculated by fitting to a 1:1 model.

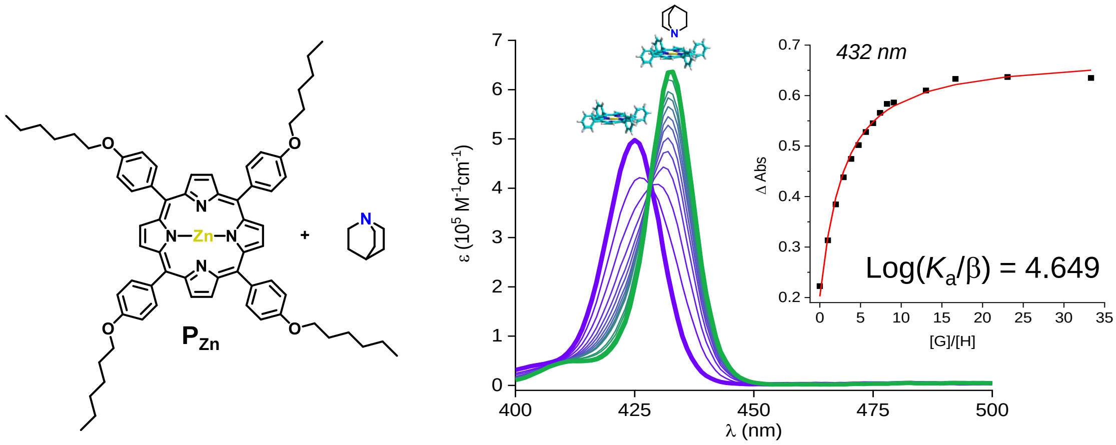

Figure S5C. UV-vis changes recorded along the titration of $\mathbf{P_{Zn}}$ with increasing amounts of quinuclidine at a constant $\mathbf{P_{Zn}}$ concentration of $1.0 \cdot 10^{-5}$ M in $CHCl_3$ at 298 K. The association constant (K_a) was calculated by fitting to a 1:1 model.

In addition, the association constants (K_a) between reference ditopic / bidentate compounds, *i.e.* **P_{Zn}** and either 4,4'-bipyridine, 2,6-naphtpyridine or DABCO, were calculated in $CHCl_3$ (Figures S5D-F) by UV-vis titration experiments at concentrations around 10^{-5} M. Such low concentrations are required to avoid the formation of the corresponding 2:1 complexes. The resulting binding isotherms were fitted to a 1:1 association model, affording K_a values in the 10^2-10^5 M^{-1} range. The use of half of the value of these association constants from ditopic guests as reference values (K_{ref}) to calculate *EM* takes into account intramolecular electronic effects that are not present in the corresponding monotopic guests, like pyridine.

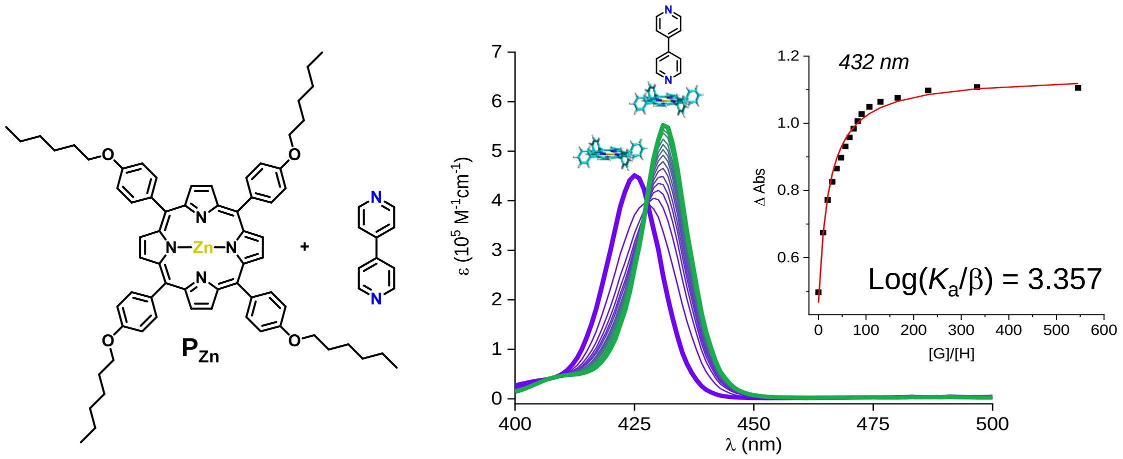

Figure S5D. UV-vis changes recorded along the titration of **P_{Zn}** with increasing amounts of *bipy* at a constant **P_{Zn}** concentration of $2.0\cdot10^{-5}$ M in $CHCl_3$ at 298 K. The association constant (K_a) was calculated by fitting to a 1:1 model.

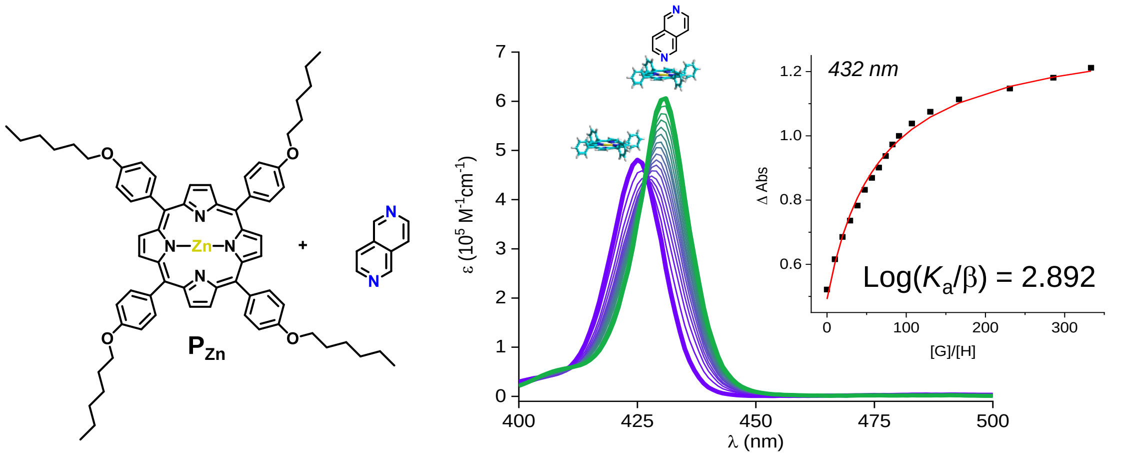

Figure S5E. UV-vis changes recorded along the titration of **P_{Zn}** with increasing amounts of *naphy* at a constant **P_{Zn}** concentration of $2.0\cdot10^{-5}$ M in $CHCl_3$ at 298 K. The association constant (K_a) was calculated by fitting to a 1:1 model.

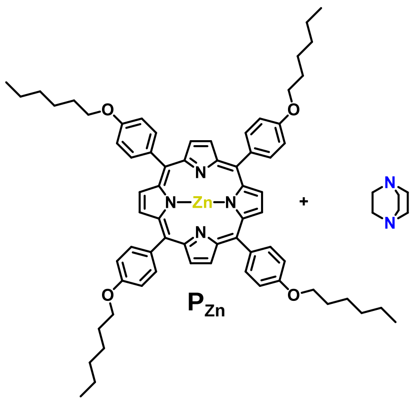

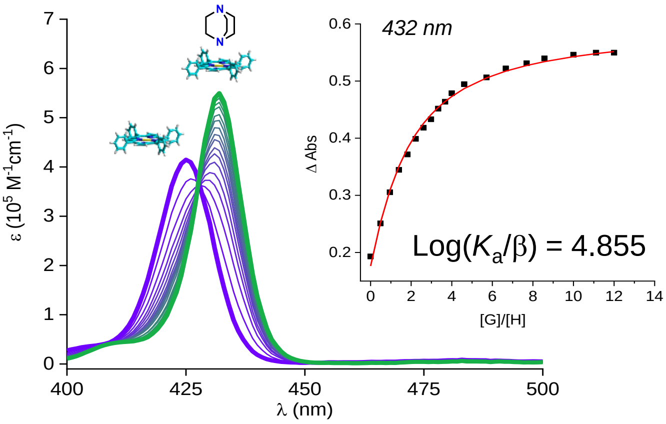

Figure S5F. UV-vis changes recorded along the titration of $\mathbf{P_{Zn}}$ with increasing amounts of DABCO at a constant $\mathbf{P_{Zn}}$ concentration of $1.0 \cdot 10^{-5}$ M in $CHCl_3$ at 298 K. The association constant (K_a) was calculated by fitting to a 1:1 model.

S6. ^{1}H NMR titration experiments with representative molecules from *Groups 1* and *4.*

Guest molecules of *Groups 1* and *4* are those that present their nitrogen atoms at either too large (for example: bis(4-pyridyl)acetylene or 1,4-bis(4'-pyridyl)butadiyne) or too short (for example: DABCO) distances, respectively, to bind to both Zn^{II} atoms within the cage cavity. Since they can only bind to one Zn^{II} center, their supramolecular behaviour and coordination strength is similar to that of regular Zn^{II}*P*s and monotopic guests, like pyridine or benzylamine (see Figure S5A-B), leading to supramolecular species that are in fast NMR exchange, as shown in Figures S6A-B for the $\mathbf{1}_{Zn}^{NC}$ cage and the $\mathbf{P}_{Zn}$ reference compound. The main difference with respect to the monotopic guests is the possibility of formation of the 2:1 complex, whose abundance is maximized upon addition of 0.5 eq. of dinitrogen ligand, resulting in up field shifts for both the cage and the monotopic P. These shifts are more pronounced for DABCO, as known and expected in view of the closer distance between Zn^{II}*P* planes. Upon surpassing 1 equivalents, the formation of the 1:1 complex (per Zn^{II}*P*) is then maximized due to saturation of the Zn(II) centers.

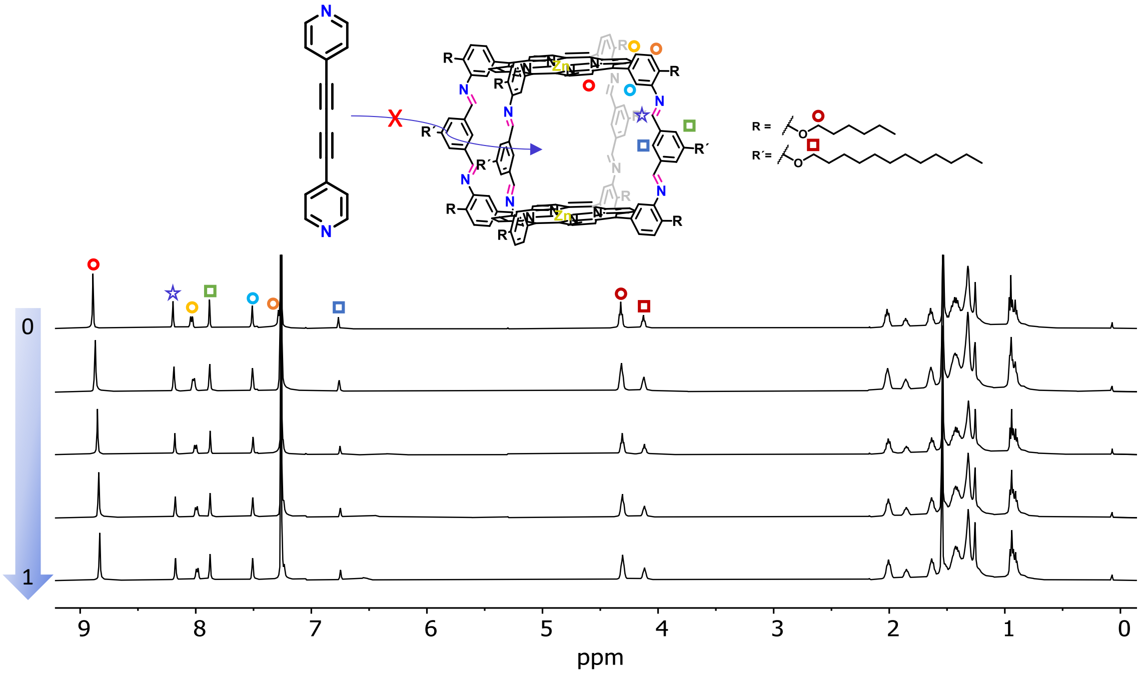

Figure S6A. ^{1}H NMR changes recorded along the titration of $\mathbf{1}_{Zn}^{NC}$ with increasing amounts of 1,4-bis(4'-pyridyl)butadiyne at a constant $\mathbf{1}_{Zn}^{NC}$ concentration of $1.0 \cdot 10^{-3}$ M in $CDCl_3$ at 298 K.

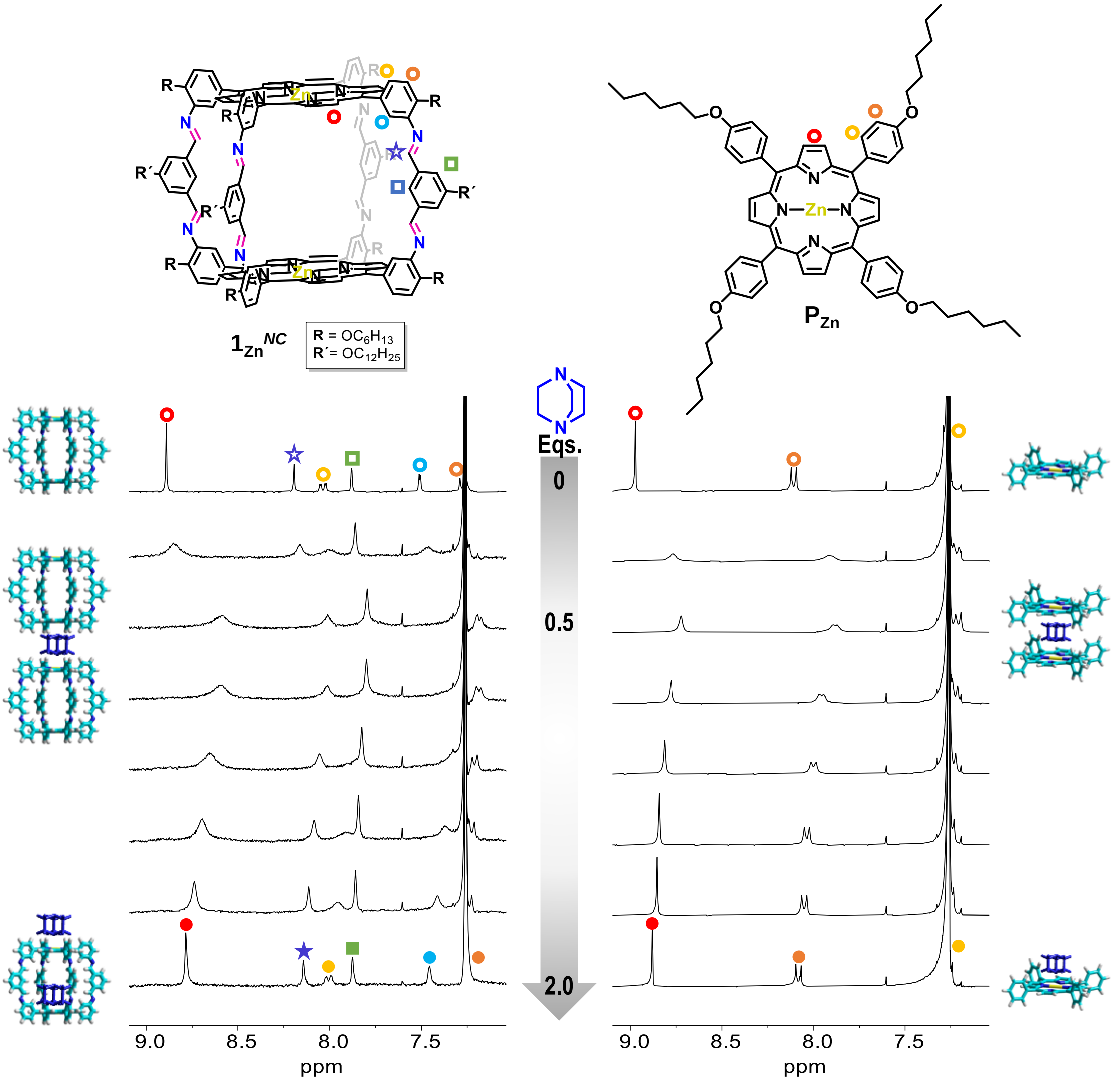

Figure S6B. Comparison of the ^{1}H NMR changes recorded along the titration of (left) $\mathbf{1_{Zn}}^{NC}$ and (right) $\mathbf{P_{Zn}}$ with increasing amounts of DABCO at a constant $\mathbf{1_{Zn}}^{NC}$/$\mathbf{P_{Zn}}$ concentration of $5.0 \cdot 10^{-4}$ M in $CDCl_3$ at 298 K. The 2:1 complex is favoured at 0.5 eqs. of added DABCO.

S7. Host-Guest Chemistry. Binding of 4,4'-bipyridine (*bipy*) to $\mathbf{1}_{Zn}{}^{NC}$

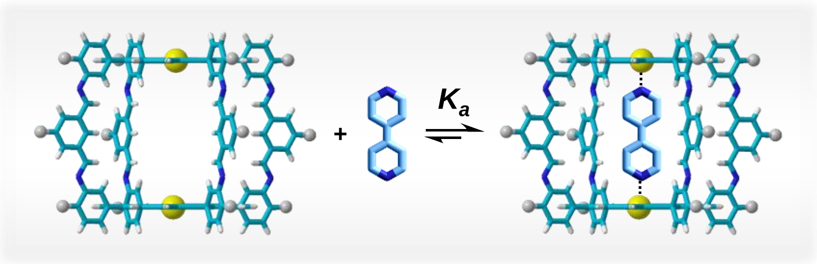

Binding of guest molecules in *Group 2*, like 4,4'-bipyridine (*bipy*), having 2 nitrogen atoms at just the right distance to bind to both Zn^{II} centers in the cavity of $\mathbf{1}_{Zn}{}^{NC}$ in the *extended* conformation, was first evaluated through titration experiments monitored by ^{1}H NMR in $CDCl_3$ (Figures S7A) and UV-vis in $CHCl_3$ (Figure S7B).

^{1}H NMR Titrations

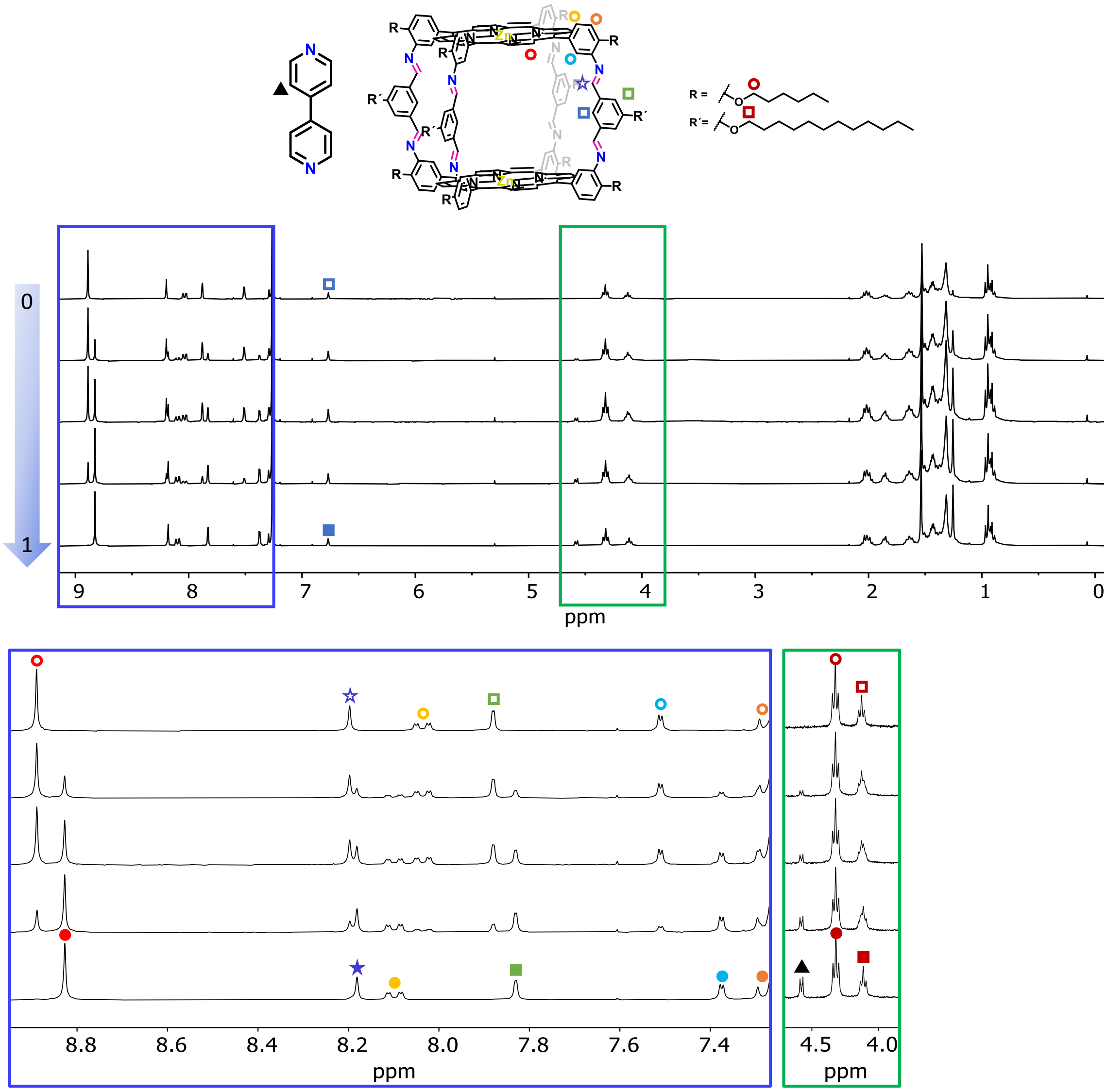

Figure S7A. ^{1}H NMR changes recorded along the titration of $\mathbf{1}_{Zn}{}^{NC}$ with increasing amounts of *bipy* in $CDCl_3$ at 298 K.

UV-vis Titrations

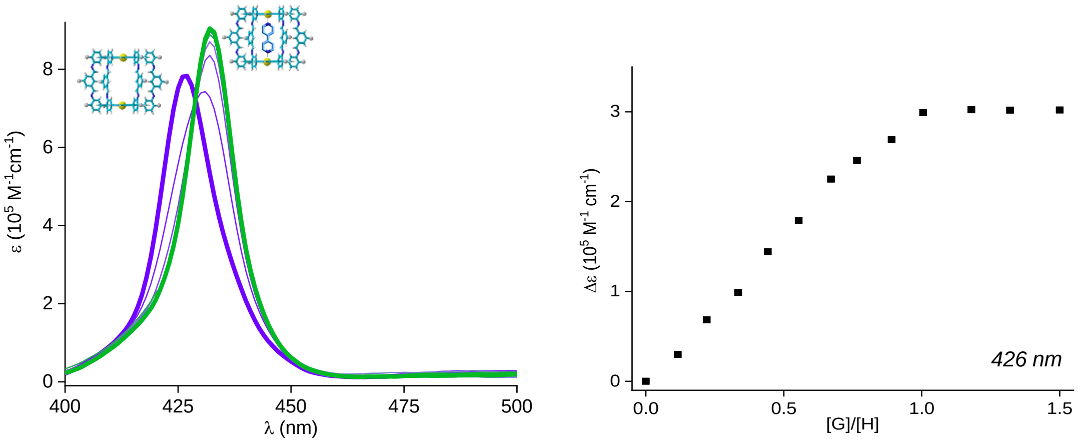

Figure S7B. UV-vis changes recorded along the titration of $\mathbf{1}_{\mathbf{Zn}}^{NC}$ with increasing amounts of *bipy* in $CHCl_3$ at a constant concentration of $1.0 \cdot 10^{-7}$ M. The binding isotherm could not be properly fitted to a 1:1 model due to a too strong binding.

NOESY Experiments

On the other hand, a NOESY spectrum in $CDCl_3$ (Figure S7C) confirmed that the $\mathbf{1}_{\mathbf{Zn}}^{NC}$ cage maintained the *extended* conformation upon binding to the *bipy* guest, since the same cross peaks were observed for the relevant protons as for the empty $\mathbf{1}_{\mathbf{Zn}}^{NC}$ cage (please compare with Figure S1C).

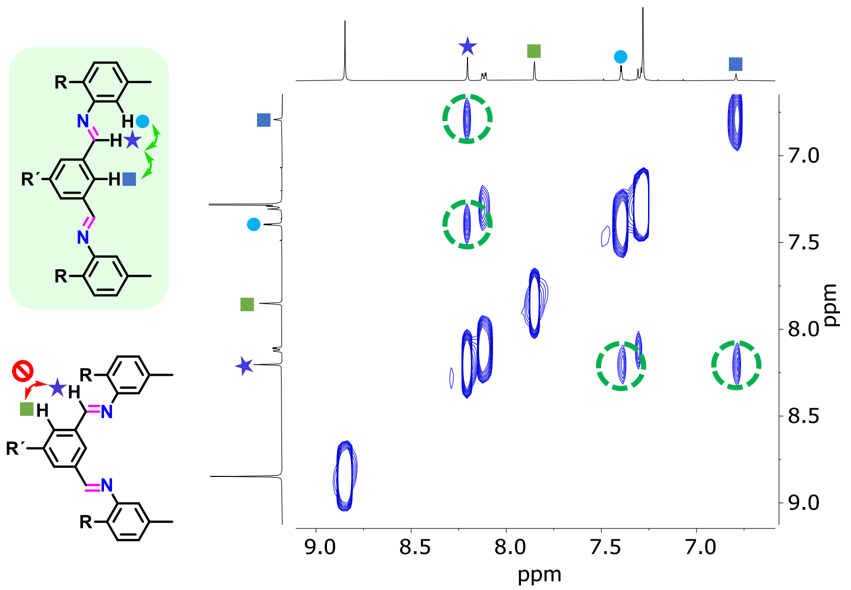

Figure S7C. 2D NOESY spectrum of the 1:1 $\mathbf{1}_{\mathbf{Zn}}^{NC}$·*bipy* complex in $CDCl_3$ at 298 K, showing NOE cross-peaks that support the prevalence of an extended conformation.

DOSY Experiments

DOSY experiments of 1:1 $\mathbf{1}_{Zn}^{NC}$·*bipy* mixture in $CDCl_3$ (Figure S7D) revealed host and guest diffusing with the same diffusion coefficient, which is an additional proof of their strong association.

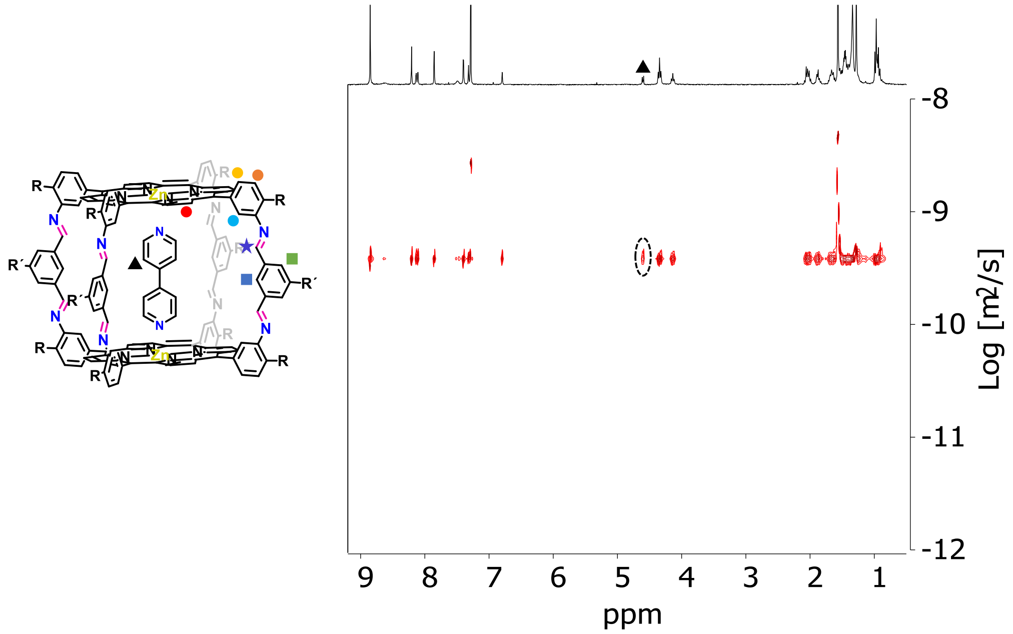

Figure S7D. 2D DOSY spectrum of the 1:1 $\mathbf{1}_{Zn}^{NC}$·*bipy* complex in $CDCl_3$ at 298 K, showing the signals of both host and guest at the same diffusion coefficient.

^{1}H NMR Competition Experiments

Since titrations of **1**$_{Zn}$^{NC} with increasing amounts of *bipy* monitored within the ^{1}H NMR and UV-vis concentration range (Figures S7A and S7B) revealed a too strong association in $CDCl_3$/$CHCl_3$ with binding isotherms that rather fit to two straight lines crossing at 1.0 eqs., association constants could not be calculated by these techniques. In other words, every addition of *bipy* resulted in quantitative complex formation within this concentration window. Reducing the concentration and employing more sensitive techniques, like fluorescence spectroscopy, did not help in this case, because the addition of *bipy* did not lead to significant emission changes.

Instead, association constants between **1**$_{Zn}$^{NC} and *bipy* in $CDCl_3$ were calculated through competition experiments monitored by ^{1}H NMR (Figures S7E). First, a dinitrogen ligand that presented a weaker binding than *bipy* and whose association constant could be determined by direct titration experiments (K_{a1}) was selected. Our option for $CDCl_3$ was *m*-xylylenediamine (*m*-xyda; $K_{a1} = 8.1 \cdot 10^6$; see Section S10). Then, a competition was established between *bipy* and *m*-xyda: increasing amounts of *m*-xyda were added to the **1**$_{Zn}$^{NC}·*bipy* complex in $CDCl_3$. From the average integration of several proton signals in the ^{1}H NMR spectra obtained at different *bipy* : *m*-xyda ratios, the equilibrium constant of the competition (K_C) was calculated. Then, from the K_{a1} and K_C values, the association constant between **1**$_{Zn}$^{NC} and *bipy* (K_{a2}) could be calculated as shown in the top schemes in Figure S7E.

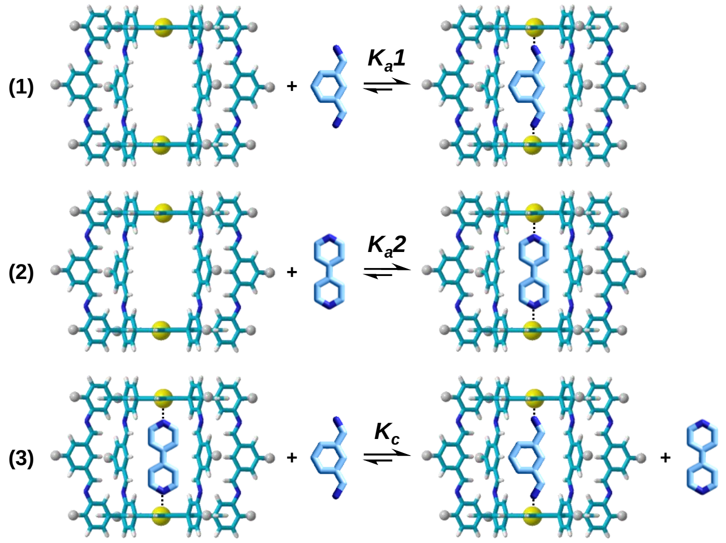

$$K_c = (1) - (2) = \frac{K_a1}{K_a2} \qquad K_c = 0.069$$

$\mathrm{Log}(K_a1/\beta) = 6.909$

$\mathrm{Log}(K_a2/\beta) = 8.070$

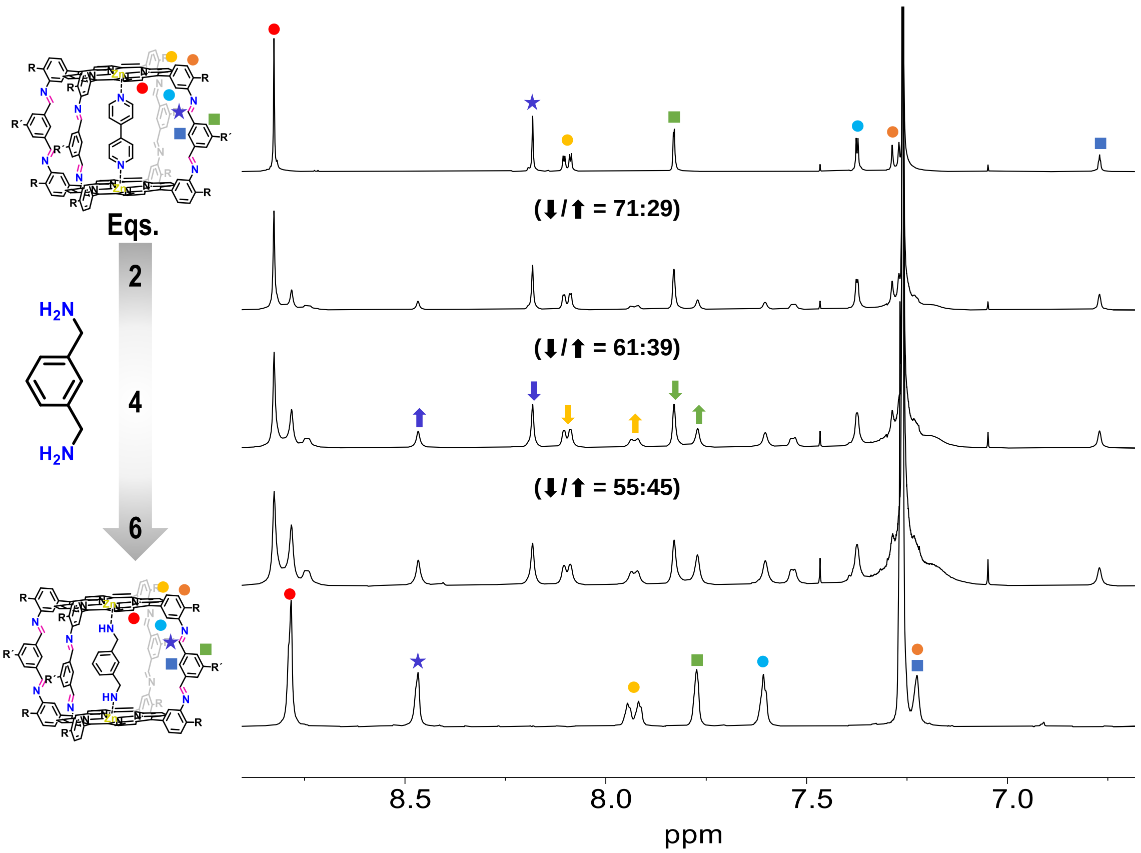

Figure S7E. ^{1}H NMR spectra acquired in the competition experiment between *bipy* and *m-xyda* for the cavity of $\mathbf{1}_{Zn}{}^{NC}$ in $CDCl_3$. 2.0, 4.0 and 6.0 eqs of *m-xyda* were added to a 1:1 complex of $\mathbf{1}_{Zn}{}^{NC}$·*bipy* at a constant concentration of $1.0 \cdot 10^{-3}$ M, in $CDCl_3$ at 298 K**.**

EXSY Experiments

As shown in the NMR titrations of $\mathbf{1}_{Zn}^{NC}$/$\mathbf{1}_{Zn}^{CN}$ with *Group 2* guests displayed in Figures S7-S11, at substoichiometric amounts of guest, empty and bound cages are clearly detected in slow exchange at the NMR timescale. When a small excess of guest is added, only the fraction bound within the cage is detected by ^{1}H NMR, the free guest displaying broad NMR signals. Only upon addition of a large excess of guest, its broad signals are detected in the spectra. These slow processes prompted us to perform 2D EXSY experiments of *ca*. 1:0.55 mixtures of $\mathbf{1}_{Zn}^{NC}$ and *bipy*. Figure S7F shows the non-uniform sampling (NUS) 2D NOESY spectra of a 1:0.55 mixture of $\mathbf{1}_{Zn}^{NC}$ and *bipy* in $CDCl_3$ at a mixing time of τ_m = 0.0 s (Figure S7Fa), τ_m = 400 ms and τ_m = 2500 ms (Figure S7Fb). The latter parameter was chosen based on preliminary 1D NOE experiments at different mixing times, and already speaks about the extremely slow nature of the exchange process, in comparison to other supramolecular systems.

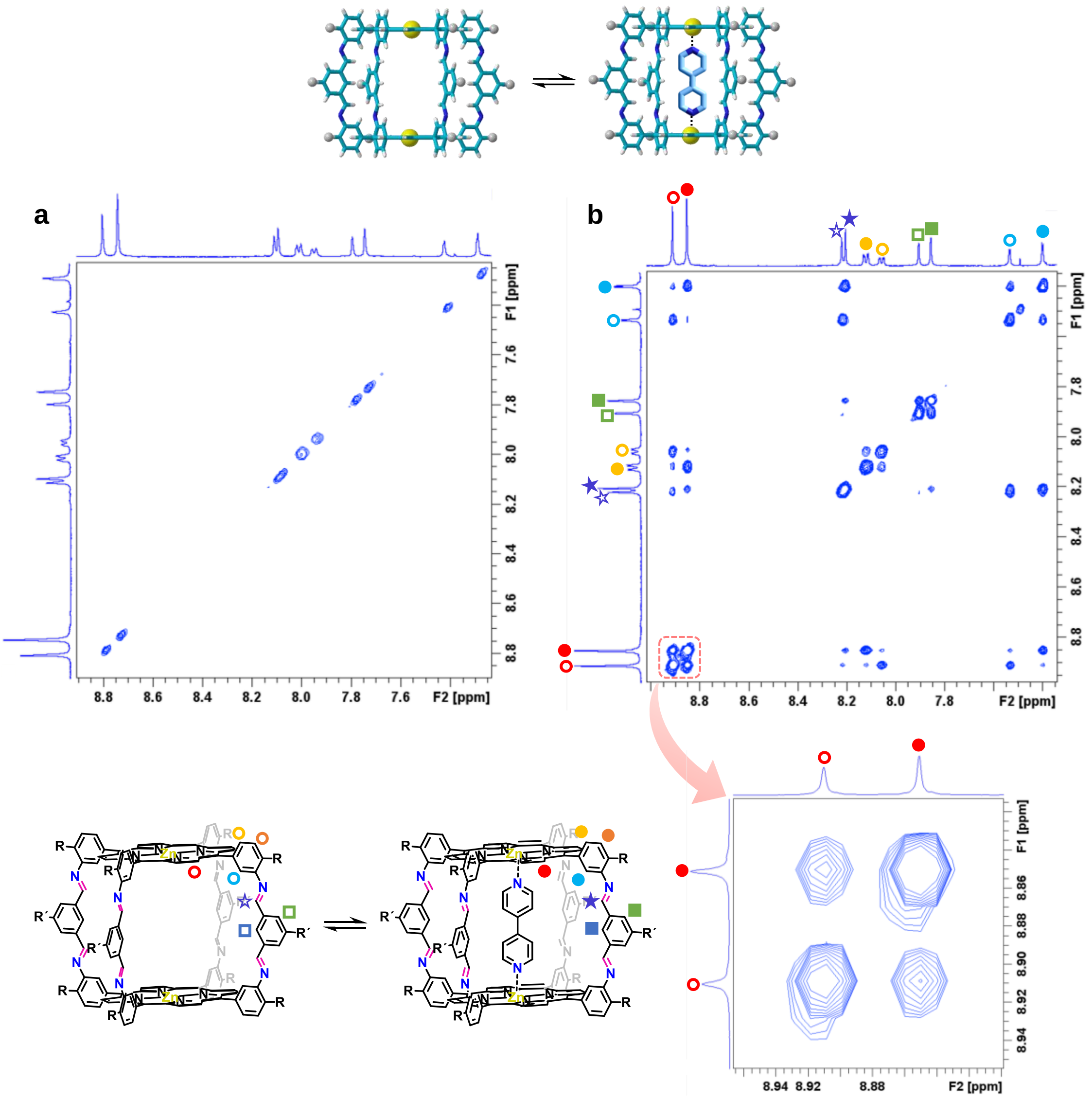

Figure S7F. 2D EXSY spectra of $\mathbf{1}_{Zn}^{NC}$ with 0.55 eq. of *bipy*. at a constant concentration of $1.0 \cdot 10^{-3}$ M at a mixing time of (**a**) τ_m = 0.0 s and (**b**) τ_m = 2.5 s in $CDCl_3$ at 298 K**.**

In order to calculate the exchange rate constants, the data at τ_m = 2500 ms was analyzed in two ways:

a) Using the equations shown below, where *k* is the exchange rate constant, τ_m is the mixing time, X_A and X_B are the molar fractions of molecules in states A and B, respectively, I_{AA} and I_{BB} are the diagonal peak intensities, and I_{AB} and I_{BA} are the cross-peak intensities, we obtained values for *k*, which are the sum of the forward (association; k_1) and backward (dissociation; k_{-1}) pseudo-first order rate constants for the assembly process.

$$k = \frac{1}{\tau_m} ln\, ln\, \frac{r+1}{r-1} \qquad r = 4X_A X_B \frac{I_{AA}+I_{BB}}{I_{AB}+I_{BA}} - (X_A - X_B)^2$$

b) Using the software EXSY Calc (from MestreLab Research), which affords a quantitative analysis of the experimental intensities of the NMR peaks obtained in EXSY experiments to calculate the magnetization exchange rates of the exchange equilibrium.

The kinetic data obtained by both methods at different mixing times is summarized in the following Table.

	**Method a**	**Method b**
$\boldsymbol{\tau_m}$ ms	***k*** s^{-1}	***k*** s^{-1}
2500	**0.258**	**0.217**

The calculated *k* values from both methods are similar, in between 0.26 and 0.21 s^{-1}. An average value of $\boldsymbol{k_{ex}}$ **= 0.24 ± 0.03 s^{-1}** was taken for the $\mathbf{1_{Zn}}^{NC}$·*bipy* - $\mathbf{1_{Zn}}^{NC}$ exchange rate constant in $CDCl_3$ at 298 K.

Solid state crystal structure of $\mathbf{1_{Zn}}^{NC}$·*bipy*

Single crystals of the supramolecular complex $\mathbf{1_{Zn}}^{NC}$·*bipy* were grown by a slow cooling process from 140 to 25 ºC of a saturated solution of $\mathbf{1_{Zn}}^{NC}$·*bipy* in DMF.

The cage complex $\mathbf{1_{Zn}}^{NC}$·*bipy* crystallized in the monoclinic $P2_1/c$ space group, which was the same than the one obtained for the empty cage. The crystal structure presented interstitial molecules of DMF and water in both the inner and outer areas of the cage (hidden for clarity).

The crystal structure of the $\mathbf{1_{Zn}}^{NC}$·*bipy* complex exhibited again mainly the *extended* conformation, attending to the arrangement of the imine bonds. However, a closer analysis revealed that 6 out of 8 of these imine bonds presented the alternance of the *extended* conformation, while the other 2 actually displayed a conformation corresponding to the *compact* form of the cage. Interestingly, the two imine bonds in the *compact* conformation can be related between them through an inversion center.

Most notably, the shape of the cage remained the same upon complexation with *bipy* (pseudo-D_{2h} symmetry), showing a cube-like geometry with square and rectangular faces. The presence of the *bipy* molecule coordinated in the cavity of the cage slightly reduced the differences between the sides of the square shaped pore from 1.7 Å to 1.1 Å, and in the rectangular shaped pore from 3.2 Å to 2.6 Å.

The Zn···Zn distance decreased from 12.02 Å to 11.53 Å due to the complexation with *bipy*, and the Zn···N distance between the metalloporphyrin and the nitrogenated guest was found as 2.20 Å, in agreement with typical bond length reported in the bibliography for the coordination of pyridine derivatives with Zn(II)porphyrins.[9-10] The shortening of the porphyrin-porphyrin distance was mainly achieved by decreasing the torsional angles formed between the *meso*-aryl substituents and the porphyrin plane from 65-70º (empty cage) to 61-68º, combined with the existence of imine bonds with a different conformation, as explained above.

The packing of the unit cell was very similar to the one displayed by the empty cage, demonstrating that in this case the presence of the guest molecule, which did not drastically modify the shape and size of the cage, did not affect either the organization of the crystal.

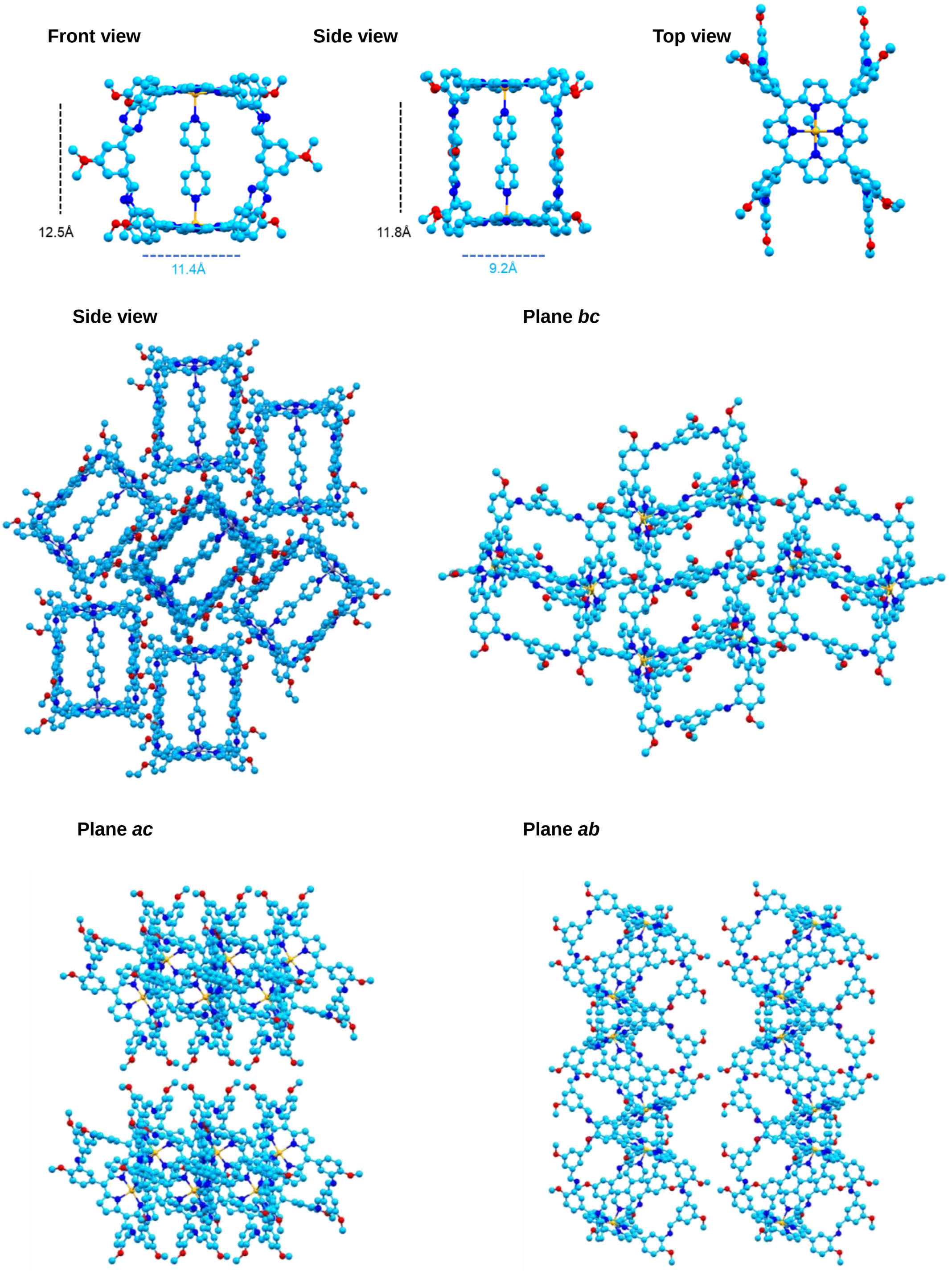

Figure S7G. Crystal structure and packing of the complex $\mathbf{1}_{Zn}^{NC}$·*bipy*. The solubilizing alkyl chains and hydrogen atoms were omitted for clarity. Residual solvent molecules are also omitted. The top images show selected front, side and top views of the cage and the packing arrangement. The bottom images show the corresponding packing as collected from side view and planes *bc*, *ac*, and *ab* of the unit cell.

Table S2. Crystallographic data of $\mathbf{1}_{Zn}^{NC}$·*bipy*

CCDC number	2375304
Empirical formula*	$C_{195}H_{197.5}N_{17}O_{12}Zn_2$,3 ($C_3H_7NO$)
Formula weight	3321.21
Temperature [K]	150.00(10)
Crystal system	monoclinic
Space group	$P2_1/c$
a [Å]	23.4209(5)
b [Å]	31.4456(4)
c [Å]	15.0148(2)
α [°]	90
β [°]	93.3440(10)
γ [°]	90
Volume [$Å^3$]	11039.3(3)
Z	2
ρ_{calc} [g/cm^3]	0.999
μ [mm^{-1}]	0.708
λ	Cu Kα (λ = 1.54184)
2Θ range for data collection/°	4.71 to 136.838
Data/restraints/parameters	20204/1098/1259
Goodness-of-fit on F^2	1.011
R [I>=2σ (I)]	0.1547
wR_2	0.4545

*The empirical formula was deduced from the atoms that could be modeled from the crystal structure.

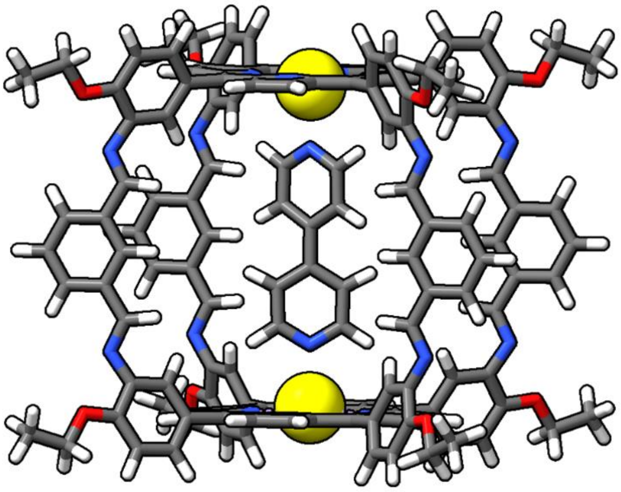

Figure S7H. B3LYP/cc-PVDZ-optimized structures calculated for $\mathbf{1}_{Zn}^{NC}$·*bipy*.

S8. Host-Guest Chemistry. Binding of 4,4'-bipyridine (*bipy*) to 1_{Zn}^{CN}

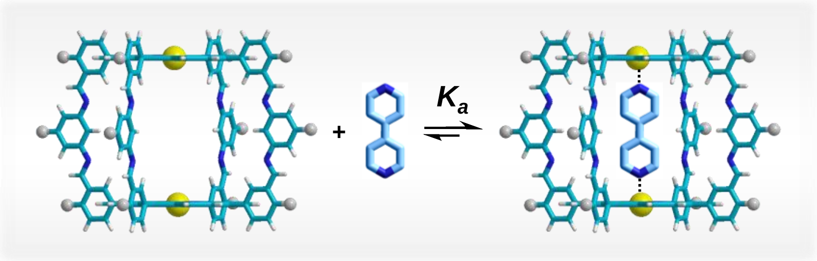

Binding of guest molecules in *Group 2*, like 4,4'-bipyridine (*bipy*), having 2 nitrogen atoms at just the right distance to bind to both Zn^{II} centers in the cavity of $\mathbf{1}_{Zn}^{CN}$ in the *extended* conformation, was first evaluated through titration experiments monitored by ^{1}H NMR (Figures S8A) in $CDCl_3$ and UV-vis (Figure S8B) in $CHCl_3$.

^{1}H NMR Titrations

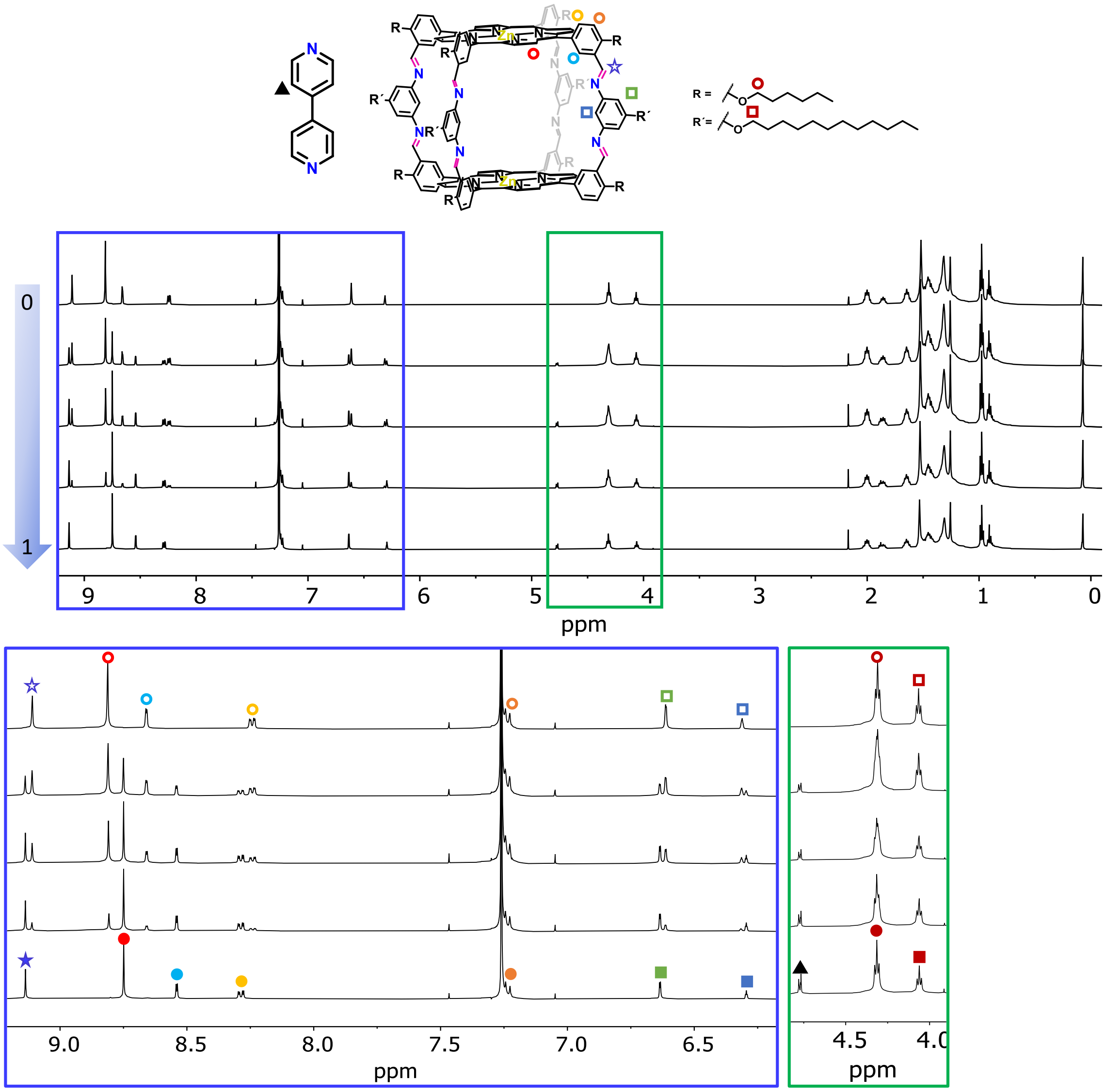

Figure S8A. ^{1}H NMR changes recorded along the titration of $\mathbf{1}_{Zn}^{CN}$ with increasing amounts of *bipy* in $CDCl_3$ at 298 K.

UV-vis Titrations

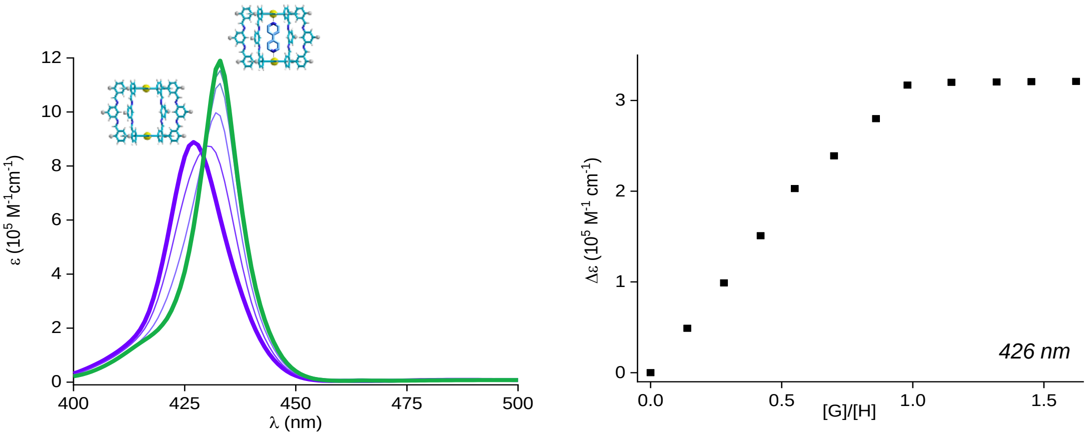

Figure S8B. UV-vis changes recorded along the titration of $\mathbf{1}_{Zn}^{CN}$ with increasing amounts of *bipy* in $CHCl_3$ at a constant concentration of $1.0 \cdot 10^{-7}$ M. The binding isotherm could not be properly fitted to a 1:1 model due to a too strong binding.

^{1}H NMR Competition Experiments

Since titrations of $\mathbf{1}_{Zn}^{CN}$ with increasing amounts of *bipy* monitored within the ^{1}H NMR and UV-vis concentration range (Figures S8A and S8B) revealed a too strong association in $CDCl_3$/$CHCl_3$ with binding isotherms that rather fit to two straight lines crossing at 1.0 eqs., association constants could not be calculated by these techniques. In other words, every addition of *bipy* resulted in quantitative complex formation within this concentration window. Reducing the concentration and employing more sensitive techniques, like fluorescence spectroscopy, did not help in this case, because the addition of *bipy* did not lead to significant emission changes.

Instead, association constants between $\mathbf{1}_{Zn}^{CN}$ and *bipy* in $CDCl_3$ were calculated through competition experiments monitored by ^{1}H NMR (Figure S8C). First, a dinitrogen ligand that presented a weaker binding than *bipy* and whose association constant could be determined by direct titration experiments (K_{a1}) was selected. Our option for $CDCl_3$ was *m*-xylylenediamine (*m*-xyda; $K_{a1} = 8.8 \cdot 10^7$; see Section S11). Then, a competition was established between *bipy* and *m*-xyda: increasing amounts of *m*-xyda were added to the $\mathbf{1}_{Zn}^{CN} \cdot$*bipy* complex in $CDCl_3$, respectively. From the average integration of several proton signals in the ^{1}H NMR spectra obtained at different *bipy* / *m*-xyda ratios, the equilibrium constant of the competition (K_C) was calculated. Then, from the K_{a1} and K_C values, the association constant between $\mathbf{1}_{Zn}^{CN}$ and *bipy* (K_{a2}) could be calculated as shown in the top schemes in Figure S8C.

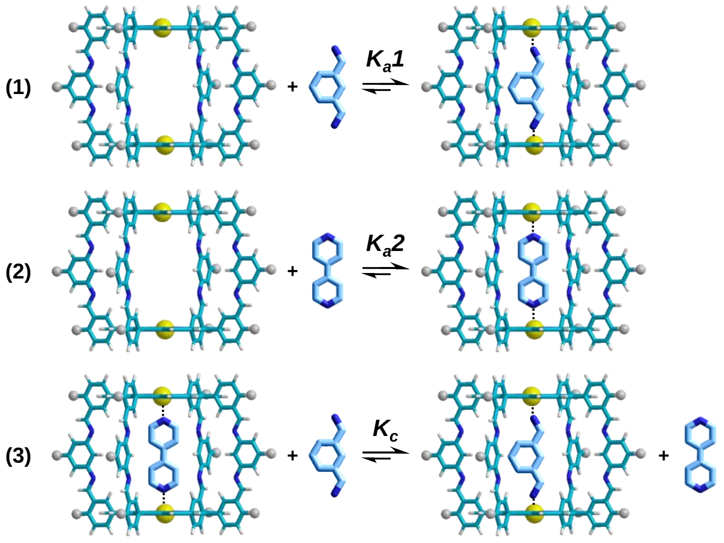

$$K_c = (1) - (2) = \frac{K_a1}{K_a2} \qquad K_c = 0.112$$

$\text{Log}(K_a1/\beta) = 7.946$

$\text{Log}(K_a2/\beta) = 8.900$

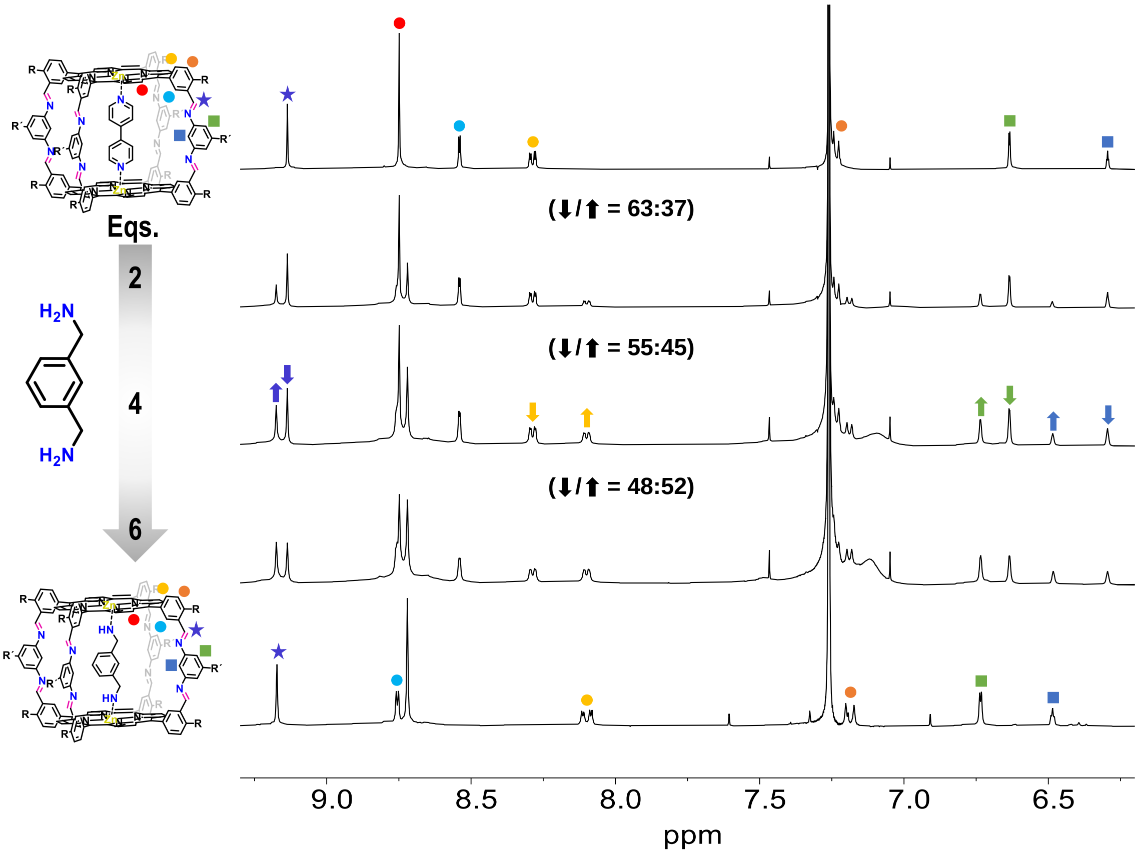

Figure S8C. ^{1}H NMR spectra acquired in the competition experiment between *bipy* and *m-xyda* for the cavity of $\mathbf{1}_{Zn}^{CN}$. 2.0, 4.0 and 6.0 eqs of *m-xyda* were added to a 1:1 complex of $\mathbf{1}_{Zn}^{CN}\cdot$*bipy* at a constant concentration of $1.0\cdot10^{-3}$ M, in $CDCl_3$ at 298 K.

EXSY Experiments

As shown in the NMR titrations of $\mathbf{1}_{Zn}{}^{NC}/\mathbf{1}_{Zn}{}^{CN}$ with *Group 2* guests displayed in Figures S7-S11, at substoichiometric amounts of guest, empty and bound cages are clearly detected in slow exchange at the NMR timescale. These slow processes prompted us to perform 2D EXSY experiments of *ca*. 1:0.54 mixtures of $\mathbf{1}_{Zn}{}^{CN}$ and *bipy*. Figure S8D shows the non-uniform sampling (NUS) 2D NOESY spectra of a 1:0.54 mixture of $\mathbf{1}_{Zn}{}^{CN}$ and *bipy* in $CDCl_3$ at a mixing time of τ_m = 0.0 s (Figure S8Da) and τ_m = 5.0 s (Figure S8Db). The latter parameter was chosen based on preliminary 1D NOE experiments at different mixing times, and already speaks about the extremely slow nature of the exchange process, in comparison to other supramolecular systems.

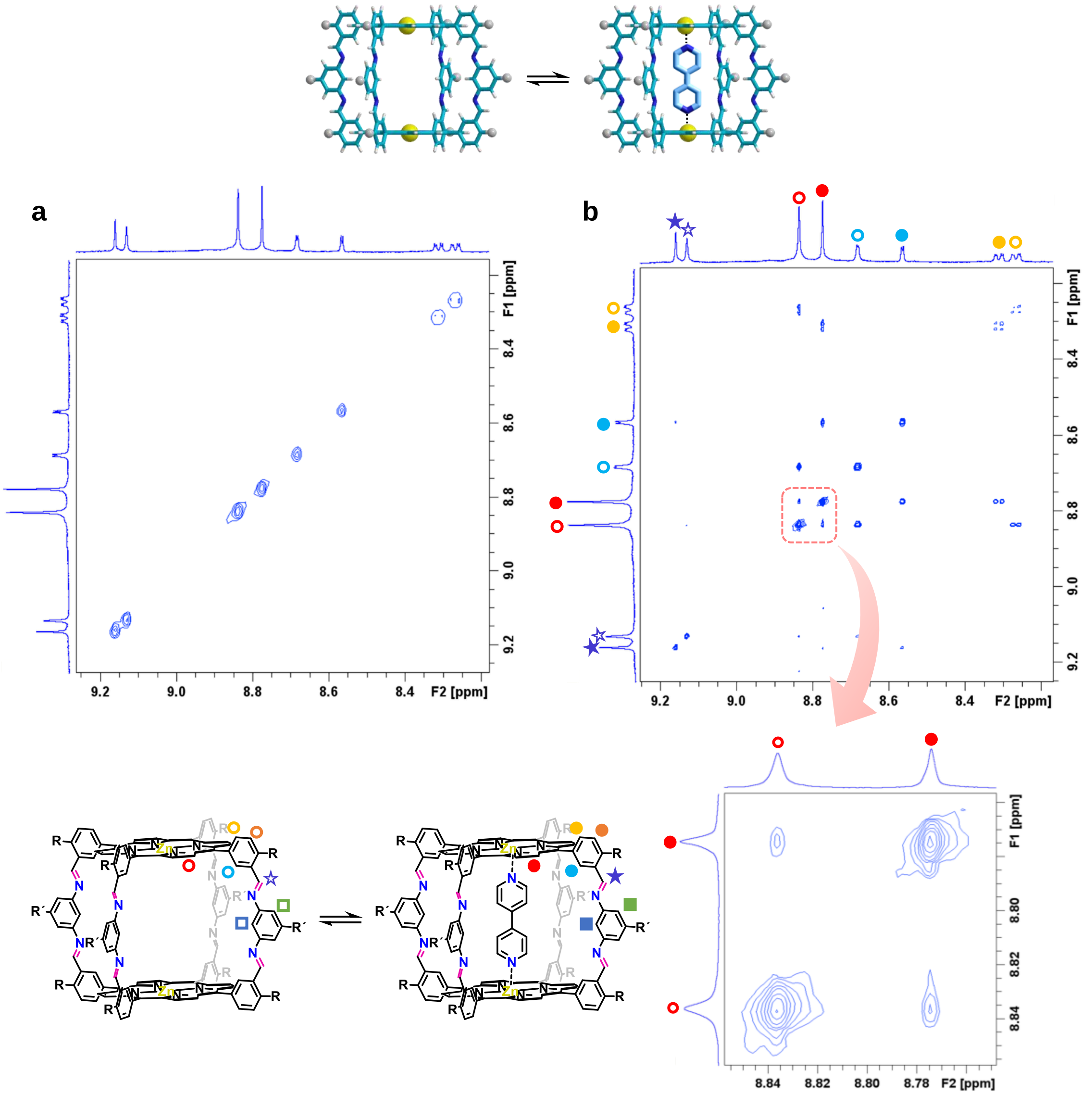

Figure S8D. 2D EXSY spectra of $\mathbf{1}_{Zn}{}^{CN}$ with 0.54 eq. of *bipy* at a constant concentration of $1.0 \cdot 10^{-3}$ M with a mixing time of (**a**) τ_m = 0.0 s and (**b**) τ_m = 5.0 s in $CDCl_3$ at 298 K.

In order to calculate the exchange rate constants, the data at τ_m = 0 and 5000 ms were analyzed in two ways (method a and b), as detailed in Section S7:

The kinetic data obtained by both methods are summarized in the following Table.

	**Method a**	**Method b**
$\boldsymbol{\tau_m}$ ms	***k*** s^{-1}	***k*** s^{-1}
5000	**0.013**	**0.014**

The calculated *k* values from both methods are similar, in between 0.013 and 0.014 s^{-1}. An average value of $\boldsymbol{k_{ex}}$ **= 0.013 ± 0.001** $\mathbf{s^{-1}}$ was taken for the $\mathbf{1_{Zn}}^{\boldsymbol{CN}}$·*bipy* - $\mathbf{1_{Zn}}^{\boldsymbol{CN}}$ exchange rate constant in $CDCl_3$ at 298 K.

S9. Host-Guest Chemistry. Binding of *p*-xylylenediamine (*p*-xyda) to $\mathbf{1}_{\mathbf{Zn}}^{\boldsymbol{NC}}$

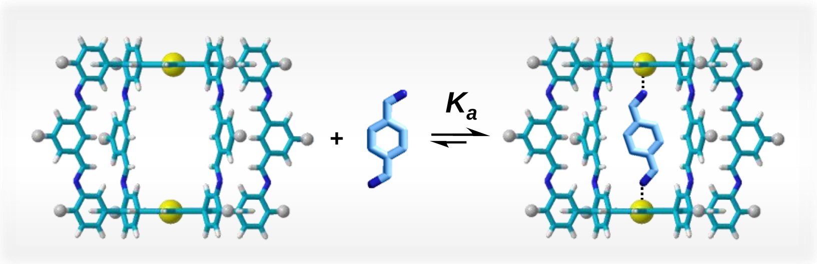

Another guest molecule that belongs to *Group 2* is *p*-xylylenediamine (*p-xyda*), having 2 nitrogen atoms at just the right distance to bind to both Zn^{II} centers in the cavity of $\mathbf{1}_{Zn}^{NC}$ in the *extended* conformation. Binding was evaluated through titration experiments, monitored by ^{1}H NMR in $CDCl_3$ (Figure S9A) and UV-vis in $CHCl_3$ (Figure S9B). In this way, we determined the binding constant (K_a) between $\mathbf{1}_{Zn}^{NC}$ and *p-xyda* in $CHCl_3$, by fitting the binding isotherms to a 1:1 model.

^{1}H NMR Titrations

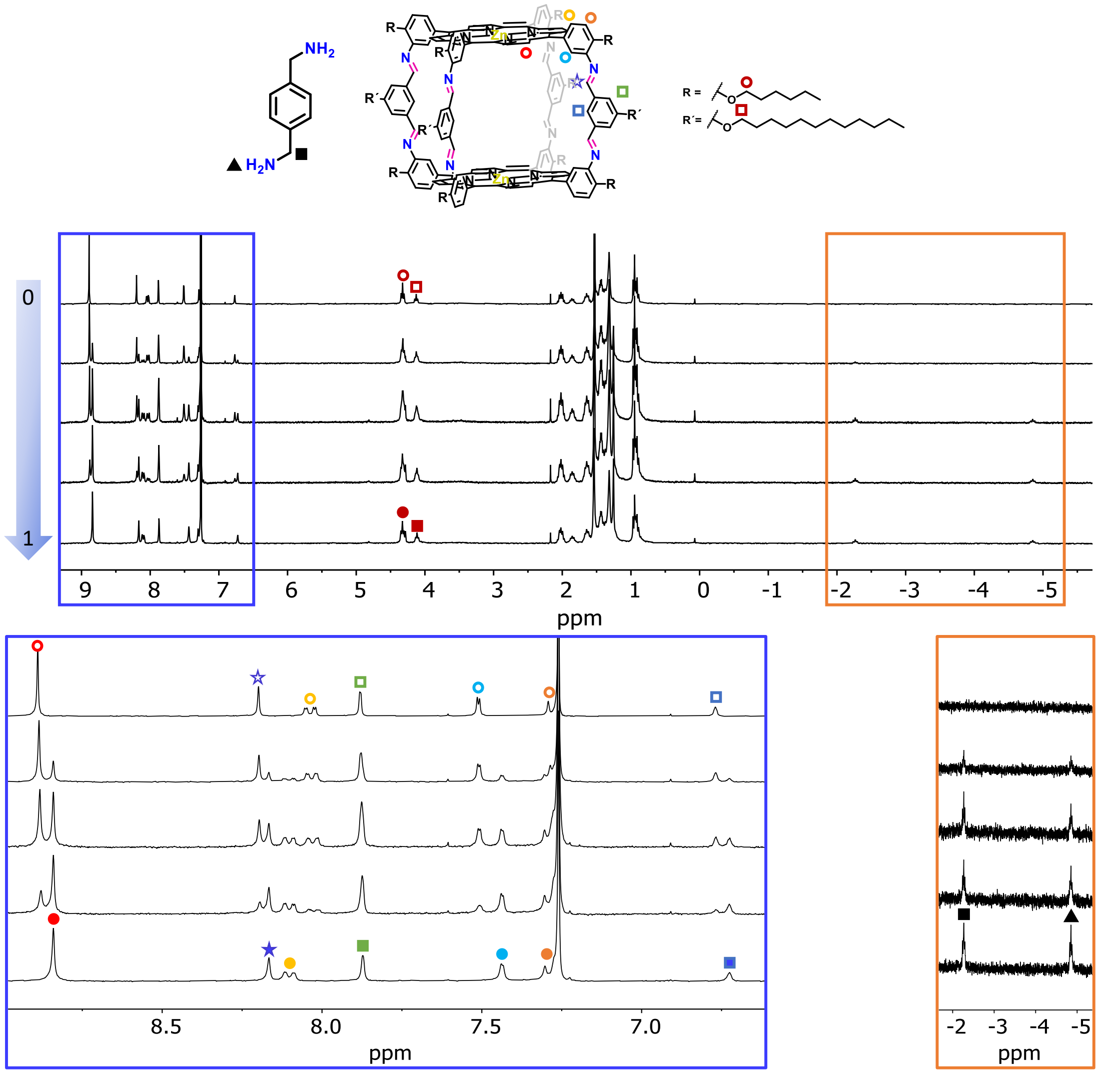

Figure S9A. ^{1}H NMR changes recorded along the titration of $\mathbf{1}_{Zn}^{NC}$ with increasing amounts of *p*-xyda in $CDCl_3$ at 298 K.

UV-vis Titrations

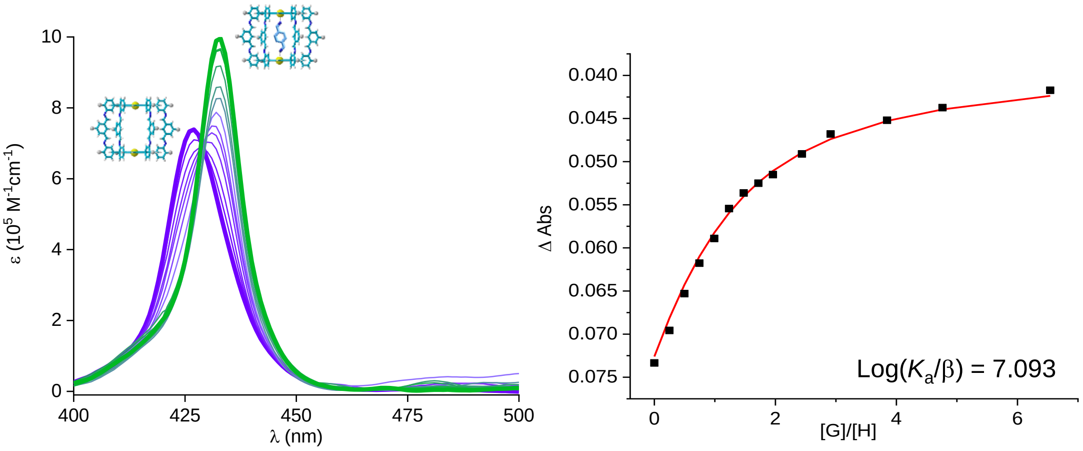

Figure S9B. UV-vis changes recorded along the titration of $\mathbf{1}_{Zn}^{NC}$ at a constant concentration of $1.0 \cdot 10^{-7}$ M with increasing amounts of *p*-xyda in $CHCl_3$ at 298 K. Binding isotherm and fitting to a 1:1 model.

NOESY Experiments

On the other hand, a NOESY spectrum (Figure S9C) confirmed that the $\mathbf{1}_{Zn}^{NC}$ cage maintained the *extended* conformation upon binding to the *p-xyda* guest, since the same cross peaks were observed for the relevant protons as for the empty $\mathbf{1}_{Zn}^{NC}$ cage (please compare with Figure S1C).

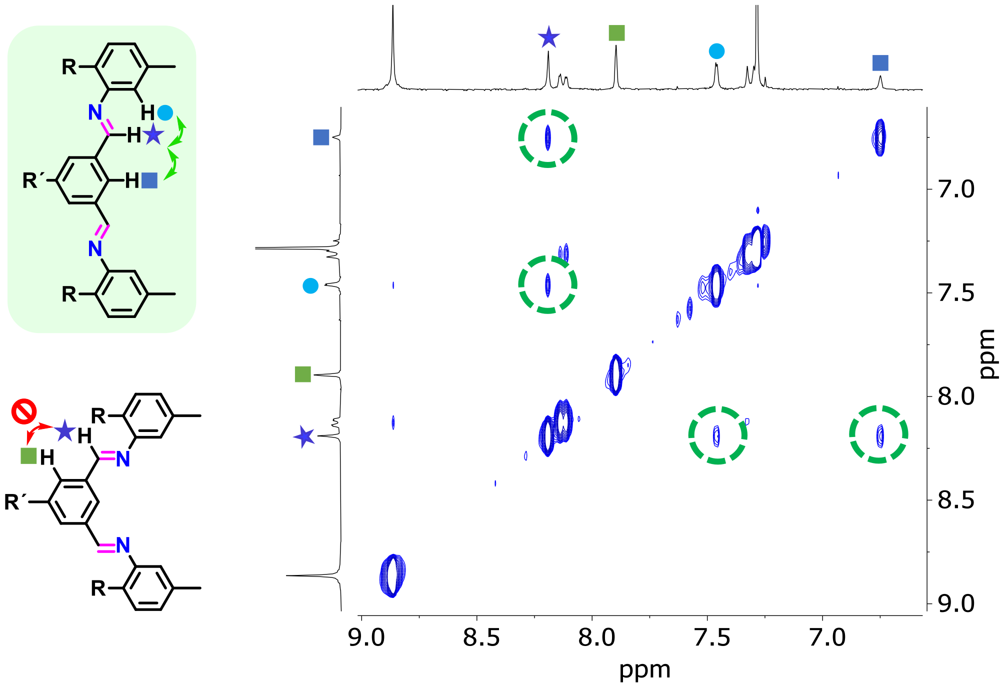

Figure S9C. 2D NOESY spectrum of the 1:1 $\mathbf{1}_{Zn}^{NC}$·*p-xyda* complex in $CDCl_3$, showing NOE cross-peaks that support the prevalence of an *extended* conformation.

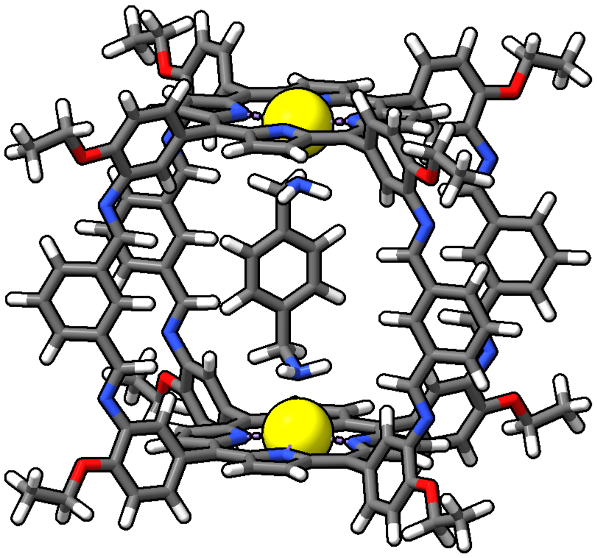

Figure S9D. B3LYP/cc-PVDZ-optimized structures calculated for $\mathbf{1}_{\mathbf{Zn}}{}^{\mathbf{\mathit{NC}}}$·*p-xyda*.

S10. Host-Guest Chemistry. Binding of *m*-xylylenediamine (*m*-xyda) to $\mathbf{1}_{Zn}^{NC}$

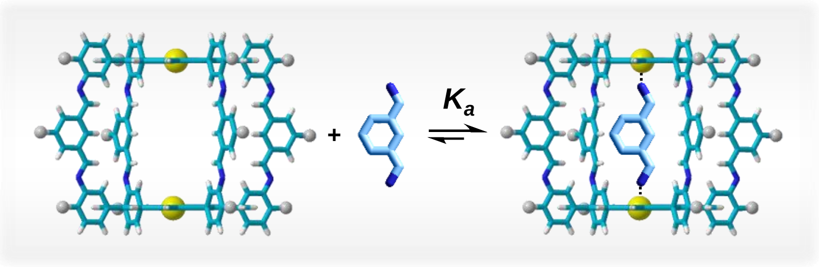

Another guest molecule that belongs to *Group 2* is *m*-xylylenediamine (*m-xyda*), having 2 nitrogen atoms at just the right distance to bind to both Zn^{II} centers in the cavity of $\mathbf{1}_{Zn}^{NC}$ in the *extended* conformation. Binding was evaluated through titration experiments, monitored by ^{1}H NMR (Figure S10A) and UV-vis (Figure S10B). In this way, we determined the binding constant (K_a) between $\mathbf{1}_{Zn}^{NC}$ and *m-xyda* in CHCl$_3$, by fitting the binding isotherms to a 1:1 model.

^{1}H NMR Titrations

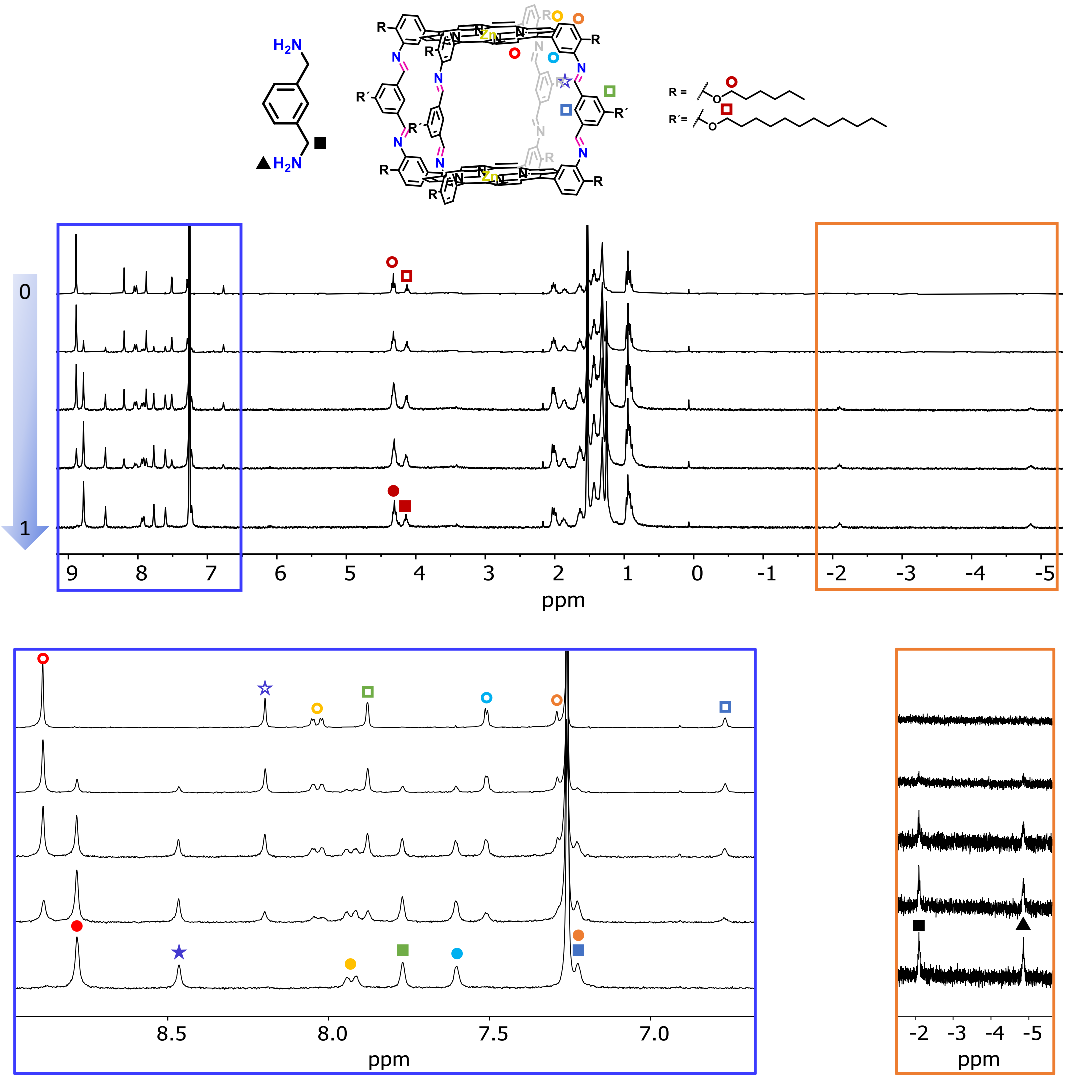

Figure S10A. ^{1}H NMR changes recorded along the titration of $\mathbf{1}_{Zn}^{NC}$ with increasing amounts of *m*-xyda in CDCl$_3$ at 298 K.

UV-vis Titrations

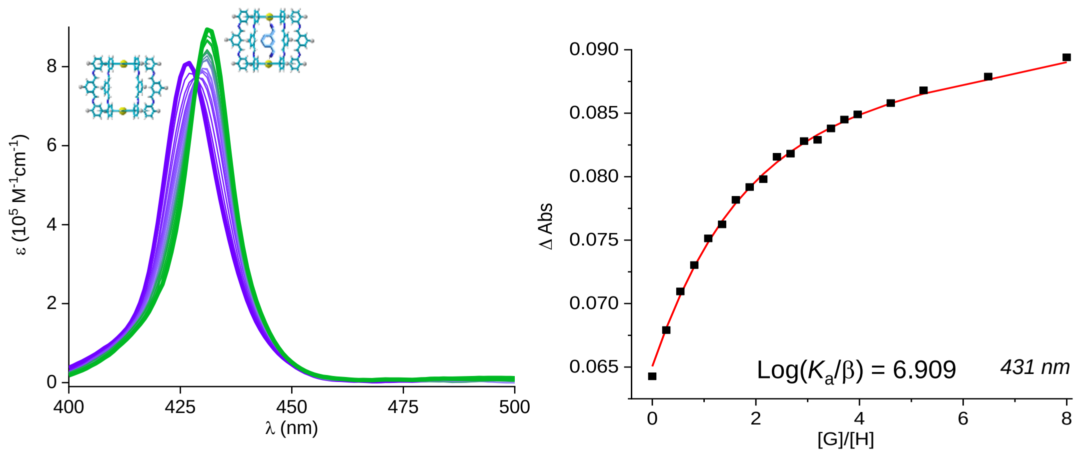

Figure S10B. UV-vis changes recorded along the titration of $\mathbf{1}_{\mathbf{Zn}}^{NC}$ at a constant concentration of 1.0·10^{-7} M with increasing amounts of *m*-xyda in $CHCl_3$ at 298 K. Binding isotherm and fitting to a 1:1 model.

NOESY Experiments

On the other hand, a NOESY spectrum (Figure S10C) confirmed that the $\mathbf{1}_{\mathbf{Zn}}^{NC}$ cage also maintained the *extended* conformation upon binding to the *m*-xyda guest, since the same cross peaks were observed for the relevant protons as for the empty $\mathbf{1}_{\mathbf{Zn}}^{NC}$ cage (please compare with Figure S1C).

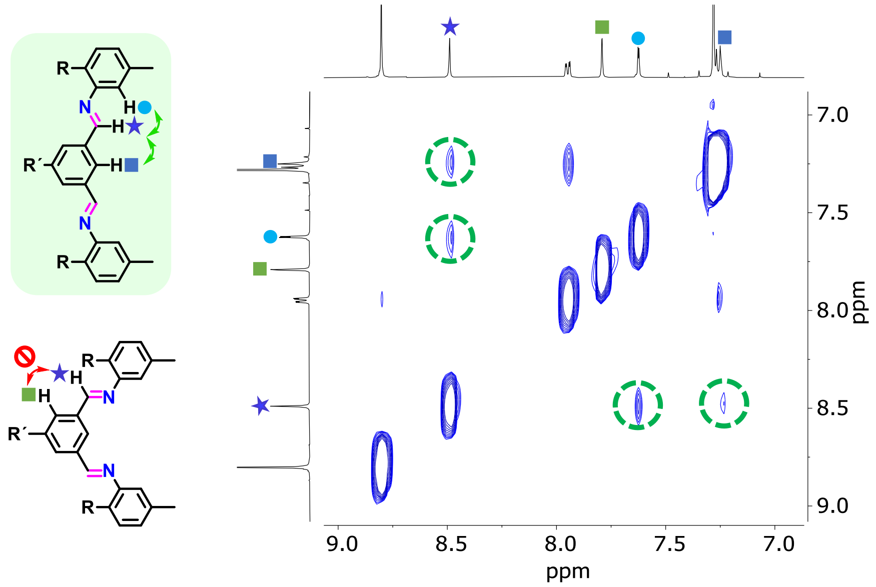

Figure S10C. 2D NOESY spectrum of the $\mathbf{1}_{\mathbf{Zn}}^{NC}$·*m-xyda* (1:1) complex in $CDCl_3$ at 298 K, showing NOE cross-peaks that support the prevalence of an *extended* conformation.

DOSY Experiments

DOSY experiments of 1:1 $\mathbf{1}_{Zn}^{NC}$·*m-xyda* mixture (Figure S10D) revealed host and guest diffusing with the same diffusion coefficient, which is an additional proof of their strong association.

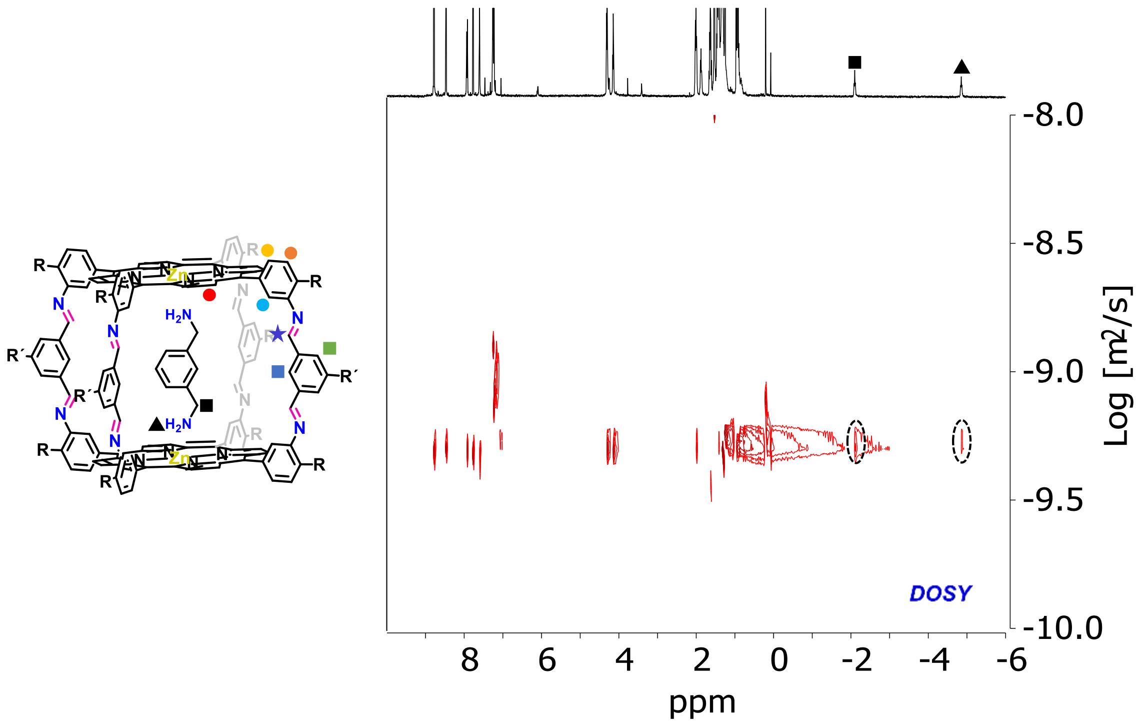

Figure S10D. 2D DOSY spectrum of the $\mathbf{1}_{Zn}^{NC}$·*m-xyda* (1:1) complex in $CDCl_3$ at 298 K, showing the signals of both host and guest at the same diffusion coefficient.

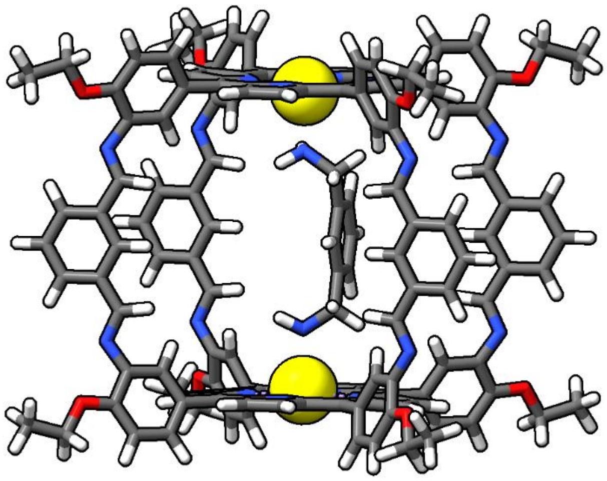

Figure S10E. B3LYP/cc-PVDZ-optimized structures calculated for $\mathbf{1}_{Zn}^{NC}$·*m-xyda*.

S11. Host-Guest Chemistry. Binding of *m*-xylylenediamine (*m*-xyda) to $\mathbf{1}_{Zn}^{CN}$

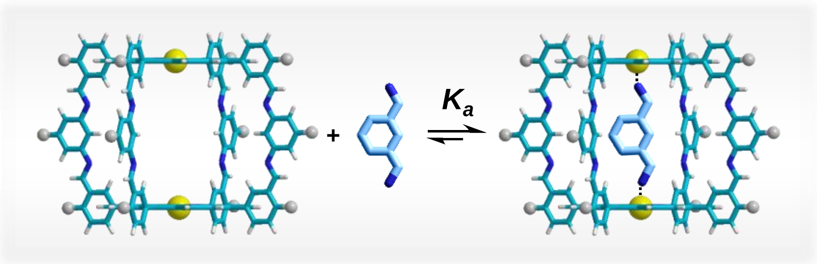

Another guest molecule that belongs to *Group 2* is *m*-xylylenediamine (*m*-xyda), having 2 nitrogen atoms at just the right distance to bind to both Zn^{II} centers in the cavity of $\mathbf{1}_{Zn}^{CN}$ in the *extended* conformation. Binding was evaluated through titration experiments, monitored by ^{1}H NMR (Figure S11A) and UV-vis (Figure S11B). In this way, we determined the binding constant (K_a) between $\mathbf{1}_{Zn}^{CN}$ and *m*-xyda in $CHCl_3$, by fitting the binding isotherms to a 1:1 model.

^{1}H NMR Titrations

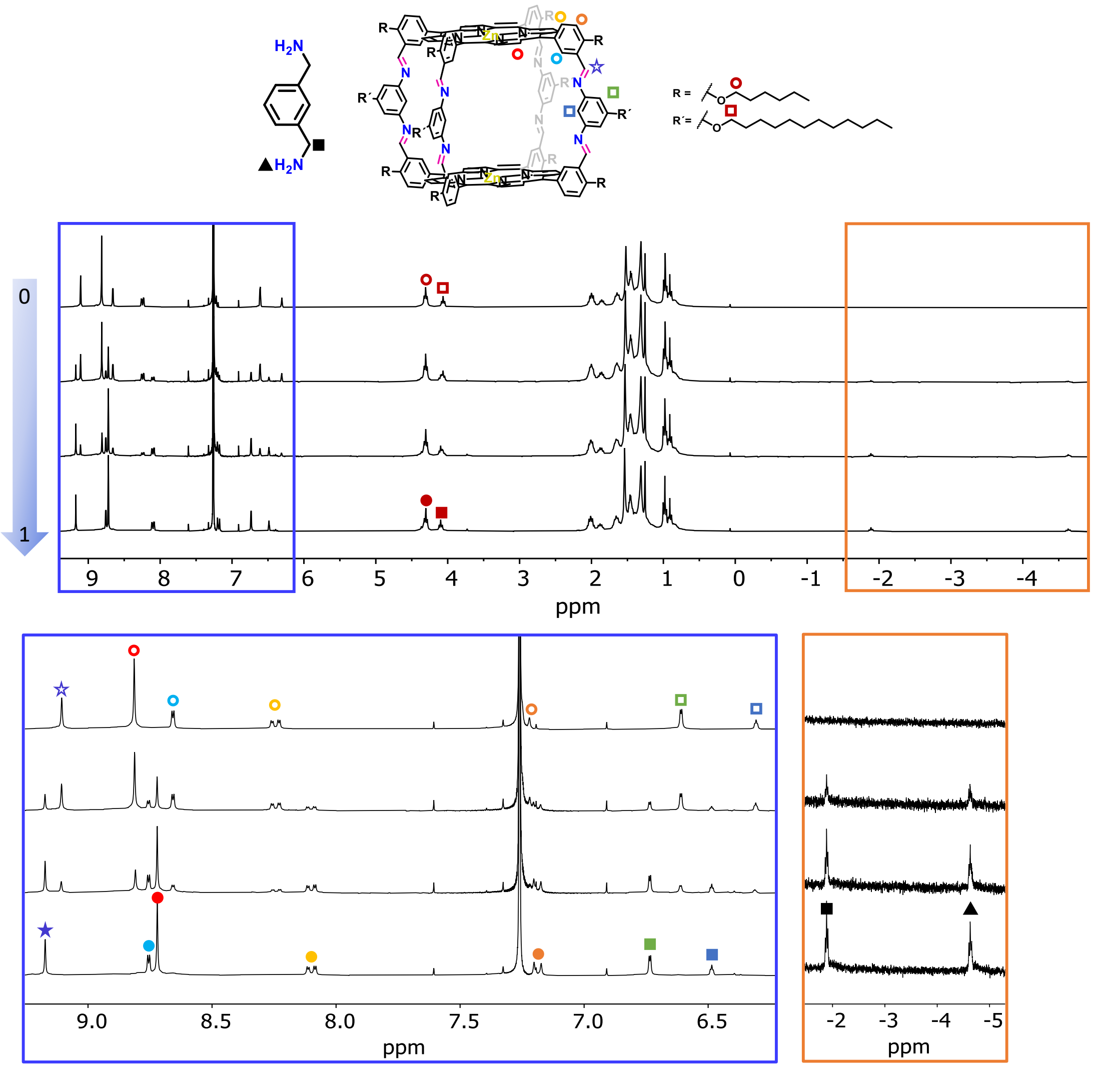

Figure S11A. ^{1}H NMR changes recorded along the titration of $\mathbf{1}_{Zn}^{CN}$ with increasing amounts of *m*-xyda in $CDCl_3$ at 298 K.

UV-vis Titrations

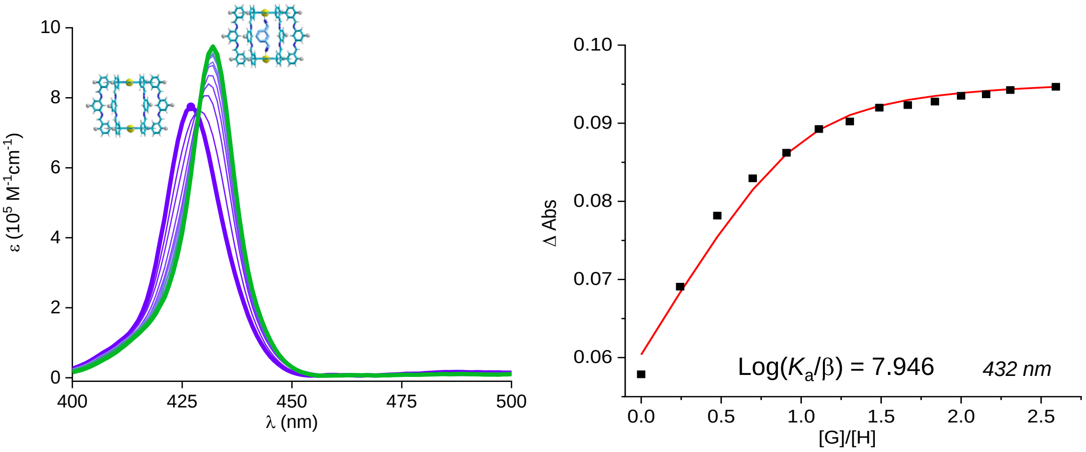

Figure S11B. UV-vis changes recorded along the titration of $\mathbf{1}_{Zn}^{CN}$ at a constant concentration of $1.0 \cdot 10^{-7}$ M with increasing amounts of *m*-xyda in $CHCl_3$ at 298 K. Binding isotherm and fitting to a 1:1 model.

S12. Calculation of *EM* from K_a and K_{ref}

Binding of dinitrogenated guest molecules to both Zn^{II} metal centers within the cages is affected by chelate cooperativity. In order to calculate the associated effective molarity (*EM*) values, we need to take into account statistical factors.

Providing that the intrinsic or microscopic equilibrium constant for the binding of a nitrogen atom (either from a pyridine or an amine derivative) to a Zn^{II} metal center in a porphyrin is represented as $\boldsymbol{K}$, then the association constant ($\boldsymbol{K_{ref}}$) for the binding of this nitrogenated guest (a pyridine derivative, like *py*, *bipy* or *naphy*, for instance) to a reference (Zn^{II})porphyrin, as shown in Figure 12A, is statistically corrected as $\boldsymbol{K_{ref}} = 2\cdot\boldsymbol{K}$, since there are two options for association in the forward process (the two *P* faces) and just one for dissociation in the reverse process.

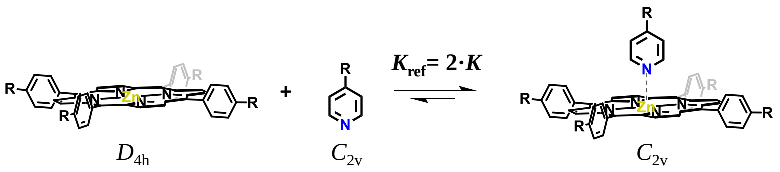

Figure S12A. Scheme for the determination of microscopic equilibrium constant ($\boldsymbol{K}$) using a reference porphyrin and a pyridine derivative as monotopic ligand.

In the case of the binding of a ditopic guest (bipyridine, for instance) to both metal centres in the cage, there are two Zn···N interactions being formed in the forward process and two Zn···N interactions being destroyed in the reverse process, so statistical factors affecting the association constant ($\boldsymbol{K_a}$) equal 1. However, the second binding interaction is intramolecular, and must therefore be corrected by the *EM* factor. Thus, in this case $\boldsymbol{K_a} = \boldsymbol{K}^2\cdot EM$ or, in other words, $\boldsymbol{K_a} = \boldsymbol{K_{ref}}^2\cdot EM/4$. The magnitudes $\boldsymbol{K_a}$ and $\boldsymbol{K_{ref}}$ are determined experimentally through titration experiments (see Table 1 in the main text and Figures S5A-F), while *EM* is hence calculated as: $EM = 4\ \boldsymbol{K_a}/\boldsymbol{K_{ref}}^2$.

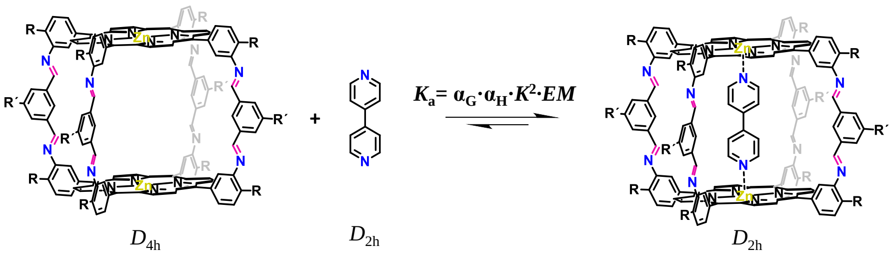

Figure S12B. Scheme for the determination of *EM* using previously determined $\boldsymbol{K_a}$ and $\boldsymbol{K_{ref}}$.

As shown in Figure S12B, other cooperativity factors (α) associated to electronic effects or intramolecular interactions present in the guest (α_G) or in the host (α_H) can also affect K_a. For example, binding of the first pyridine moiety in *bipy* can influence electronically the binding strength of the second pyridine moiety. However, these cooperativity factors, which may be neglected for the host but that can be significant for guests like *naphy*, were not independently measured in this work and, consequently, are integrated in the calculated *EM*s.

S13. Calculation of *EM* from competition experiments

In order to corroborate these *EM* values through a different approach, we conducted competition experiments between ditopic bipyridine (*bipy*) and monotopic pyridine (*py*) guests, whereby increasing amounts of pyridine-D_5 are added to dissociate the $\mathbf{1}_{Zn}^{NC}$/$\mathbf{1}_{Zn}^{CN}$ cage·*bipy* complexes in $CDCl_3$. In these experiments, we are essentially making the intermolecular and intramolecular versions of the same interaction to compete, and the equilibrium constant of this competition (K_C) can be directly related to *EM* as: $K_C = 4 \cdot EM$, as shown in Figures S13A and S13C for $\mathbf{1}_{Zn}^{NC}$ and $\mathbf{1}_{Zn}^{CN}$, respectively.

The outcome of these experiments is displayed in Figures S13B and S13D for $\mathbf{1}_{Zn}^{NC}$ and $\mathbf{1}_{Zn}^{CN}$, respectively. In general, the addition of pyridine-D_5 only results in very minor chemical shifts of the $\mathbf{1}_{Zn}^{NC}$/$\mathbf{1}_{Zn}^{CN}$ proton signals. The only signals that are significantly affected are those of the encapsulated *bipy* guests, which broaden and decrease in intensity as more *py* competitor is added. No signals for the expeled *bipy* molecules outside the cage were detected, likely due to a fast exchange with *py* molecules to coordinate the external Zn^{II} sites. Thus, the key proton signals that were considered for the quantitative analysis of the titration data were the linker protons pointing to the inner cavity (blue square at around 6.5 ppm), which represent the total concentration of cages present in equilibrium, and the complexed *bipy* protons (black triangle at around 4.7 ppm), which account for the concentration of $\mathbf{1}_{Zn}^{NC}$/$\mathbf{1}_{Zn}^{CN}$·*bipy* complex. The relative integration of each signal, as indicated in Figures S13B and S13D, supplies the concentration of $\mathbf{1}_{Zn}^{NC}$/$\mathbf{1}_{Zn}^{CN}$·*bipy* complex. K_C could be in this way calculated, since the total concentration of cage is known and the concentration of *py* can be approximated to the actual pyridine-D_5 added. Due to the mentioned broadening, ^{1}H NMR signal integration became unreliable above *ca.* 40 eqs. ($\mathbf{1}_{Zn}^{NC}$) and 200 eqs ($\mathbf{1}_{Zn}^{CN}$) of added pyridine-D_5, titration points in which the $\mathbf{1}_{Zn}^{NC}$/$\mathbf{1}_{Zn}^{CN}$·*bipy* complexes are still very abundant (>75%). Still, we could obtain several data points to afford an average K_C, from which *EM* was derived using the equations shown in Figures S13A and S13C.

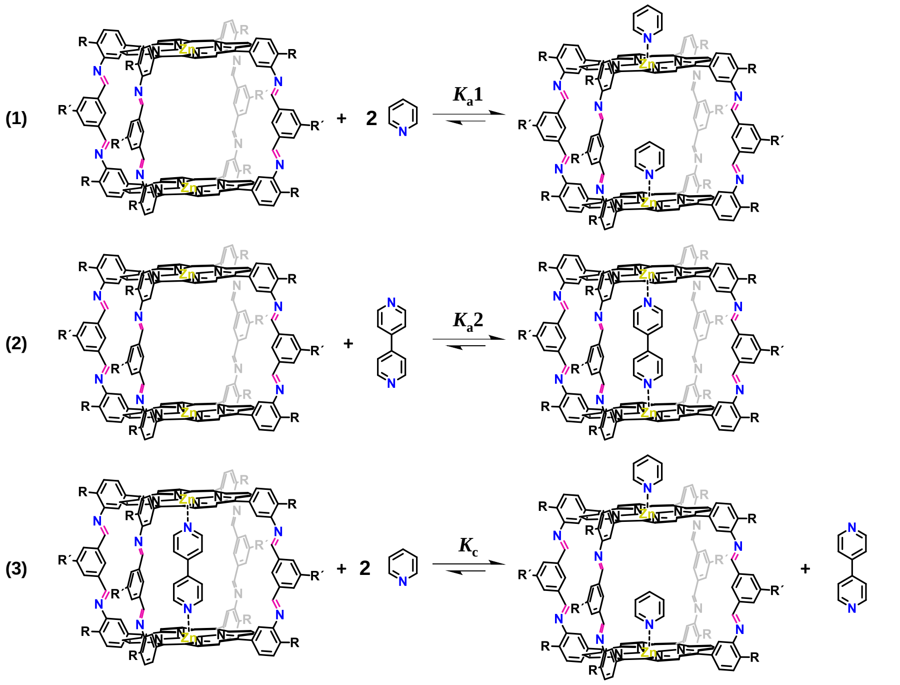

$$K_c = (1) - (2) = \frac{K_a1}{K_a2} = \frac{[\mathbf{1}_{Zn}^{NC}\cdot py]\cdot[bipy]}{[\mathbf{1}_{Zn}^{NC}\cdot bipy]\cdot[py]^{2}} = \frac{4}{EM} \qquad K_c = 0.062$$

Figure S13A. Scheme for the determination of *EM* from a competition experiment where *py* can displace *bipy* in the cage $\mathbf{1}_{Zn}{}^{NC}$.

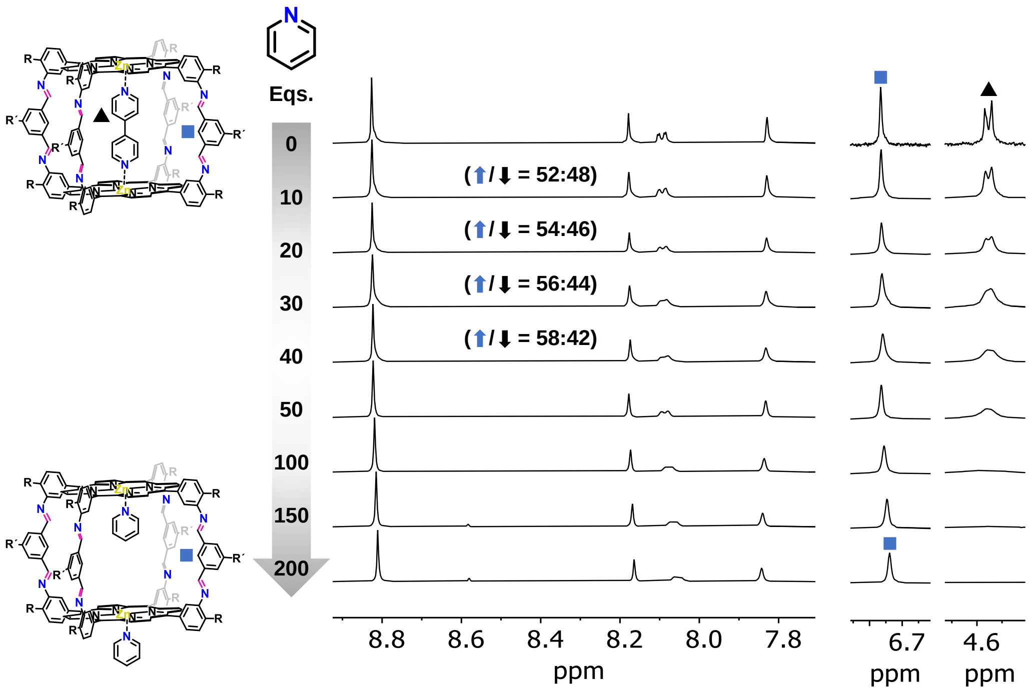

Figure S13B. ^{1}H NMR spectra acquired in the competition experiment between *bipy* and *py* for the cavity of $\mathbf{1}_{Zn}{}^{NC}$. Up to 200 eqs of *pyridine-D*$_5$ were added to a 1:1 complex of $\mathbf{1}_{Zn}{}^{NC}\cdot$*bipy* at a constant concentration of $1.0\cdot10^{-3}$ M, in $CDCl_3$ at 298 K.

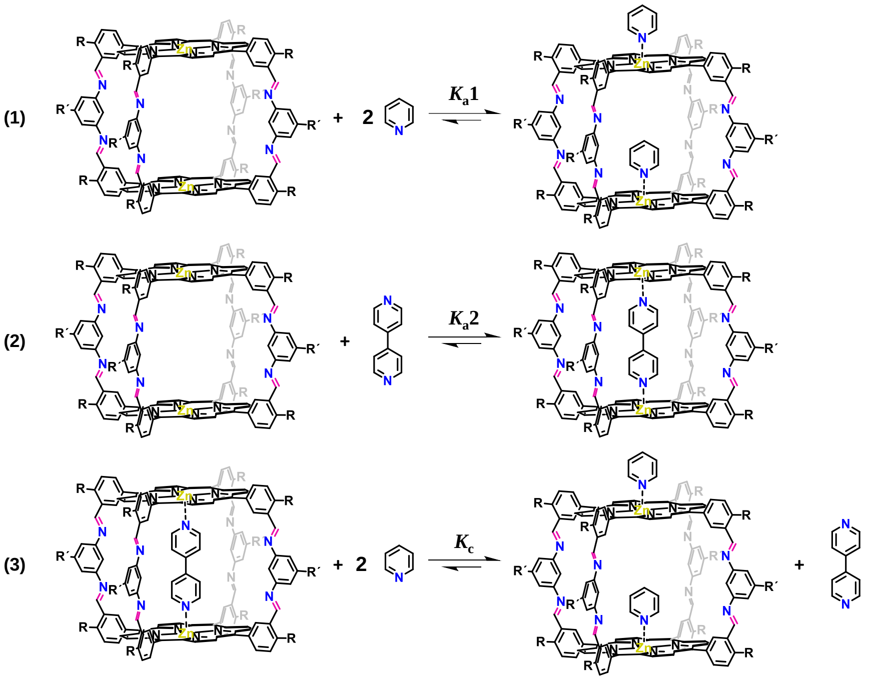

$$K_c = (1) - (2) = \frac{K_a 1}{K_a 2} = \frac{[1_{Zn}{}^{CN}\cdot py]\cdot[bipy]}{[1_{Zn}{}^{CN}\cdot bipy]\cdot[py]^2} = \frac{4}{EM} \qquad K_c = 0.006$$

Figure S13C. Scheme for the determination of *EM* from a competition experiment where pyridine can displace *bipy* in the cage $\mathbf{1}_{Zn}{}^{CN}$.

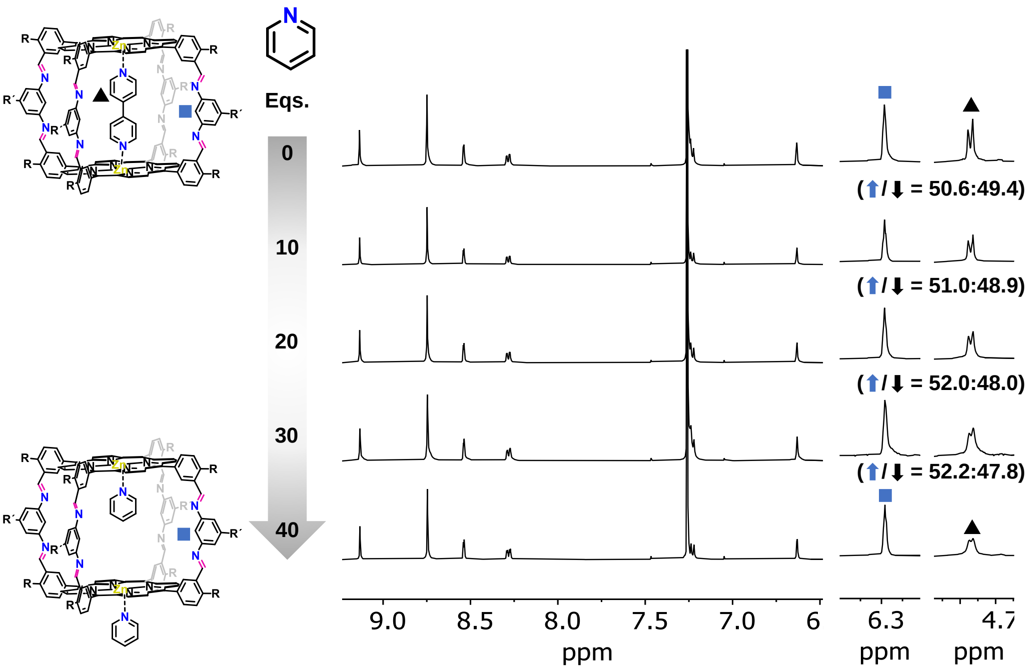

Figure S13D. ^{1}H NMR spectra acquired in the competition experiment between *bipy* and *py* for the cavity of $\mathbf{1}_{Zn}{}^{CN}$. Up to 200 eqs of *pyridine-D*$_5$ were added (only up to 40 eqs are shown here) to a 1:1 complex of $\mathbf{1}_{Zn}{}^{CN}\cdot$*bipy* at a constant concentration of $1.0\cdot10^{-3}$ M, in $CDCl_3$ at 298 K.

S14. Host-Guest Chemistry. Binding of 4-(methylamino)pyridine (mapy) to 1_{Zn}^{NC}

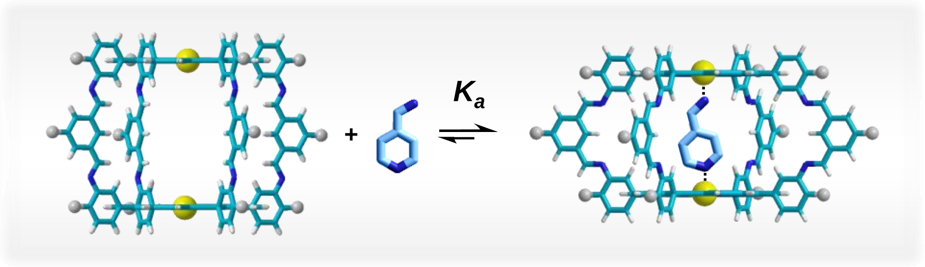

Binding of guest molecules in *Group 3*, like 4-(methylamino)pyridine (*mapy*), having 2 nitrogen atoms at the right distance to bind to both Zn^{II} centers in the cavity of $\mathbf{1}_{Zn}^{NC}$ in the *compact* conformation, was first evaluated through titration experiments in chloroform and in toluene monitored by ^{1}H NMR (Figures S14A-1,2) and UV-vis (Figure S14B).

^{1}H NMR Titrations

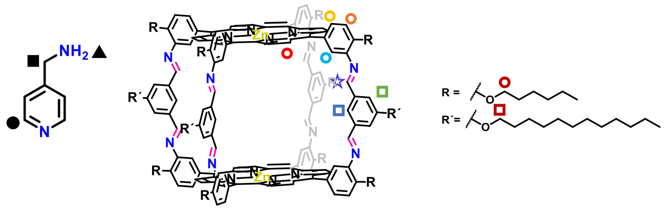

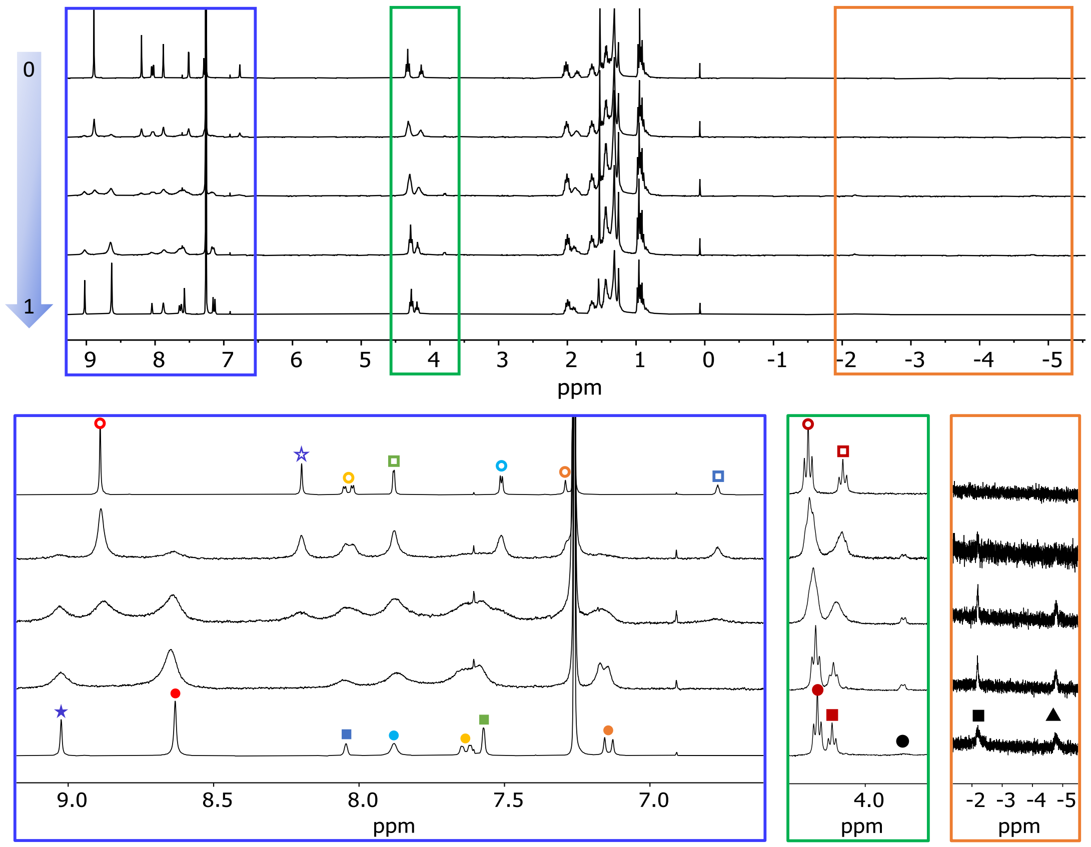

Figure S14A-1. ^{1}H NMR changes recorded along the titration of $\mathbf{1}_{Zn}^{NC}$ with increasing amounts of *mapy* in $CDCl_3$ at 298 K.

The slower exchange attained in toluene-D_8 allowed us to additionally observe the desymmetrization of the cage caused by binding of the non-symmetric ligand. Two set of signals were observed for $\mathbf{1}_{Zn}{}^{NC}$ that corresponded to the two halves of the cage: the one bound to the amino group and the one bound to the pyridine group (Figure S14A-2).

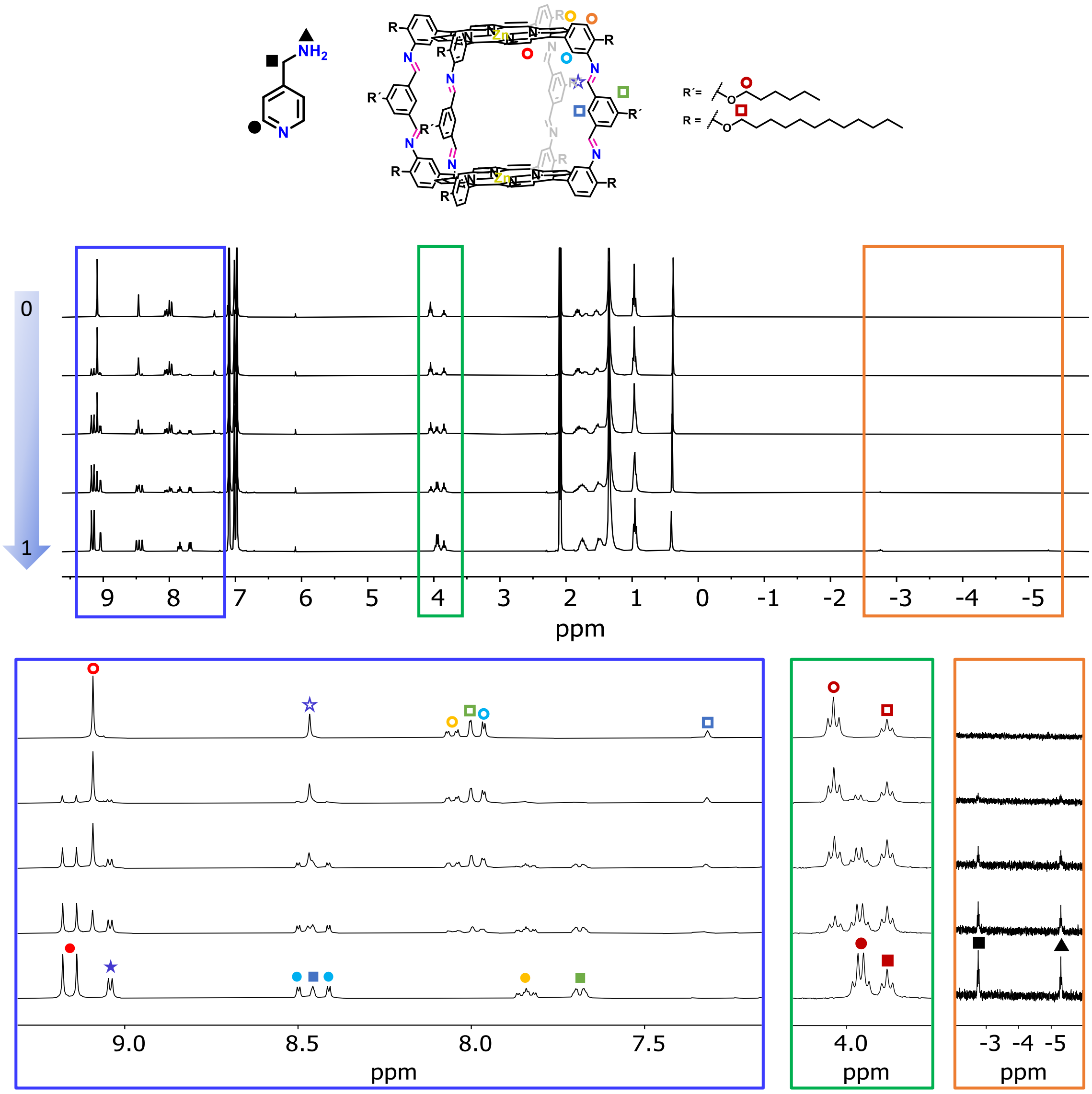

Figure S14A-2. ^{1}H NMR changes recorded along the titration of $\mathbf{1}_{Zn}{}^{NC}$ with increasing amounts of *mapy* in toluene-D_8 at 298 K.

UV-vis Titrations

UV-vis titrations allowed us to determine the binding constant (K_a) between **1**$_{Zn}^{NC}$ and *mapy* in $CHCl_3$, by fitting the binding isotherms to a 1:1 model.

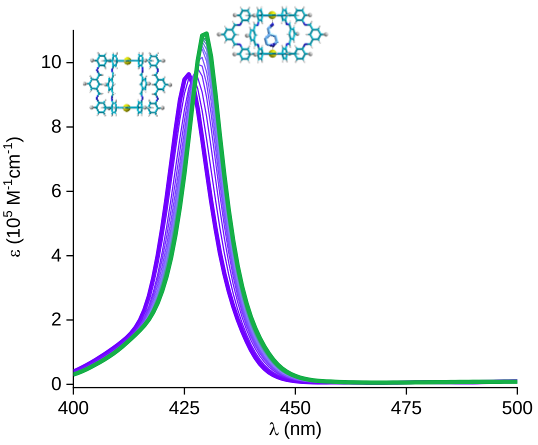

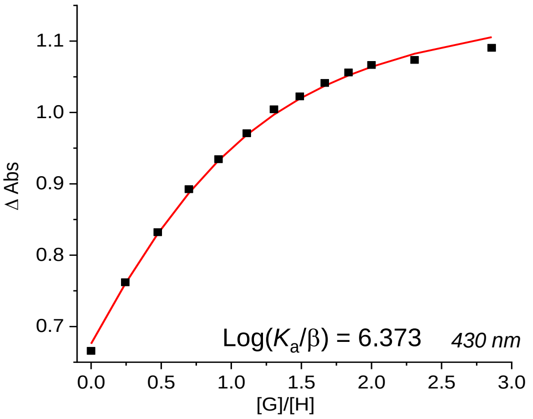

Figure S14B. UV-vis changes recorded along the titration of **1**$_{Zn}^{NC}$ at a constant concentration of $1.0 \cdot 10^{-6}$ M with increasing amounts of *mapy* $CHCl_3$ at 298 K.

NOESY Experiments

On the other hand, and in contrast to the empty cages (please compare with Figure S1C) or the cages bound to *Group 2* guests (please compare with Figure S7C), the NOESY spectrum of $\mathbf{1}_{Zn}^{NC}$ upon binding to the *mapy* guest in $CDCl_3$ (Figure S14C) displayed now cross-peaks that are compatible with a *compact* conformation (marked in blue), although the cross peaks assigned to the *extended* conformation were still observed (marked in green). We believe this is due to the coexistence of both conformations in fast exchange.

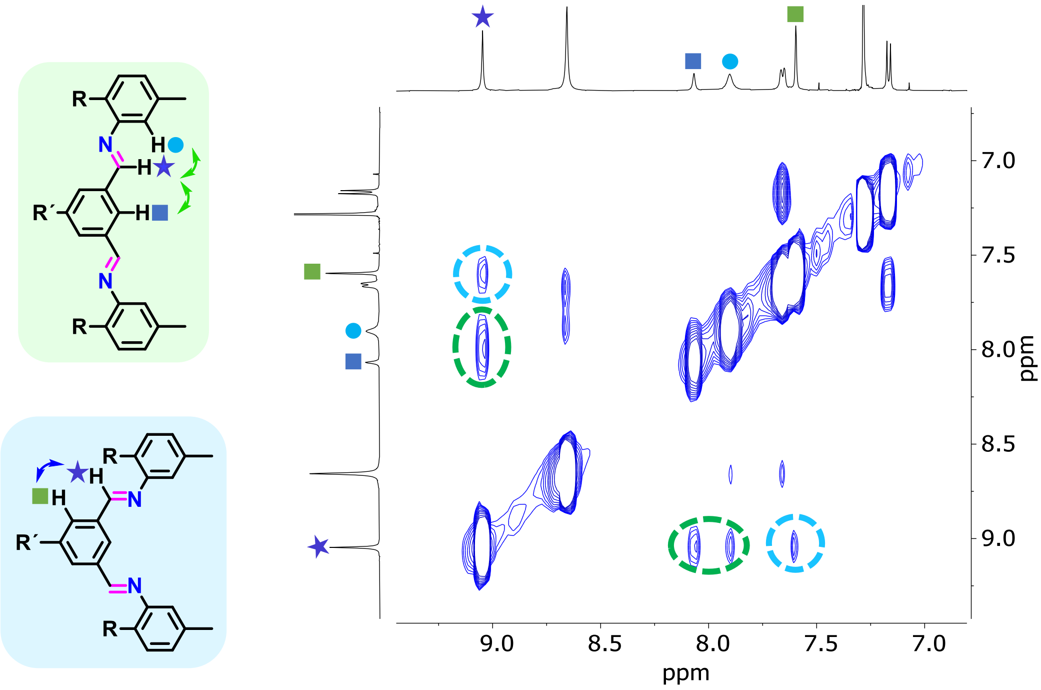

Figure S14C. 2D NOESY spectrum of $\mathbf{1}_{Zn}^{NC}$·*mapy* (1:1) complex, showing ^1H-^1H cross-peaks that are in agreement with the coexistence of both the *extended* (marked in green) and *compact* (marked in blue) conformations in $CDCl_3$ at 298 K.

DOSY Experiments

DOSY experiments of 1:1 **1**$_{Zn}^{NC}$·*mapy* mixture in $CDCl_3$ (Figure S14D) revealed host and guest diffusing with the same diffusion coefficient, which is an additional proof of their strong association.

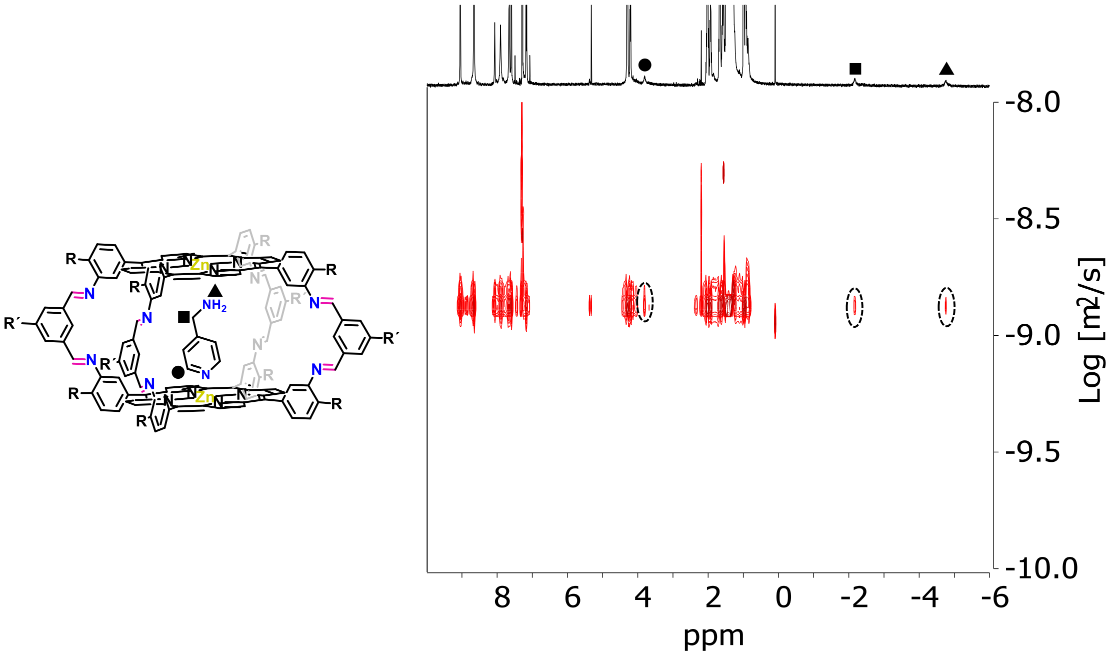

Figure S14D. 2D DOSY spectrum of 1:1 **1**$_{Zn}^{NC}$·*mapy* complex in $CDCl_3$ at 298 K, showing the signals of both host and guest at the same diffusion coefficient.

Temperature-dependent experiments at different Host:Guest ratios

^{1}H NMR experiments as a function of the temperature were carried out at two different $\mathbf{1}_{Zn}^{NC}$:*mapy* ratios (excess of $\mathbf{1}_{Zn}^{NC}$ (Figure S14E-1) or excess of *mapy* (Figure S14E-2)) in order to see the effect on the guest exchange dynamics. Upon decreasing the temperature, the exchange was slowed down in the NMR timescale. Additionally, just like in toluene-D$_8$ (see Figure S14A-2) we could observe the desymmetrization of the cage caused by binding of the non-symmetric ligand. Two set of signals were observed at low temperatures for $\mathbf{1}_{Zn}^{NC}$ that corresponded to the two halves of the cage: the one bound to the amino group and the one bound to the pyridine group.

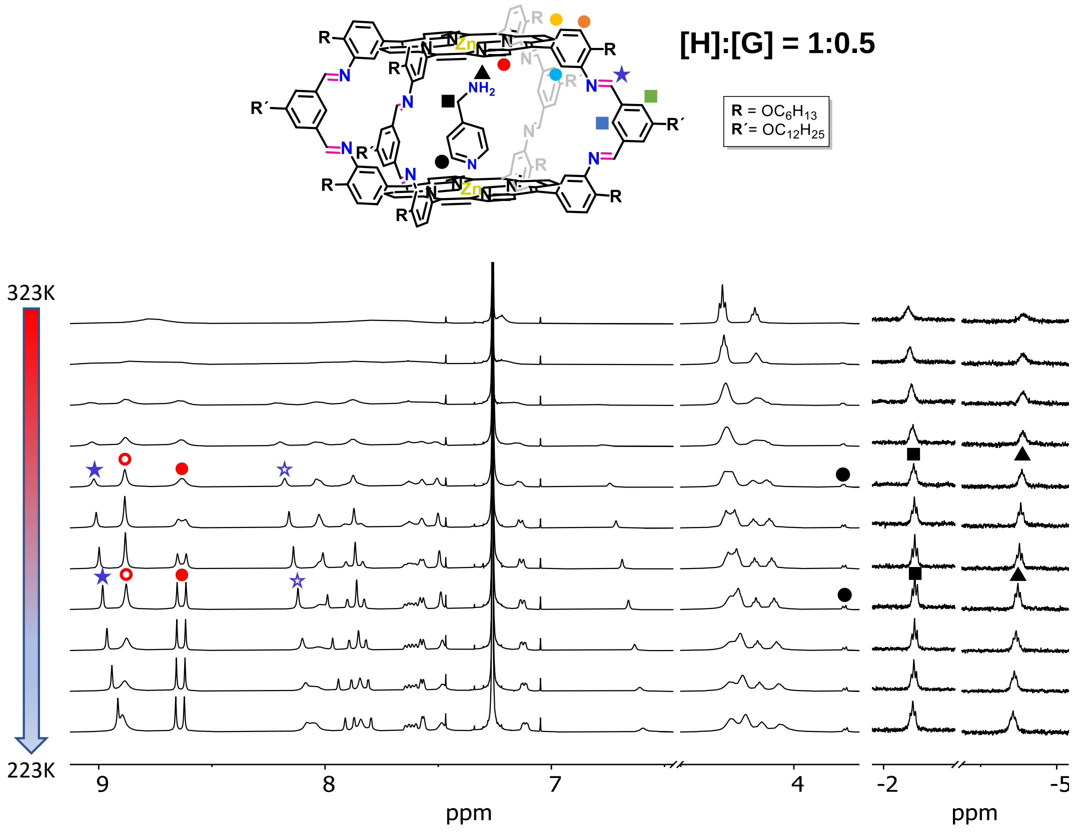

Figure S14E-1. ^{1}H NMR spectra of $\mathbf{1}_{Zn}^{NC}$ as a function of temperature in the presence of **0.5 eqs. of *mapy*** at a constant concentration of 1.0·10^{-3} M in CDCl$_3$**.** Two set of signals corresponding to the occupied (filled circles) and unoccupied (empty circles) $\mathbf{1}_{Zn}^{NC}$ cage are observed.

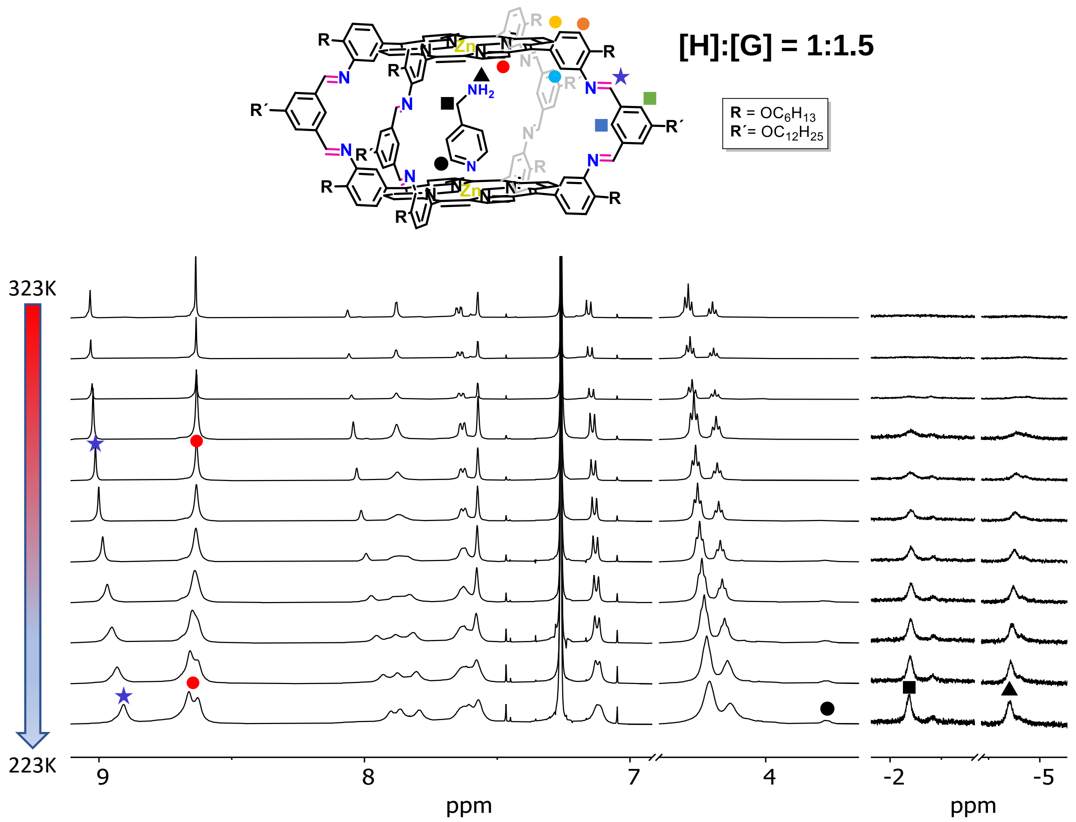

Figure S14E-2. ^{1}H NMR spectra of $\mathbf{1}_{\mathrm{Zn}}^{\boldsymbol{NC}}$ as a function of temperature in the presence of **1.5 eqs. of *mapy*** at a constant concentration of $1.0{\cdot}10^{-3}$ M in $CDCl_3$. At low temperatures the desymmetrization of the $\mathbf{1}_{\mathrm{Zn}}^{\boldsymbol{NC}}$ cage is noticed as a double set of signals.

S15. Host-Guest Chemistry. Binding of 4-(methylamino)pyridine (mapy) to 1_{Zn}^{CN}

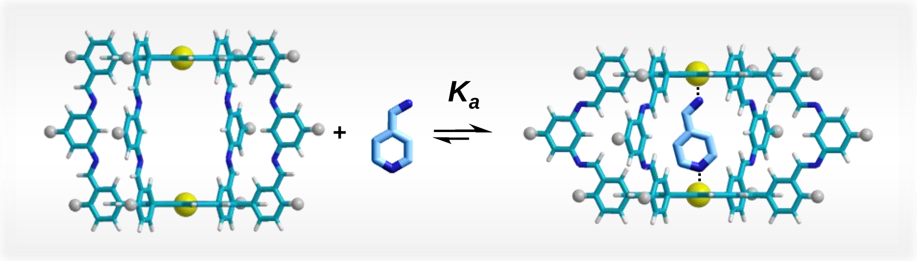

Binding of guest molecules in *Group 3*, like 4-(methylamino)pyridine (*mapy*), having 2 nitrogen atoms at the right distance to bind to both Zn^{II} centers in the cavity of $\mathbf{1}_{Zn}^{CN}$ in the *compact* conformation, was first evaluated through titration experiments in chloroform and in toluene monitored by ^{1}H NMR (Figures S15A-1,2) and UV-vis (Figure S15B).

^{1}H NMR Titrations

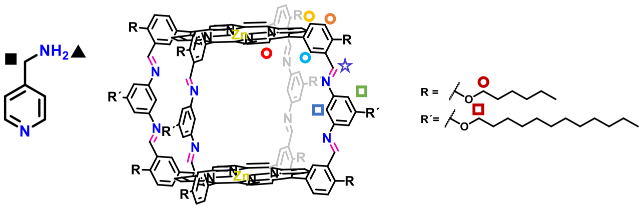

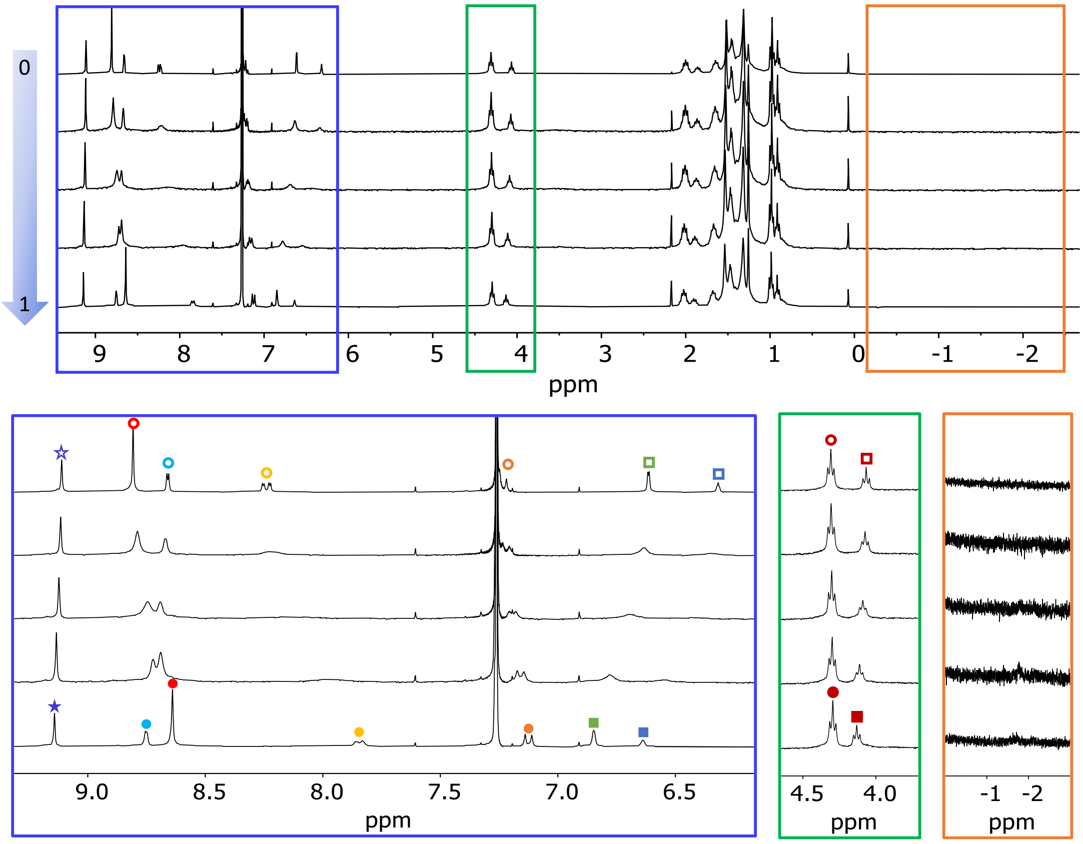

Figure S15A-1. ^{1}H NMR changes recorded along the titration of $\mathbf{1}_{Zn}^{CN}$ with increasing amounts of *mapy* in $CDCl_3$ at 298 K.

In contrast to any other dinitrogen ligand that fit within the rigid **1**$_{Zn}$^{NC} or **1**$_{Zn}$^{CN} cavities (*Groups 2 and 3*), which displayed slow exchange in the ^{1}H NMR scale, the **1**$_{Zn}$^{CN}·*mapy* complex exhibited fast NMR exchange in $CDCl_3$ (Figure S15A-1). This is not entirely surprising, since the **1**$_{Zn}$^{NC}·*mapy* complex already presented broad signals in this solvent (see Figure S14A-1) that suggested that exchange was fast, in comparison to Group 2 guests. As for the **1**$_{Zn}$^{NC}·*mapy* complex, a solvent change to toluene-D_8 resulted in a much slower exchange and allowed us to additionally observe the desymmetrization of the cage caused by binding of the non-symmetric ligand. Two set of signals were observed for **1**$_{Zn}$^{CN} that corresponded to the two halves of the cage: the one bound to the amino group and the one bound to the pyridine group (Figure S15A-2).

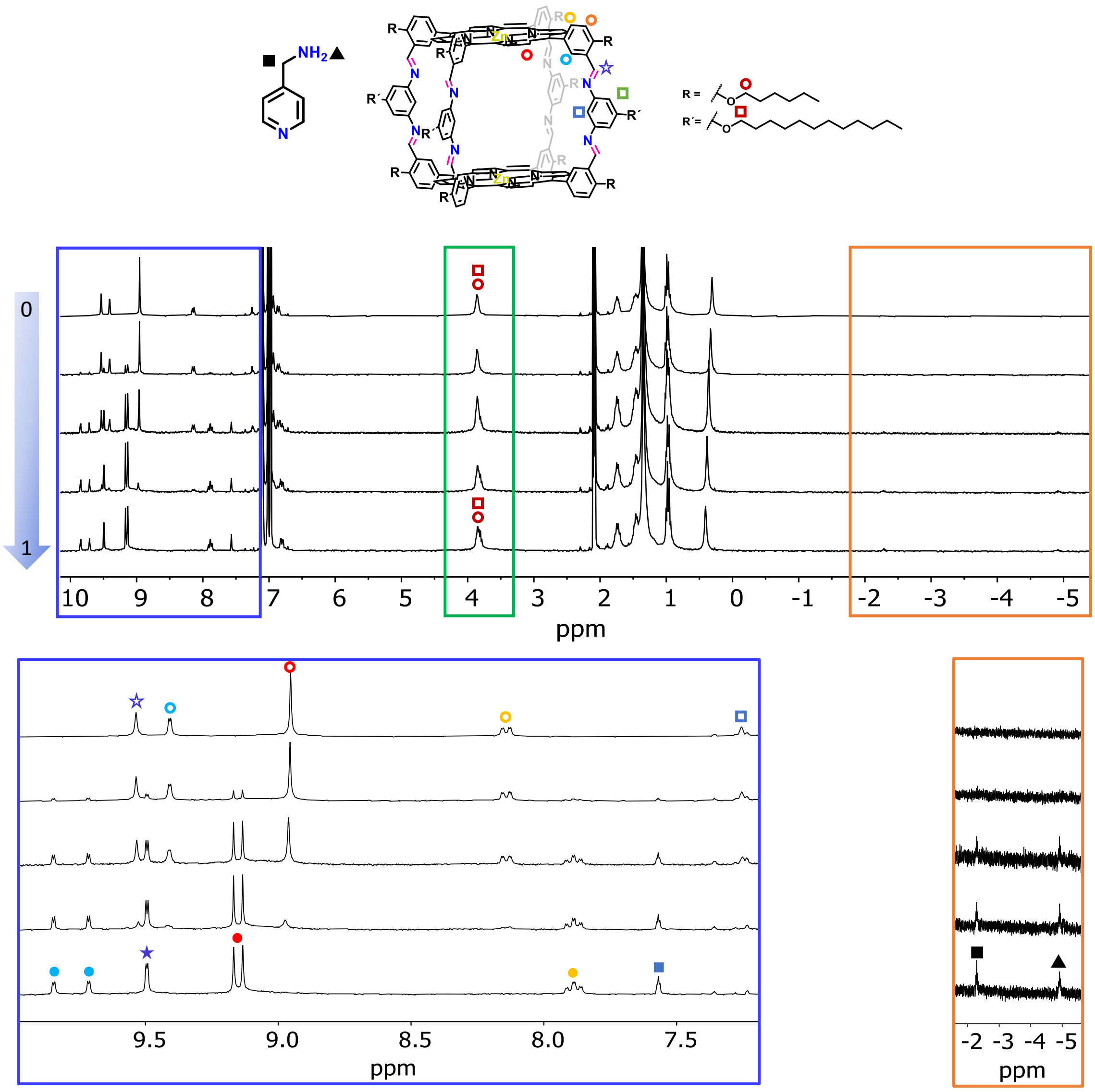

Figure S15A-2. ^{1}H NMR changes recorded along the titration of **1**$_{Zn}$^{CN} with increasing amounts of *mapy* in toluene-D_8 at 298 K.

UV-vis Titrations

UV-vis titrations allowed us to determine the binding constant (K_a) between $\mathbf{1}_{Zn}^{CN}$ and *mapy* in $CHCl_3$, by fitting the binding isotherms to a 1:1 model.

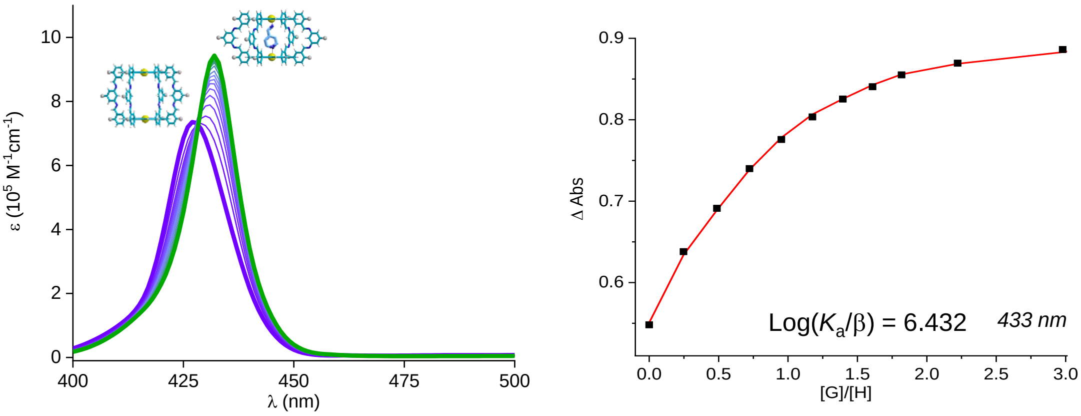

Figure S15B. UV-vis changes recorded along the titration of $\mathbf{1}_{Zn}^{CN}$ at a constant concentration of $1.0 \cdot 10^{-6}$ M with increasing amounts of *mapy* in $CHCl_3$ at 298 K.

NOESY Experiments

However, and in contrast to the situation observed for the $\mathbf{1}_{Zn}^{NC}$·*mapy* complex (Figure S14C), the NOESY spectrum of the $\mathbf{1}_{Zn}^{CN}$·*mapy* complex in $CDCl_3$ (Figure S15C) suggested that the $\mathbf{1}_{Zn}^{CN}$ cage still adopted preferentially the *extended* conformation, since the same cross peaks were observed (marked in green) for the relevant protons as for the empty $\mathbf{1}_{Zn}^{CN}$ cage (please compare with Figure S2C). This is in line with the fast exchange observed in ^{1}H NMR (Figure S15A-1) and suggests that binding is not so strong and/or that the cage is too rigid and stable to force the imine groups to twist to a *compact* conformation.

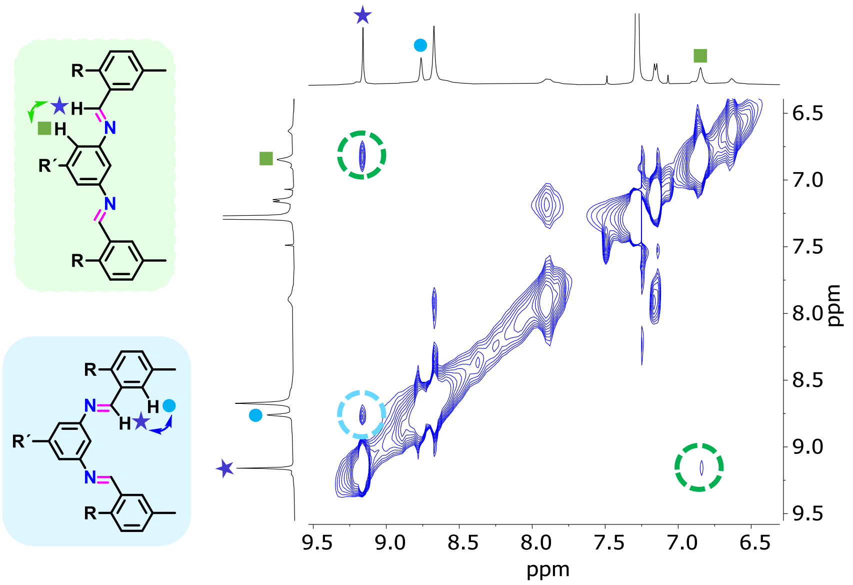

Figure S15C. 2D NOESY spectrum of $\mathbf{1}_{Zn}^{CN}$·*mapy* (1:1) complex in $CDCl_3$ at 298 K that shows cross-peaks consistent with the *extended* conformation.

S16. Host-Guest Chemistry. Binding of 2,6-naphthyridine (*naphy*) to $1_{Zn}{}^{NC}$

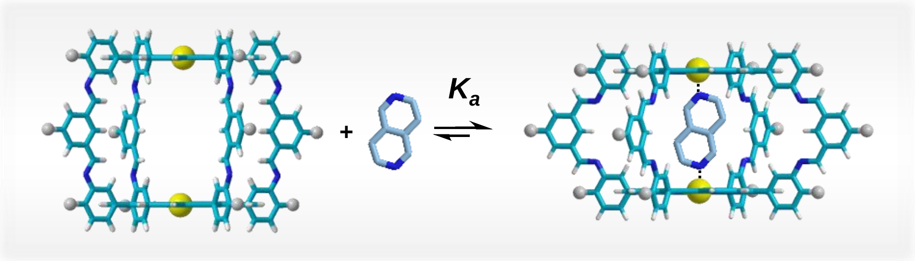

Binding of guest molecules in *Group 3*, like 2,6-naphthyridine (*naphy*), having 2 nitrogen atoms at just the right distance to bind to both Zn^{II} centers in the cavity of $\mathbf{1}_{Zn}{}^{NC}$ in the *compact* conformation, was first evaluated through titration experiments in chloroform monitored by ^{1}H NMR (Figures S16A-1,2) and absorption (Figure S16B).

^{1}H NMR Titrations

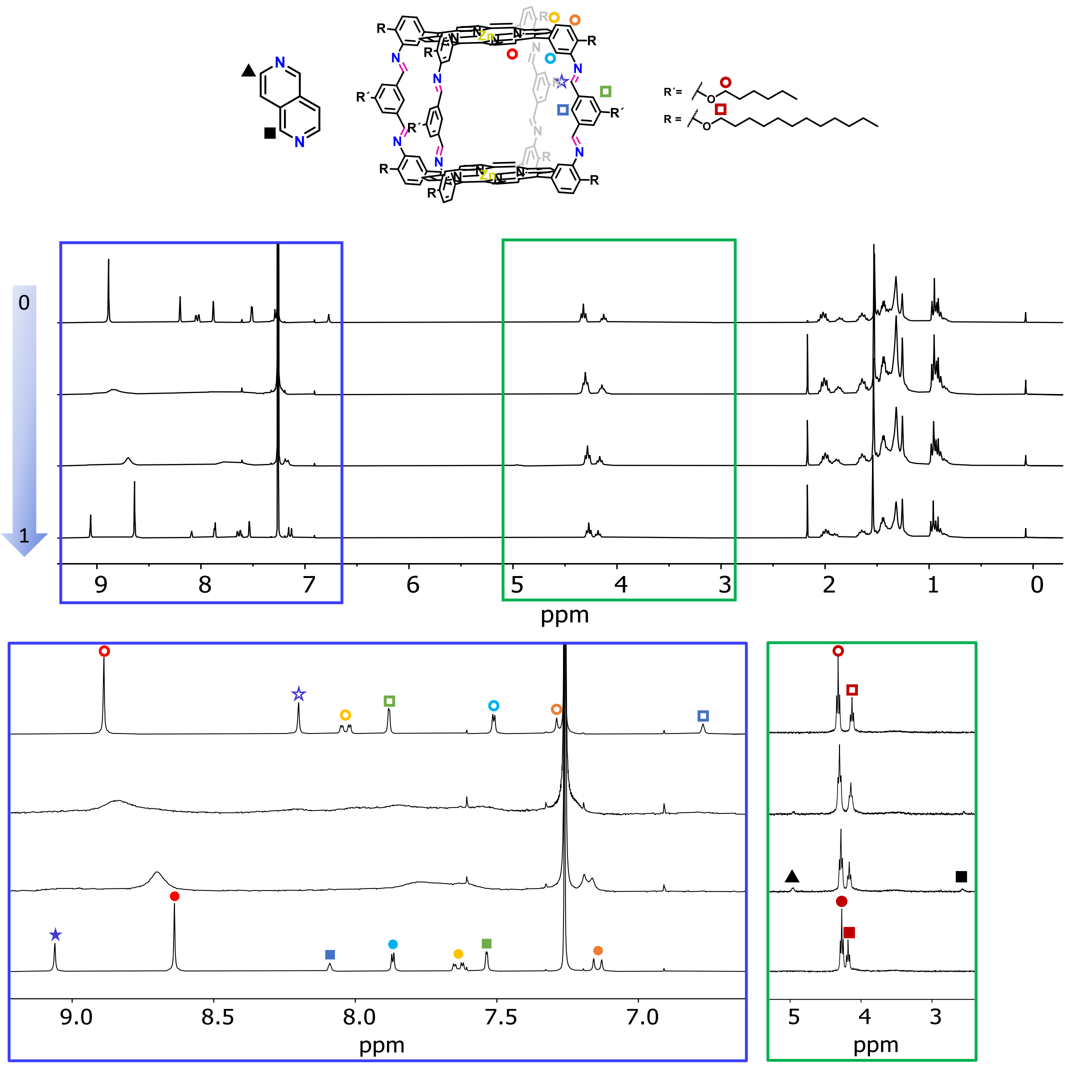

Figure S16A-1. ^{1}H NMR changes recorded along the titration of $\mathbf{1}_{Zn}{}^{NC}$ in $CDCl_3$ at 298 K with increasing amounts of *naphy*.

Just like the complexes formed by $\mathbf{1}_{Zn}^{NC}$ or $\mathbf{1}_{Zn}^{CN}$ and *naphy*, host-guest exchange in $CDCl_3$ for $\mathbf{1}_{Zn}^{NC}$ and *mapy* (Figure S16A-1) was much faster than the one observed for *Group 2* guests, and close to the ^{1}H NMR timescale, which resulted in broad signals at substoichiometric amounts of guest added. As for the $\mathbf{1}_{Zn}^{NC}$·*mapy* complex, a solvent change to toluene-D_8 resulted in a much slower exchange of the $\mathbf{1}_{Zn}^{NC}$·*naphy* complex (Figure S16A-2).

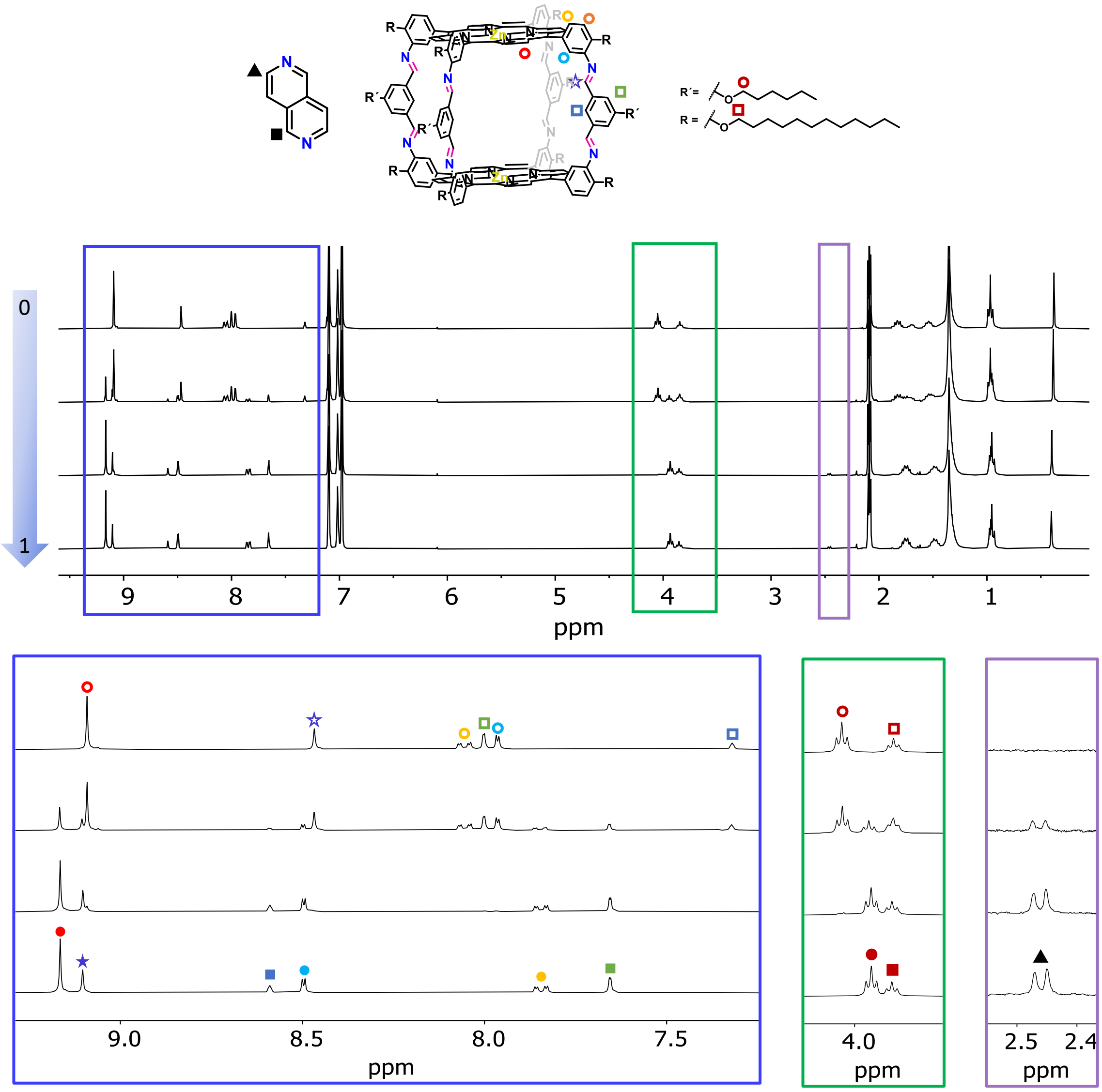

Figure S16A-2. ^{1}H NMR changes recorded along the titration of $\mathbf{1}_{Zn}^{NC}$ in toluene-D_8 at 298 K with increasing amounts of *naphy*.

UV-vis Titrations

UV-vis titrations allowed us to determine the binding constant (K_a) between $\mathbf{1}_{Zn}^{\mathit{NC}}$ and *naphy* in $CHCl_3$, by fitting the binding isotherms to a 1:1 model.

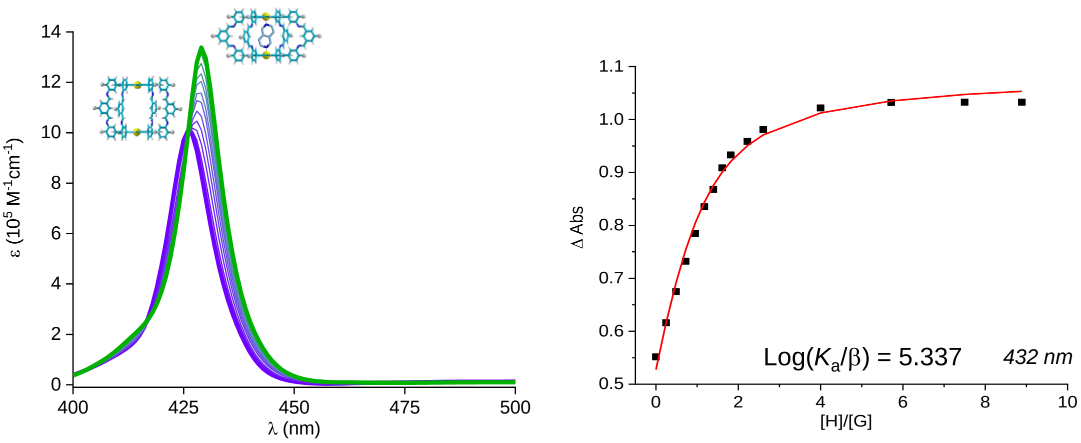

Figure S16B. UV-vis changes recorded along the titration of $\mathbf{1}_{Zn}^{\mathit{NC}}$ at a constant concentration of $1.0 \cdot 10^{-5}$ M with increasing amounts of *naphy* in $CHCl_3$.

NOESY Experiments

As for the $\mathbf{1}_{Zn}^{\mathit{NC}}$·*mapy* complex, the cross-peaks observed in the NOESY spectrum of the $\mathbf{1}_{Zn}^{\mathit{NC}}$·*naphy* complex in $CDCl_3$ (Figure S16C) confirmed that the $\mathbf{1}_{Zn}^{\mathit{NC}}$ cage presented a mixture of the *extended* (marked in green) and *compact* (marked in blue) conformations upon binding to the *naphy* guest (please compare with the empty $\mathbf{1}_{Zn}^{\mathit{NC}}$ cage in Figure S1C).

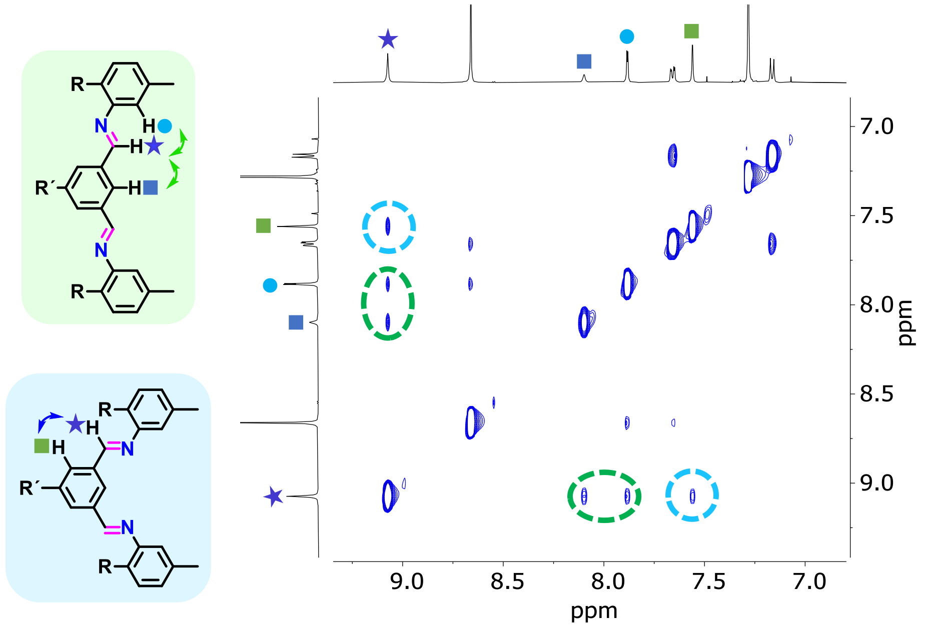

Figure S16C. 2D NOESY spectrum of $\mathbf{1}_{Zn}^{\mathit{NC}}$·*naphy* (1:1) complex in $CDCl_3$ at 298 K, showing ^1H-^1H cross-peaks that are in agreement with the coexistence of the *extended* (marked in green) and *compact* (marked in blue) conformations.

S17. Host-Guest Chemistry. Binding of 2,6-naphthyridine (naphy) to 1_{Zn}^{CN}

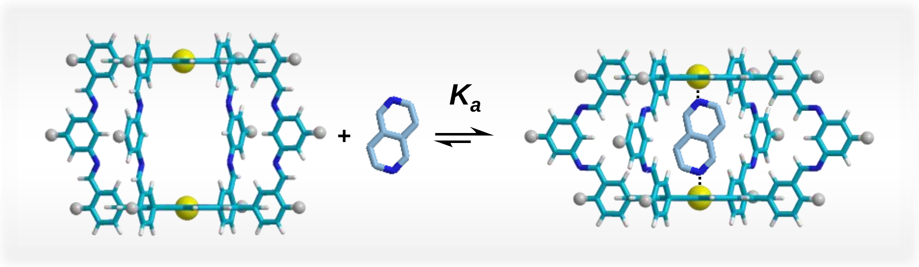

Binding of guest molecules in *Group 3*, like 2,6-naphthyridine (*naphy*), having 2 nitrogen atoms at just the right distance to bind to both Zn^{II} centers in the cavity of $\mathbf{1}_{Zn}^{CN}$ in the *compact* conformation, was first evaluated through titration experiments in chloroform monitored by ^1H NMR (Figures S17A-1,2) and absorption (Figure S17B).

^{1}H NMR Titrations

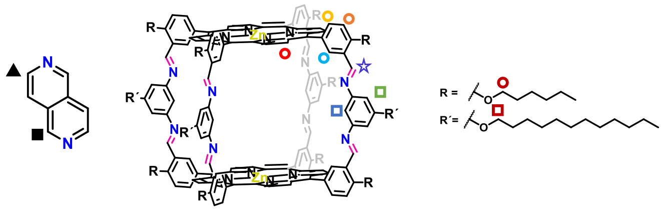

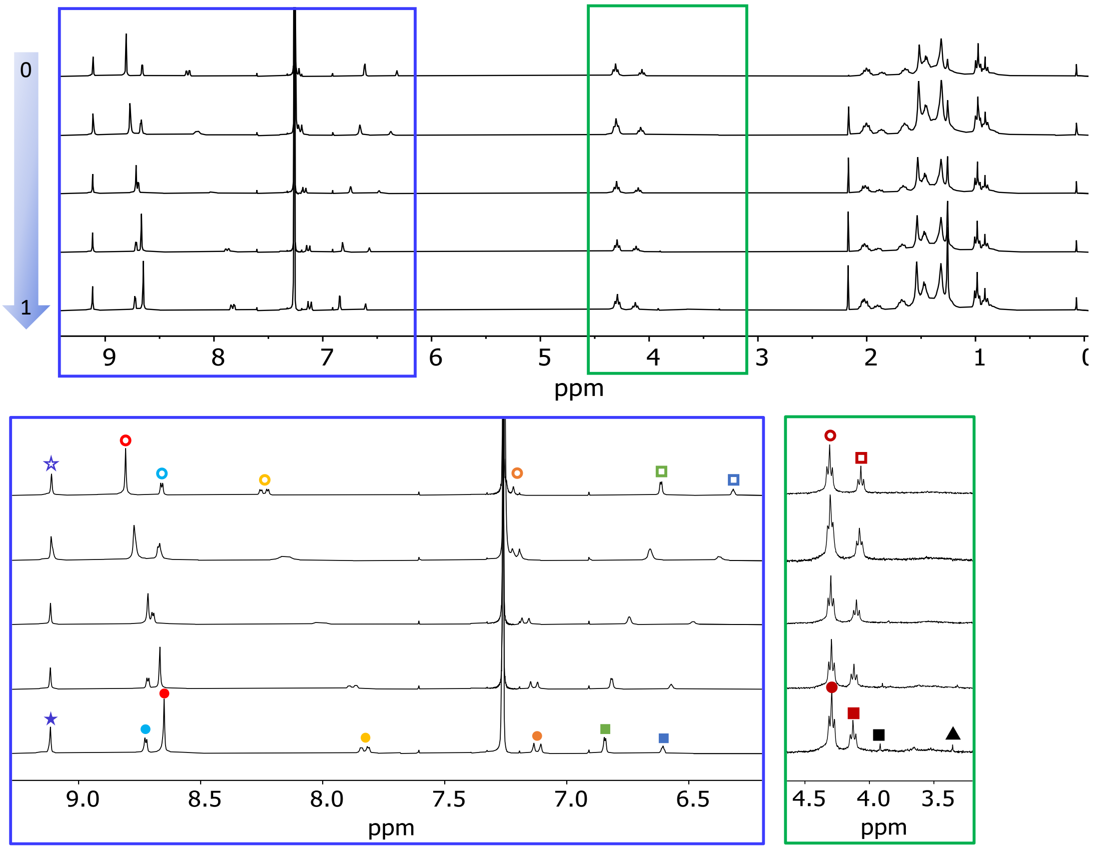

Figure S17A-1. ^{1}H NMR changes recorded along the titration of $\mathbf{1}_{Zn}^{CN}$ with increasing amounts of *naphy* in $CDCl_3$ at 298 K.

Just like the $\mathbf{1}_{Zn}{}^{CN}$·*mapy* complex (Figure S15A-1), the $\mathbf{1}_{Zn}{}^{CN}$·*naphy* complex exhibited now fast NMR exchange in $CDCl_3$ (Figure S17A-1). This is not entirely surprising, since the $\mathbf{1}_{Zn}{}^{NC}$·*naphy* complex already presented broad signals in this solvent (see Figure S16A-1) that suggested that exchange was fast, in comparison to *Group 2* guests. As for the $\mathbf{1}_{Zn}{}^{CN}$·*mapy* complex, a solvent change to toluene-D_8 resulted in a much slower exchange (Figure S17A-2).

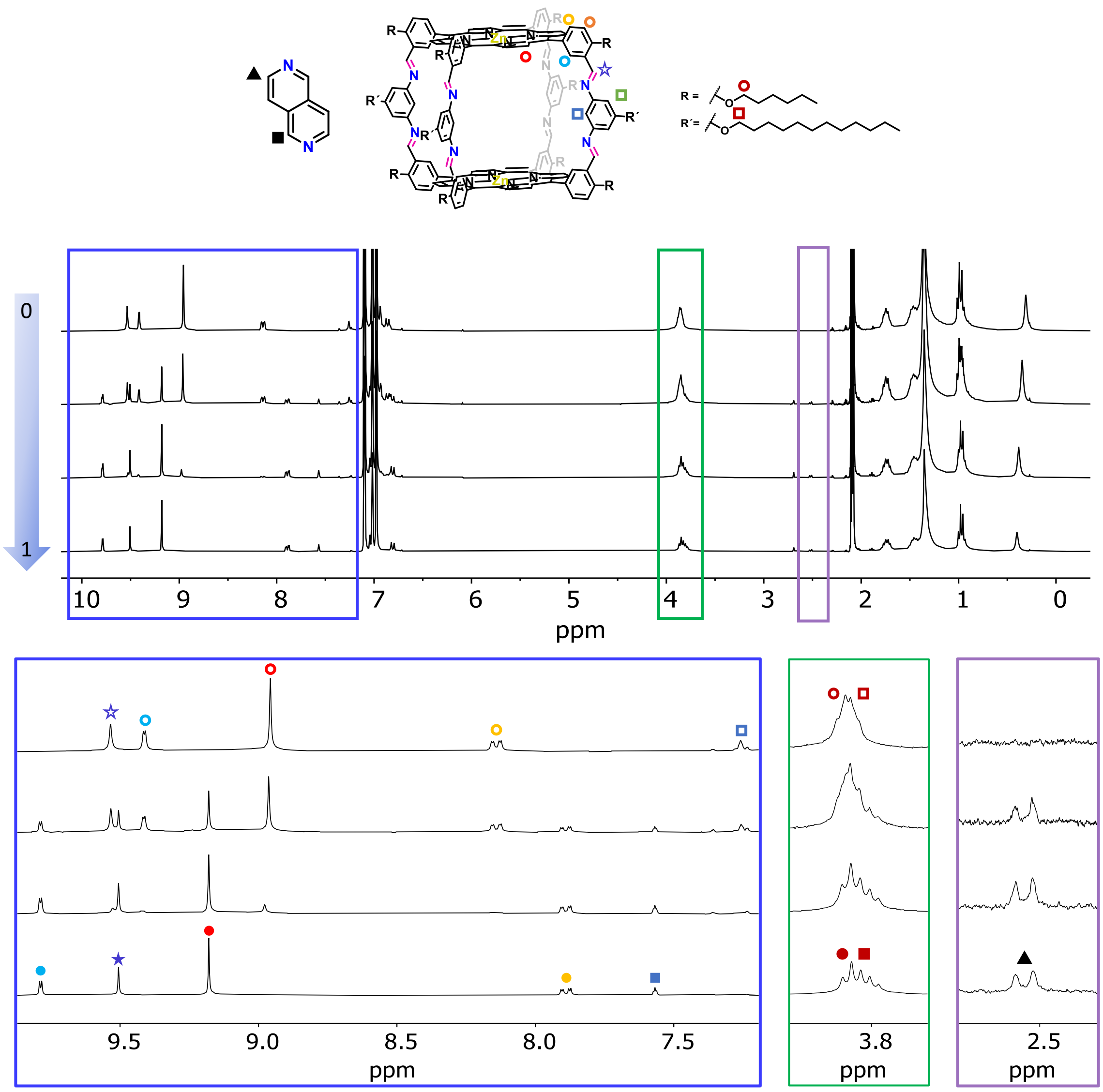

Figure S17A-2. ^{1}H NMR changes recorded along the titration of $\mathbf{1}_{Zn}{}^{CN}$ with increasing amounts of *naphy* in toluene-D_8 at 298 K.

UV-vis Titrations

UV-vis titrations allowed us to determine the binding constant (K_a) between $\mathbf{1}_{Zn}^{CN}$ and *naphy* in $CHCl_3$, by fitting the binding isotherms to a 1:1 model.

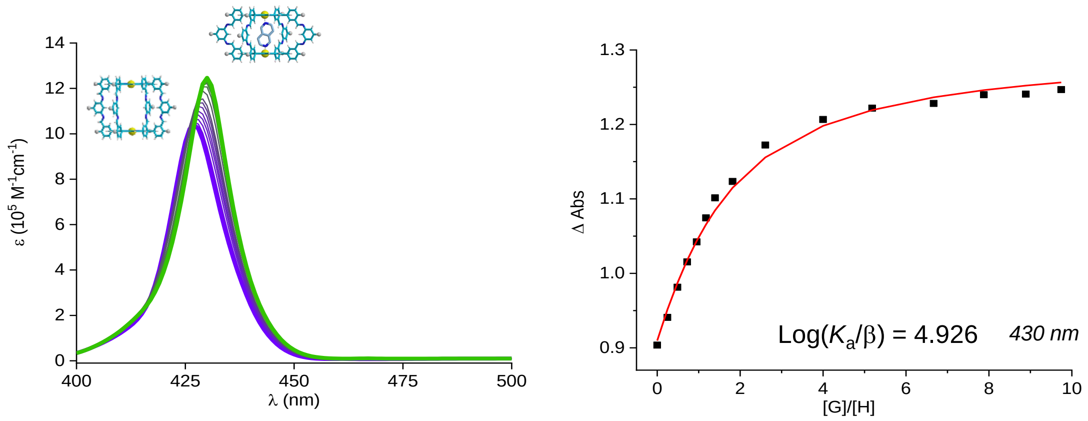

Figure S17B. UV-vis changes recorded along the titration of $\mathbf{1}_{Zn}^{CN}$ at a constant concentration of $1.0 \cdot 10^{-5}$ M with increasing amounts of *naphy* in $CHCl_3$ at 298 K.

NOESY Experiments

On the other hand, a NOESY spectrum in $CDCl_3$ (Figure S17C) suggested that the $\mathbf{1}_{Zn}^{CN}$·*naphy* complex presented mainly an *extended* conformation (cross-peaks marked in green), but the participation of imine arrangements in a *compact* conformation (cross-peaks marked in blue) was in this case noy negligible (please compare with the empty $\mathbf{1}_{Zn}^{CN}$ cage in Figure S2C or with the $\mathbf{1}_{Zn}^{CN}$·*mapy* complex in Figure S15C).

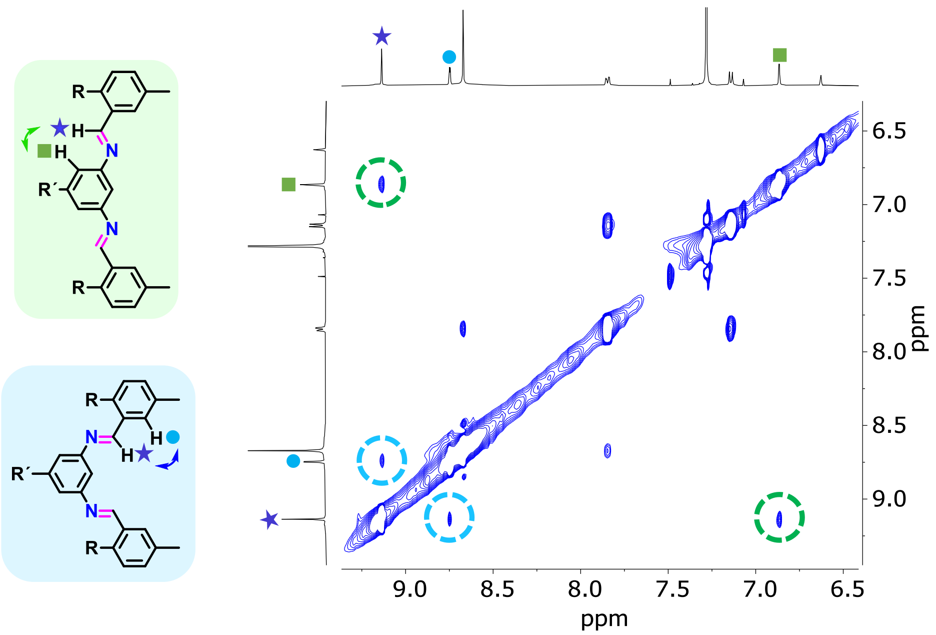

Figure S17C. 2D NOESY spectrum of $\mathbf{1}_{Zn}^{CN}$·*naphy* (1:1) complex in $CDCl_3$ at 298 K, showing ^{1}H-^{1}H cross-peaks that are in agreement with the coexistence of both the *extended* (marked in green) and *compact* (marked in blue) conformations.

S18. Estimation of the energy associated to the *extended*-to-*compact* conformational rearrangement

The energetic cost accompanying the *extended* to *compact* conformational transformation ($\Delta G^0_{e\to c}$) was estimated from the difference between the free energy of association of *bipy* and *naphy* to $\mathbf{1}_{Zn}^{NC}$/$\mathbf{1}_{Zn}^{CN}$ as (Figure S18):

$$\Delta G^0_{e\to c} = \Delta G^0_{compact} - \Delta G^0_{extended} = \Delta G^0_{naphy} - \Delta G^0_{bipy}$$

$\Delta G^0_{e\to c}$ is *ca.* 2 times larger for $\mathbf{1}_{Zn}^{CN}$ than for $\mathbf{1}_{Zn}^{NC}$ in chloroform, which is a manifestation of the fact that $\mathbf{1}_{Zn}^{CN}$ binds more strongly *bipy* and weaklier *naphy* than $\mathbf{1}_{Zn}^{NC}$.

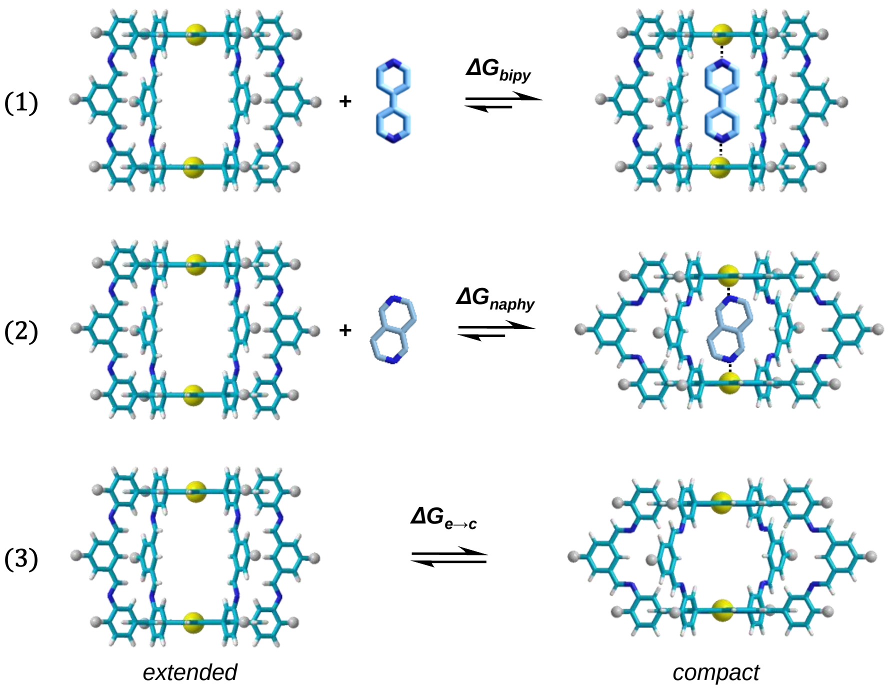

$$(1)\quad \Delta G_{bipy} = -\mathrm{RTLn}(K_{bipy})$$
$$(2)\quad \Delta G_{naphy} = -\mathrm{RTLn}(K_{naphy})$$
$$(3)\quad \Delta G_{e\to c} = \Delta G_{naphy} - \Delta G_{bipy}$$

	H+G System	CHCl$_3$ ΔG (Kcal/mol)	CHCl$_3$ ΔG$_{e\to c.}$ (Kcal/mol)	CHCl$_3$ *Compact* (%)	CHCl$_3$ *Extended* (%)
N=C	$\mathbf{1}_{Zn}^{NC}$ + *bipy*	-11.003	-3.726	0.18	99.82
	$\mathbf{1}_{Zn}^{NC}$ + *naphy*	-7.277			
C=N	$\mathbf{1}_{Zn}^{CN}$ + *bipy*	-12.135	-5.418	0.01	99.99
	$\mathbf{1}_{Zn}^{CN}$ + *naphy*	-6.717			

Figure S18. Estimation of the energetic costs associated to the *extended* to *compact* conformational transformations ($\Delta G^0_{e\to c}$) in CHCl$_3$.

S19. Theoretical Calculations. Conformational Energy Landscapes

In order to shed light on the conformational preferences of cages **1**$_{\mathbf{Zn}}$$^{\boldsymbol{NC}}$ and **1**$_{\mathbf{Zn}}$$^{\boldsymbol{CN}}$ and the geometry adaptations they undergo as a function of the guest added, we performed a set of theoretical calculations in which the imine groups are twisted one-by-one in going from the fully *extended* (no imine twisted) to the totally *compact* (8 imines twisted) conformation. This interconversion can be achieved by modifying the dihedral angle formed by any of the single bonds adjacent to the imine C_I=N bond and two C atoms from the linker (C_L) or the *meso* ring (C_M) (Figure S19A). By means of these calculations, we intend to know: 1) the relative energy between conformations, 2) the energy barriers ruling their interconversion, and 3) the changes in Zn⋯Zn distance for both the empty cages, and the cages were *bipy* or *naphy* were complexed.

Figure S19A shows the relevant dihedrals for the interconversion of *extended* and *compact* conformations for one linker, and the potential energy profile of a relaxed scan along such coordinate. The energy barrier for the twisting of one imine group around dihedral C_L−C_L−C_I−N amounts to 8.5 kcal mol^{-1} in the **1**$_{\mathbf{Zn}}$$^{\boldsymbol{NC}}$ cage, and to a similar value of 8.7 kcal mol^{-1} around dihedral C_M−C_M−C_I−N in the **1**$_{\mathbf{Zn}}$$^{\boldsymbol{CN}}$ cage. These numbers should be taken as upper bounds of the barrier because they are calculated by twisting around only one single bond and more complex concerted motions could take place. The values are well above thermal energy at room temperature (~0.6 kcal mol^{-1}) but collisions with solvent molecules can be energetic enough to overcome it. For the empty **1**$_{\mathbf{Zn}}$$^{\boldsymbol{CN}}$ cage (see Figure S19F below) each imine rotation leads to a constant increase in energy of about 3.0−3.5 kcal mol^{-1}, up to the calculated value of 27.30 kcal mol^{-1} for the fully *compact* conformer. Interestingly, for the **1**$_{\mathbf{Zn}}$$^{\boldsymbol{NC}}$ box (see Figure S19B), the energy slightly decreases up to four rotated imines, and then raises by ~4 kcal mol^{-1} when all imines are converted into the *compact* arrangement.

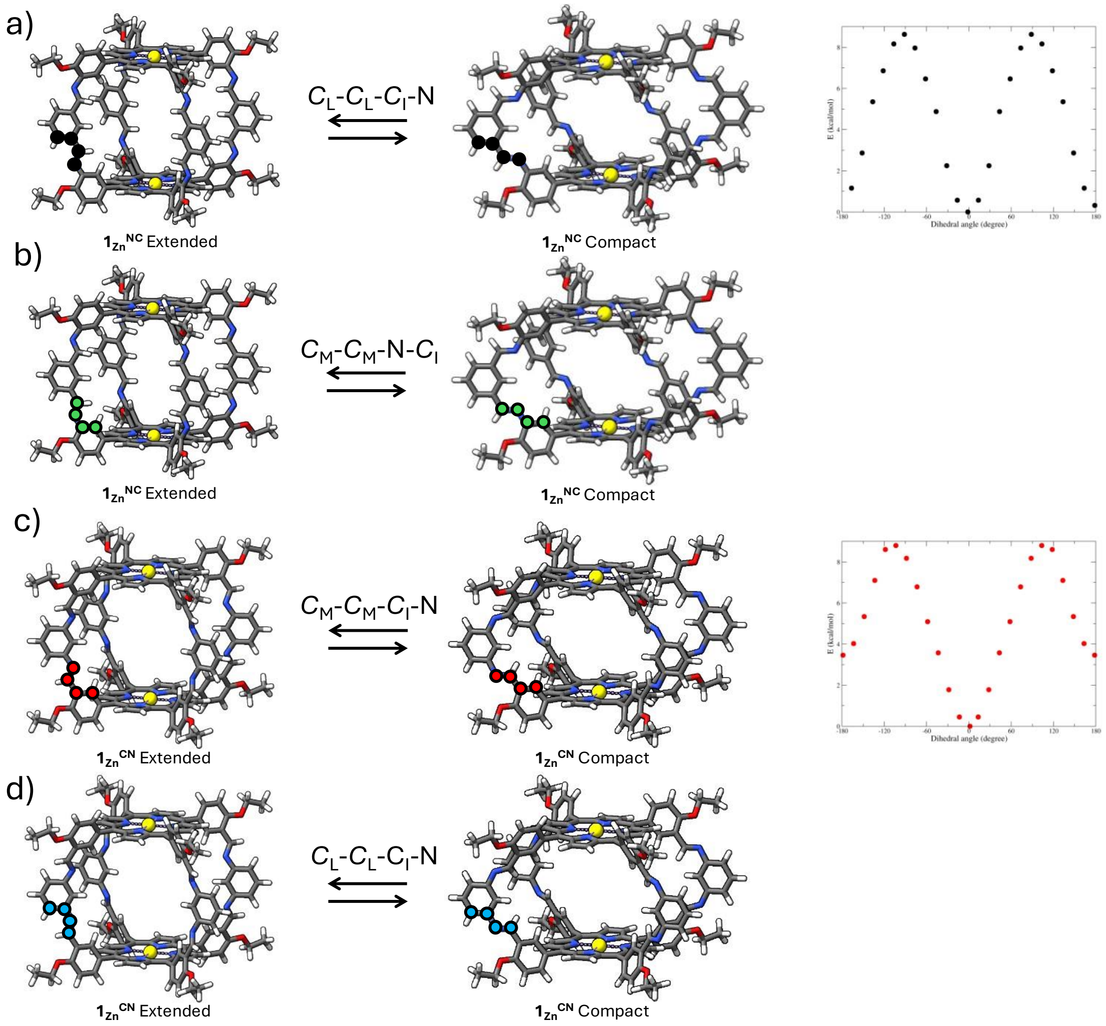

Figure S19A. Interconversion of the *extended* and *compact* conformations for $\mathbf{1}_{Zn}{}^{NC}$ (a,b) and $\mathbf{1}_{Zn}{}^{CN}$ (c,d). One representative dihedral involved in the interchange is highlighted with colored circles and labelled above the arrows (C_L = linker carbon, C_M = *meso* carbon and C_I = imine carbon). The full conversion of one cage into the other requires eight dihedral rotations. The potential energy profiles calculated for the twisting around the highlighted dihedral angle are displayed for (a) and (c).

The binding of *bipy* and *naphy* molecules by the $\mathbf{1}_{Zn}{}^{NC}$ / $\mathbf{1}_{Zn}{}^{CN}$ cages in both *extended* and *compact* conformations has been studied in great detail at the B3LYP/cc-PVDZ level. According to theoretical calculations, the size of the guest is indeed decisive for the preferred conformation adopted by the cage.

Figure S19B displays the minimum-energy optimized structures of the fully *extended* and *compact* conformations of the $\mathbf{1}_{Zn}{}^{NC}$•*bipy* complex, in comparison with the empty $\mathbf{1}_{Zn}{}^{NC}$ cage. It also plots the evolution of the relative energy, and the Zn···Zn distance calculated for the empty cage and the complex as the imine groups are rotated. When *bipy* is hosted inside each receptor, the energy increases quadratically with each imine rotation, reaching up to ~15 and ~42 kcal mol^{-1} for the totally *compact* conformations of $\mathbf{1}_{Zn}{}^{NC}$ and $\mathbf{1}_{Zn}{}^{CN}$, respectively (Figures S19B,F). The Zn···Zn distance in the empty $\mathbf{1}_{Zn}{}^{NC}$ cage diminishes almost linearly between the completely

extended (12.30 Å) and *compact* (9.75 Å) conformations by ~0.4 Å per rotated imine. In contrast,for the $\mathbf{1}_{Zn}{}^{NC}$·*bipy* complex, the Zn···Zn distance remains almost constant in the 11.50-11.75 Å range in passing from the *extended* to the *compact* conformation. This nicely matches the length of *bipy* (~7.10 Å) plus twice the N···Zn distance (~2.3 Å).

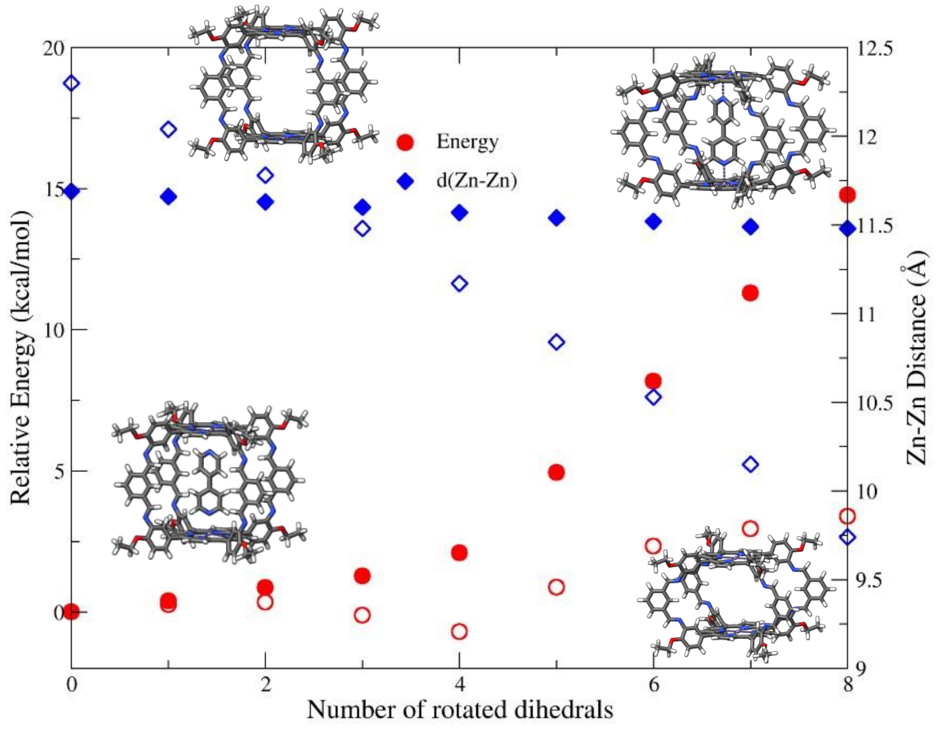

Figure S19B. Relative energy (circles) and Zn···Zn distance (diamonds) calculated at the B3LYP/cc-PVDZ level for the empty $\mathbf{1}_{Zn}{}^{NC}$ cage (hollow icons) and the $\mathbf{1}_{Zn}{}^{NC}$·*bipy* complex (filled icons) as a function of the number of imine dihedral angles rotated. Zero rotated dihedrals correspond to the *extended* conformation and eight rotated dihedrals to the *compact* conformation.

The above calculations show that, in order to fit *bipy* into the *compact* conformation, the $\mathbf{1}_{Zn}{}^{NC}$ cage should be largely elongated to provide a cavity almost 2 Å larger than its optimal value (9.75 Å, as shown in Figure S19C (empty cage in blue and $\mathbf{1}_{Zn}{}^{NC}$·*bipy* complex in red). The high energy cost implied in the transformation from the *extended* to the *compact* conformation suggests that, upon *bipy* uptake, any dynamic equilibrium among conformations is fully displaced towards the *extended* version.

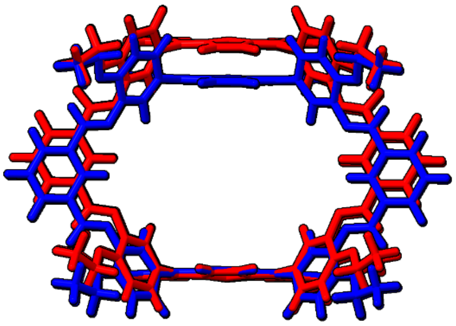

Figure S19C. Comparison of the optimized $\mathbf{1}_{Zn}{}^{NC}$ structure in the *compact* conformation when the cage is empty (blue) and in the $\mathbf{1}_{Zn}{}^{NC}$·*bipy* complex (red). The Zn···Zn distances are 9.75 and 11.48 Å, respectively.

The binding of *Group 3* molecules by $\mathbf{1}_{Zn}{}^{NC}$ and $\mathbf{1}_{Zn}{}^{CN}$ cages was especially studied for the *naphy* guest. Figure S19D displays the minimum-energy optimized structures of the fully *extended* and *compact* conformations of the $\mathbf{1}_{Zn}{}^{NC}$·*naphy* complex. It also plots the evolution of the relative energy, and the Zn···Zn distance calculated for the empty cage and the complex as the imine groups are rotated. When *naphy* (or *mapy*) is included as a guest in $\mathbf{1}_{Zn}{}^{NC}$, the twisting of the imine groups always results in a more stable complex, a minimum energy being achieved for the conformation with 4/5 imines rotated, resulting in a $\mathbf{1}_{Zn}{}^{NC}$·*naphy* conformer 8.5 kcal mol^{-1} more stable than the totally *extended* conformation. Further compaction of the cage slightly destabilizes the complex. This suggests that the size of *naphy* (N···N distance of 5.10 Å) is actually optimal for an intermediate conformation in between the *extended* and *compact* cages, where imine bonds in these two arrangements coexist. This finding is confirmed by the analysis of the Zn···Zn distance, that abruptly decreases upon twisting the first imine group, then undergoes a smooth shortening of 0.1 Å per rotated imine until four, and finally lowers by 0.05 Å per imine group, suggesting that from this point the cavity is below the optimal size. A Zn···Zn distance of 9.52 Å is obtained for the fully compacted conformation lodging *naphy*, which is calculated 1.65 kcal mol^{-1} less stable than the intermediate structure with only 4 rotated imines (Figure S19D). Computational simulations also reveal that in the *extended* conformation the *naphy* guest is attached to a single Zn atom (Zn···Zn distance of 11.67 Å), whereas in all other cases it is linked to both Zn atoms (Zn···Zn distances in the 9.5–10.0 Å range).

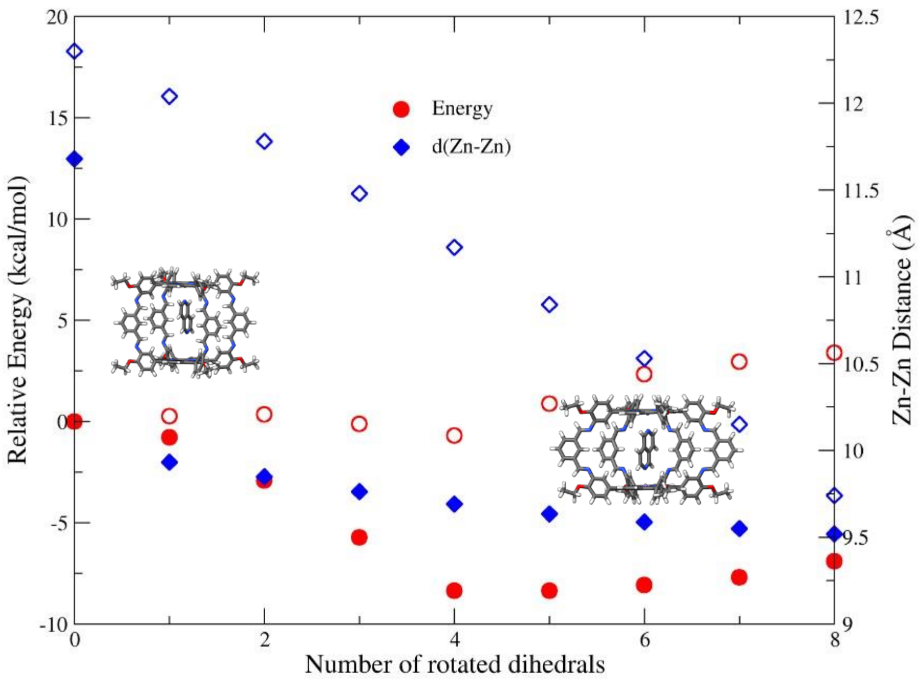

Figure S19D. Relative energy (circles) and Zn···Zn distance (diamonds) calculated at the B3LYP/cc-PVDZ level for the empty $\mathbf{1}_{Zn}^{NC}$ cage (hollow icons) and the $\mathbf{1}_{Zn}^{NC}$·*naphy* complex (filled icons) as a function of the number of imine dihedral angles rotated. Zero rotated dihedrals result in the *extended* conformation and eight rotated dihedrals in the *compact* conformation.

Due to the high energy required to transform the $\mathbf{1}_{Zn}^{CN}$ cage from the *extended* to the *compact* conformation, this cage prefers to incorporate the *naphy* guest preserving the *extended* conformation. Two minimum-energy structures calculated at the B3LYP/cc-PVDZ level were actually obtained upon introducing *naphy* into the $\mathbf{1}_{Zn}^{CN}$ cage (Figure S19E). The first structure corresponds to *naphy* linked to only one Zn atom and shows a Zn···Zn distance of 11.09 Å, significantly shorter than the empty cage (11.95 Å), with well differentiated Zn–N_{naphy} distances of 2.30 and 3.80 Å. In the second structure, the $\mathbf{1}_{Zn}^{CN}$ cage is more compressed (Zn···Zn distance of 9.87 Å) to bind both *naphy* nitrogens at Zn–N_{naphy} distances of 2.42 Å. The second structure, bischelating the *naphy* guest, results slightly more stable (0.98 kcal mol^{-1}) than the former one. This compression of the *extended* conformation has an energy cost of 15.24 kcal mol^{-1}, which is however largely compensated by the stabilizing interaction between *naphy* and the distorted cage (–30.84 kcal mol^{-1}). Thus, considering only enthalpic contributions, theoretical calculations would suggest that the $\mathbf{1}_{Zn}^{CN}$·*naphy* complex might actually coexist as a mixture of monochelated and bischelated structures with the $\mathbf{1}_{Zn}^{CN}$ cage in the *extended* conformation. However, the contribution of cooperativity, which is dominated by entropic considerations, actually adds an extra stabilization in favor of the bischelated species.

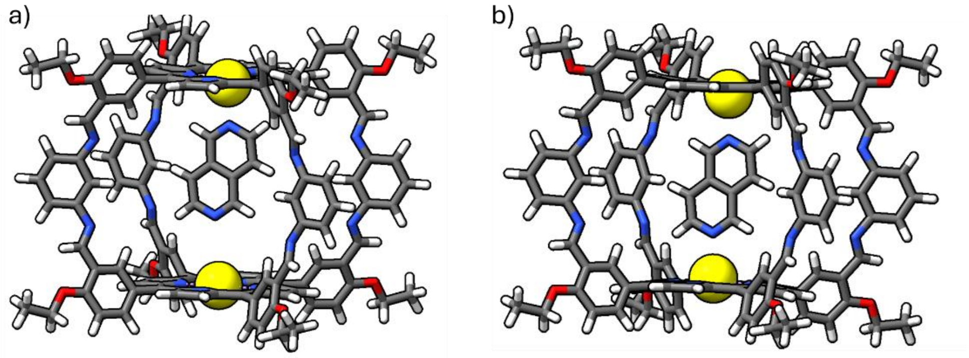

Figure S19E. Monochelated (a) and bischelated (b) minimum-energy structures calculated at the B3LYP/cc-PVDZ level for the $\mathbf{1}_{Zn}^{CN}$·*naphy* complex in the *extended* conformation of $\mathbf{1}_{Zn}^{CN}$.

Figure S19F depicts the fully optimized minimum-energy structure of the $\mathbf{1}_{Zn}^{CN}$·*naphy* complex using the *compact* conformation of $\mathbf{1}_{Zn}^{CN}$ and shows how the energy of the empty $\mathbf{1}_{Zn}^{CN}$ cage, the $\mathbf{1}_{Zn}^{CN}$·*bipy* complex, and the $\mathbf{1}_{Zn}^{CN}$·*naphy* complex increases as the imine groups are rotated in going from the *extended* (zero rotated imines) to the *compact* (eight rotated imines) conformation. In contrast to the $\mathbf{1}_{Zn}^{CN}$·*bipy* complex, for which the increase in energy (41.98 kcal mol^{-1}) is higher than for the empty cage (27.30 kcal mol^{-1}) due to the size of the *bipy* guest, the inclusion of *naphy* leads to a lower but still relevant energy increase (23.13 kcal mol^{-1}) upon imine rotation.

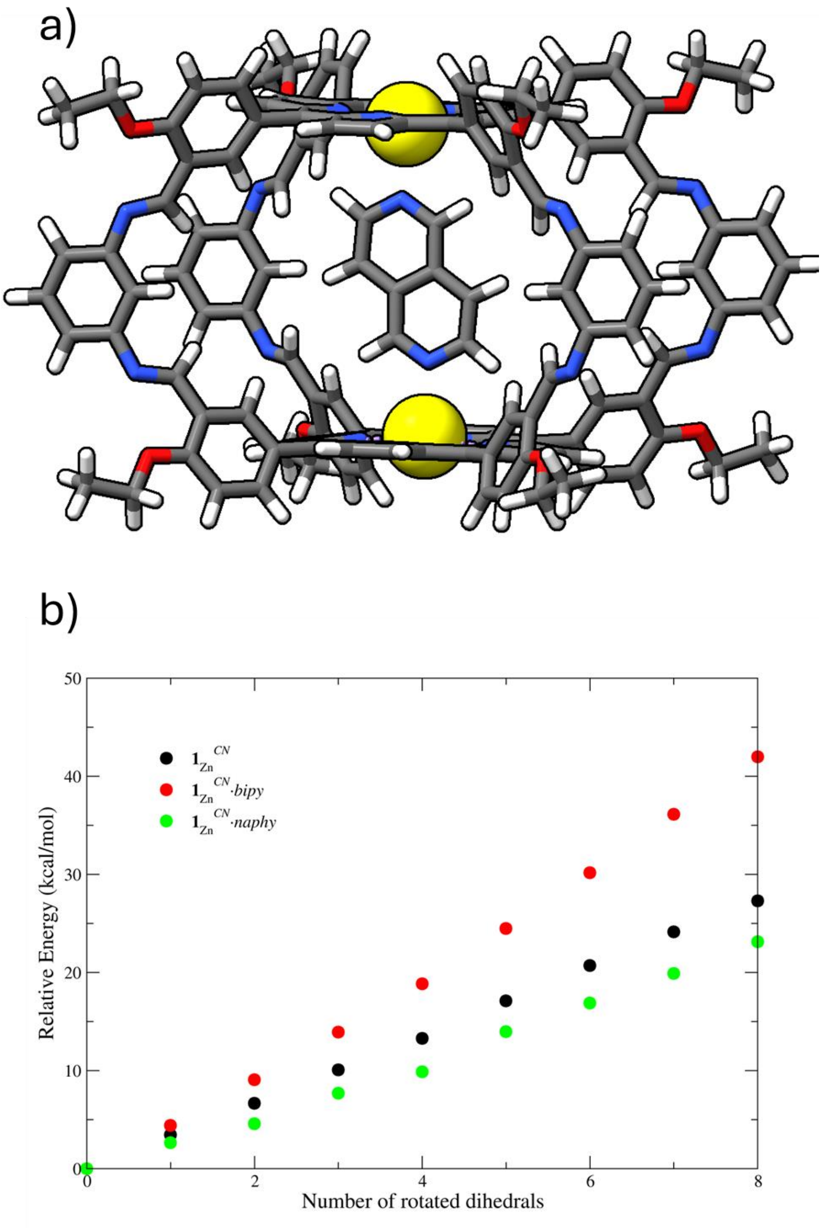

Figure S19F. a) Minimum-energy structure calculated at the B3LYP/cc-PVDZ level for the $\mathbf{1}_{\mathbf{Zn}}^{\boldsymbol{CN}}$·*naphy* complex in the *compact* conformation of $\mathbf{1}_{\mathbf{Zn}}^{\boldsymbol{CN}}$. b) Relative energy computed for the empty $\mathbf{1}_{\mathbf{Zn}}^{\boldsymbol{CN}}$ cage, the $\mathbf{1}_{\mathbf{Zn}}^{\boldsymbol{CN}}$·*bipy* complex, and the $\mathbf{1}_{\mathbf{Zn}}^{\boldsymbol{CN}}$·*naphy* complex as a function of the number of imine dihedral angles rotated in going from the *extended* (zero rotated imines) to the *compact* (eight rotated imines) conformation.

Figure S19G shows a summary of the enthalpic energy landscape for each empty cage and the corresponding complexes with *bipy* and *naphy*. Needless to say, the actual supramolecular picture in solution is a dynamically interconverting mixture of diverse conformations and bound and unbound species that contribute with different populations to the overall energy landscape. The average information provided by solution experiments and the single-molecule picture afforded by theory, as summarized in Figure S19G, both contributing to our understanding of this landscape.

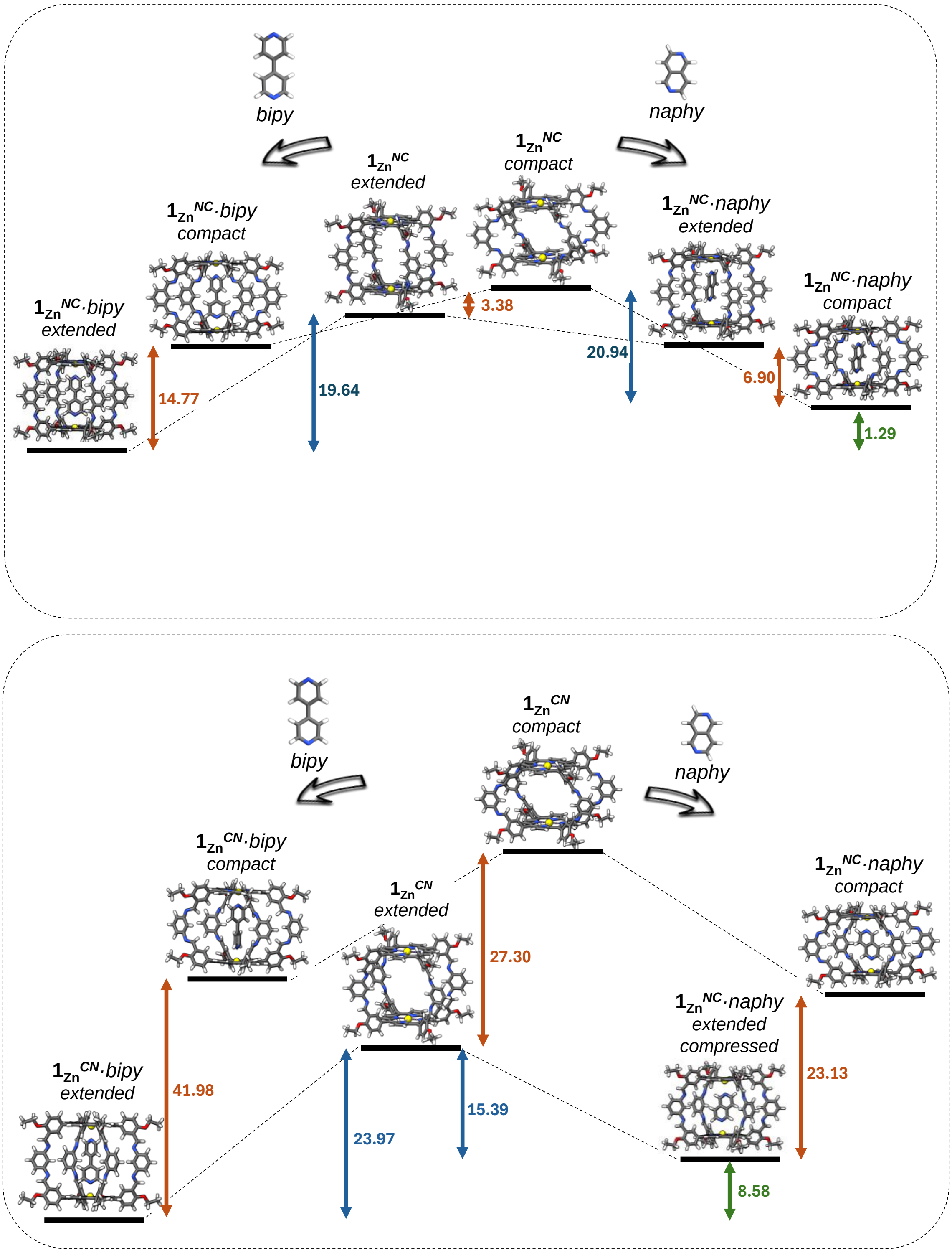

Figure S19G. Conformational energy landscape for the *extended* and *compact* conformations of empty $\mathbf{1}_{Zn}^{NC}$ and $\mathbf{1}_{Zn}^{CN}$ cages and their complexes with *bipy* and *naphy* guests calculated at the B3LYP/cc-PVDZ level. Energy differences between cages (empty or occupied) in *extended* and *compact* conformations are given in red. Binding energies (E_{bind}) in hosting *bipy* and *naphy* are denoted in blue. The differences in E_{bind} between the respective *bipy* and *naphy* complexes are quoted in green. Binding energies are calculated as the energy difference between the total energies of the fully optimized structures of the supramolecular complex and the respective components (*i.e.*, the empty cage and the guest).

S20. Characterization of 2_{Zn}^{NC}

In order to assess the actual contribution of rigidity to the binding affinities and selectivities attained by the $\mathbf{1}_{Zn}^{NC}$ and $\mathbf{1}_{Zn}^{CN}$ *sp*^{2}-cages, the imine C=N bonds of $\mathbf{1}_{Zn}^{NC}$ were reduced in the presence of NaBH(OAc)$_3$, leading to $\mathbf{2}_{Zn}^{NC}$ in 70% yield. As shown in Figure S20A, the imine bond disappears upon reduction and the aromatic signals, including the β-pyrrolic protons, suffer an up field shift that can be attributed to the loss of π-conjugation in the linker and to the different electronic character of the amine substituent, on one hand, and to the closer distance between porphyrins, on the other, as predicted by the theoretical calculations.

^{1}H NMR

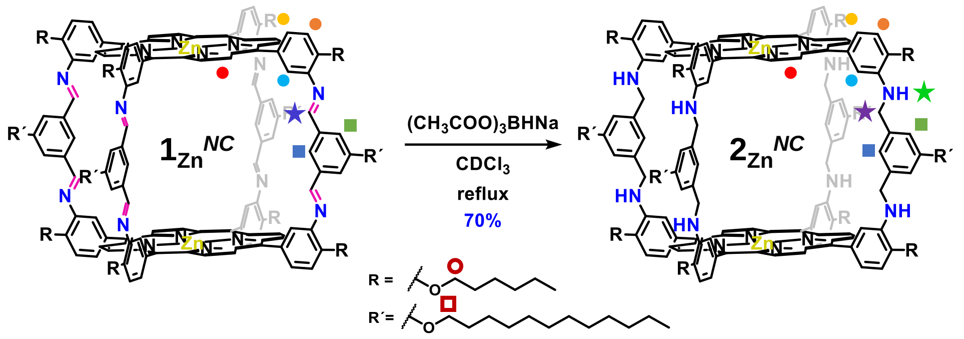

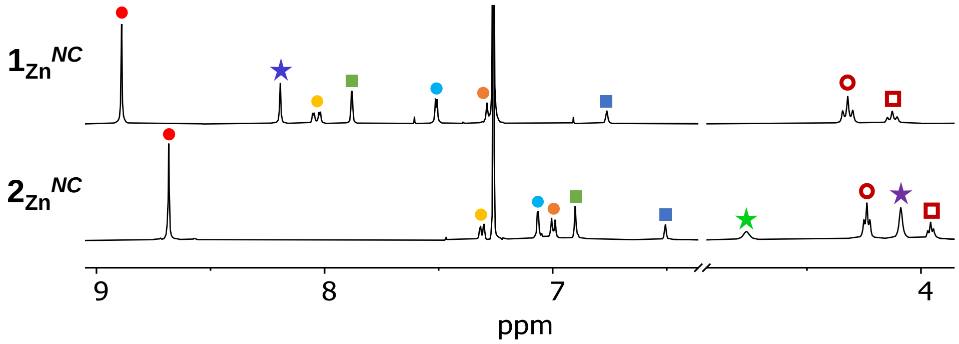

Figure S20A. Comparison of the ^{1}H NMR spectra of $\mathbf{1}_{Zn}^{NC}$ and $\mathbf{2}_{Zn}^{NC}$ at a constant concentration of $1.0 \cdot 10^{-3}$ M at 298 K in CDCl$_3$.

Absorption and Emission

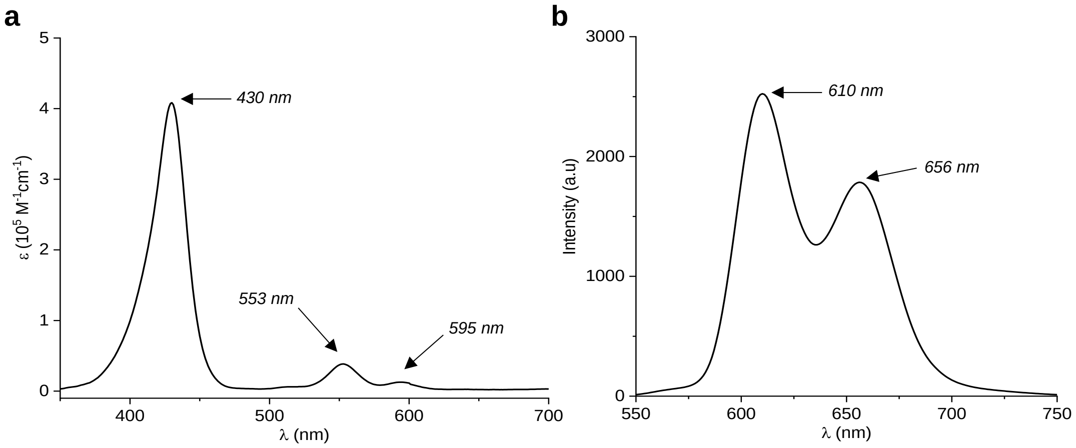

Figure S20B. (**a**) UV-vis absorption and (**b**) fluorescence emission spectra of $\mathbf{2}_{\mathbf{Zn}}^{\boldsymbol{NC}}$ in $CHCl_3$ at 298 K.

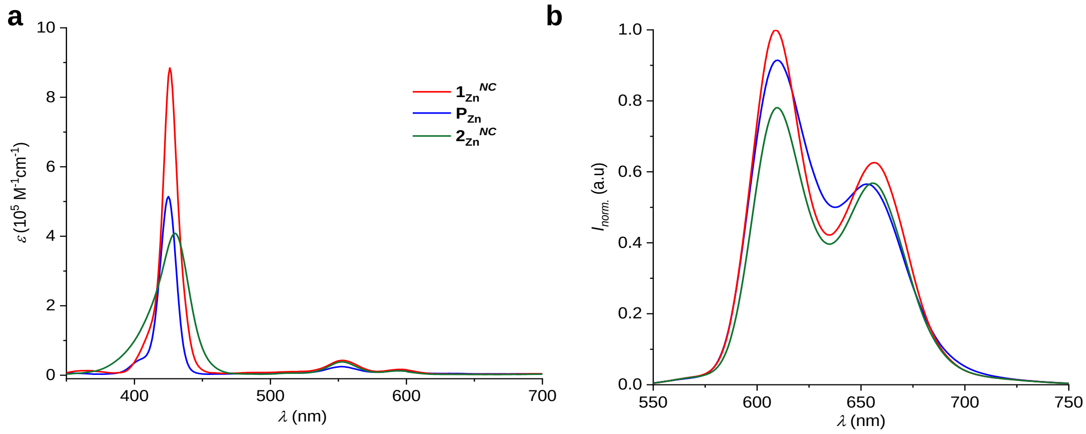

Figure S20C. Comparison of (**a**) UV-vis absorption and (**b**) fluorescence emission spectra of $\mathbf{1}_{\mathbf{Zn}}^{\boldsymbol{NC}}$ (red line), $\mathbf{P}_{\mathbf{Zn}}$ (blue line) and $\mathbf{2}_{\mathbf{Zn}}^{\boldsymbol{NC}}$ (green line) in $CHCl_3$ at 298 K.

NOESY Experiments

The NOESY spectrum in $CDCl_3$ (Figure S20D) confirmed that the $\mathbf{2}_{Zn}^{NC}$ cage presented cross peaks not seen before in the rigid $\mathbf{1}_{Zn}^{NC}$ and $\mathbf{1}_{Zn}^{CN}$ cages (please compare with Figures S1C and S2C) due to its higher flexibility. For instance, the methylene protons in the linker displayed cross-peaks with all the neighbouring aromatic protons of the *meso*-arenes and the linker (marked in green), as well as with the β-pyrrolic protons (marked in blue).

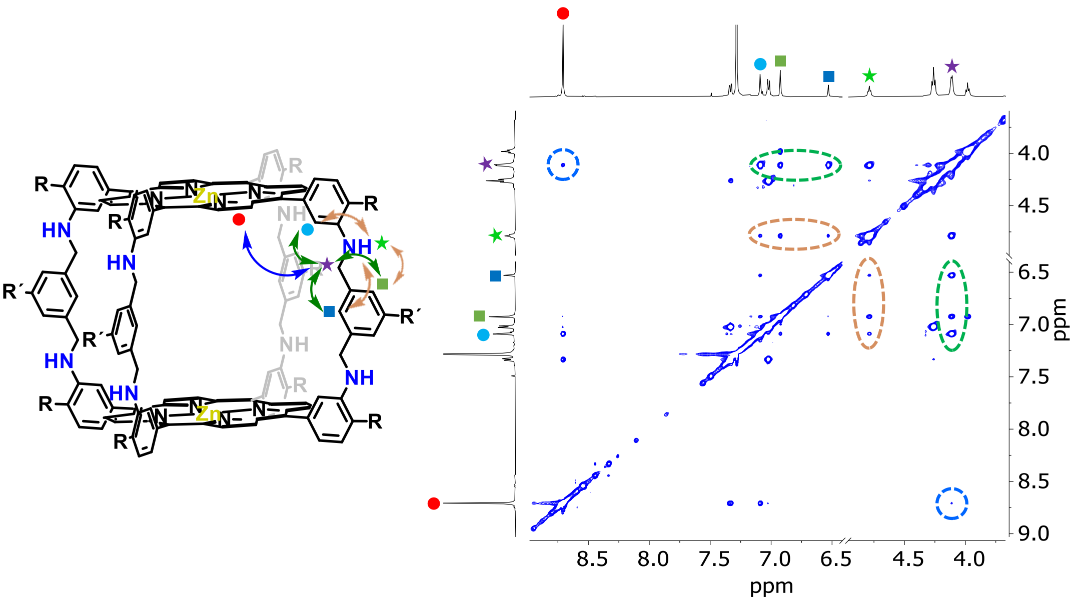

Figure S20D. 2D NOESY spectrum of the $\mathbf{2}_{Zn}^{NC}$ cage in $CDCl_3$ at 298 K, showing a larger number of NOE cross-peaks that suggest a higher flexibility of this cage.

Mass spectrometry

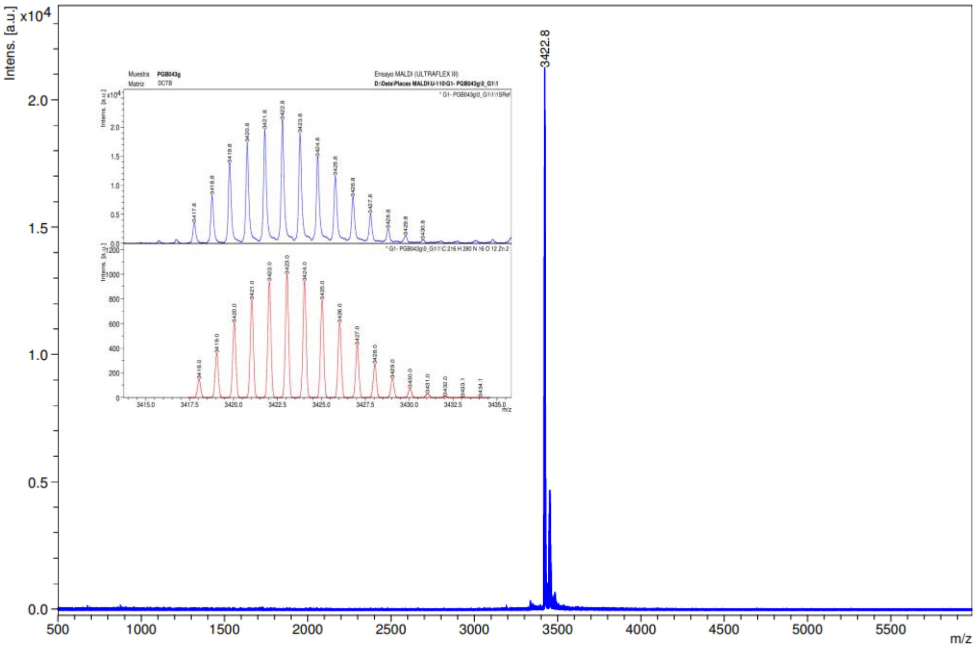

Figure S20E. MS (MALDI) spectra of $\mathbf{2}_{Zn}^{NC}$, showing the comparison of the experimental and theoretical isotopic distribution of the parent *ion* $[M]^+$.

S21. Theoretical calculations. Binding of guest molecules to $\mathbf{2}_{Zn}^{NC}$

The geometry of the empty $\mathbf{2}_{Zn}^{NC}$ cage in different conformations was first studied at the B3LYP/cc-PVDZ level. Figure S21 (top) shows the minimum-energy fully extended conformation in which the Zn atoms are separated by a distance of 13.54 Å. The reduction of the imine –HC=N− groups to −H_2C−NH− increases the flexibility of the linkers and the porphyrin rings can approach to each other. Indeed, when introducing the Grimme's D3 correction to include dispersion effects in the calculation,[5] a close-contact structure, as shown in Figure S21 (bottom), in which the porphyrin planes are at an average distance of only 3.5 Å is obtained. The higher flexibility of the linkers compared with $\mathbf{1}_{Zn}^{NC}$ allows $\mathbf{2}_{Zn}^{NC}$ to bind molecules from *Groups 2*, *3* and *4*.

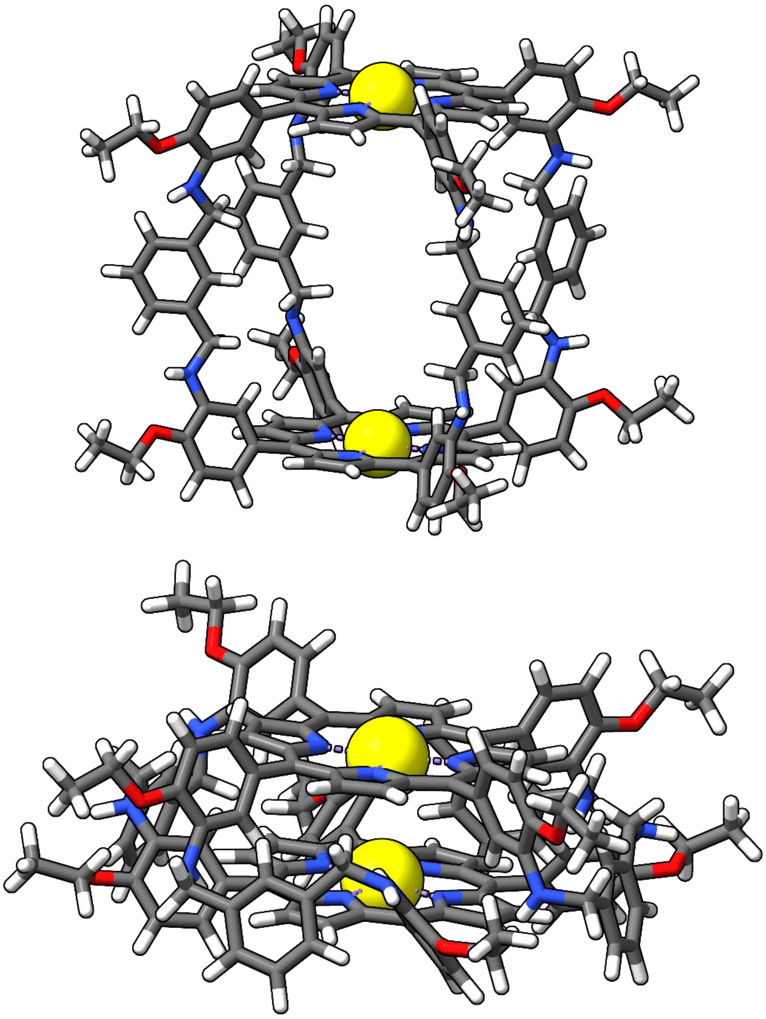

Figure S21. B3LYP/cc-PVDZ-optimized structures calculated for the reduced $\mathbf{2}_{Zn}^{NC}$ cage in a fully extended conformation (a) and in a compressed conformation (b) in which the porphyrins moieties are in close contact. Dispersion D3 corrections are included in the calculation.

S22. Host-Guest Chemistry. Binding of *Group 4* guests (*DABCO*) to $\mathbf{2}_{Zn}^{NC}$

Binding of guest molecules in *Group 4*, like *DABCO*, was first evaluated through ^{1}H NMR titration experiments in $CDCl_3$ (Figure S22A). The gradual addition of *DABCO* now shows bound and unbound receptors as sharp signals in slow NMR exchange.

^{1}H NMR Titrations

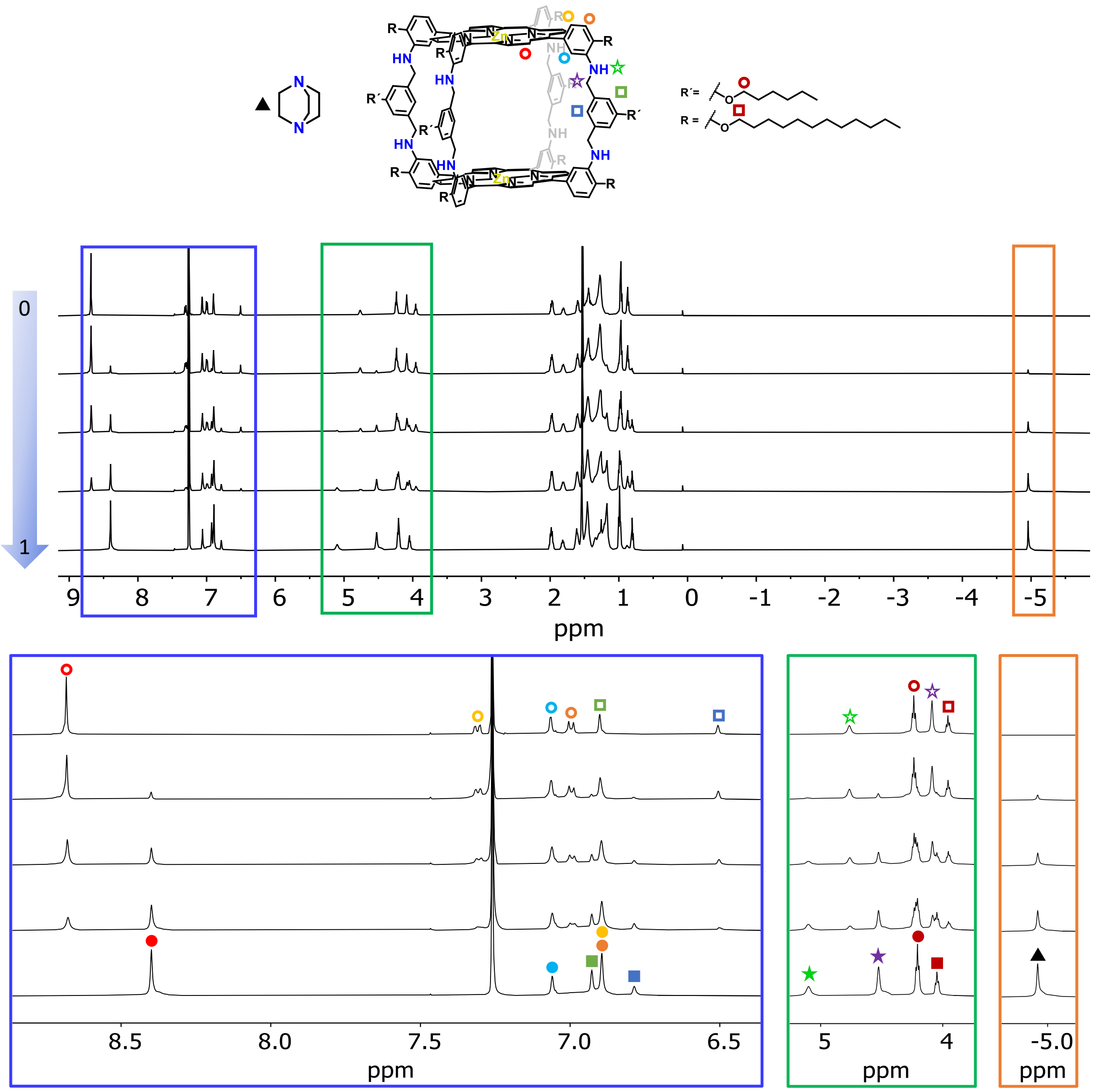

Figure S22A. ^{1}H NMR changes recorded along the titration of $\mathbf{2}_{Zn}^{NC}$ with increasing amounts of *DABCO* in $CDCl_3$ at 298 K (up to 1.0 eq.).

UV-vis Titrations

UV-vis titrations allowed us to determine the binding constant (K_a) between **2**$_{Zn}$^{NC} and *DABCO* in $CHCl_3$, by fitting the binding isotherms to a 1:1 model.

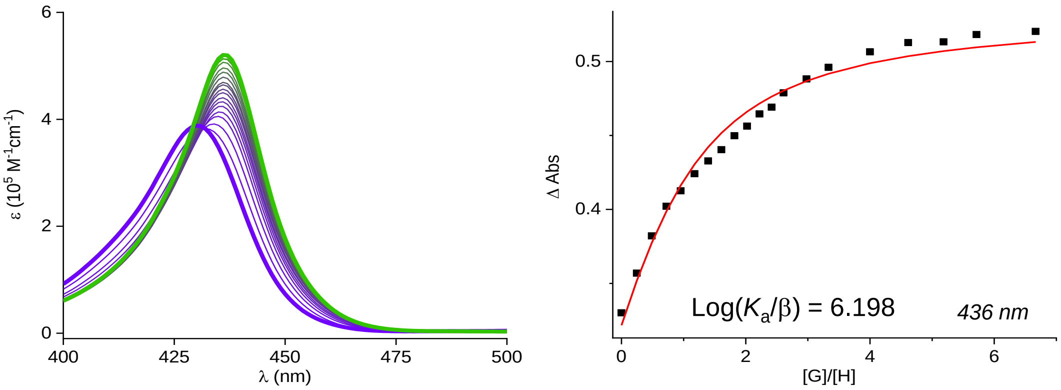

Figure S22B. UV-vis changes recorded along the titration of **2**$_{Zn}$^{NC} at a constant concentration of $1.0 \cdot 10^{-6}$ M with increasing amounts of *DABCO* in $CHCl_3$ at 298 K.

NOESY Experiments

In NOESY experiments in $CDCl_3$ (Figure S22C) very similar cross-peaks were observed for the **2**$_{Zn}$^{NC}·*DABCO* complex than for the empty **2**$_{Zn}$^{NC} cage (please compare with Figure S20D). Interestingly, the *DABCO* protons at –4.9 ppm displayed NOE cross peaks with the pyrrolic protons and the internal aromatic protons at the *meso*-arene groups.

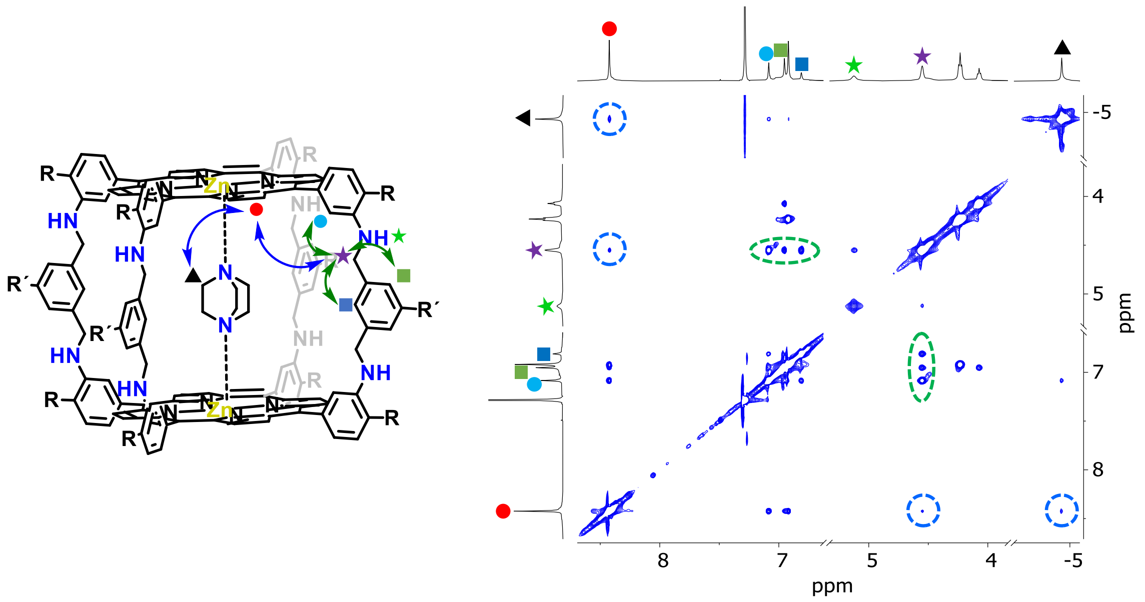

Figure S22C. 2D NOESY spectrum of the **2**$_{Zn}$^{NC}·*DABCO* (1:1) complex in $CDCl_3$ at 298 K.

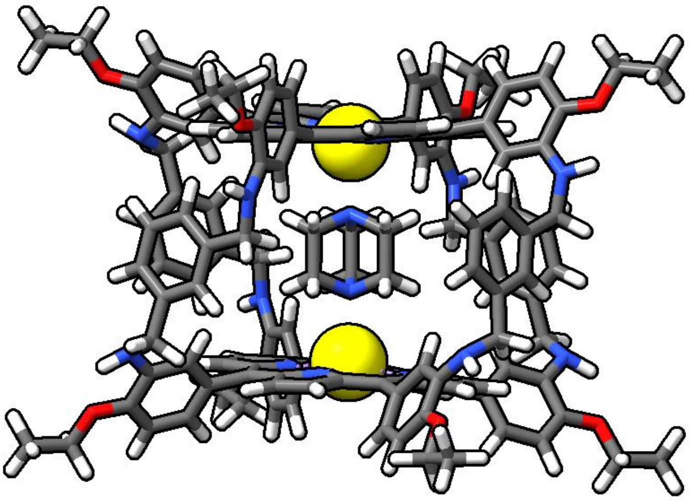

Figure S22D. Minimum-energy B3LYP/cc-PVDZ-optimized structure calculated for $\mathbf{2_{Zn}}^{NC}$·*DABCO*.

S23. Host-Guest Chemistry. Binding of *Group 2* guests (*bipy*) to $\mathbf{2}_{Zn}^{NC}$

Binding of guest molecules in *Group 2*, like 4,4'-bipyridine (*bipy*) was first evaluated through ^{1}H NMR titration experiments in $CDCl_3$ (Figure S23A). Binding of *bipy* is detected in slow NMR exchange (please look at the signals marked with yellow circles and blue squares, to better appreciate it), but the $\mathbf{2}_{Zn}^{NC}$ proton signals at substoichiometric amounts of guest are broad, which indicates a much faster exchange than with $\mathbf{1}_{Zn}^{NC}$ (please compare with Figure S7A).

^{1}H NMR Titrations

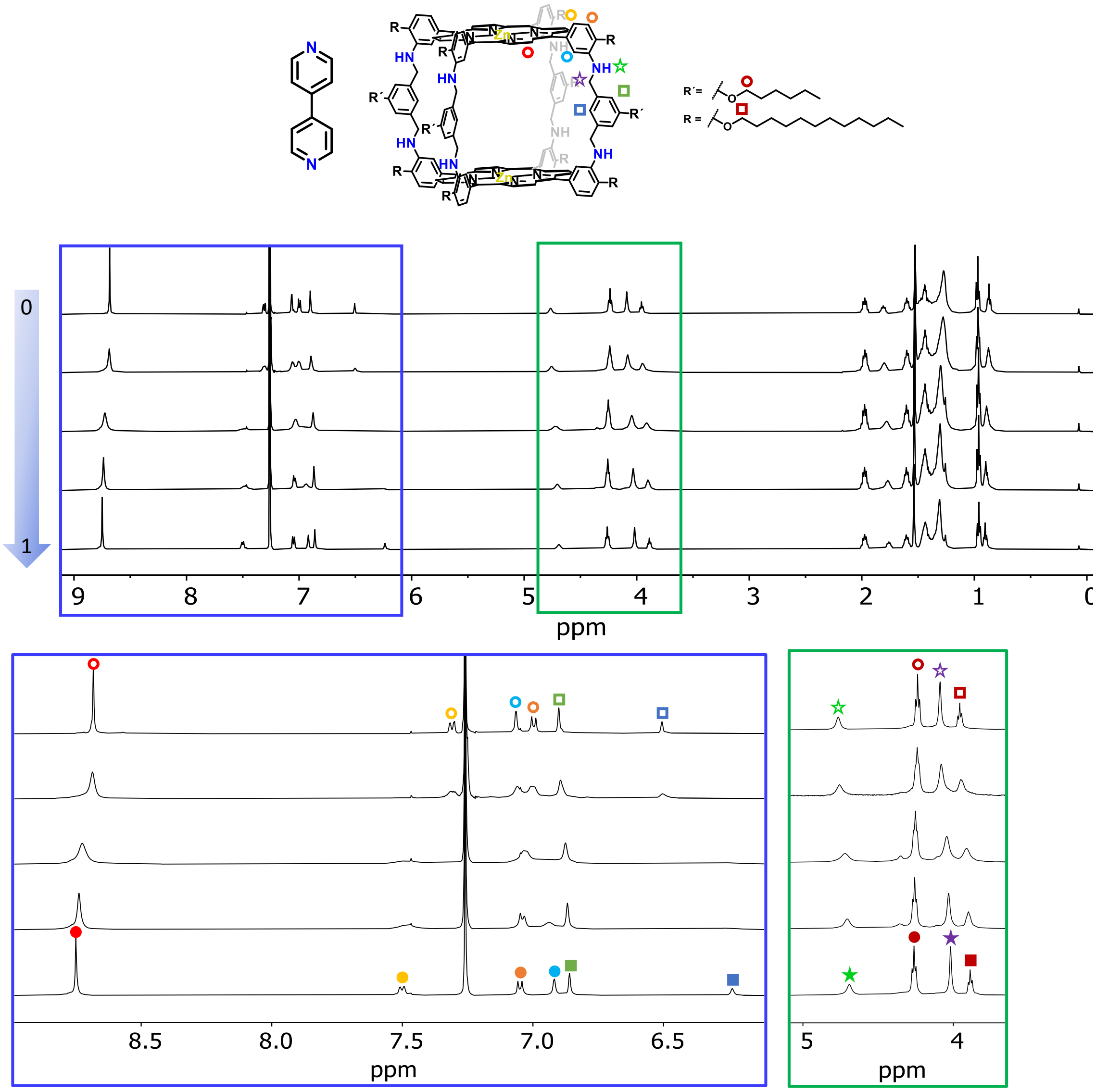

Figure S23A. ^{1}H NMR changes recorded along the titration of $\mathbf{2}_{Zn}^{NC}$ with increasing amounts of *bipy* in $CDCl_3$ at 298 K.

UV-vis Titrations

UV-vis titrations allowed us to determine the binding constant (K_a) between **2**$_{Zn}$^{NC} and *bipy* in $CHCl_3$, by fitting the binding isotherms to a 1:1 model.

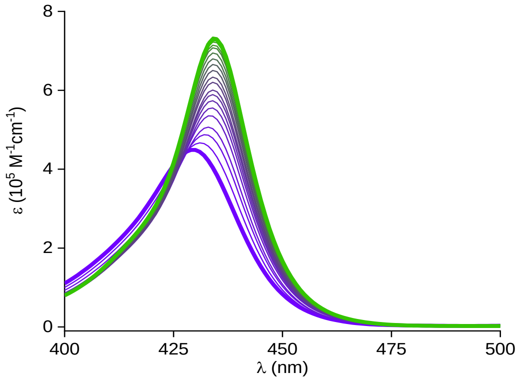

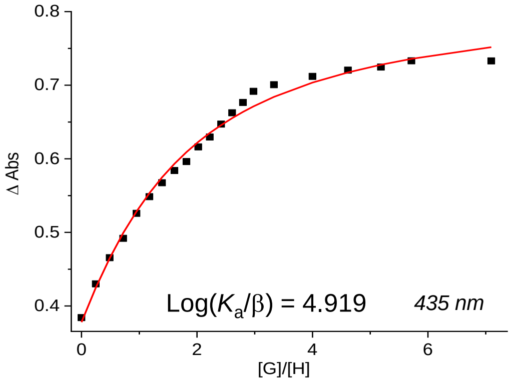

Figure S23B. UV-vis changes recorded along the titration of **2**$_{Zn}$^{NC} at a constant concentration of 1.0·10^{-5} M with increasing amounts of *bipy* in $CHCl_3$ at 298 K.

NOESY Experiments

In NOESY experiments in $CDCl_3$ (Figure S23C) very similar cross-peaks were observed for the **2**$_{Zn}$^{NC}·*bipy* complex than for the empty **2**$_{Zn}$^{NC} cage (please compare with Figure S20D).

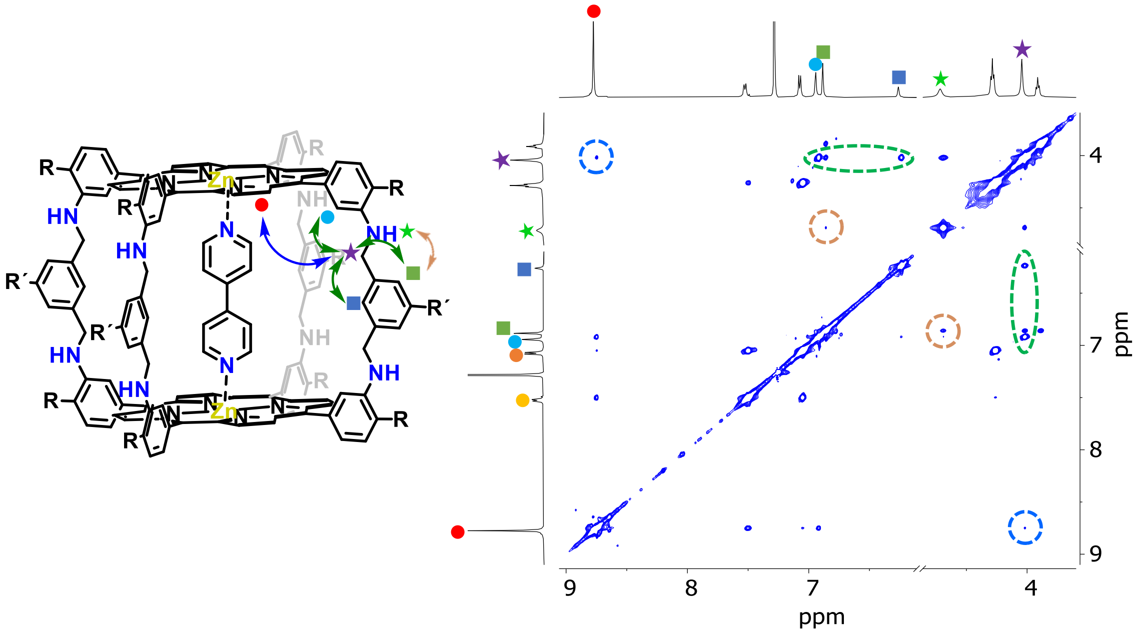

Figure S23C. 2D NOESY spectrum of the **2**$_{Zn}$^{NC}·*bipy* (1:1) complex in $CDCl_3$ at 298 K.

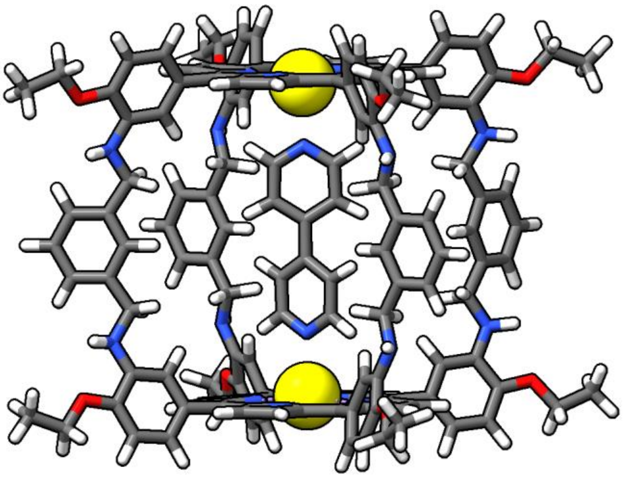

Figure S23D. Minimum-energy B3LYP/cc-PVDZ-optimized structure calculated for $\mathbf{2}_{\mathbf{Zn}}^{\boldsymbol{NC}}$·*bipy*.

S24. Host-Guest Chemistry. Binding of *Group 3* guests (*naphy*) to $\mathbf{2}_{Zn}^{NC}$

Binding of guest molecules in *Group 3*, like 2,6-naphthyridine (*naphy*), was first evaluated through ^{1}H NMR titration experiments in $CDCl_3$ (Figure S24A). Binding of *naphy* is detected in slow NMR exchange in $CDCl_3$, and the $\mathbf{2}_{Zn}^{NC}$ proton signals at substoichiometric amounts of guest are broad, which indicates a similar exchange dynamics than with $\mathbf{1}_{Zn}^{NC}$ (please compare with Figure S16A-1).

^{1}H NMR Titrations

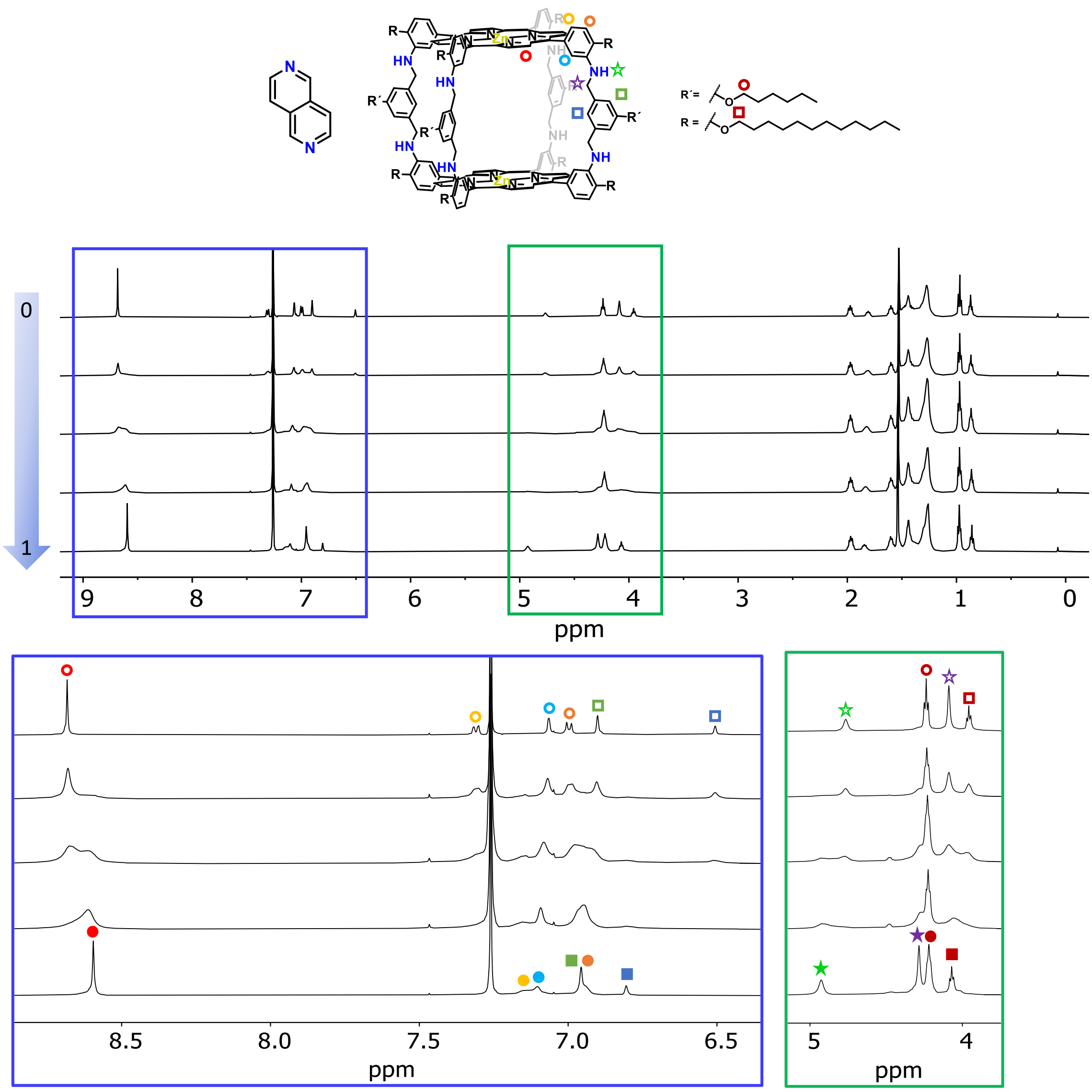

Figure S24A. ^{1}H NMR changes recorded along the titration of $\mathbf{2}_{Zn}^{NC}$ with increasing amounts of *naphy* in $CDCl_3$ at 298 K.

UV-vis Titrations

UV-vis titrations allowed us to determine the binding constant (K_a) between $\mathbf{2_{Zn}}^{NC}$ and *naphy* in $CHCl_3$, by fitting the binding isotherms to a 1:1 model.

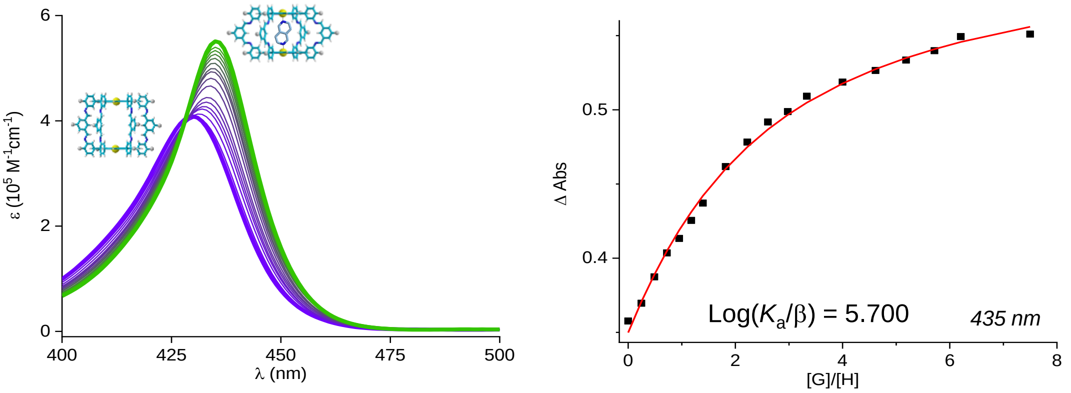

Figure S24B. UV-vis changes recorded along the titration of $\mathbf{2_{Zn}}^{NC}$ at a constant concentration of $1.0{\cdot}10^{-6}$ M with increasing amounts of *naphy* in $CHCl_3$ at 298 K.

NOESY Experiments

In NOESY experiments in $CDCl_3$ (Figure S24C) very similar cross-peaks were observed for the $\mathbf{2_{Zn}}^{NC}{\cdot}$*naphy* complex than for the empty $\mathbf{2_{Zn}}^{NC}$ cage (please compare with Figure S20D).

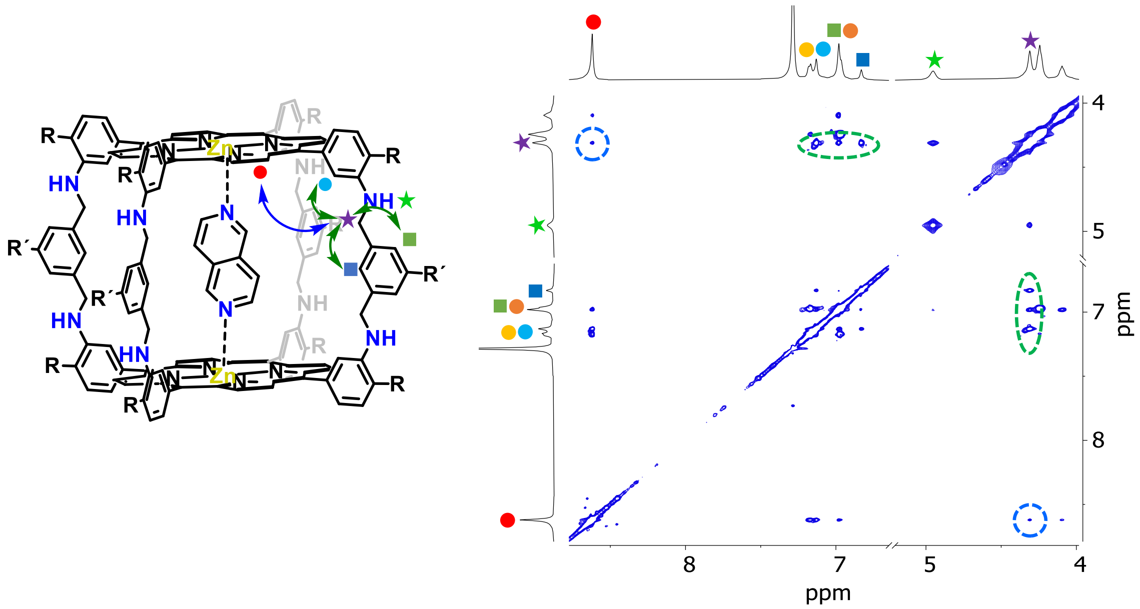

Figure S24C. 2D NOESY spectrum of the $\mathbf{2_{Zn}}^{NC}{\cdot}$*naphy* (1:1) complex in $CDCl_3$ at 298 K.

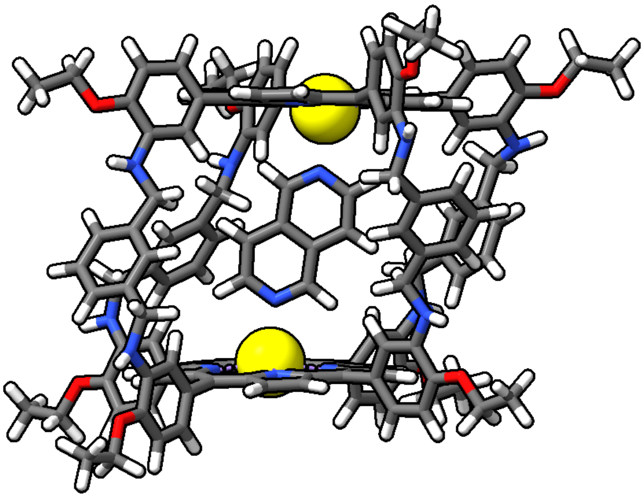

Figure S24D. Minimum-energy B3LYP/cc-PVDZ-optimized structure calculated for $\mathbf{2}_{\mathbf{Zn}}{}^{\boldsymbol{NC}}$·*naphy*.

S25. Non-selective performance of reduced cage

Two different experiments were carried out to prove the non-selective nature of **2_{Zn}^{NC}**, considering the ability of this cage to bind guest molecules of *Groups 2, 3* and *4*. Firstly, to a **2_{Zn}^{NC}** cage solution, 1.0 eq. of *bipy*, *naphy* and *pyr* were added in a stepwise manner (Figure S25a), resulting in a (1:1:1:1) mixture. Furthermore, to a **2_{Zn}^{NC}** cage solution, 1.0 eq. of *pyr*, *naphy* and *bipy* were added (Figure S25b). In both experiments the final situation was the same, a mixture of different host-guest complexes and free guest molecules.

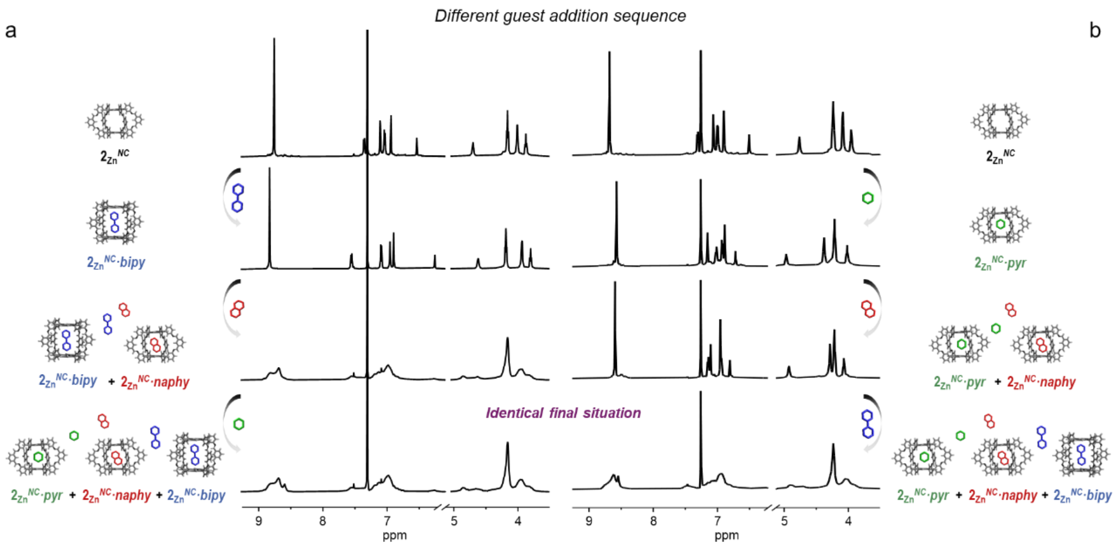

Figure S25. Changes observed in selected regions of the ^{1}H NMR spectrum of **2_{Zn}^{NC}** (1 mM in $CDCl_3$) upon sequential addition of (from top to bottom) 1 equivalent of (**a**) *bipy*, *naphy* and *pyr* or (**b**) *pyr*, *naphy* and *bipy* T = 298 K.

S26. Self-Sorting

In order to test the different size adaptability of **1_{Zn}^{NC}** and **1_{Zn}^{CN}** cages, self-sorting experiments were performed by preparing a 1:1:1:1 mixture of **1_{Zn}^{NC}**, **1_{Zn}^{CN}**, ***bipy***, and ***naphy*** following three different protocols.

Protocol A. To a 1:1 mixture ($n_{t\ cages}$ = 4.0·10^{-4} mol) of **1_{Zn}^{NC}** and 1_{Zn}^{CN} cages (top spectrum, black and grey NMR signals, respectively) 0.5 eq of ***bipy*** (n = 2.0·10^{-4} mol) was added, giving place to a 0.8:0.2:0.2:0.8 mixture of **1_{Zn}^{NC}**, 1_{Zn}^{CN}, **1_{Zn}^{NC}·*bipy***, and **1_{Zn}^{CN}·*bipy***. Upon the addition of 0.5 eq of ***naphy*** (n = 2.0·10^{-4} mol) to the solution the self-sorting process takes place reaching a new equilibrium composed mainly by **1_{Zn}^{NC}·*naphy*** and **1_{Zn}^{CN}·*bipy***, that were the most stable supramolecular complexes. However, it is possible to observe a 5% of **1_{Zn}^{NC}·*bipy*** in the mixture (green NMR signals in the middle spectrum).

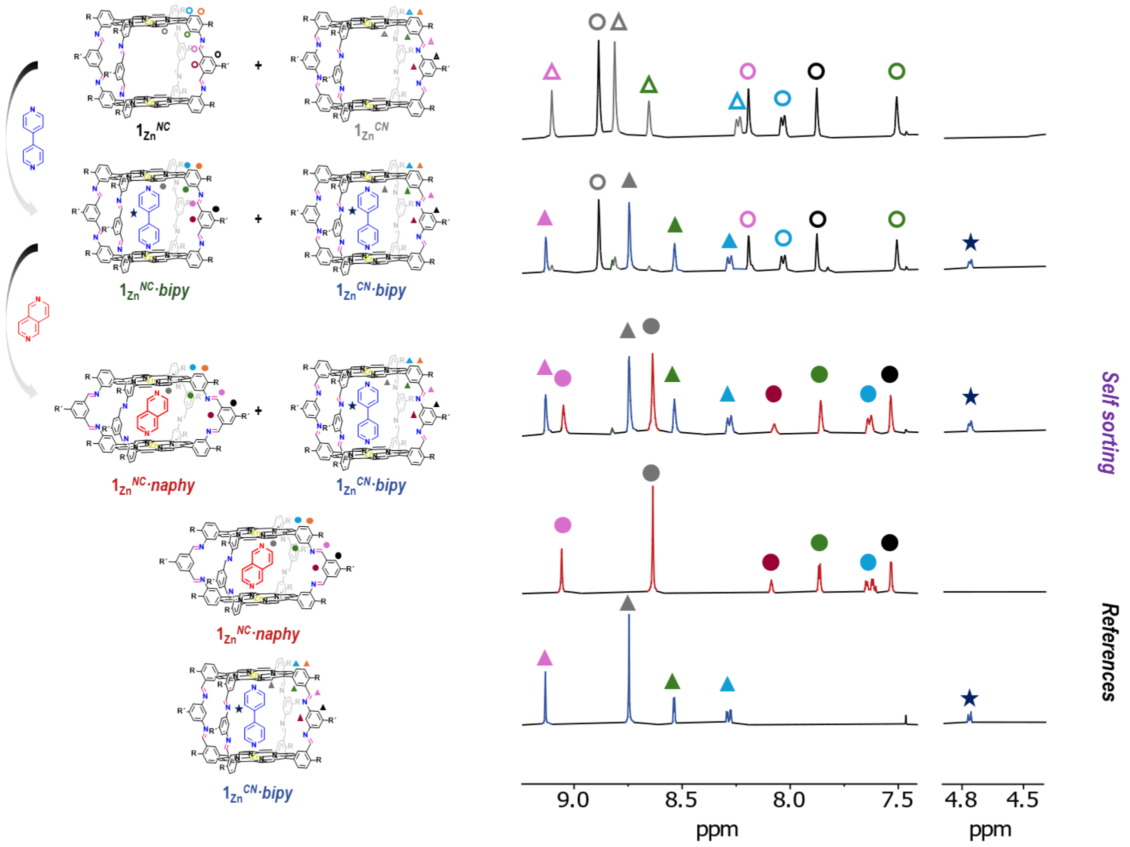

Figure S26A. ^{1}H NMR spectra recorded along the self-sorting experiment following approach 1. From top to bottom: a 1:1 mixture of $\mathbf{1_{Zn}}^{NC}$ and $\mathbf{1_{Zn}}^{CN}$ cages (represented by circles and triangles, respectively, total concentration $1 \cdot 10^{-3}$ M), upon addition of 0.5 eq of bipy, and upon addition of 0.5 eq of naphy (self-sorting) in $CDCl_3$ at 298 K. Reference spectra of the most stable supramolecular complexes are shown at the bottom of the figure.

Protocol B. Equivalent volumes of a **1**$_{Zn}^{NC}$***·bipy*** ($1\cdot10^{-3}$ M) and a **1**$_{Zn}^{CN}$***·naphy*** ($1\cdot10^{-3}$ M) solutions in $CDCl_3$, that are the less stable combination of supramolecular complexes, were mixed observing how the guest molecules were transferred from **1**$_{Zn}^{NC}$***·bipy*** and **1**$_{Zn}^{CN}$***·naphy*** to form **1**$_{Zn}^{NC}$***·naphy*** and **1**$_{Zn}^{CN}$***·bipy*** complexes, with a residual amount of **1**$_{Zn}^{NC}$***·bipy*** (5%) in the mixture (green NMR signals in the middle spectrum).

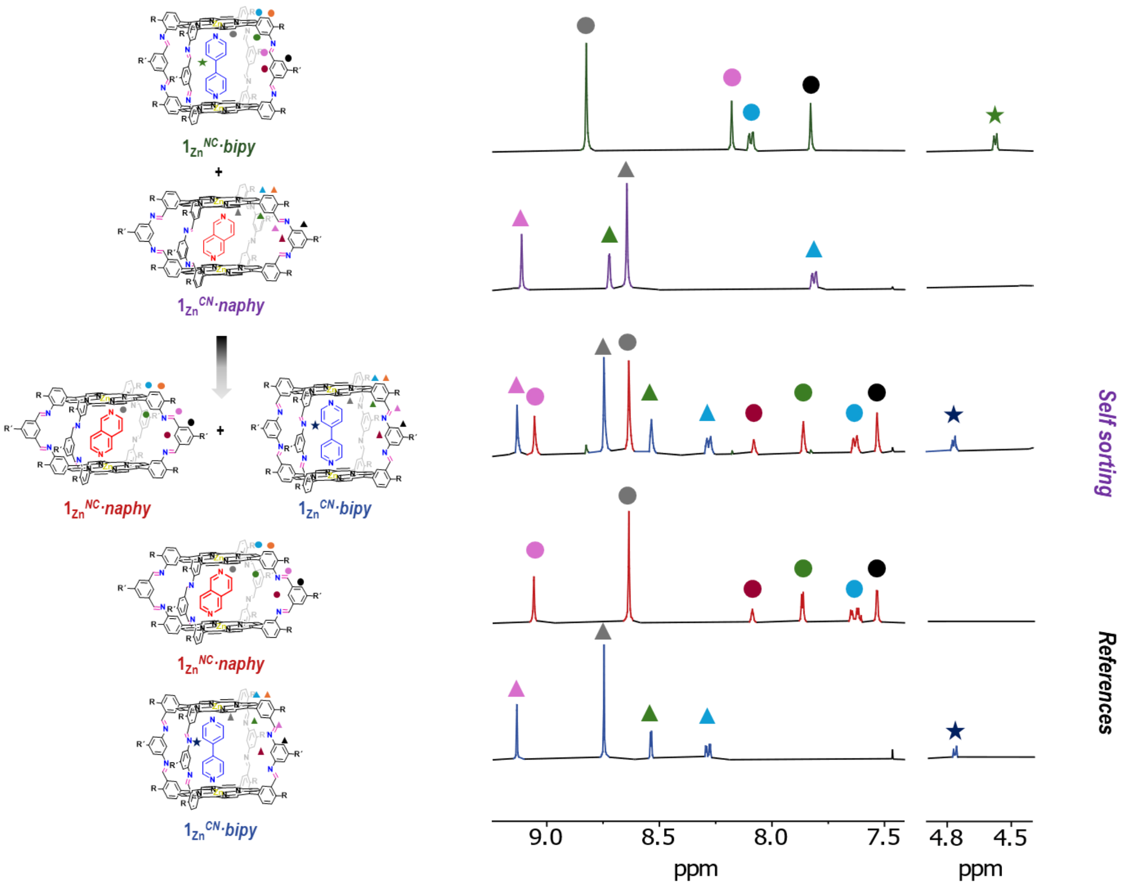

Figure S26B. ^{1}H NMR spectra recorded along the self-sorting experiment following approach 2. From top to bottom: **1**$_{Zn}^{NC}$***·bipy*** (concentration $1\cdot10^{-3}$ M), **1**$_{Zn}^{CN}$***·naphy*** (concentration $1\cdot10^{-3}$ M), and the result obtained upon mixing equimolar amount of **1**$_{Zn}^{NC}$***·bipy*** and **1**$_{Zn}^{CN}$***·naphy*** (self-sorting) in $CDCl_3$ at 298 K. Reference spectra of the most stable supramolecular complexes are shown at the bottom of the figure.

Protocol C. To a 1:1 mixture ($n_{t\ cages}$ = 4.0·10^{-4} mol) of $\mathbf{1}_{Zn}^{NC}$ and 1_{Zn}^{CN} cages (top spectrum, black and grey NMR signals, respectively) 0.5 eq of ***naphy*** (n = 2.0·10^{-4} mol) was added, obtaining a mixture of $\mathbf{1}_{Zn}^{NC}$, 1_{Zn}^{CN}, **1_{Zn}^{NC}·*naphy***, and **1_{Zn}^{CN}·*naphy*** which composition could not be quantify due to the complex behaviour exhibited in the formation of both **1_{Zn}^{NC}·*naphy***, and **1_{Zn}^{CN}·*naphy*** complexes (see in Figures S16A-1 and S17A-1). Upon the addition of 0.5 eq of ***bipy*** (n = 2.0·10^{-4} mol) to the solution the self-sorting process takes place reaching the same equilibrium shown above composed mainly by **1_{Zn}^{NC}·*naphy*** and **1_{Zn}^{CN}·*bipy***, with a 5% of **1_{Zn}^{NC}·*bipy*** in the mixture (green NMR signals in the middle spectrum).

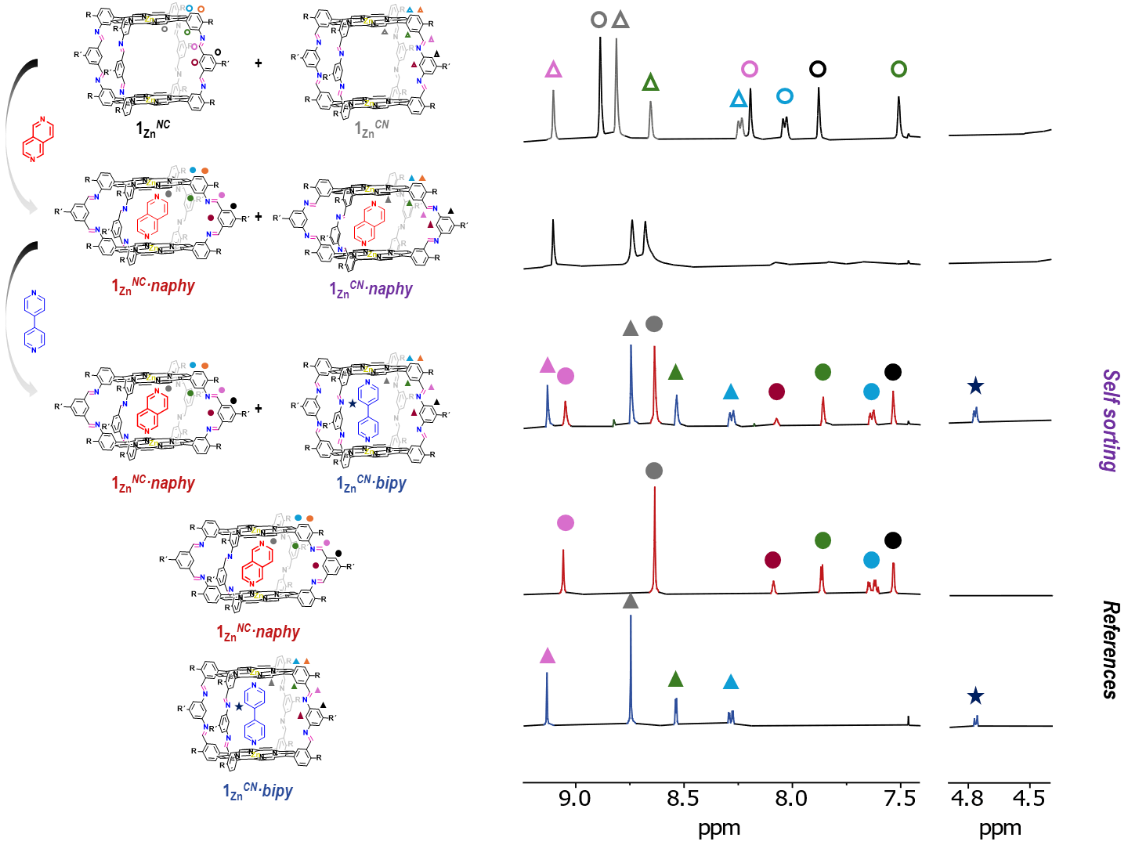

Figure S26C. ^{1}H NMR spectra recorded along the self-sorting experiment following approach 3. From top to bottom: a 1:1 mixture of $\mathbf{1}_{Zn}^{NC}$ and $\mathbf{1}_{Zn}^{CN}$ cages (total concentration 1·10^{-3} M), upon addition of 0.5 eq of naphy, and upon addition of 0.5 eq of bipy (self-sorting) in $CDCl_3$ at 298 K. Reference spectra of the most stable supramolecular complexes are shown at the bottom of the figure.

References

1) Zamora-Olivares D., Kaoud T. S., Dalby K. N., Anslyn E. V., In-Situ Generation of Differential Sensors that Fingerprint Kinases and the Cellular Response to Their Expression, *J. Am. Chem. Soc*., **2013**, *135*, 14814-14820.

2) Chen S., Zhang S., Bao C., Wang C., Lin Q., Zhu L., Oligo(aryl-triazole)s CH···Cl− interactions guide chloride efficient and selective transmembrane transport, *Chem. Commun.* ***2016**, 52, 13132-13135.*

3) Martí-Centelles V., Piskorz T. K., Duarte F., CageCavityCalc (C3): A Computational Tool for Calculating and Visualizing Cavities in Molecular Cages, *J. Chem. Inf. Model.* ***2024**, 64, 5604-5616.*

4) Frisch, M. J., Trucks, G. W., Schlegel, H. B., Scuseria, G. E., Robb, M. A., Cheeseman, J. R., et al. Gaussian 16, Revision C. 02. Wallingford, CT: Gaussian Inc. 2019.

5) Becke A. D., Density-functional thermochemistry. III. The role of exact Exchange, *The Journal of Chemical Physics* **1993**, *98*, 5648-5652.

6) Stephens, P. J., Devlin, F. J., Chabalowski, C. F., and Frisch, M. J. Ab initio calculations of vibrational absorption and circular dichroism spectra using density functional force fields. *J. Phys. Chem*. **1994**, 98, 11623–11627.

7) Dunning Jr., T. H., Gaussian basis sets for use in correlated molecular calculations. I. The atoms boron through neon and hydrogen. *J. Chem. Phys*. **1989**, 90, 1007-1023.

8) Grimme, S., Antony, J., Ehrlich, S., Krieg, H. A consistent and accurate ab initio parameterization of density functional dispersion correction (DFT-D) for the 94 elements H-Pu. *J. Chem. Phys*. **2010**, **132**, 154104.

9) Devillers C. H.; Dimé A. K.D.; Cattey H.; Lucas D., Crystallographic, spectroscopic and electrochemical characterization of pyridine adducts of magnesium(II) and zinc(II) porphine complexes, *Comptes Rendus Chimie* **2013**, *16*, 540-549;

10) Cook, L.P.; Brewer, G.; Wong-Ng, W., Structural Aspects of Porphyrins for Functional Materials Applications, *Crystals* **2017**, 7, 223-245.
